# CPSARST: an efficient circular permutation search tool applied to the detection of novel protein structural relationships

**DOI:** 10.1186/gb-2008-9-1-r11

**Published:** 2008-01-18

**Authors:** Wei-Cheng Lo, Ping-Chiang Lyu

**Affiliations:** 1 Institute of Bioinformatics and Structural Biology, National Tsing Hua University, Hsinchu 30013, Taiwan

## Abstract

Circular permutation of a protein can be visualized as if the original amino- and carboxyl termini were linked and new ones created elsewhere. It has been well-documented that circular permutants usually retain native structures and biological functions. Here we report CPSARST (Circular Permutation Search Aided by Ramachandran Sequential Transformation) to be an efficient database search tool. In this post-genomics era, when the amount of protein structural data is increasing exponentially, it provides a new way to rapidly detect novel relationships among proteins.

## Abstract 2

CPSARST (Circular Permutation Search Aided by Ramachandran Sequential Transformation) is an efficient database search tool that provides a new way for rapidly detecting novel relationships among proteins.

## Background

Circular permutation (CP) in a protein structure is the rearrangement of the amino acid sequence such that the amino- and carboxy-terminal regions are interchanged [1,2]. It can be visualized as if the original termini of the polypeptide were linked and new ones created elsewhere [3,4]. Since the first observation of naturally occurring circular permutations in plant lectins [5], a substantial number of natural examples have been reported, including some bacterial β-glucanases, swaposins, glucosyltransferases, β-glucosidases, SLH domains, transaldolases, C2 domains (for a review, see [6]), FMN-binding proteins [7], double-φ β-barrels [8], glutathione synthetases [9], DNA and other methyltransferases [1,10], ferredoxins [11], and proteinase inhibitors [12,13]. In most of the cases, circular permutants (CPs) have conserved function or enzymatic activity [6,14], sometimes with increased functional diversity [15-17].

To reveal the influences of CP on the structure, function and folding mechanism of proteins, many artificial CPs have been generated, inclusive of trypsin inhibitor, anthranilate isomerase, dihydrofolate reductase, T4 lysozyme, ribonucleases, aspartate transcarbamoylase, the α-spectrin SH3 domain, the *Escherichia coli *DsbA protein, ribosomal protein S6 and *Bacillus *β-glucanase [18,19]. The outcomes have indicated that three-dimensional structure seems remarkably insensitive to CP [6] and CPs generally retain their biological functions [3,4], although the structural stabilities, the folding nuclei, transition states or pathways might be altered [18,20,21]. Since CP generally preserves protein structure and function, with sometimes increased stability or activity, it has been applied to trigger crystallization [22], improve enzyme activities [15], determine critical elements [23,24], and create novel fusion proteins, the tethered sites of which are not confined to the native termini [25-28], such as the famous fluorescent calcium sensor [28].

In spite of these interesting properties and applications, there is still much uncertainty about the genetic mechanisms, the evolutionary importance and the natural prevalence of CP [6,18,29,30]. CPs can arise from posttranslational modifications [5,31] but a majority may arise from genetic events [29]. There have been several genetic and evolutionary mechanisms proposed, for instance, duplication/deletion models [6,32], duplication-by-permutation models [1,33], fusion/fission models [2,30], and plasmid-mediated 'cut and paste' [10]. However, which plays the major role or what proportion each mechanism contributes to the evolution of CPs and protein families remains uncertain. Besides, because of the disagreement between definitions of CPs, conflicting conclusions can be observed. In general, previous studies that considered the whole protein as the unit that undergoes CP concluded that CP is rare in nature [6,14,30] while those viewing the domain as the unit that undergoes CP suggested CP to be frequent [1,29,34].

In this post-genomic era, the amount of protein structure data is increasing exponentially, and plenty of information should be extractable to reveal the natural prevalence and evolutionary mechanism of CP; however, CP search tools are still very rare. It has been indicated that traditional sequence comparison methods are linearly sequential in nature and inefficient at identifying CP [6,35]. Three-dimensional structural comparisons may identify more evolutionarily far-related CPs [6]; nevertheless, conventional methods such as DALI [36] and CE [37] are also inefficient due to their sequential nature [34]. To detect CP, the most exact approach is to use an algorithm that generates all possible CPs of one protein and subsequently aligns them with another protein to find an alignment better than the linear alignment [2,38], although this is apparently very time-consuming. A few brilliant approaches have been developed to achieve higher efficiency. Uliel *et al*. [30,38] proposed a heuristic method based on duplicating one of the two protein sequences followed by manual verifications. Though being much faster, it still takes several CPU months to survey tens of thousands of sequences. The requirement of manual examinations also makes it unrealistic for searching large datasets [2]. Weiner *et al*. [2] condensed amino acid sequences into tiny domain strings to achieve an extremely high speed, scanning hundreds of thousands of sequences in hours; however, without suitable domain annotations or when a CP disrupts a domain, false negatives occur. Structural alignment methods applicable to the identification of CPs have also been developed. For instance, Jung and Lee [29] developed SHEBA to screen the SCOP database. They suggested that CPs are very frequent and many have symmetric structures. However, since internal symmetry may introduce noise into the detection of CPs [39], certain false positive predictions can be produced. Regardless of the capability of detecting distantly related CPs, a pair-wise comparison by structure-based CP-detecting algorithms may take from seconds to minutes [34], making routine database searches infeasible.

### Overview of CPSARST

**Figure 1:**
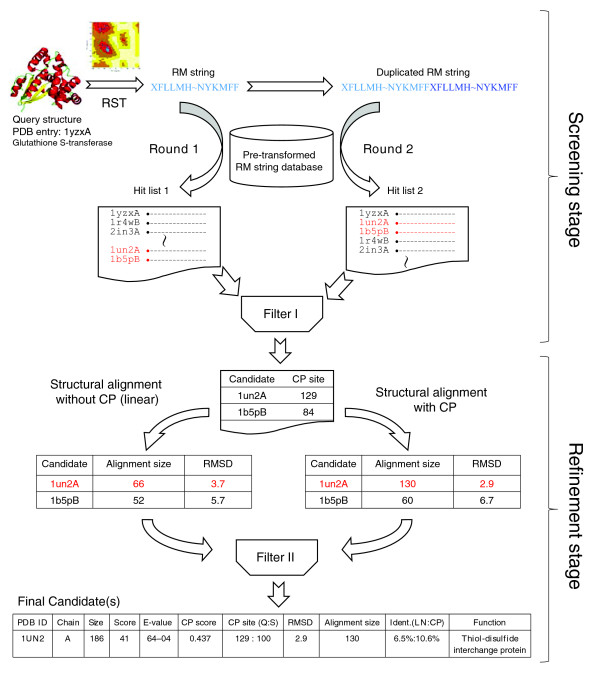
Flowchart of CPSARST. CPSARST uses a 'double filter-and-refine' strategy combining a fast screening and an accurate refinement step, each having two different rounds. In the screening stage, the three-dimensional structure of the query protein is transformed into a one-dimensional structural string by a RST algorithm [40]. This query string is subjected to two rounds of database searches. In round 1, it is searched against a pre-transformed structural string database by a heuristic method. In round 2, it is duplicated prior to the database search. Results of the two rounds are filtered; hits with meaningfully improved similarity scores are considered as CP candidates (colored red). In the refinement stage, candidates are analyzed by an accurate structural alignment algorithm, FAST [63], with and without CP manipulation, to determine their reliabilities and to retrieve permutation sites more precisely. After filtering out improbable cases, final answers with detailed information are output. The example used in this figure is a real case with simplified hit lists.

Here we present CPSARST (Circular Permutation Search Aided by Ramachandran Sequential Transformation), an efficient tool for searching for CPs. It describes three-dimensional protein structures as one-dimensional text strings by using a Ramachandran sequential transformation (RST) algorithm [40], which transforms protein structures through a Ramachandran (RM) map organized by nearest-neighbor clustering. This linear encoding methodology converts complicated and time-consuming structural comparison problems into string comparisons that can be done very rapidly. CPSARST has also achieved high efficiency by duplicating the query structure and working through a 'double filter-and-refine' strategy. These approaches are illustrated in Figure 1. A web service and a stand-alone Java program of CPSARST are available at [41]. CPSARST not only inherits the speed advantages of sequence-based methods but retains sensitivity to detect distantly related CPs mostly detectable only by structure-based methods. To the best of our knowledge, it is the first structural similarity search method that makes large scale all-against-all database searches for CP achievable and practicable. We suppose that this procedure can be applied to reveal the evolutionary importance of CP and detect novel protein structural relationships. Several novel CP relationships have been detected by CPSARST and are reported in this article; also, some rational estimations of the prevalence of CP in protein structural databases have been made by doing all-against-all database searches of non-redundant Protein Data Bank (PDB) and SCOP.

## Results

### Performance on random circular permutants

Although CPSARST basically uses structurally meaningful RM strings to search protein databases, its algorithm is actually applicable to amino acid sequences. To evaluate their amino acid sequence-based algorithm, Uliel *et al*. performed *in silico *random CP followed by various levels of regular mutations (substitutions, insertions and deletions) on a number of proteins [38]. We adapted this approach in a more thorough manner and developed a random CP dataset containing 20,000 chains (RCP dataset; see Materials and methods) to assess the performance of CPSARST with amino acid sequences. Two parameters were monitored: the proportion of cases in which the exact permutation site was retrieved; and the percentage distance of the retrieved permutation site to the exact one, which is defined as:

(1) $$D(\%)=\frac{\text{Number of residues off the exact permutation site}}{\text{Sequence length}}\times 100$$

**Figure 2:**
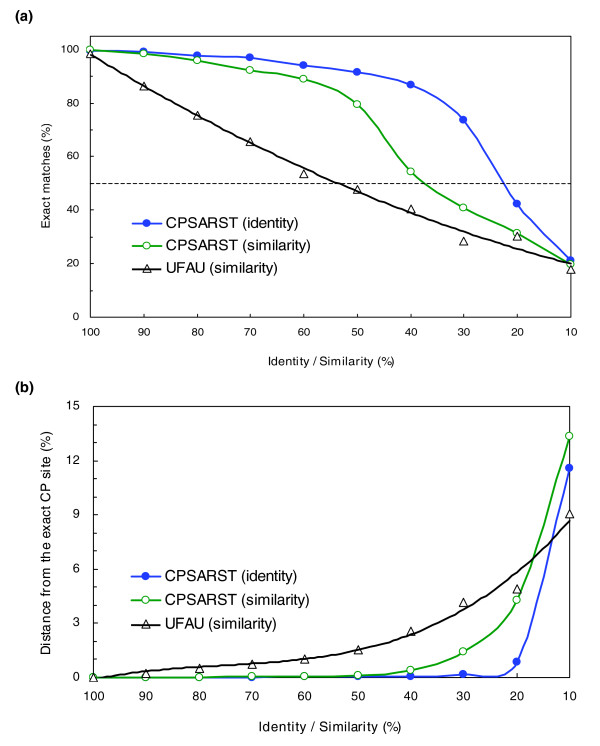
Performance on RCPs. The methodology of CPSARST is not only applicable to structurally meaningful RM strings but also to amino acid sequences. Random CP followed by various degrees of random substitutions, insertions and deletions were performed on 100 amino acid sequences. The performance of CPSARST was monitored by **(a) **the percentage of cases in which the exact permutation site was retrieved, and **(b) **the percentage distance of the retrieved permutation site to the exact one. The dashed line in (a) represents a 50% cut, above which more than half of the permutation sites were exactly predicted. When it only depends on amino acid sequences to detect CP, CPSARST can be reliable even if the identity is as low as 20%. UFAU stands for the CP-detecting method developed by Uliel *et al*. [38].

As shown in Figure 2a, the percentage of exact matched cases retrieved by CPSARST remains over 80% until the sequence identities fall between 40% and 30%. When we made a 50% exact matches cut, the results indicated CPSARST ensures that at least 50% of the retrieved cases are exact as long as the sequence identities are higher than 22%.

The curve of the percentage distance of CPSARST has a half hyperbolic shape (Figure 2b). Provided that the sequence identity is > 20%, the percentage distance will be < 1%. Combining these data, we suggest that when our approach is applied to amino acid sequences, it will be reliable in detecting CPs with sequence identities as low as about 20%.

### Accuracy evaluations with engineered circular permutants

Since there are many artificial CPs, each with a definite parent protein, a known permutation site, and sometimes some regular mutations, they provide a good resource to assess the performance of a CP search method. We used keyword searches to find the engineered CPs recorded in the PDB [42], and subjected them to CPSARST searches. As summarized in Table 1, among the 15 non-redundant cases, all the parent proteins were successfully retrieved. Their average percentage distance is only 0.08%, which means that the CP sites identified are very close to the exact ones, demonstrating the high accuracy of CPSARST for engineered CPs.

**Table 1:** Retrieved parent proteins of engineered CPs by CPSARST

PDB entry	Chain	Size	Function	Parent structure/recorded CP site	Retrieved structure/determined CP site	*D *(%)*
1AJK	A,B	214	Circularly permuted (1-3,1-4)-beta-D-glucan 4-glucanohydrolase H	2AYH/84	2AYH/84	0.00
1AJO	A,B	214	Circularly permuted (1-3,1-4)-beta-D-glucan 4-glucanohydrolase H	2AYH/127	2AYH/127	0.00
1ALQ		266	CP254 beta-lactamase	3BLM/254	3BLM/254	0.00
1BD7	A,B	176	Circularly permuted BB2-crystallin	1BLBC/87	1BLBC/87	0.00
1CPM		214	Glucanase	2AYH/59	2AYH/59	0.00
1CPN		208	Glucanase	2AYH/59	2AYH/59	0.00
1FW8	A	416	Phosphoglycerate kinase	3PGK/72	3PGK/73	0.24
1G2B	A	62	Spectrin alpha chain	1SHG/47	1SHG/47	0.00
1N02	A	102	Cyanovirin-N	2EZM/50	2EZM/51	0.98
1P5C	A-D	167	Lysozyme	1LW9A/12	1LW9A/12	0.00
1SWF	A-D	128	Circularly permuted core-streptavidin E51/A46	1STP/51	1STP/51	0.00
1SWG	A-D	128	Circularly permuted core-streptavidin E51/A46	1STP/51	1STP/51	0.00
1TUC		63	alpha-Spectrin	1SHG/20	1SHG/20	0.00
1TUD		62	alpha-Spectrin	1SHG/48	1SHG/48	0.00
1UN2	A	197	Thiol-disulfide interchange protein	1A2J/100	1A2J/100	0.00
Average						0.08

*Percentage distance of the retrieved permutation site to the exact one. See text for definition.

### Pair-wise comparisons of naturally occurring circular permutants

To our knowledge, current CP-detecting methods based on structural comparisons work in only a pair-wise fashion. Although CPSARST is a database search procedure, it can be simplified to perform pair-wise comparisons (see Materials and methods). Here, we used naturally occurring CP candidates to test the performance of CPSARST. These candidate pairs were detected by doing all-against-all searches against a non-redundant PDB dataset (see below for details) and then filtering out engineered permutants. The 'structural diversity' defined by Lu [43] that integrates the concepts of normalized alignment size and root mean square distance (RMSD) was used to evaluate the quality of pair-wise comparisons:

(2) $$\text{structure diversity}=\frac{\text{RMSD}}{{\text{(}\frac{\text{alignment size}}{{\text{avg(N}}_{\text{q}}{\text{,N}}_{\text{s}}\text{)}}\text{)}}^{\text{1.5}}}$$

where avg(N_q_, N_s_) is the average size of the query and subject protein. Lower structural diversities stand for higher structural alignment qualities of the assessed methods. The results are listed in Tables 2 and 3. In terms of structural diversity, the performance of CPSARST is better than that of SHEBA [11] and is comparable to SAMO [34]. In addition, CPSARST is 9.3 times faster than SAMO in these pair-wise comparisons (Table 2). Protein size has no effect on the alignment qualities of these structure-based methods while the running time increases as the size becomes larger. This increase in running time is lowest for CPSARST, apparently much lower than that of SAMO. Sequence identities greatly influence the performance, especially for SHEBA (Table 3). The differences in structural diversities calculated by CPSARST and SAMO are not obvious until the sequence identity of the CP pair becomes lower than 20%.

**Table 2:** Performance of pair-wise comparisons for natural candidate CP pairs over various protein sizes

Length of the query protein (residues)	No. of candidate CP pairs	CPSARST		SHEBA		SAMO	
				
		Structural diversity	Average running time (s)	Structural diversity	Average running time (s)	Structural diversity	Average running time (s)
≤ 100	135	5.269	0.245	6.600	0.506	4.024	0.765
100-150	223	6.629	0.381	10.255	0.767	4.359	2.243
150-200	464	6.105	0.520	12.730	0.955	4.591	3.554
200-250	177	4.410	0.922	10.683	1.390	3.499	6.793
250-300	39	6.645	1.063	11.092	1.774	4.277	10.820
> 300	30	6.918	1.894	6.976	2.224	4.423	22.345
Average			0.838		1.269		7.753

**Table 3:** Performance of pair-wise comparisons for natural candidate CP pairs over various sequence identities

Identity (%)	No. of candidate CP pairs	Structural diversity		
		
		CPSARST	SHEBA	SAMO
≤ 10	823	6.309	11.180	4.396
10-20	152	5.864	13.881	4.994
20-30	11	3.581	4.506	3.363
30-40	33	1.868	3.284	2.210
40-50	40	1.755	3.096	1.544
> 50	9	1.385	2.247	1.520

CPSARST runs very rapidly in pair-wise comparisons. When searching databases, its speed will be even higher since it does not work in a pair-wise manner but with a 'double filter-and-refine' strategy. Chen had estimated that using SAMO to compare two proteins mostly took around ten seconds [34]. Searching the current PDB (approximately 90,000 polypeptides) by one-against-all comparisons will, therefore, require over 15,000 minutes. However, CPSARST can do this one-against-all comparison in 1.7 minutes (see below). As shown by these naturally occurring cases, CPSARST achieves a high speed with a reasonable compromise in alignment accuracy.

### Protein structural database searches

To examine the database searching performance of CPSARST, two non-redundant protein databases were used, the 90% sequence identity subsets of PDB (January 2007) and the ASTRAL SCOP dataset (v.1.71) [44], which were abbreviated as nrPDB-90 (14,422 polypeptides) and nrSCOP-90 (11,688 domains), respectively (see Additional data files 1 and 2 for lists of entry IDs). As summarized in Table 4, the all-against-all survey of large protein databases like nrPDB-90 took 65.7 hours. Since there were approximately 200 million protein pairs for this database (14,422 × 14,422), these data demonstrated that CPSARST could scan around 52,800 pairs per minute. At this speed, a full search of the current PDB could be finished in 1.7 minutes per query protein. In comparison with 6.4 minutes required by the sequence-based UFAU method (developed by S Uliel, A Fliess, A Amir and R Unger) [38] and 15,000 minutes by the structure-based SAMO [34], CPSARST runs fairly fast. Besides, CPSARST gives the user two parameters, expectation value (E-value) and CP score, to evaluate the significance of the retrieved information.

**Table 4:** Statistics of protein structural database searches

Database	nrPDB-90	nrSCOP-90
No. of proteins	14,422	11,688
No. of candidate pairs		
Detected by amino acid sequence	5,020	1,802
Detected only by Ramachandran string	252,287	196,533
Confirmed after the refinement stage		
Total	2,911	4,228
Symmetric CP	682	1,161
Total no. of protein pairs	208.0 × 10^6^	136.6 × 10^6^
Total running time (minutes)	3,942	1,974
No. of protein pairs scanned per minute	52,764	69,204

As a database search method, CPSARST provides a list of hits ranked by the statistically meaningful E-value. Given that a hit has a similarity score *S*, the E-value is the number of different alignments with scores equivalent to or better than *S *that are expected to occur in this particular database search by chance [45-47]. A lower E-value indicates a higher significance for the score. This statistical significance is a useful indicator of the reliability of the search results.

To determine the extent to which two proteins are related by a CP, we used the CP scoring scheme described by Vesterstrom and Taylor [39]. The minimum value of this CP score is -1 for a pair of completely linearly aligned proteins, and its maximum value is 1 for a perfect CP alignment. In general, a small positive CP score indicates that only a small fraction of the protein is permutated while a larger one reveals that the CP site is closer to the middle of the polypeptide chain.

In the survey of nrPDB-90 and nrSCOP-90, we had set the RMSD cutoff as 5 Å, the E-value cutoff as 0.1 and the CP score threshold as 0.2. Under these criteria, 2,911 and 4,228 candidate pairs were identified in nrPDB-90 and nrSCOP-90, respectively. For nrPDB-90, the 2,911 candidate pairs consisted of 1,822 different polypeptides, that is 12.6% (1,822 of 14,422) of the polypeptides have CP relationships with at least one other polypeptide. For nrSCOP-90, the proportion is 17.6% (2,060 of 11,688).

### Novel circular permutation family detected by CPSARST

**Figure 3:**
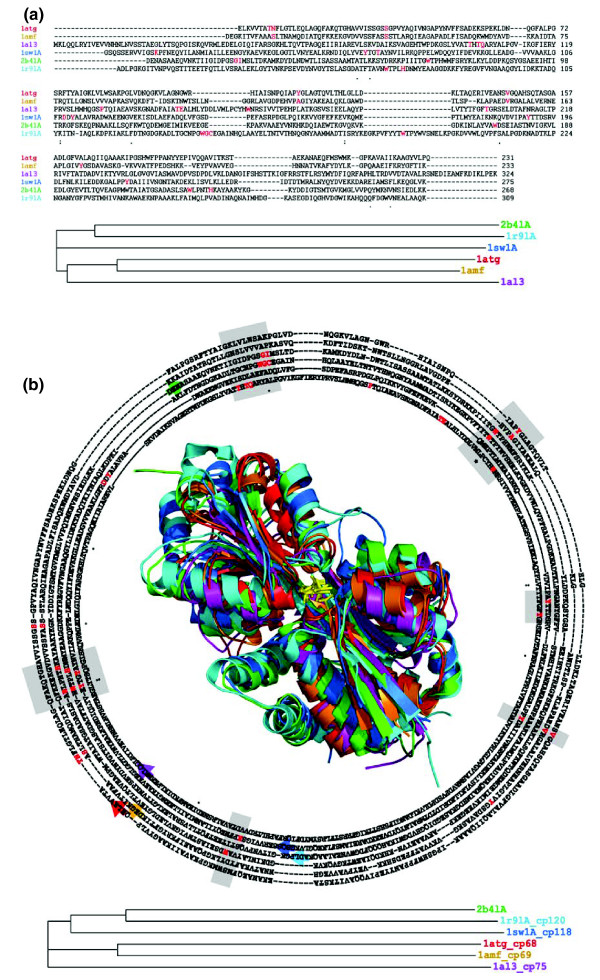
A novel CP family detected by CPSARST. Entries 2b4lA ([PDB:2B4L], chain A), 1r9lA ([PDB:1R9L], chain A) and 1sw1A ([PDB:1SW1], chain A) are GBBPs. Entries 1atg ([PDB:1ATG]) and 1amf ([PDB:1AMF]) are molybdate-binding proteins (MoBPs) and 1al3 ([PDB:1AL3]) is the cysteine regulon transcriptional activator CysB from *Klebsiella aerogenes*. Any pair of these proteins share < 24% sequence identity (calculated by FASTA [48]). **(a) **Multiple sequence alignment of these GBBPs, MoBPs and CysB does not well reveal their functional and evolutionary relationships. Residues interacting with the ligands [65-67] are colored red; they are rather scattered. GBBPs and MoBPs are basically ligand transporters while CysB is a transcriptional regulator; however, the phylogram tree built from this alignment correlates CysB and MoBPs into the same branch and the three GBBPs are separated into two branches; these evolutionary relationships do not agree with their functional relatedness. **(b) **Multiple circularly permuted sequence alignment and structural superimposition of these six proteins. The numbers after '_cp' following PDB entry IDs stand for the residue numbers of the new amino termini after circular permutations, which are indicated by colored arrows. The ligand-interacting residues are better clustered in this alignment (gray regions) and the phylogram tree agrees well with the functional relatedness. The image of the superimposed proteins shows that these proteins have similar overall structures and the positions of their ligand-binding pockets are conserved (ligands are shown as yellow stick models); the colors used in this image are the same as in the alignment text and phylogram tree. Structures shown in this report were all drawn by using PyMOL [68]. Multiple sequence alignments and the tree building were performed by Clustal W [69].

After visual inspections of superimposed CP pairs detected by CPSARST, we found that it is possible for proteins with very different functions and divergent amino acid sequences to share CP relationships structurally, forming novel CP families, which are difficult to identify using conventional comparison methods. For instance, although glycine betaine-binding proteins (GBBPs), molybdate-binding proteins and *Klebsiella aerogenes *cysteine regulon transcriptional activator CysB share similar overall structures when judged by the naked eye, their sequence identity is low (< 24%; calculated by FASTA [48]) and structural relatedness is hard to detect by conventional methods (Figure 3). CPSARST detected CP relationships among GBBPs themselves and among these three groups of proteins. To our knowledge, these CP relationships have not been reported previously. Figure 3 illustrates that the functional and evolutionary relationships among these proteins cannot be correctly determined by their raw sequences; their ligand-interacting residues are not well-aligned and proteins with more similar functions are separated while those with less similar functions cluster together in the phylogram tree. However, the circularly permuted sequences retrieved by CPSARST can be well-aligned and the phylogram tree agrees with the functional relatedness among these proteins. A superimposition of six of these proteins is also shown in Figure 3 to demonstrate their structural similarity and the conserved position of their ligand binding pockets.

### Circular permutants detected by CPSARST

We examined the candidate pairs detected by CPSARST with RMSD ≤ 3.5 Å by visual inspection of superimposed structures and found that approximately 55%, 25% and 20% are mainly alpha, mainly beta, and alpha-beta structures, respectively. These CP pairs are listed, each with a superimposed image, in Additional data file 3; many well-known CP cases are listed, such as some lectins, glucanases, transaldolases, methyltransferases, ferredoxins, protease inhibitors and GTPases. Furthermore, a large number of these CP relationships have not been reported yet, for example, chorismate mutases ([PDB:1CSM] versus [PDB:2AO2]); some (approximately 20%) even involve hypothetical proteins, implying that CPSARST can be applied to suggest possible functions for hypothetical proteins.

**Figure 4:**
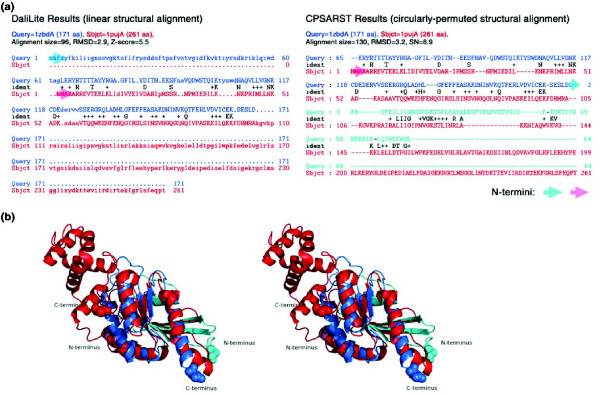
CP relationship between GTPase and hypothetical protein YlqF. Rab3A ([PDB:1ZBD], chain A) is a small G protein with GTPase activity [49] while YlqF ([PDB:1PUJ], chain A) is a conserved hypothetical protein from *B. subtilis*. **(a) **These two proteins can be structurally aligned by DALI [36] only partially (left); however, CPSARST detects their CP relationship (right). If the 64 residue amino-terminal region of Rab3A (in cyan text) is permuted to the carboxul terminus, it can be extensively aligned to YlqF with an RMSD of 3.2 Å (right). The transparent cyan and pink arrows indicate the amino termini of Rab3A and YlqF, respectively. **(b) **The superimposition of Rab3A and YlqF made by CPSARST (cross-eye stereo view). Colors are the same as in (a). Residues shown as cyan/pink and blue/red spacefill models are the amino and carboxyl termini, respectively.

Rat Rab3A is a small G protein with GTPase activity [49]. CPSARST detected that it has a CP relationship with a conserved hypothetical protein YlqF from *Bacillus subtilis*, the structure of which was determined by the New York Structural Genomics Research Consortium. When we searched with YlqF against the PDB using the DALI server [50], a number of isomerases, elongation factors, G proteins, transferases and other hypothetical proteins with inconvincible quality of structural alignments, i.e. small alignment sizes and large RMSD, were returned (Additional data file 4). However, CPSARST detected that many G proteins superimpose well with YlqF, suggesting that it may possess GTP binding/GTPase activity (Table 5). Figure 4 shows that DALI can only partially align Rab3A and YlqF (alignment size, 96; RMSD, 2.9 Å), while CPSARST successfully detects the CP relationship between them (alignment size, 130; RMSD, 3.2 Å).

**Table 5:** Top 20 CP relationships detected from the nrPDB-90 dataset for hypothetical protein YlqF*

No.	PDB entry/size	E-value	RMSD/Alignment size	Function
1	1ZBD/203	4.00E-13	3.17/130	Rabphilin-3A
2	1KY2/182	4.00E-13	3.07/122	GTP-binding
3	2F7S/217	4.00E-13	3.52/125	Ras-related protein Rab-27B protein YPT7P
4	2NZJ/175	8.00E-13	2.94/123	GTP-binding protein REM 1
5	1T91/207	9.00E-13	3.06/123	Ras-related protein Rab-7
6	1X3S/195	2.00E-12	2.80/117	Ras-related protein Rab-18
7	1YU9/175	6.00E-12	2.70/123	GTP-binding protein, GTPase domain
8	2EW1/201	6.00E-12	2.74/128	Ras-related protein Rab-30
9	2GF9/189	7.00E-12	2.89/126	Ras-related protein Rab-3D
10	1YVD/169	8.00E-12	2.12/123	Ras-related protein Rab-22A
11	1PUI/210	1.00E-11	3.00/130	Probable GTP-binding protein engB
12	2O52/200	1.00E-11	2.92/127	Ras-related protein Rab-4B
13	1U8Y/168	1.00E-11	2.81/110	Ras-related protein Ral-A
14	1HUQ/164	1.00E-11	2.80/123	Rab5C, GTPase domain
15	2HUP/201	1.00E-11	3.11/129	Ras-related protein Rab-43
16	1FZQ/181	1.00E-11	2.58/123	ADP-ribosylation factor-like protein 3
17	2OCB/180	3.00E-11	2.78/121	Ras-related protein Rab-9B
18	1OIV/191	4.00E-11	2.81/121	Ras-related protein Rab-11A
19	2FN4/181	4.00E-11	3.11/129	Ras-related protein R-Ras
20	1Z0F/179	6.00E-11	3.04/121	Rab14, member Ras oncogene family

*YlqF ([PDB:1PUJ], chain A) is a conserved hypothetical protein from *B. subtilis*. This structure was determined by the New York Structural Genomics Research Consortium (NYSGRC).

**Figure 5:**
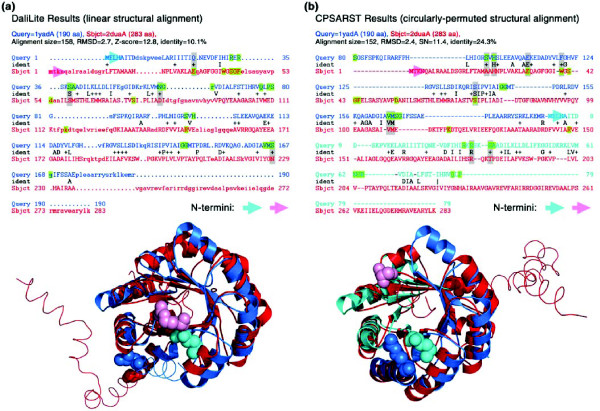
Symmetric CP with significant sequence clues. Proteins with symmetric structure may have symmetric CPs [29]. *B. subtilis *thiaminase I ([PDB:1YAD]) [51] and *Variovorax sp. Pal2 *phosphonopyruvate hydrolase ([PDB:2DUA]) [52] shown here are symmetric TIM-barrel proteins. Although their structures can be well-aligned both by linear and CP alignments, significant sequence conservation is observed only in the latter. **(a) **Linear alignment performed by DALI [36]. The upper text demonstrates that the sequence identity calculated from these structurally aligned residues is 10.1%. Ligand-interacting residues in both proteins are highlighted green; four of them are aligned with identical or physiochemically similar amino acids (gray highlighted strips). The lower image is the superimposition of these two structures. Terminal unaligned regions are shown as ribbons to make the spatial closeness of the termini more easily observable. In this linear alignment, the amino termini of the two proteins are close to each other, as are the carboxyl termini. **(b) **CP relationship detected by CPSARST. After CP, the sequence identity significantly rises to 24.3% and there are nine ligand-interacting residues aligned with identical or similar amino acids. The amino- and carboxy-terminal halves of 1yadA bounded by the putative CP site are colored cyan and blue, respectively. The orientation of 1yadA in the superimposed image is the same as that in (a). In this CP alignment, the amino and carboxyl termini of the two proteins are separated, a feature of symmetric CP.

Jung and Lee [29] suggested that when a pair of proteins can be well-aligned, with or without CP of the sequences, they are symmetric CPs. Considering this definition, proteins containing repeats or duplications will be included. However, Uliel *et al*. [30] supposed that these should be differentiated from true CPs. In our point of view, the certification of a CP relationship between symmetric proteins is conditional upon the observation of a reasonable increase in sequence homology after the CP. For instance, *B. subtilis *thiaminase I [51] and *Variovorax sp. Pal2 *phosphonopyruvate hydrolase [52] are a pair of symmetric TIM-barrel proteins detected by CPSARST that superimpose well, with (alignment size, 151; RMSD, 2.4 Å) or without (alignment size, 158; RMSD, 2.7 Å) CP. Their sequence identity rises from 10.1% to 24.3% upon CP. As shown in Figure 5, their ligand-interacting residues are not well-aligned without CP while, for each protein, these functionally important residues can be aligned with physiochemically related amino acids on the other protein with CP. Therefore, we suggest that this is a true CP case.

## Discussion

### Detecting circular permutants with low sequence identities

Generally speaking, although protein similarity search methods based on amino acid sequence alignments are much faster than those based on structural comparisons, they are less sensitive in detecting remote homology [53]. In the case of detecting CP, sequence-based methods have met great challenges because of the evolutionary complexity and diversity of circular permutants. Except the post-translational modification model, all the other proposed mechanisms for CP involve at least two stages of genetic modifications in evolution (see Background), implying that the formation of CP may require a long period during which other common mutations (substitutions, insertions and deletions) can accumulate to such an extent that the circular permutants have much diverged from the parent protein in sequence. Therefore, sequence-based methods may be limited in identifying distantly related CPs. For instance, Uliel *et al*. used an amino acid sequence-based heuristic algorithm to screen the entire Swiss-Prot database (version 34.0; approximately 80,000 proteins) and the Pfam database [54] for CP pairs, and identified only 32 cases [30]. However, in the same year, Jung and Lee [29] used a structure-based algorithm to survey a protein dataset (3,035 domains) collected from SCOP and reported that approximately 47% (1,433 of 3,035) of the domains each had at least one circular permutant. Furthermore, they discovered that less than 0.3% of the abundant symmetric CPs have > 30% sequence identities. Although this large difference is partially caused by the fact that Uliel *et al*. used more stringent criteria to identify CP, it basically indicates that amino acid sequence-based methods can miss many distantly related CPs [34].

Among the CP candidate pairs detected by CPSARST in nrSCOP-90, 27.5% can be considered as symmetric CPs (Table 4). Similar to the observation of Jung and Lee, few of these symmetric CPs (2.6%) have sequence identities > 30%. Furthermore, although 91% of the naturally occurring CP pairs listed in Table 2 have sequence identities ≤ 20%, CPSARST shows good performance when compared with other structure-based methods. These data demonstrate that CPSARST is able to detect CPs with low sequence identities.

### Speed improvements

In most cases, it is not easy to achieve high accuracy and speed simultaneously for a database search method; instead, some compromising balance is usually reached. Judging from the fact that using previous structure-based CP-detecting methods such as SAMO to search the current PDB requires more than 15,000 minutes [34] per query, it is reasonable that speed should be weighted more than accuracy in the field of CP searching, especially in this post-genomic era when the amount of protein structural data is increasing rapidly. CPSARST has been shown to achieve accuracy substantially higher than sequence-based UFAU (Figure 2) and comparable to structure-based SAMO (Tables 2 and 3); as to the speed, it can scan 52,800 database proteins per minute (Table 4), approximately 4 and 8,824 times faster than UFAU and SAMO, respectively. This improvement in speed is achieved by two features: it transforms three-dimensional information of protein structures into one-dimensional text strings and, thus, converts structural comparison problems into text sequence alignment problems, which can be solved much more rapidly; and, in both the screening and refinement stages, CPSARST does not stick to the absolute qualities of the alignments. By focusing on the relative qualities between two rounds of alignments, it can rapidly sieve out useful information. We call this strategy 'double filter-and-refine'. Here we propose that it is efficient, flexible and applicable to other biological research fields, especially where the data analyses require large-scale computational power.

### The prevalence and definition of circular permutation

Previous studies have made conflicting conclusions; some presumed that CP is rare in nature [6,14,30] - approximately 5% as indicated by Vogel and Morea [14] - while others supposed that CP is frequent [1,29,34] - approximately 47% as estimated by Jung and Lee [29]. In our observation, studies based on structural analyses usually discovered more CPs than sequence-based ones; besides, studies that consider the whole protein as the unit that undergoes CP would conclude that CP is rare whereas those viewing the domain as the unit that undergoes CP would suggest CP to be frequent.

As we have discussed, it is reasonable that more cases of CP are detected by structural comparison than by amino acid sequence alignment. However, although proteins with similar structures are usually functionally related [55], when a pair of structurally and functionally similar proteins share extremely low sequence identity, we still cannot exclude the possibility that they are just the products of convergent evolution [56-58] and do not share the same origin. In the case of identifying CP, it is noteworthy that even if a pair of proteins shows a high extent of CP topologically, it does not directly mean that an evolutionary CP event has indeed taken place. Therefore, we argue that detecting CP only by structure would result in too many false positives when judged from the point of view of molecular evolution. This is why we have set up a user-adjustable sequence identity filter in the web service of CPSARST [41] (see Materials and methods). When this filter was not enabled, the prevalence of CP estimated by CPSARST was 12.6-17.6% (see Results). When we considered that a real CP should have a higher sequence identity in the CP alignment than in the linear alignment, around one-fourth of the candidate pairs counted in Table 4 was filtered out, lowering the estimated prevalence of CP to 9.0-13.0%.

The fact that the frequency of CP estimated by CPSARST is only one-third of that estimated by Jung and Lee [29] is probably because of the more stringent criteria used by CPSARST. We set the RMSD cutoff as 5 Å, the CP score threshold as 0.2 and the least permutation size as 20% for a pair of proteins to be considered as CP candidates; similar criteria were not seen in the report of Jung and Lee. Also, considering their methodology, there is a large likelihood that proteins containing repeats and duplications are regarded as CPs, many of which have been treated as false cases by Uliel *et al*. [30] and us (see Materials and methods). When we loosened the criteria to 6 Å (RMSD cutoff), 0.1 (CP score threshold) and 10% (least permutation size), and did not filter out proteins containing repeats, the CP prevalence estimated by CPSARST was 34.7-36.7% (see Additional data file 5 for statistics), similar to Jung and Lee's estimation. However, since they did not provide any supplementary list of their CP candidates, we are unable to check our speculation.

To our knowledge, all the currently available CP-detecting methods are more sensitive to global CP (the unit undergoing CP is the whole protein) than partial CP (the CP is within a region of the protein), as is CPSARST. To detect partial CP, domain databases such as SCOP and Pfam are usually used as the target databases instead of the PDB and Swiss-Prot. Although considering the domain as the unit undergoing CP, that is, partial CP, can identify more candidates (as shown in Table 4), some scientists have argued that these cases should be considered as 'swaps' rather than CPs [30]. This controversy is another cause of the conflicting conclusions about the prevalence of CP.

To sum up, despite the conflicting conclusions made by previous studies, there seem to be rational explanations for this situation. We suppose that the identification of CPs requires a precise definition of CP depending on the purpose of the study. In our opinion, if evolutionary importance and mechanisms are concerned, global CP with reasonable sequence identity limitation will be suitable, while partial CP without limitation of sequence identity in the definition may help scientists to discover novel functional relationships among proteins and to reveal the principles of protein folding.

### Possible applications of CPSARST

The performance of CPSARST suggests that it is an efficient approach to the detection of CPs in large protein structural datasets; routine bank-against-bank searches are thus achievable. The multiple indexes produced by CPSARST, for example, the structural similarity score, statistically meaningful E-value, sequence identity, alignment size, RMSD and CP score, are beneficial to develop automated procedures such as a functional assignment system for novel hypothetical proteins. Also, information retrieved by bank-against-bank searches can be organized into a CP database.

Since the first observation of CP in plant lectins [5], many natural and artificial cases have been studied and several CP detecting methods have been developed; however, there is still no CP database and no standard procedure for evaluating CP detection methods. We suppose that a well-organized CP database will help move this field forward. It could provide a standard for the evaluations of CP-related programs, such as CP search tools and predictors of viable CP sites [59], and provide information to reveal the evolutionary mechanisms of CP.

CP has been applied to X-ray crystallography [22], modification of enzymes [15], creation of novel fusion proteins [25,28], and construction of protein switches and sensors [26,27]. All these applications depend on a proper choice of position to create CP. A CP database offering plenty of materials for the discovery of the rules by which Nature selects CP sites should be advantageous to the technical applications of CP.

Although interesting, there is still much uncertainty about the evolutionary mechanisms and importance of CP [6,18,29,30]. Weiner *et al*. [60] have proposed that the frequency of incomplete or intermediate CP may help determine the major mechanism of CP. The 'double filter-and-refine' strategy of CPSARST is very flexible. With extended boundary criteria, CPSARST can specifically detect incomplete or intermediate CP. The ability of CPSARST to perform rapid bank-against-bank searches by structural comparisons gives it the potential to reveal how, why and to what extent Nature achieves protein evolutionary and functional diversity by using CP.

## Conclusion

We have developed an efficient circular permutation search method, CPSARST, which linearly encodes protein structures as text strings and achieves a structural similarity searching speed thousands of times as high as related algorithms. When tested with engineered CPs, CPSARST successfully retrieved all the natural proteins with accurate permutation site predictions. Its ability to identify natural CPs is also comparable to other structure-based CP-detecting methods. Its high efficiency makes routine database surveys and bank-against-bank searches achievable. After all-against-all searches of non-redundant PDB and SCOP, we have found that most candidate CP pairs share sequence identity < 20%, explaining why previous sequence-based CP-detecting methods have identified much less CP cases than structure-based algorithms. Based on these search results, we have suggested that the identification of CPs requires a suitable definition of CP depending on the purpose of the study. If global CP with reasonable sequence identity limitation is considered as true CP, the prevalence of CP in protein structural databases is estimated to be 16% by CPSARST, whereas the prevalence of partial CP without limitation of sequence identity in the definition is estimated to be 36%. Several new CP cases have been detected and reported here, inclusive of a novel CP family consisting of microbial GBBPs, molybdate-binding proteins and a cysteine regulon transcriptional activator. In this post-genomics era, when the amount of protein structural data is increasing exponentially, CPASRST can provide a new way to rapidly detect novel relationships among proteins and help to reveal how Nature achieves protein evolutionary and functional diversity by using CP. Its web service and stand-alone Java program are available at [41].

## Materials and methods

All the developments and experiments were performed on an IBM e-server 336 machine with dual 3.2GHz Intel processors, 1 GB RAM and linux operating system.

### Linear encoding of protein structures

CPSARST describes three-dimensional protein structures as one-dimensional strings by using a RST algorithm [40]. The torsion angles (*φ*,*δ*) of a number of proteins were plotted onto a 10° × 10° dissected RM map. The 1,296 cells on this map were then clustered into 22 groups by nearest-neighbor clustering [61] based on their spot numbers and angular distances. These groups were assigned a set of English letters called 'Ramachandran codes'. Coordinates of a protein structure could be accordingly transformed into a text string. The scoring matrix for these codes was produced by using a 'regenerative approach' [40]. This linear encoding system converts complicated and time-consuming structural comparison problems into sequence comparisons, which can be done very rapidly. It has been applied to protein structural similarity searching and achieved speeds hundreds of thousands of times higher than CE with an acceptable compromise of accuracy [40]. The structural string generated by RST is different from the amino acid sequence in nature; therefore, we termed it 'Ramachandran sequence' or 'Ramachandran string'.

### Generation and analyses of random circular permutants

A hundred polypeptide sequences each longer than 100 residues and sharing < 40% sequence identities were randomly selected from the PDB to perform *in silico *circular permutations. Regular mutations, i.e. substitutions, insertions and deletions, were introduced in the ratio 150:1:1 to generate random CPs, resulting in 100 levels of decreasing sequence identities/similarities for every polypeptide sequence. The collection of these computer-generated random CPs is called the RCP dataset.

The substitution rates of various amino acids used to generate the RCP dataset were calculated by analyzing a large number of multiple alignment blocks, the sequences of which shared < 45% identity, as described previously [62]. Since every sequence in the RCP dataset was evolved independently to avoid any possible bias, we supposed that it is suitable for the evaluation of CP detection methods. RCP has two subsets, the identity subset and similarity subset, each containing 10,000 CP pairs (100 parent sequences × 100 circular permutants). They are listed in Additional data file 6.

Comparisons between each parent sequence and its CPs in the identity subset of the RCP dataset were performed by the traditional heuristic method blast [45]. Two parameters were monitored to assess the performance: the percentage of cases in which the exact permutation site was retrieved; and the average percentage distance of the found permutation site to the exact one (see Results). Another two parameters were monitored to optimize the filter for RM sequence searches: the ratio of similarity scores and the negative logarithm in base 10 (-log_10_) of the E-value ratios, before and after the duplication of query sequences (see Additional data file 7 for the results). We found that all the score ratios are equal to or higher than 1, indicating that when the sequence of a CP is duplicated (DL), it always aligns to its parent sequence better than the normal length (NL). As to the E-value ratios, that is, -log_10_(*E-value*_*DL*_/*E-value*_*NL*_), approximately 80% of them are larger than 2, which stands for a 10^2^-fold improvement of the significance of the similarity score after duplicating the query sequence (see Results for detailed information about E-values).

### Screening of circular permutant candidates

It has been supposed that using heuristic methods like blast to search for CPs is difficult because an unambiguous reconstruction of the alignment results is problematic [38]. CPSARST, however, overcomes this problem by duplicating the query structure, doing two rounds (with and without the duplication) of database searches, and analyzing the results mutually. The hits with improved alignment qualities are picked as CP candidates, the permutation sites of which can be easily determined from the alignment results of duplicated sequences. In the screening stage, the search results of RM strings were filtered with simple criteria referring to previous studies and our experimental results on RCP amino acid sequences mentioned above. The permutation site should be at between 20% and 80% along the length of the query protein, ensuring a significant permutation size (20%). It has been supposed that a tiny permutation size is unlikely a real CP [39]. The size of the candidate could be different from that of the query protein by at most 50% because proteins of very different sizes are improbable candidates for CPs [38]. The similarity score of the duplicated query string (*Score*_*DL*_) should be higher than that of the normal query string (*Score*_*NL*_), and the -log_10 _value of the E-value ratio should be larger than -0.5 (see Additional data file 7 for detailed information about these settings):

(3) $$\frac{Score_{DL}}{Score_{NL}}>1$$

(4) $$-\log_{10}(\frac{Evalue_{DL}}{Evalue_{NL}})>-0.5$$

### Refinement of the search results

The refinement of search results of RM sequences were performed by FAST, an accurate structural alignment algorithm [40,63] and a CP scoring scheme developed by Vesterstrom and Taylor [39], following these steps. Step 1: for each candidate, the putative permutation site is parsed from the alignment result of the duplicated query string. Step 2: performing two rounds of FAST structural alignments. The first round is a normal linear alignment. In the second round, the circularly permuted alignment, the PDB file of the query structure was manipulated by exchanging the amino- and carboxy-terminal halves according to the putative permutation site so that FAST will do the structural alignment 'backside first'. Step 3: if the FAST alignment size after CP is no larger than 50% of the smaller size of the query and subject proteins, it is screened out. Step 4: the RMSD cutoff of the CP alignment is set as 5 Å. Step 5: in order to differentiate true CP from protein with internal repeats or duplications, two criteria have been set: the alignment size of the CP alignment should be larger than that of the linear alignment; and the FAST similarity score [63] or TOP score [43] (see formula (2)) calculated from the CP alignment should gain at least 25% improvement over the linear alignment. Step 6: the CP score [39] was calculated from the aligned positions by FAST. It has a theoretical minimum value of -1 (a completely linear alignment) and a maximum value of 1 (a perfect CP). Although Vesterstrom and Taylor suggested that an alignment with this CP score higher than 0.25 can be considered as a significant CP, we find that 0.2 is still suitable in our multi-filter system. Step 7: the putative CP site is refined by parsing the output of FAST structural alignment.

### Pair-wise circularly-permuted structural alignments

The procedure of the database search tool CPSARST can be simplified to perform pair-wise structure alignments as follows. First, transform the query and subject protein structures into RM sequences Q and S, respectively. Second, duplicate Q string to QQ, and align it to S. Third, find the best local alignment and trace it back to the 'start point', which is the putative permutation site. For example, if in the best local alignment, the fragment between residues *q*_1 _and *q*_2 _of Q is aligned to the fragment between *s*_1 _and *s*_2 _of S, then the permutation site of Q will be traced back to *q*_1 _- *s*_1 _+ 1. Fourth, introduce a CP into the query structure according to the putative CP site. Compare this new structure with the subject protein by using an accurate structural alignment algorithm mentioned above.

### Implementation

CPSARST basically works on the structurally meaningful RM strings transformed by RST; however, since there have been many errors and inconsistencies reported in PDB entries [64], a few polypeptides (approximately 2%) cannot be successfully transformed into RM strings. Therefore, in the implementation of CPSARST, we have added two extra rounds of amino acid sequence alignment searches, one by the normal length and the other by the duplicated sequence, prior to the RM string searches. Besides, the sequence homology filter can be enabled to guarantee a higher evolutionary significance of the search results (see Discussion), and several parameters are adjustable by the users according their needs or the property of materials.

#### Word size and gap penalties

These are traditional parameters used by sequence alignment search tools such as BLAST [45] and FASTA [48]. For CPSARST, a smaller word size can provide a more accurate determination of the CP site while taking more running time. In our experience, lower gap penalties can give CPSARST higher sensitivity, although there is a trade-off for running time, too. Generally speaking, these parameters have only minor effects on the performance.

#### Permutation size limit and circular permutation score threshold

It has been supposed that a tiny permutation size is unlikely a real CP [39], but there is yet no common conclusion made for the generally suitable permutation size limit. Setting a large limit ensures that CPSARST identifies unambiguous CP relationships; however, novel cases can thus be missed. If the query protein is large enough, for example, > 150 residues, a small size limit such as 10% may still work well, but we would like to suggest a 15% limit for general situations. The CP score threshold has similar effects on the search quality of CPSARST to the permutation size limit (see Results and Materials and methods for further information).

#### RMSD cutoff and structural similarity improvement filter

Closer-related protein structures will have a lower RMSD when superimposed. This is also true for CPs. This cutoff can be used as a basic quality control in the same way as other conventional structural comparison tools. The normalized structural similarity score of FAST [63] is another basic quality control. Candidate pairs without enough improvement in structural similarity after CP can be screened out.

Examples of practical settings for these parameters can be found in Additional data file 8. CPSARST is available at [41].

## Abbreviations

CP, circular permutation; CPs, circular permutants; CPSARST, Circular Permutation Search Aided by Ramachandran Sequential Transformation; DL, duplicated; GBBP, glycine betaine-binding protein; NL, normal length; PDB, Protein Data Bank; RM, Ramachandran; RCP, random circular permutation; RMSD, root mean square distance; RST, Ramachandran sequential transformation.

## Authors' contributions

WCL designed and carried out this study and drafted the manuscript. PCL conceived the study, participated in its design and helped to draft the manuscript.

## Additional data files

The following additional data are available with the online version of this paper. Additional data file 1 lists the nrPDB-90 dataset, the 90% sequence identity subset of the PDB (January 2007). Additional data file 2 lists the nrSCOP-90 dataset, the 90% sequence identity subset of SCOP (v.1.71). Additional data file 3 is a table listing candidate CP pairs in the nrPDB-90 dataset detected by CPSARST with RMSD ≤ 3.5 Å. Additional data file 4 is a list of the structural neighbors of the hypothetical protein YlqF in PDB retrieved by DALI [50]. Additional data file 5 is a table showing statistical results of protein structural database searches with broad criteria by CPSARST. Additional data file 6 lists the RCP dataset, a collection of 20,000 *in silico *random CPs. Additional data file 7 is a plot summarizing the score and E-value ratios calculated from the RCP dataset. Additional data file 8 is a list of the parameter settings used throughout this article.

## Supplementary Material

Additional data file 1 The 90% sequence identity subset of the PDB (January 2007). Click here for file

Additional data file 2 The 90% sequence identity subset of SCOP (v.1.71). Click here for file

Additional data file 3 Protein structures shown in this large table were drawn by using Chime [70]. Click here for file

Additional data file 4 Structural neighbors of the hypothetical protein YlqF in PDB retrieved by DALI [50]. Click here for file

Additional data file 5 Statistical results of protein structural database searches with broad criteria. Click here for file

Additional data file 6 A collection of 20,000 *in silico *random CPs. Click here for file

Additional data file 7 Score and E-value ratios calculated from the RCP dataset. Click here for file

Additional data file 8 Parameter settings used throughout this article. Click here for file
