# Supplementary material for: CPSARST: an efficient circular permutation search tool applied to the detection of novel protein structural relationships
Source: Genome Biol. 2008 Jan 18;9(1):R11. doi: 10.1186/gb-2008-9-1-r11 (PMC2395249; doi:10.1186/gb-2008-9-1-r11)
Supplement: Additional data file 3 — Protein structures shown in this large table were drawn by using Chime [70]. [file gb-2008-9-1-r11-S3.pdf]

CPSARST – An efficient circular permutation search tool applied to the detection of novel protein structural relationships

Wei-Cheng Lo and Ping-Chiang Lyu
Institute of Bioinformatics and Structural Biology, National Tsing Hua University, Hsinchu, Taiwan

Additional data file 3 – Candidate CP pairs in nrPDB-90†

Description: Candidate CP pairs in nrPDB-90 detected by CPSARST with RMSD ≤ 3.5 Å.
†The 90% sequence identity non-redundant subset of Protein Data Bank (Jan., 2007).

No.	PDB ID:chain (size)	Function	CP site	Score	E-value	CP score	RMSD	Aligned residues	Identity (LN:CP)	Superimposed Structures
1	121p:_ (166)	H-RAS P21 PROTEIN	67	46	2E-06	0.24	2.79	123	10.8% : 17.9% (9/83 : 22/123)	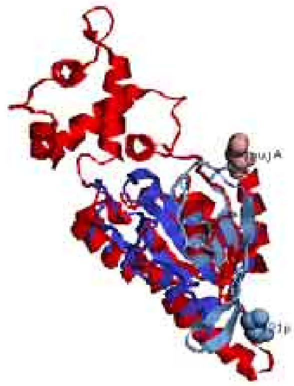
	1puj:A (262)	conserved hypothetical protein ylqF								
2	1a0b:_ (117)	AEROBIC RESPIRATION CONTROL SENSOR PROTEIN AR	77	35	0.003	0.35	3.06	75	7.8% : 6.7% (4/51 : 5/75)	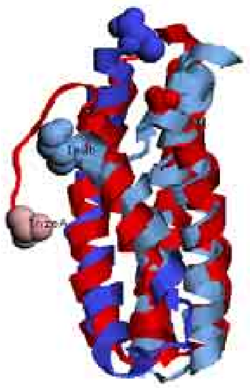
	1nze:A (112)	Oxygen-evolving enhancer protein 3								

3	* 1g2b:A (63)	SPECTRIN ALPHA CHAIN	**17**	45	3E-06	0.62	1.42	52	35.9% : 34.6% (14/39 : 18/52)	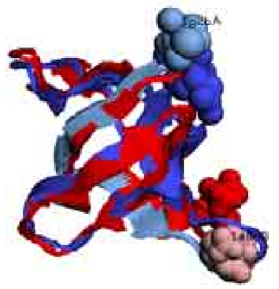
	* 1a0n:B (61)	FYN								
4	* 1a0n:B (69)	FYN	**17**	37	0.0009	0.50	1.42	49	30.8% : 36.7% (12/39 : 18/49)	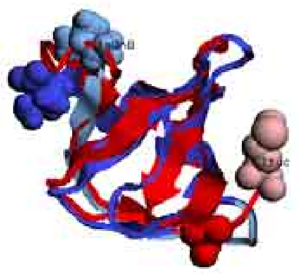
	* 1tuc_ (63)	ALPHA-SPECTRIN								
5	1a22:A (182)	GROWTH HORMONE	**64**	36	0.002	0.34	3.32	115	19.0% : 7.8% (11/58 : 9/115)	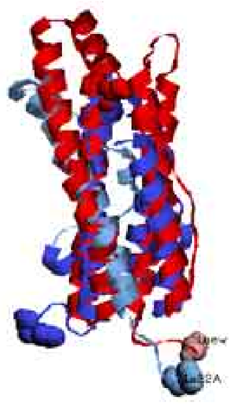
	1aew_ (170)	FERRITIN								

6	1dps:A (159)	DPS	**48**	38	0.0005	0.31	3.09	104	13.7% : 6.7% (10/73 : 7/104)	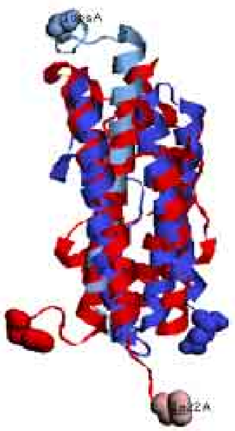
	1a22:A (182)	GROWTH HORMONE								
7	1a22:A (182)	GROWTH HORMONE	**72**	34	0.01	0.34	3.32	110	9.8% : 7.3% (6/61 : 8/110)	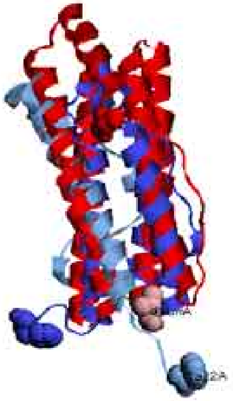
	1eum:A (161)	FERRITIN 1								
8	1a22:A (182)	GROWTH HORMONE	**64**	39	0.0003	0.32	3.28	107	10.7% : 10.3% (9/84 : 11/107)	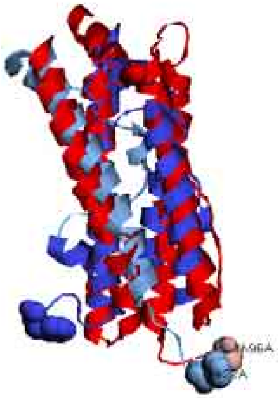
	1h96:A (167)	FERRITIN LIGHT CHAIN 1								

9	1a22:A (182)	GROWTH HORMONE	**69**	34	0.008	0.34	3.40	113	11.4% : 7.1% (9/79 : 8/113)	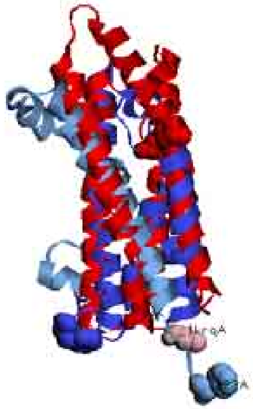
	1krq:A (164)	ferritin								
10	1mfr:A (171)	M FERRITIN	**119**	37	0.001	0.32	3.38	112	8.6% : 8.0% (7/81 : 9/112)	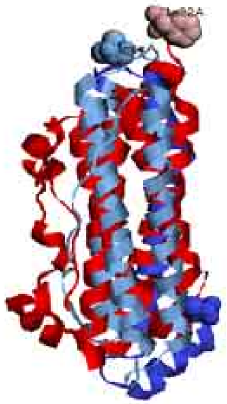
	1a22:A (182)	GROWTH HORMONE								
11	1a22:A (182)	GROWTH HORMONE	**103**	34	0.008	0.25	2.73	83	8.6% : 7.2% (6/70 : 6/83)	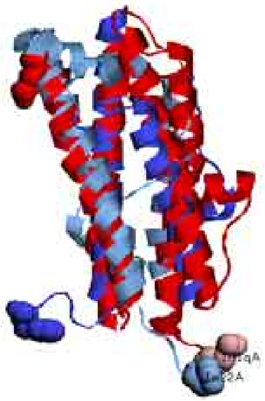
	1n1q:A (149)	DPS Protein								

12	1a22:A (182)	GROWTH HORMONE	**67**	33	0.017	0.32	3.41	112	7.7% : 8.9% (6/78 : 10/112)	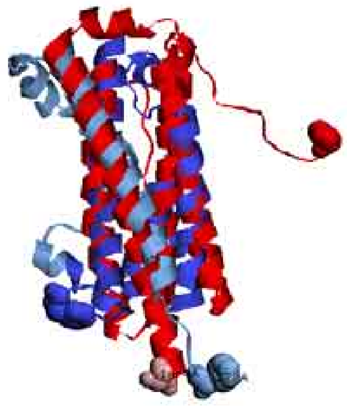
	1nf4:A (169)	bacterioferritin								
13	1rcc:_ (171)	L FERRITIN	**122**	38	0.0005	0.29	3.25	111	12.3% : 7.2% (10/81 : 8/111)	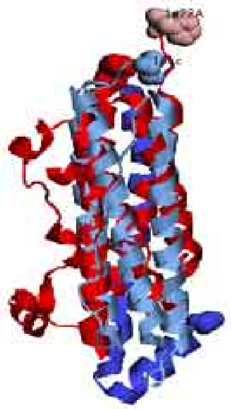
	1a22:A (182)	GROWTH HORMONE								
14	1a22:A (182)	GROWTH HORMONE	**69**	38	0.0007	0.30	3.06	97	3.8% : 5.2% (2/53 : 5/97)	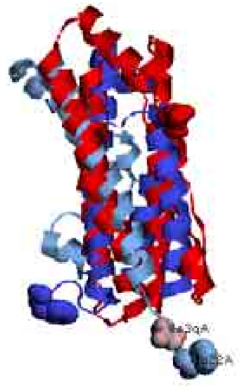
	1s3q:A (162)	ferritin								

15	1a22:A (182)	GROWTH HORMONE	**67**	36	0.003	0.37	2.97	113	5.1% : 5.3% (3/59 : 6/113)	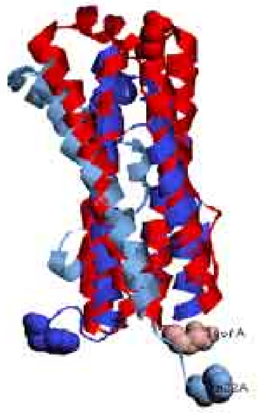
	1sof:A (155)	Bacterioferritin								
16	1a22:A (182)	GROWTH HORMONE	**70**	33	0.024	0.39	3.08	110	12.5% : 8.2% (7/56 : 9/110)	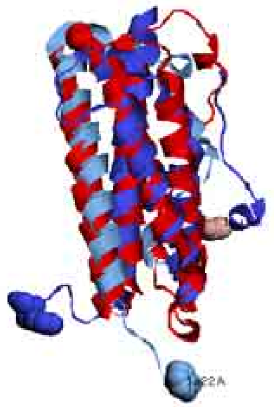
	1vjx:A (148)	putative ferritin-like diiron-carboxylate pro								
17	1a22:A (182)	GROWTH HORMONE	**55**	33	0.02	0.26	2.66	97	11.5% : 11.3% (6/52 : 11/97)	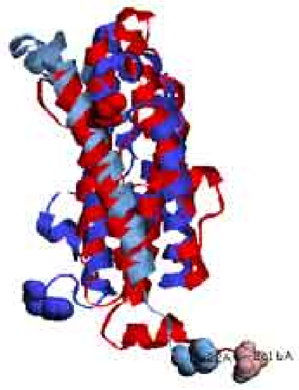
	2clb:A (169)	DPS-LIKE PROTEIN								

18	1a22:A (182)<br>2ffx:J (173)	GROWTH HORMONE<br>ferritin light chain	**63**	40	0.0001	0.30	3.49	108	7.9% : 9.3%<br>(6/76 : 10/108)	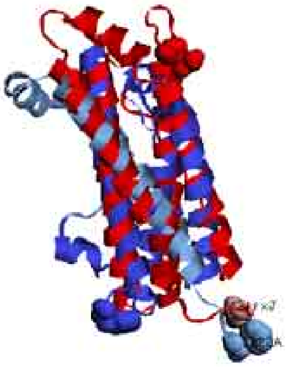
19	1a22:A (182)<br>2fjc:A (151)	GROWTH HORMONE<br>Antigen TpF1	**103**	33	0.02	0.25	2.84	85	11.1% : 8.2%<br>(8/72 : 7/85)	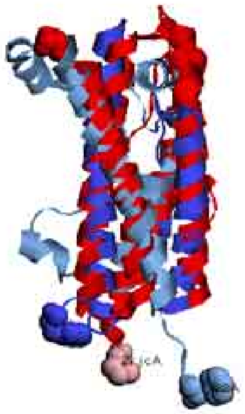
20	1a22:A (182)<br>2fzf:A (158)	GROWTH HORMONE<br>hypothetical protein	**66**	31	0.1	0.37	2.37	114	14.8% : 13.2%<br>(13/88 : 15/114)	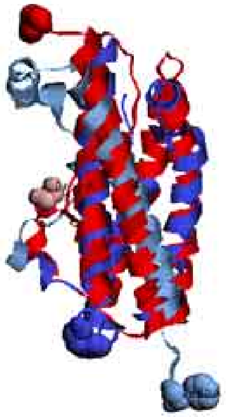

21	1a22:A (182)	GROWTH HORMONE	**106**	31	0.1	0.25	2.87	80	1.8% : 8.8% (1/57 : 7/80)	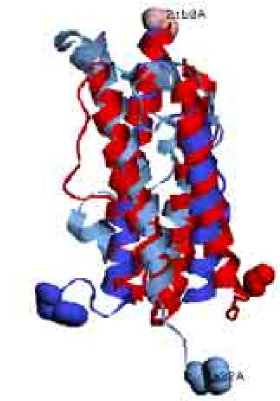
	2ib0:A (142)	CONSERVED HYPOTHETICAL ALANINE RICH PROTEIN								
22	* 1a23:_ (189)	DSBA	**100**	108	5E-25	0.90	2.46	179	100.0% : 100.0% (96/96 : 179/179)	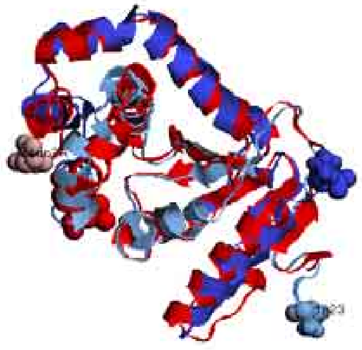
	* 1un2:A (186)	THIOL-DISULFIDE INTERCHANGE PROTEIN								
23	1a29:_ (144)	CALMODULIN	**81**	39	0.0002	0.49	2.10	52	37.5% : 26.9% (18/48 : 14/52)	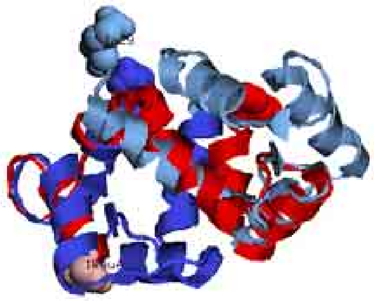
	1k9u:A (76)	Polcalcin Phl p 7								

24	1a2b:_ (178)<br>1puj:A (262)	TRANSFORMING PROTEIN RHOA<br>conserved hypothetical protein ylqF	**59**	41	9E-05	0.21	2.67	123	11.2% : 15.4% (10/89 : 19/123)	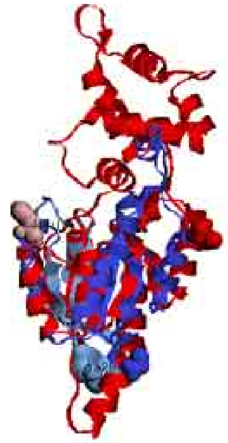
25	* 1ajk:A (213)<br>* 1a3k_ (137)	CIRCULARLY PERMUTED (1-3,1-4)-BETA-D-GLUCAN 4<br>GALECTIN-3	**187**	43	3E-05	0.31	2.93	102	13.8% : 12.7% (11/80 : 13/102)	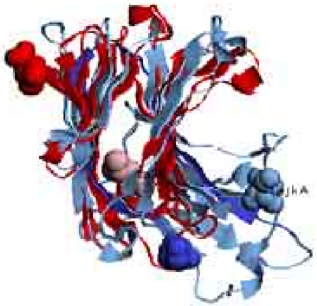
26	* 1ajo:A (212)<br>* 1a3k_ (137)	CIRCULARLY PERMUTED (1-3,1-4)-BETA-D-GLUCAN 4<br>GALECTIN-3	**143**	43	2E-05	0.46	2.87	100	14.0% : 13.0% (8/57 : 13/100)	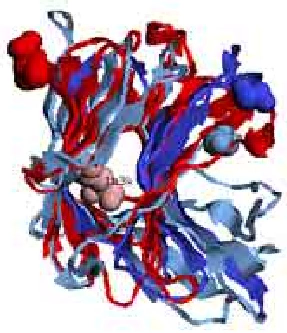

27	1a3k:_ (137)	GALECTIN-3	**42**	42	4E-05	0.33	2.94	95	10.6% : 15.8% (5/47 : 15/95)	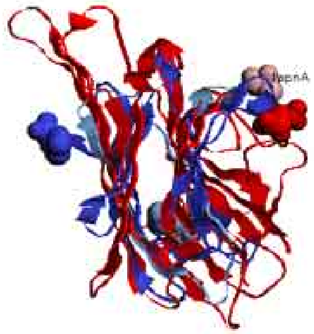
	1apn:A (226)	CONCANAVALIN A								
28	1a3k:_ (137)	GALECTIN-3	**36**	40	0.0001	0.24	2.65	91	10.2% : 17.6% (5/49 : 16/91)	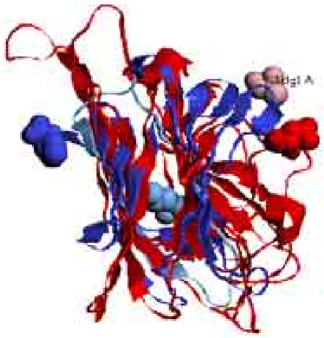
	1dgl:A (237)	LECTIN								
29	1a3k:_ (137)	GALECTIN-3	**36**	49	3E-07	0.28	2.99	93	4.3% : 17.2% (2/47 : 16/93)	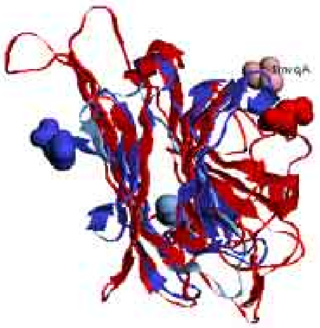
	1mvq:A (236)	lectin, isoform 1								

30	1nps:A (87)	DEVELOPMENT-SPECIFIC PROTEIN S	**47**	36	0.002	0.30	1.57	80	22.9% : 30.0% (16/70 : 24/80)	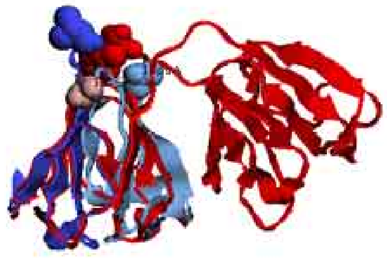
	1a45_ (174)	GAMMAF CRYSTALLIN								
31	1a50:A (261)	TRYPTOPHAN SYNTHASE (ALPHA CHAIN)	**189**	33	0.031	0.25	3.41	162	13.9% : 12.3% (20/144 : 20/162)	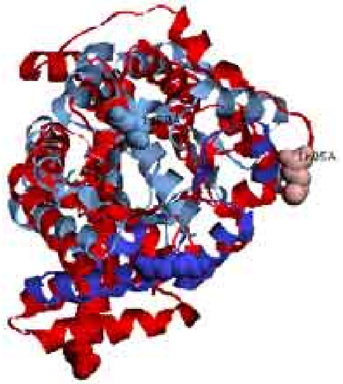
	1f05:A (322)	TRANSALDOLASE								
32	1a50:A (261)	TRYPTOPHAN SYNTHASE (ALPHA CHAIN)	**133**	38	0.001	0.36	3.12	158	13.6% : 13.3% (20/147 : 21/158)	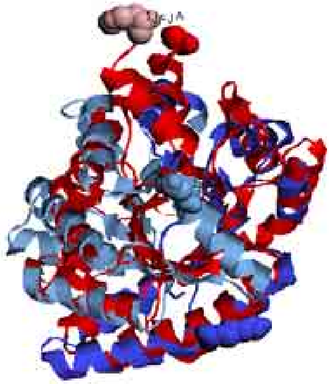
	1jcj:A (252)	DEOXYRIBOSE-PHOSPHATE ALDOLASE								

33	1nps:A (87)	DEVELOPMENT-SPECIFIC PROTEIN S	**47**	32	0.038	0.29	1.65	79	26.4% : 24.1% (19/72 : 19/79)	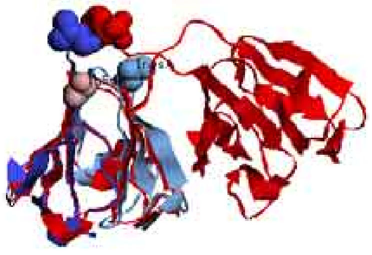
	1a5d:A (174)	GAMMAE CRYSTALLIN								
34	* 1ajk:A (213)	CIRCULARLY PERMUTED (1-3,1-4)-BETA-D-GLUCAN 4	**188**	39	0.0004	0.32	2.70	100	10.1% : 11.0% (8/79 : 11/100)	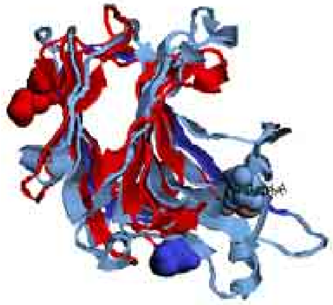
	* 1a78:A (134)	GALECTIN-1								
35	* 1ajo:A (212)	CIRCULARLY PERMUTED (1-3,1-4)-BETA-D-GLUCAN 4	**144**	35	0.006	0.47	2.64	98	5.0% : 11.2% (3/60 : 11/98)	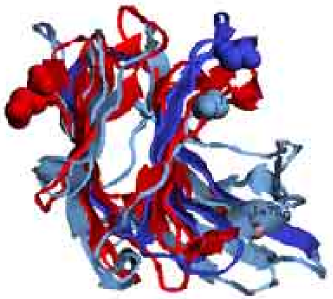
	* 1a78:A (134)	GALECTIN-1								

36	1a78:A (134)	GALECTIN-1	**44**	37	0.0007	0.31	2.92	92	9.1% : 13.0% (5/55 : 12/92)	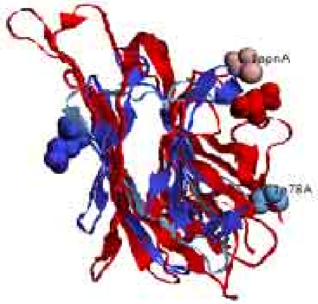
	1apn:A (226)	CONCANAVALIN A								
37	1a78:A (134)	GALECTIN-1	**37**	35	0.004	0.27	2.16	89	12.9% : 13.5% (9/70 : 12/89)	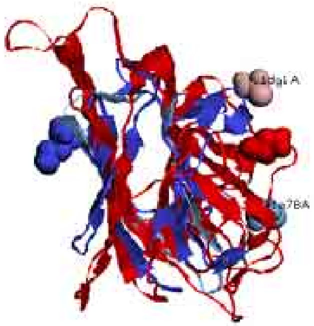
	1dgl:A (237)	LECTIN								
38	1a78:A (134)	GALECTIN-1	**37**	42	4E-05	0.28	2.30	90	8.0% : 13.3% (4/50 : 12/90)	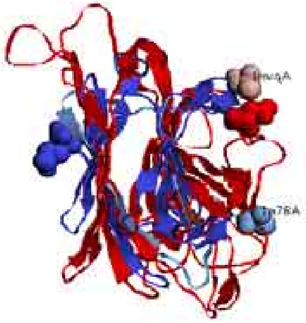
	1mvq:A (236)	lectin, isoform 1								

39	1a7d:_ (118)	MYOHEMERYTHRIN	**61**	38	0.0005	0.35	3.08	85	6.0% : 5.9% (5/83 : 5/85)	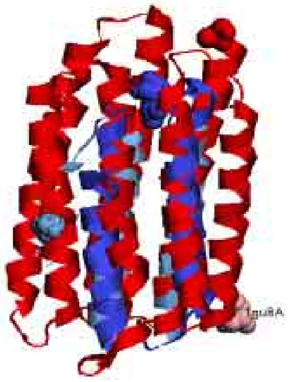
	1gu8:A (218)	SENSORY RHODOPSIN II								
40	1a7d:_ (118)	MYOHEMERYTHRIN	**41**	42	2E-05	0.21	2.90	75	7.1% : 8.0% (3/42 : 6/75)	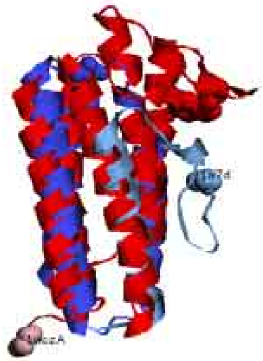
	1woz:A (159)	177aa long conserved hypothetical protein (ST								
41	1a7d:_ (118)	MYOHEMERYTHRIN	**41**	42	2E-05	0.23	2.70	74	2.5% : 5.4% (1/40 : 4/74)	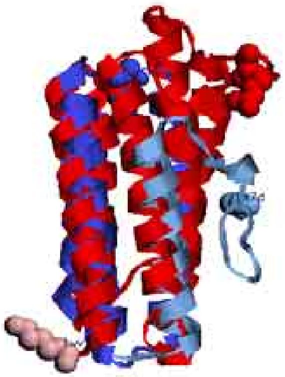
	1wvt:A (148)	hypothetical protein ST2180								

42	1a7d:_ (118)	MYOHEMERYTHRIN	**41**	32	0.026	0.24	3.09	75	6.7% : 6.7% (3/45 : 5/75)	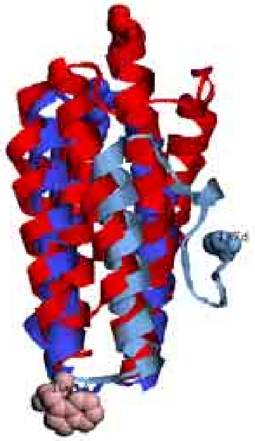
	1wy1:A (142)	hypothetical protein PH0671								
43	1a7d:_ (118)	MYOHEMERYTHRIN	**41**	32	0.031	0.21	3.29	74	3.2% : 4.1% (2/62 : 3/74)	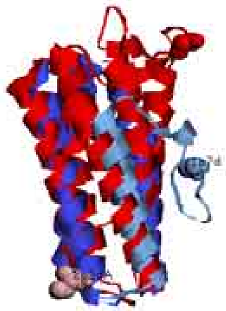
	2g2d:A (160)	ATP:cobalamin adenosyltransferase								
44	1a7v:A (125)	CYTOCHROME C'	**63**	32	0.025	0.27	3.10	71	12.5% : 9.9% (7/56 : 7/71)	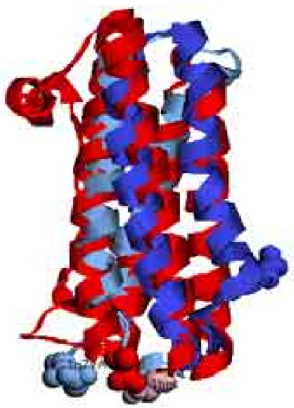
	1bgc_ (159)	GRANULOCYTE COLONY-STIMULATING FACTOR								

45	1a7v:A (125)	CYTOCHROME C'	**60**	30	0.089	0.24	2.42	71	12.7% : 11.3% (7/55 : 8/71)	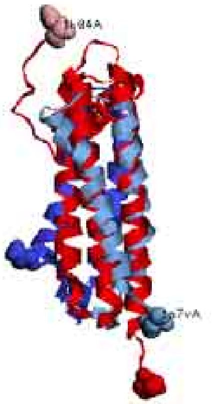
	1k04:A (142)	FOCAL ADHESION KINASE 1								
46	1rty:A (159)	yvqk protein	**91**	35	0.003	0.27	3.23	89	8.3% : 5.6% (4/48 : 5/89)	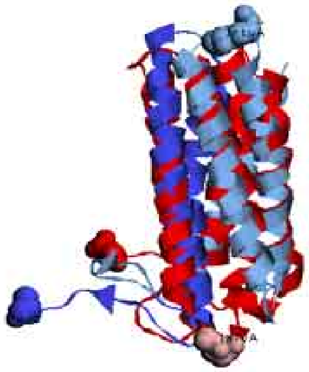
	1a7v:A (125)	CYTOCHROME C'								
47	1vjx:A (148)	putative ferritin-like diiron-carboxylate pro	**88**	37	0.001	0.23	3.04	70	4.0% : 7.1% (2/50 : 5/70)	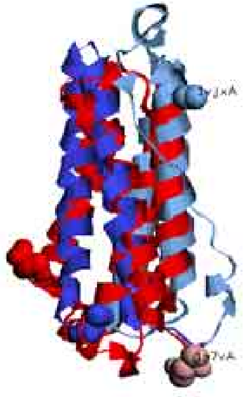
	1a7v:A (125)	CYTOCHROME C'								

48	2ah6:A (152)	BH1595, unknown conserved protein	**87**	33	0.013	0.29	3.08	88	8.3% : 8.0% (5/60 : 7/88)	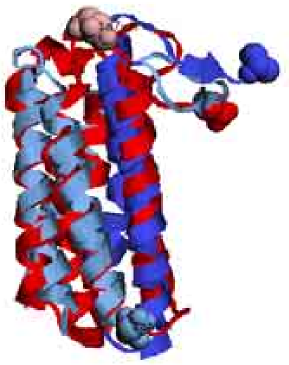
	1a7v:A (125)	CYTOCHROME C'								
49	2g2d:A (160)	ATP:cobalamin adenosyltransferase	**95**	40	0.0002	0.26	2.96	82	10.6% : 11.0% (5/47 : 9/82)	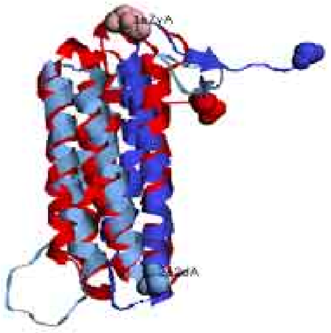
	1a7v:A (125)	CYTOCHROME C'								
50	1ab0:_ (131)	ADIPOCYTE LIPID BINDING PROTEIN	**88**	37	0.0008	0.29	2.43	91	3.5% : 2.2% (3/85 : 2/91)	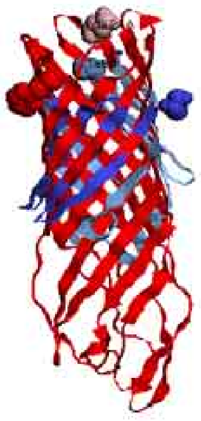
	1k24:A (249)	outer membrane protein								

51	1ad1:A<br>(264)<br>1vcv:A<br>(226)	DIHYDROPTEROATE SYNTHETASE<br>Probable deoxyribose-phosphate aldolase	**155**	36	0.004	0.25	2.59	135	17.3% : 19.3%<br>(23/133 : 26/135)	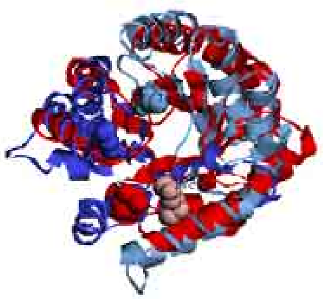
52	1jmw:A<br>(146)<br>1aep_<br>(153)	methyl-accepting chemotaxis protein II<br>APOLIPOPHORIN III	**121**	38	0.0003	0.24	3.39	98	5.5% : 13.3%<br>(4/73 : 13/98)	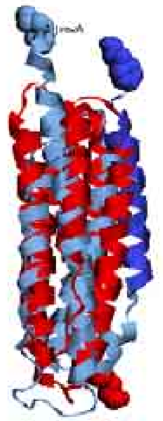
53	1aep:_<br>(153)<br>1wdz:A<br>(231)	APOLIPOPHORIN III<br>insulin receptor substrate p53	**88**	60	1E-10	0.27	2.86	94	2.5% : 10.6%<br>(2/81 : 10/94)	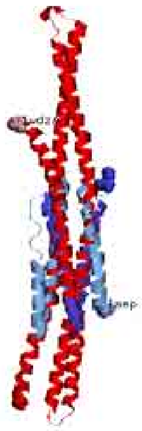

54	1bgc:_ (159)	GRANULOCYTE COLONY-STIMULATING FACTOR	**47**	53	2E-08	0.37	2.79	115	7.1% : 7.8% (4/56 : 9/115)	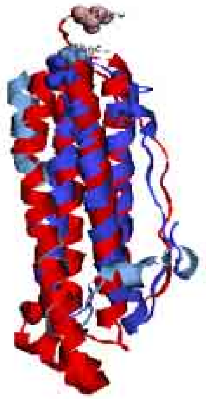
	1aew_ (170)	FERRITIN								
55	1bgd:_ (158)	GRANULOCYTE COLONY-STIMULATING FACTOR	**46**	47	1E-06	0.40	3.34	117	3.3% : 7.7% (2/61 : 9/117)	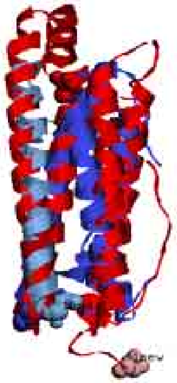
	1aew_ (170)	FERRITIN								
56	1cd9:A (171)	PROTEIN (GRANULOCYTE COLONY-STIMULATING FACTO	**51**	48	6E-07	0.37	3.06	124	4.1% : 7.3% (3/74 : 9/124)	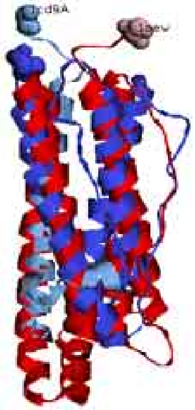
	1aew_ (170)	FERRITIN								

57	1aew:_ (170)	FERRITIN	91	40	0.0002	0.36	3.11	90	11.6% : 5.6% (10/86 : 5/90)	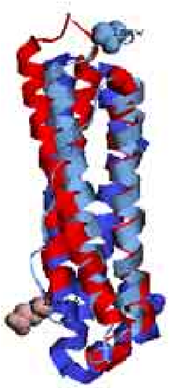
	1lvf:A (106)	syntaxin 6								
58	1aew:_ (170)	FERRITIN	59	49	2E-07	0.21	3.13	82	8.7% : 3.7% (6/69 : 3/82)	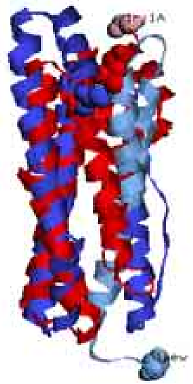
	1rj1:A (148)	invertase inhibitor								
59	1z7c:A (165)	Chorionic somatomammotropin hormone	44	36	0.003	0.29	3.36	103	8.5% : 5.8% (5/59 : 6/103)	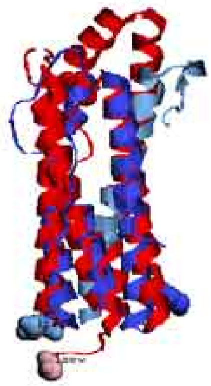
	1aew_ (170)	FERRITIN								

60	2g2d:A (160)	ATP:cobalamin adenosyltransferase	**85**	37	0.001	0.37	2.38	98	9.6% : 14.3% (9/94 : 14/98)	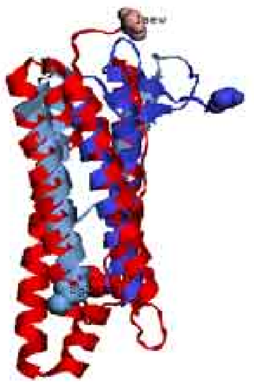
	1aew_ (170)	FERRITIN								
61	* 1g2b:A (63)	SPECTRIN ALPHA CHAIN	**16**	96	2E-21	0.62	0.86	53	100.0% : 100.0% (40/40 : 53/53)	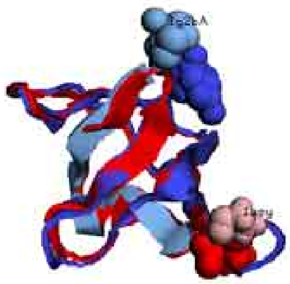
	* 1aey_ (58)	ALPHA-SPECTRIN								
62	* 1aey:_ (58)	ALPHA-SPECTRIN	**18**	95	3E-21	0.63	1.21	54	100.0% : 100.0% (40/40 : 54/54)	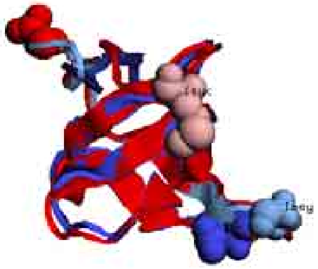
	* 1tuc_ (65)	ALPHA-SPECTRIN								

63	1l3p:A (102)	POLLEN ALLERGEN Phl p 5b	**77**	34	0.006	0.35	2.05	44	5.0% : 2.3% (2/40 : 1/44)	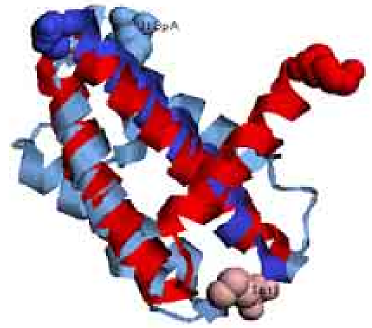
	1ail_ (70)	NONSTRUCTURAL PROTEIN NS1								
64	1o3u:A (121)	conserved hypothetical protein TM0613	**94**	36	0.002	0.34	1.41	42	11.8% : 4.8% (4/34 : 2/42)	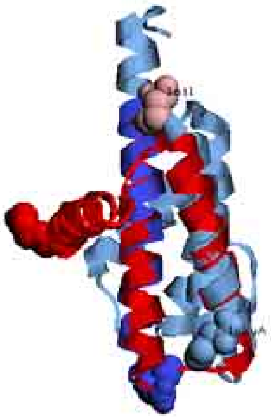
	1ail_ (70)	NONSTRUCTURAL PROTEIN NS1								
65	1qbz:A (103)	PROTEIN (SIV GP41 ECTODOMAIN)	**34**	43	2E-05	0.47	3.43	68	8.2% : 11.8% (4/49 : 8/68)	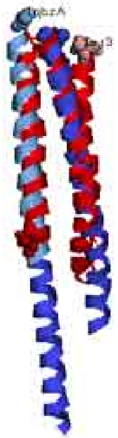
	1aj3_ (98)	ALPHA SPECTRIN								

66	1wdf:A (92)	E2 glycoprotein	**65**	31	0.062	0.33	2.20	54	7.0% : 16.7% (3/43 : 9/54)	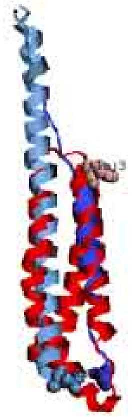
	1aj3_ (98)	ALPHA SPECTRIN								
67	1aj3:_ (98)	ALPHA SPECTRIN	**55**	48	5E-07	0.34	3.31	72	9.1% : 4.2% (6/66 : 3/72)	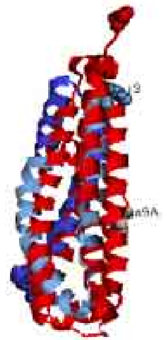
	1ya9:A (169)	Apolipoprotein E								
68	1aj3:_ (98)	ALPHA SPECTRIN	**42**	50	1E-07	0.43	3.23	77	3.2% : 7.8% (2/62 : 6/77)	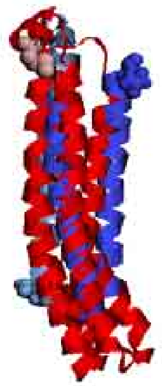
	2bl2:A (156)	V-TYPE SODIUM ATP SYNTHASE SUBUNIT K								

69	1aj3:_ (98)	ALPHA SPECTRIN	**73**	41	7E-05	0.33	3.49	59	9.1% : 16.9% (4/44 : 10/59)	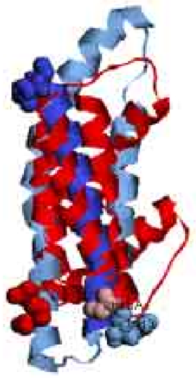
	2hsb:A (126)	Hypothetical UPF0332 protein AF0298								
70	* 1ajk:A (213)	CIRCULARLY PERMUTED (1-3,1-4)-BETA-D-GLUCAN 4	**46**	203	1E-53	0.56	0.45	195	100.0% : 100.0% (155/155 : 195/195)	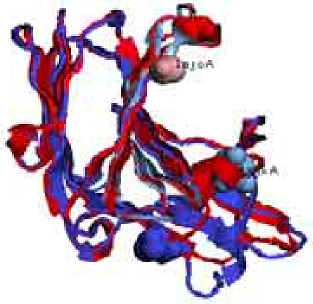
	* 1ajo:A (212)	CIRCULARLY PERMUTED (1-3,1-4)-BETA-D-GLUCAN 4								
71	* 1ajk:A (213)	CIRCULARLY PERMUTED (1-3,1-4)-BETA-D-GLUCAN 4	**187**	53	2E-08	0.37	2.86	111	12.9% : 11.7% (11/85 : 13/111)	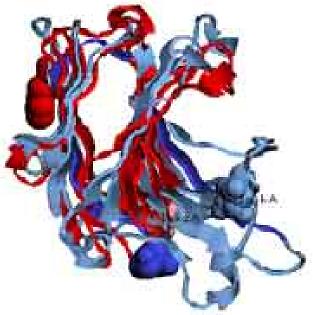
	* 1bkz:A (135)	GALECTIN-7								

72	* 1byh:_ (214)	HYBRID	**84**	176	2E-45	0.79	0.71	193	100.0% : 100.0% (115/115 : 193/193)	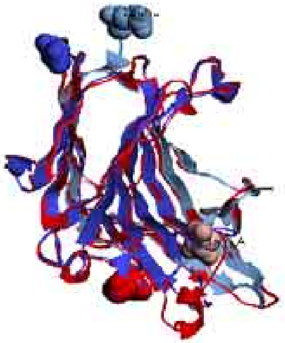
	* 1ajk:A (213)	CIRCULARLY PERMUTED (1-3,1-4)-BETA-D-GLUCAN 4								
73	* 1ajk:A (213)	CIRCULARLY PERMUTED (1-3,1-4)-BETA-D-GLUCAN 4	**191**	35	0.008	0.31	3.36	96	9.5% : 9.4% (7/74 : 9/96)	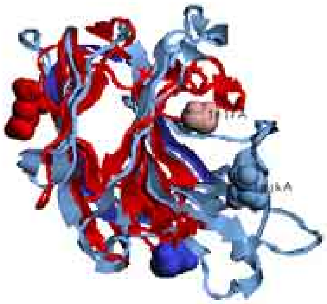
	* 1c1f:A (135)	PROTEIN (CONGERIN I)								
74	* 1ajk:A (213)	CIRCULARLY PERMUTED (1-3,1-4)-BETA-D-GLUCAN 4	**174**	35	0.008	0.23	2.69	114	12.9% : 9.6% (13/101 : 11/114)	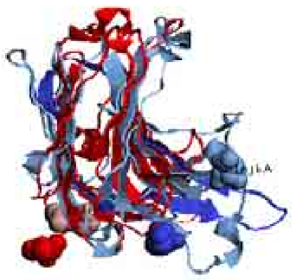
	* 1c4r:A (183)	NEUREXIN-I BETA								

75	* 1ajk:A (213)	CIRCULARLY PERMUTED (1-3,1-4)-BETA-D-GLUCAN 4	**189**	196	2E-51	0.35	0.67	193	98.8% : 99.0% (167/169 : 191/193)	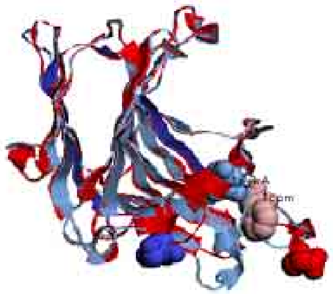
	* 1cpm_ (214)	CIRCULARLY PERMUTED								
76	* 1ajk:A (213)	CIRCULARLY PERMUTED (1-3,1-4)-BETA-D-GLUCAN 4	**147**	36	0.003	0.36	2.80	129	10.1% : 8.5% (9/89 : 11/129)	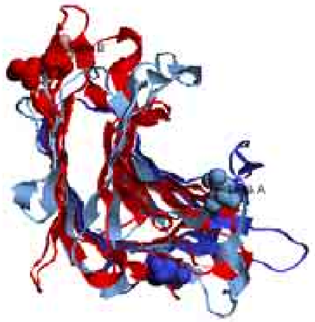
	* 1dhk:B (197)	BEAN LECTIN-LIKE INHIBITOR								
77	* 1dyp:A (271)	KAPPA-CARRAGEENASE	**132**	51	2E-07	0.44	2.34	157	22.3% : 23.6% (23/103 : 37/157)	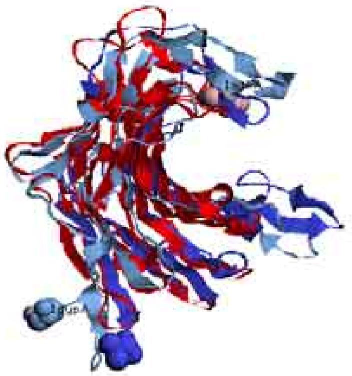
	* 1ajk:A (213)	CIRCULARLY PERMUTED (1-3,1-4)-BETA-D-GLUCAN 4								

78	* 1fat:A (233)	PHYTOHEMAGGLUTININ-L	**116**	35	0.005	0.39	3.09	134	10.5% : 8.2% (4/38 : 11/134)	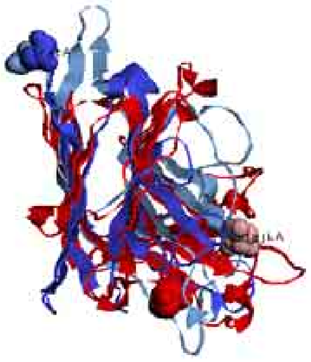
	* 1ajk:A (213)	CIRCULARLY PERMUTED (1-3,1-4)-BETA-D-GLUCAN 4								
79	* 1ajk:A (213)	CIRCULARLY PERMUTED (1-3,1-4)-BETA-D-GLUCAN 4	**145**	34	0.012	0.30	2.80	126	3.6% : 11.9% (2/56 : 15/126)	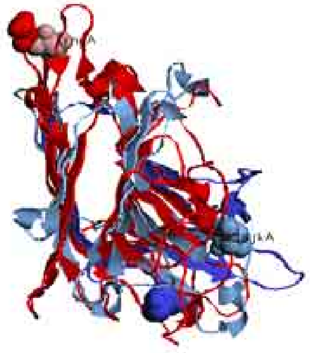
	* 1fny:A (235)	BARK AGGLUTININ I,POLYPEPTIDE A								
80	* 1ajk:A (213)	CIRCULARLY PERMUTED (1-3,1-4)-BETA-D-GLUCAN 4	**182**	52	6E-08	0.34	3.18	113	9.0% : 8.8% (8/89 : 10/113)	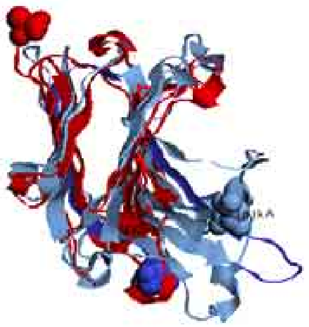
	* 1g86:A (141)	CHARCOT-LEYDEN CRYSTAL PROTEIN								

81	* 1g9f:A (242)	LECTIN	**113**	35	0.01	0.38	3.00	137	11.5% : 12.4% (10/87 : 17/137)	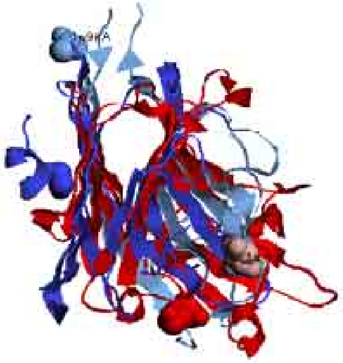
	* 1ajk:A (213)	CIRCULARLY PERMUTED (1-3,1-4)-BETA-D-GLUCAN 4								
82	* 1gbg:_ (214)	(1,3-1,4)-BETA-D-GLUCAN 4 GLUCANOHYDROLASE	**84**	199	3E-52	0.80	0.86	193	80.7% : 76.7% (92/114 : 148/193)	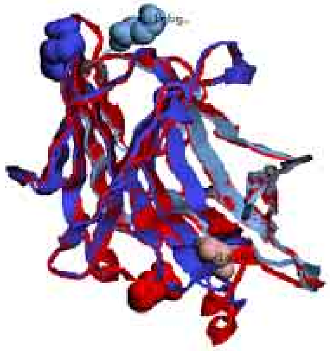
	* 1ajk:A (213)	CIRCULARLY PERMUTED (1-3,1-4)-BETA-D-GLUCAN 4								
83	* 1ajk:A (213)	CIRCULARLY PERMUTED (1-3,1-4)-BETA-D-GLUCAN 4	**115**	34	0.01	0.24	3.31	85	3.5% : 11.8% (2/57 : 10/85)	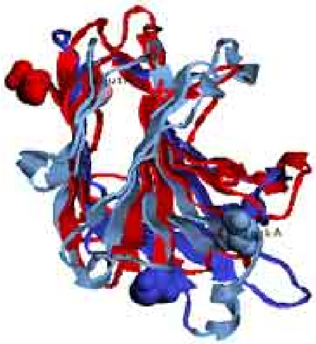
	* 1gui:A (155)	LAMINARINASE 16A								

84	* 1ajk:A (213)<br>* 1gv9:A (224)	CIRCULARLY PERMUTED (1-3,1-4)-BETA-D-GLUCAN 4<br>P58/ERGIC-53	**139**	35	0.006	0.33	2.73	133	4.1% : 9.8% (2/49 : 13/133)	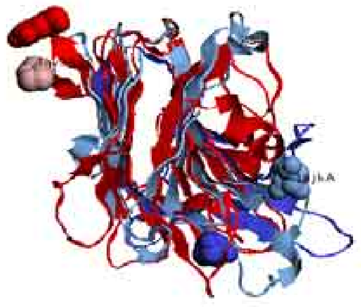
85	* 1ajk:A (213)<br>* 1gzw:A (134)	CIRCULARLY PERMUTED (1-3,1-4)-BETA-D-GLUCAN 4<br>GALECTIN-1	**189**	41	0.0001	0.32	2.63	99	9.0% : 11.1% (7/78 : 11/99)	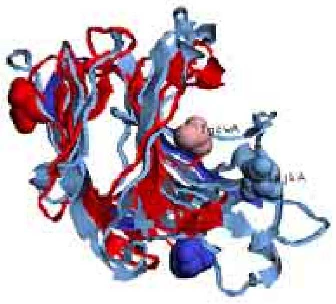
86	* 1ajk:A (213)<br>* 1is3:A (134)	CIRCULARLY PERMUTED (1-3,1-4)-BETA-D-GLUCAN 4<br>CONGERIN II	**190**	37	0.001	0.34	2.55	103	9.9% : 8.7% (8/81 : 9/103)	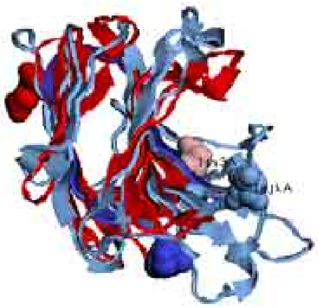

87	* 1ajk:A (213)	CIRCULARLY PERMUTED (1-3,1-4)-BETA-D-GLUCAN 4	**139**	34	0.017	0.33	3.28	138	4.0% : 13.0% (2/50 : 18/138)	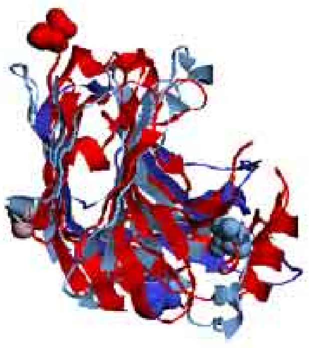
	* 1j1t:A (228)	Alginate Lyase								
88	* 1ajk:A (213)	CIRCULARLY PERMUTED (1-3,1-4)-BETA-D-GLUCAN 4	**183**	105	3E-24	0.28	1.95	177	29.4% : 29.9% (45/153 : 53/177)	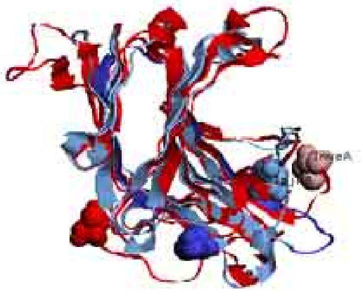
	* 1mve:A (243)	Truncated 1,3-1,4-beta-D-glucanase								
89	* 1o4y:A (270)	beta-agarase A	**130**	53	3E-08	0.43	2.42	160	22.2% : 18.8% (24/108 : 30/160)	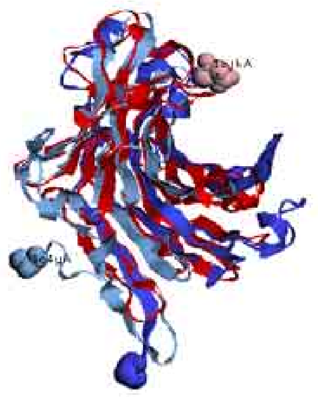
	* 1ajk:A (213)	CIRCULARLY PERMUTED (1-3,1-4)-BETA-D-GLUCAN 4								

90	* 1ajk:A (213)	CIRCULARLY PERMUTED (1-3,1-4)-BETA-D-GLUCAN 4	**106**	52	4E-08	0.35	1.81	155	26.2% : 23.9% (27/103 : 37/155)	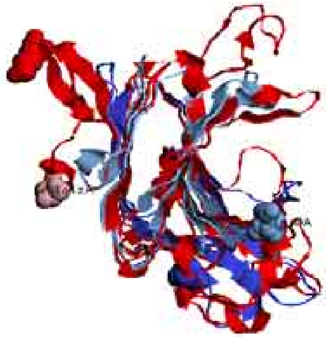
	* 1o4z:A (295)	beta-agarase B								
91	* 1ajk:A (213)	CIRCULARLY PERMUTED (1-3,1-4)-BETA-D-GLUCAN 4	**126**	40	0.0002	0.40	2.79	134	11.9% : 13.4% (10/84 : 18/134)	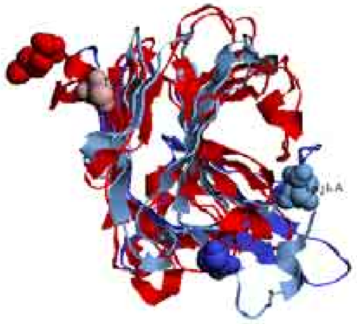
	* 1oq1:A (223)	Protein yesU								
92	* 1ajk:A (213)	CIRCULARLY PERMUTED (1-3,1-4)-BETA-D-GLUCAN 4	**191**	35	0.005	0.33	2.78	99	12.8% : 13.1% (10/78 : 13/99)	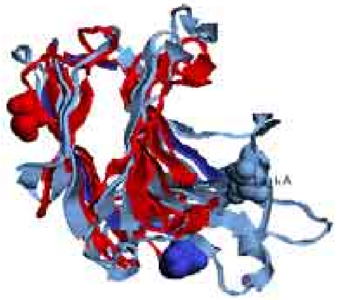
	* 1qmj:A (133)	BETA-GALACTOSIDE-BINDING LECTIN								

93	* 1ajk:A (213)	CIRCULARLY PERMUTED (1-3,1-4)-BETA-D-GLUCAN 4	**189**	39	0.0004	0.31	2.61	99	10.3% : 11.1% (8/78 : 11/99)	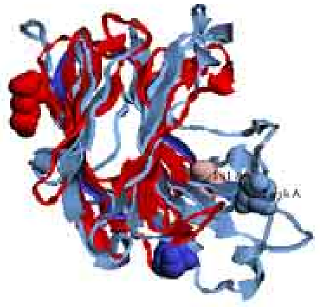
	* 1sla:A (134)	BOVINE GALECTIN-1								
94	* 1ajk:A (213)	CIRCULARLY PERMUTED (1-3,1-4)-BETA-D-GLUCAN 4	**109**	56	4E-09	0.26	2.87	112	14.1% : 11.6% (12/85 : 13/112)	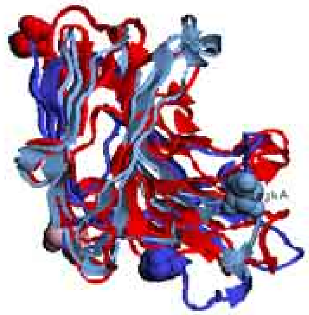
	* 1uai:A (223)	polyguluronate lyase								
95	* 1ajk:A (213)	CIRCULARLY PERMUTED (1-3,1-4)-BETA-D-GLUCAN 4	**151**	79	5E-16	0.28	1.98	151	35.5% : 31.1% (39/110 : 47/151)	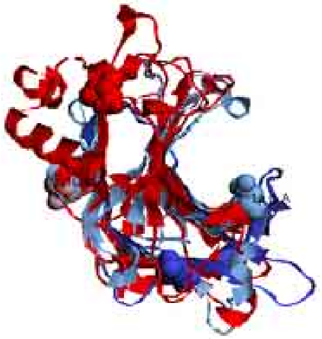
	* 1umz:A (267)	XYLOGLUCAN ENDOTRANSGLYCOSYLASE								

96	* 1wbf:A (237)	PROTEIN (AGGLUTININ)	**114**	34	0.02	0.36	2.95	131	1.9% : 9.2% (1/53 : 12/131)	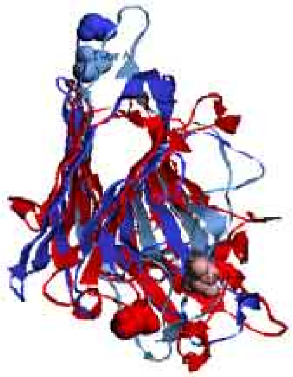
	* 1ajk:A (213)	CIRCULARLY PERMUTED (1-3,1-4)-BETA-D-GLUCAN 4								
97	* 1ajk:A (213)	CIRCULARLY PERMUTED (1-3,1-4)-BETA-D-GLUCAN 4	**146**	35	0.008	0.32	2.27	129	12.9% : 16.3% (12/93 : 21/129)	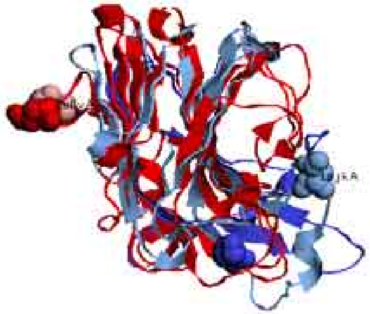
	* 2a6v:A (218)	Emp46p								
98	* 1ajk:A (213)	CIRCULARLY PERMUTED (1-3,1-4)-BETA-D-GLUCAN 4	**139**	41	8E-05	0.34	2.66	138	13.7% : 12.3% (13/95 : 17/138)	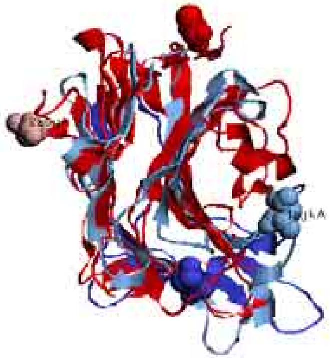
	* 2a6y:A (232)	Emp47p (form1)								

99	* 2d6k:A (148)	lectin, galactose binding, soluble 9	**35**	50	1E-07	0.25	3.10	104	12.9% : 10.6% (11/85 : 11/104)	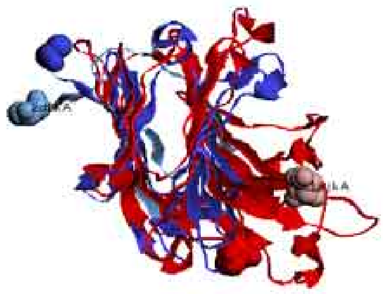
	* 1ajk:A (213)	CIRCULARLY PERMUTED (1-3,1-4)-BETA-D-GLUCAN 4								
100	* 1ajo:A (212)	CIRCULARLY PERMUTED (1-3,1-4)-BETA-D-GLUCAN 4	**145**	179	2E-46	0.49	0.72	206	93.5% : 95.6% (129/138 : 197/206)	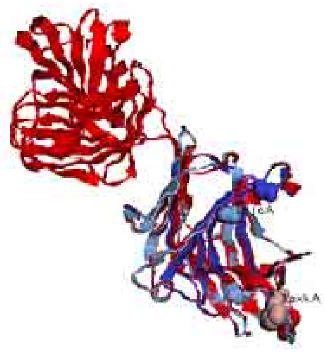
	* 1axk:A (393)	GLUXYN-1								
101	* 1ajo:A (212)	CIRCULARLY PERMUTED (1-3,1-4)-BETA-D-GLUCAN 4	**142**	40	0.0002	0.27	3.17	110	10.0% : 15.5% (6/60 : 17/110)	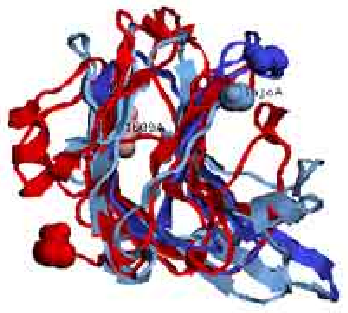
	* 1b09:A (206)	PROTEIN (C-REACTIVE PROTEIN)								

102	* 1ajo:A (212)	CIRCULARLY PERMUTED (1-3,1-4)-BETA-D-GLUCAN 4	**143**	43	3E-05	0.53	2.66	105	15.5% : 12.4% (9/58 : 13/105)	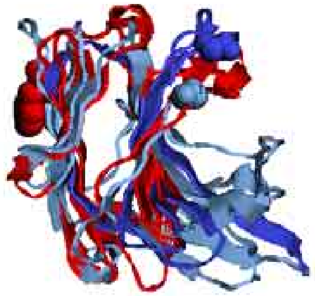
	* 1bkz:A (135)	GALECTIN-7								
103	* 1ajo:A (212)	CIRCULARLY PERMUTED (1-3,1-4)-BETA-D-GLUCAN 4	**87**	190	1E-49	0.93	0.58	208	100.0% : 100.0% (122/122 : 208/208)	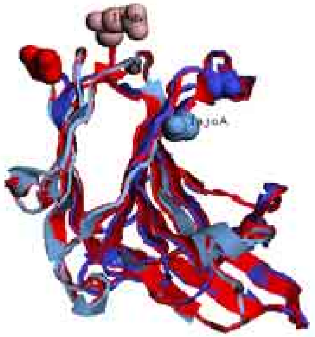
	* 1byh_ (214)	HYBRID								
104	* 1ajo:A (212)	CIRCULARLY PERMUTED (1-3,1-4)-BETA-D-GLUCAN 4	**118**	35	0.008	0.42	3.32	93	22.8% : 9.7% (13/57 : 9/93)	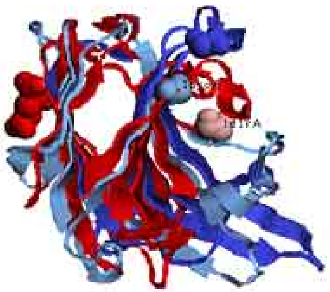
	* 1c1f:A (135)	PROTEIN (CONGERIN I)								

105	* 1c4r:A (183)	NEUREXIN-I BETA	**77**	34	0.014	0.43	2.88	125	11.1% : 10.4% (6/54 : 13/125)	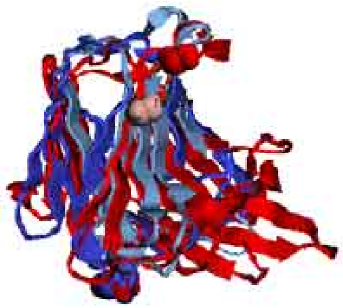
	* 1ajo:A (212)	CIRCULARLY PERMUTED (1-3,1-4)-BETA-D-GLUCAN 4								
106	* 1cpm:_ (214)	CIRCULARLY PERMUTED	**71**	214	6E-57	0.85	0.50	208	100.0% : 100.0% (141/141 : 208/208)	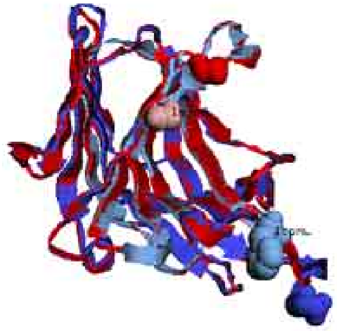
	* 1ajo:A (212)	CIRCULARLY PERMUTED (1-3,1-4)-BETA-D-GLUCAN 4								
107	* 1d2s:A (171)	SEX HORMONE-BINDING GLOBULIN	**73**	45	3E-06	0.36	2.68	108	14.3% : 10.2% (6/42 : 11/108)	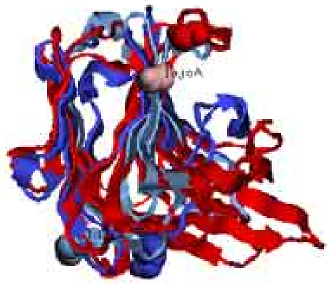
	* 1ajo:A (212)	CIRCULARLY PERMUTED (1-3,1-4)-BETA-D-GLUCAN 4								

108	* 1dhk:B (197)	BEAN LECTIN-LIKE INHIBITOR	**110**	33	0.027	0.50	3.01	143	11.1% : 9.8% (6/54 : 14/143)	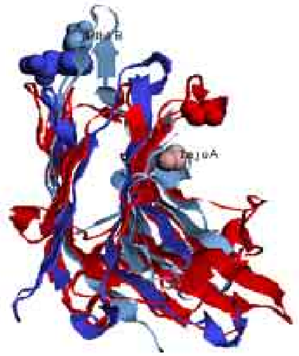
	* 1ajo:A (212)	CIRCULARLY PERMUTED (1-3,1-4)-BETA-D-GLUCAN 4								
109	* 1dyp:A (271)	KAPPA-CARRAGEENASE	**177**	60	2E-10	0.45	1.50	159	25.3% : 25.2% (23/91 : 40/159)	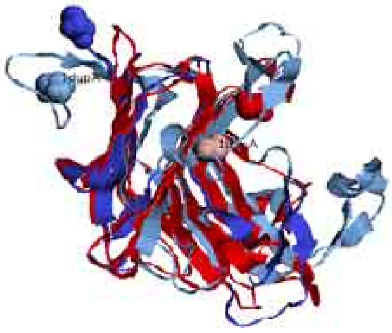
	* 1ajo:A (212)	CIRCULARLY PERMUTED (1-3,1-4)-BETA-D-GLUCAN 4								
110	* 1g86:A (141)	CHARCOT-LEYDEN CRYSTAL PROTEIN	**72**	41	5E-05	0.51	3.26	108	10.3% : 10.2% (6/58 : 11/108)	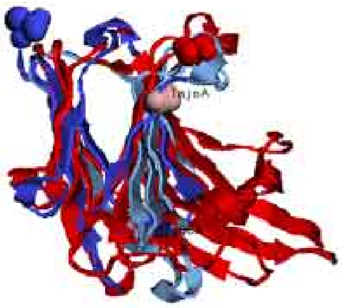
	* 1ajo:A (212)	CIRCULARLY PERMUTED (1-3,1-4)-BETA-D-GLUCAN 4								

111	* 1gbg:_ (214)	(1,3-1,4)-BETA-D-GLUCAN 4 GLUCANOHYDROLASE	**129**	221	4E-59	0.94	0.81	209	77.9% : 76.1% (95/122 : 159/209)	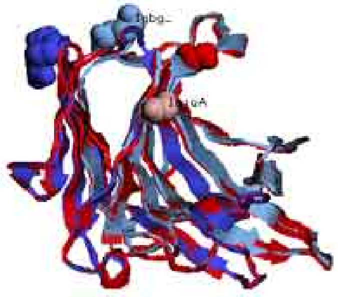
	* 1ajo:A (212)	CIRCULARLY PERMUTED (1-3,1-4)-BETA-D-GLUCAN 4								
112	* 1ajo:A (212)	CIRCULARLY PERMUTED (1-3,1-4)-BETA-D-GLUCAN 4	**109**	34	0.01	0.24	2.66	83	14.1% : 10.8% (9/64 : 9/83)	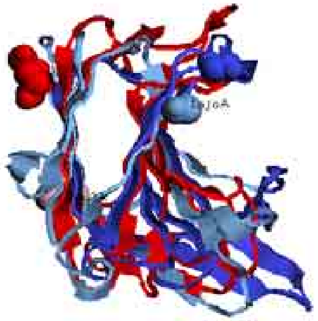
	* 1gu3:A (142)	ENDOGLUCANASE C								
113	* 1gv9:A (224)	P58/ERGIC-53	**140**	32	0.054	0.41	2.74	135	16.1% : 9.6% (9/56 : 13/135)	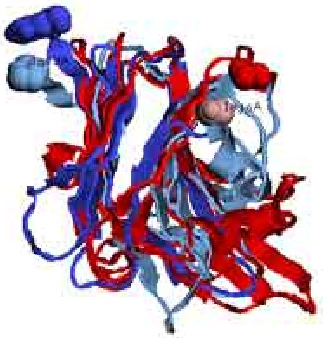
	* 1ajo:A (212)	CIRCULARLY PERMUTED (1-3,1-4)-BETA-D-GLUCAN 4								

114	* 1ajo:A (212)	CIRCULARLY PERMUTED (1-3,1-4)-BETA-D-GLUCAN 4	**142**	37	0.002	0.32	3.37	120	5.5% : 10.0% (3/55 : 12/120)	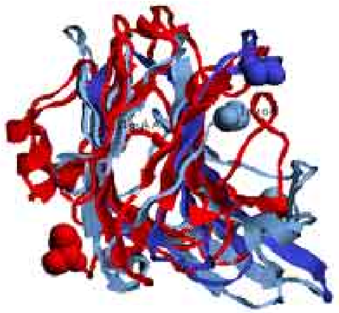
	* 1gyk:A (204)	SERUM AMYLOID P-COMPONENT								
115	* 1ajo:A (212)	CIRCULARLY PERMUTED (1-3,1-4)-BETA-D-GLUCAN 4	**145**	38	0.001	0.46	2.69	97	17.6% : 10.3% (9/51 : 10/97)	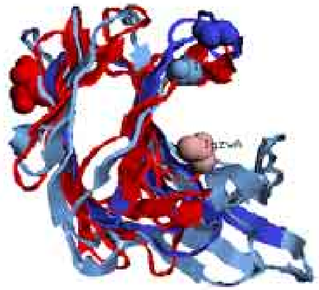
	* 1gzw:A (134)	GALECTIN-1								
116	* 1ajo:A (212)	CIRCULARLY PERMUTED (1-3,1-4)-BETA-D-GLUCAN 4	**147**	34	0.017	0.50	2.52	102	8.6% : 8.8% (5/58 : 9/102)	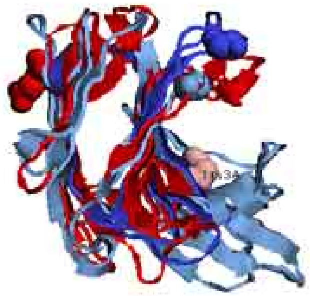
	* 1is3:A (134)	CONGERIN II								

117	* 1ajo:A (212)	CIRCULARLY PERMUTED (1-3,1-4)-BETA-D-GLUCAN 4	**139**	112	4E-26	0.60	1.40	188	25.2% : 29.8% (32/127 : 56/188)	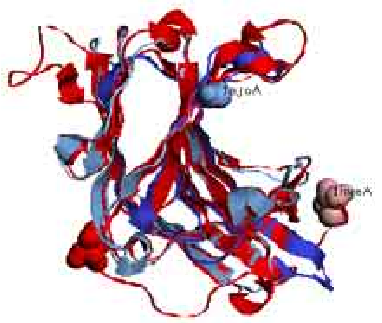
	* 1mve:A (243)	Truncated 1,3-1,4-beta-D-glucanase								
118	* 1o4y:A (270)	beta-agarase A	**168**	50	2E-07	0.53	2.03	177	21.6% : 20.9% (22/102 : 37/177)	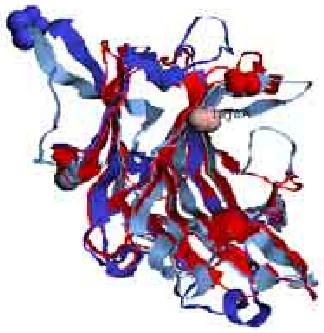
	* 1ajo:A (212)	CIRCULARLY PERMUTED (1-3,1-4)-BETA-D-GLUCAN 4								
119	* 1ajo:A (212)	CIRCULARLY PERMUTED (1-3,1-4)-BETA-D-GLUCAN 4	**62**	48	9E-07	0.38	1.98	163	22.3% : 23.3% (23/103 : 38/163)	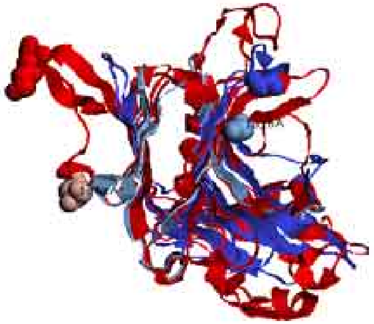
	* 1o4z:A (295)	beta-agarase B								

120	* 1oq1:A (223)	Protein yesU	143	42	4E-05	0.43	2.96	137	10.0% : 13.1% (7/70 : 18/137)	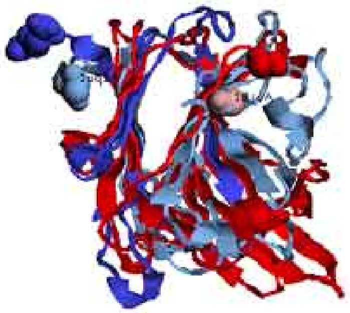
	* 1ajo:A (212)	CIRCULARLY PERMUTED (1-3,1-4)-BETA-D-GLUCAN 4								
121	* 1ajo:A (212)	CIRCULARLY PERMUTED (1-3,1-4)-BETA-D-GLUCAN 4	129	37	0.002	0.38	2.81	121	7.7% : 12.4% (4/52 : 15/121)	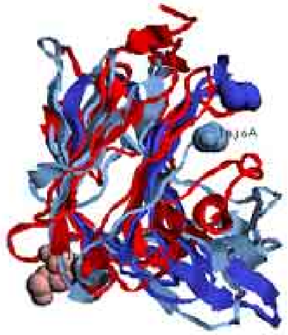
	* 1pz7:A (189)	Agrin								
122	* 1qmj:A (133)	BETA-GALACTOSIDE-BINDING LECTIN	68	30	0.09	0.45	2.77	95	10.5% : 12.6% (6/57 : 12/95)	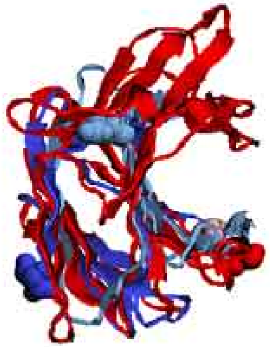
	* 1ajo:A (212)	CIRCULARLY PERMUTED (1-3,1-4)-BETA-D-GLUCAN 4								

123	* 1s2b:A (201)	Scytalidopepsin B	80	39	0.0004	0.31	3.23	118	3.4% : 4.2% (2/58 : 5/118)	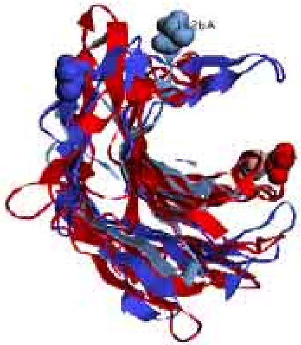
	* 1ajo:A (212)	CIRCULARLY PERMUTED (1-3,1-4)-BETA-D-GLUCAN 4								
124	* 1uai:A (223)	polyguluronate lyase	141	52	5E-08	0.33	2.96	128	8.6% : 11.7% (5/58 : 15/128)	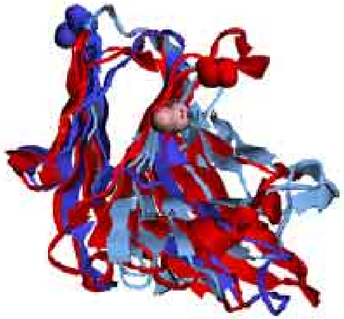
	* 1ajo:A (212)	CIRCULARLY PERMUTED (1-3,1-4)-BETA-D-GLUCAN 4								
125	* 1ajo:A (212)	CIRCULARLY PERMUTED (1-3,1-4)-BETA-D-GLUCAN 4	111	70	2E-13	0.40	1.69	157	28.6% : 29.9% (24/84 : 47/157)	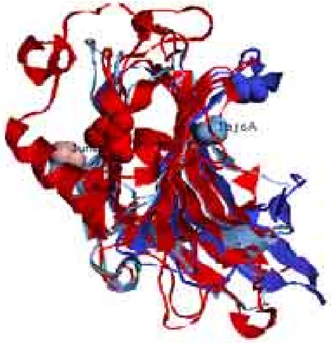
	* 1umz:A (267)	XYLOGLUCAN ENDOTRANSGLYCOSYLASE								

126	* 1ajo:A (212)	CIRCULARLY PERMUTED (1-3,1-4)-BETA-D-GLUCAN 4	**131**	38	0.001	0.42	3.04	107	19.3% : 10.3% (11/57 : 11/107)	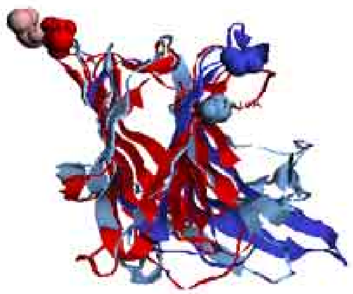
	* 1ww4:A (160)	galectin								
127	* 1x50:A (164)	Galectin-4	**93**	34	0.009	0.37	3.05	106	10.0% : 11.3% (6/60 : 12/106)	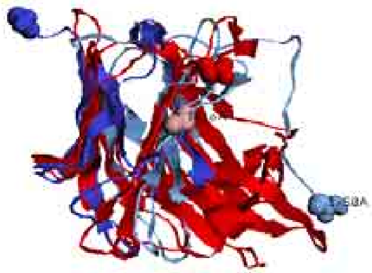
	* 1ajo:A (212)	CIRCULARLY PERMUTED (1-3,1-4)-BETA-D-GLUCAN 4								
128	* 1y43:B (171)	Aspergillopepsin II heavy chain	**45**	38	0.0004	0.26	3.41	108	1.7% : 10.2% (1/60 : 11/108)	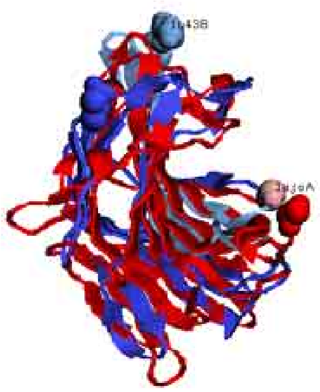
	* 1ajo:A (212)	CIRCULARLY PERMUTED (1-3,1-4)-BETA-D-GLUCAN 4								

129	* 1ajo:A (212)	CIRCULARLY PERMUTED (1-3,1-4)-BETA-D-GLUCAN 4	**102**	37	0.002	0.40	2.12	132	6.0% : 14.4% (3/50 : 19/132)	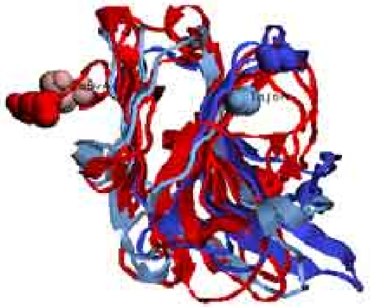
	* 2a6v:A (218)	Emp46p								
130	* 2a6y:A (232)	Emp47p (form1)	**139**	49	5E-07	0.44	2.72	144	5.3% : 12.5% (3/57 : 18/144)	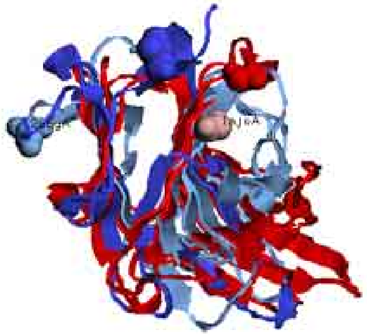
	* 1ajo:A (212)	CIRCULARLY PERMUTED (1-3,1-4)-BETA-D-GLUCAN 4								
131	* 1ajo:A (212)	CIRCULARLY PERMUTED (1-3,1-4)-BETA-D-GLUCAN 4	**88**	39	0.0003	0.47	2.96	148	10.7% : 8.1% (9/84 : 12/148)	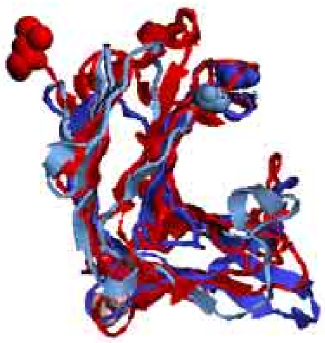
	* 2cws:A (227)	alginate lyase A1-II'								

132	* 2d6k:A (148)	lectin, galactose binding, soluble 9	**80**	40	0.0001	0.40	3.09	101	9.5% : 10.9% (6/63 : 11/101)	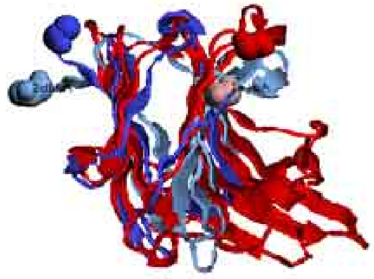
	* 1ajo:A (212)	CIRCULARLY PERMUTED (1-3,1-4)-BETA-D-GLUCAN 4								
133	* 1ajo:A (212)	CIRCULARLY PERMUTED (1-3,1-4)-BETA-D-GLUCAN 4	**137**	38	0.001	0.41	2.92	121	7.3% : 13.2% (4/55 : 16/121)	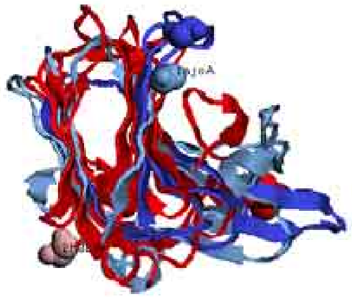
	* 2h0b:A (184)	Neurexin-1-alpha								
134	1al3:_ (237)	CYS REGULON TRANSCRIPTIONAL ACTIVATOR CYSB	**75**	34	0.014	0.28	3.08	130	4.9% : 13.8% (2/41 : 18/130)	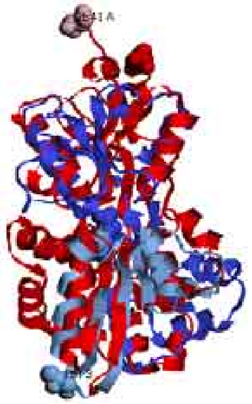
	2b4l:A (268)	Glycine betaine-binding protein								

135	* 1blc:_ (260)	BETA-LACTAMASE	220	379	0	0.44	0.35	252	100.0% : 100.0% (220/220 : 252/252)	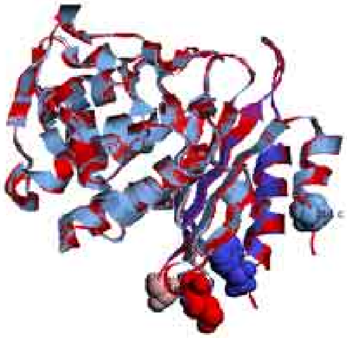
	* 1alq_ (264)	CP254 BETA-LACTAMASE								
136	* 1bsg:_ (265)	BETA LACTAMASE	228	115	6E-27	0.42	2.00	249	32.1% : 31.3% (69/215 : 78/249)	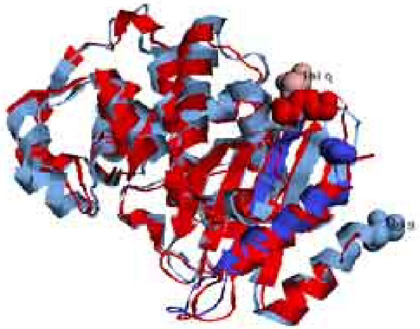
	* 1alq_ (264)	CP254 BETA-LACTAMASE								
137	* 1bue:A (266)	PROTEIN (IMIPENEM-HYDROLYSING BETA-LACTAMASE)	227	128	9E-31	0.42	1.66	244	37.1% : 35.2% (78/210 : 86/244)	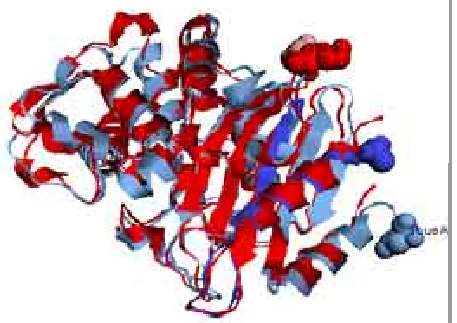
	* 1alq_ (264)	CP254 BETA-LACTAMASE								

138	* 1bza:_ (260)	BETA-LACTAMASE	**225**	128	5E-31	0.40	1.65	243	37.9% : 35.8% (80/211 : 87/243)	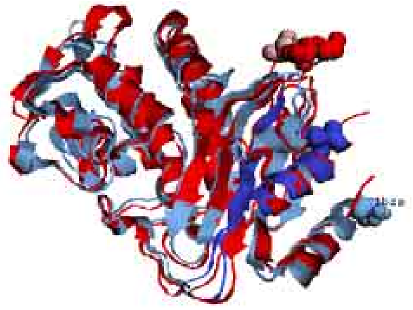
	* 1alq_ (264)	CP254 BETA-LACTAMASE								
139	* 1dy6:A (268)	CARBAPENEM-HYDROLYSING BETA-LACTAMASE SME-1	**229**	143	3E-35	0.44	1.80	246	37.6% : 35.8% (79/210 : 88/246)	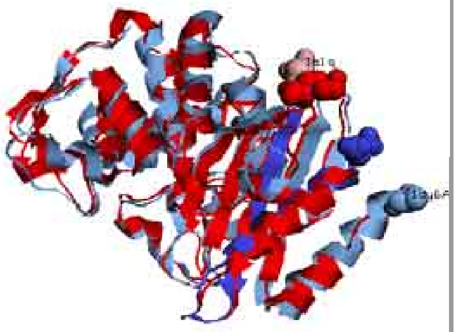
	* 1alq_ (264)	CP254 BETA-LACTAMASE								
140	* 1hzo:A (267)	BETA-LACTAMASE	**227**	127	2E-30	0.44	1.69	248	38.2% : 36.3% (81/212 : 90/248)	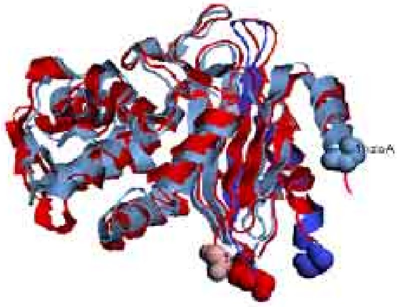
	* 1alq_ (264)	CP254 BETA-LACTAMASE								

141	* 2cc1:A (267)	BETA-LACTAMASE	224	108	7E-25	0.42	1.78	243	35.4% : 33.3% (74/209 : 81/243)	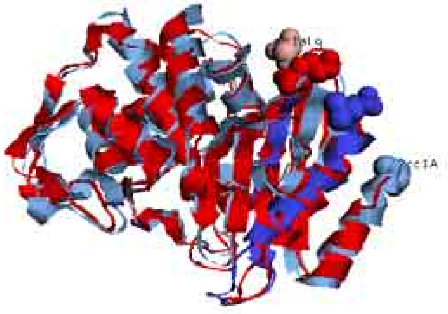
	* 1alq_ (264)	CP254 BETA-LACTAMASE								
142	* 2gdn:A (265)	Beta-lactamase	227	119	5E-28	0.40	1.86	246	35.0% : 32.9% (75/214 : 81/246)	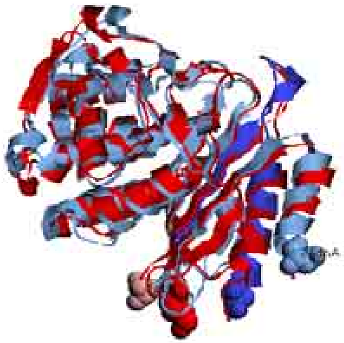
	* 1alq_ (264)	CP254 BETA-LACTAMASE								
143	1dps:A (159)	DPS	51	38	0.0006	0.41	2.82	114	7.8% : 8.8% (7/90 : 10/114)	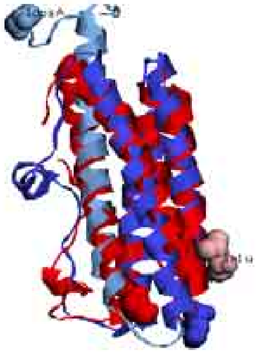
	1alu_ (158)	INTERLEUKIN-6								

144	1fha:_<br>(173)<br>1alu_<br>(158)	FERRITIN<br>INTERLEUKIN-6	**43**	40	0.0001	0.34	3.42	112	10.9% : 8.9%<br>(6/55 : 10/112)	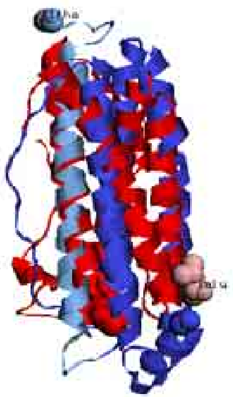
145	1h96:A<br>(167)<br>1alu_<br>(158)	FERRITIN LIGHT CHAIN 1<br>INTERLEUKIN-6	**43**	38	0.0006	0.34	3.25	109	10.0% : 8.3%<br>(5/50 : 9/109)	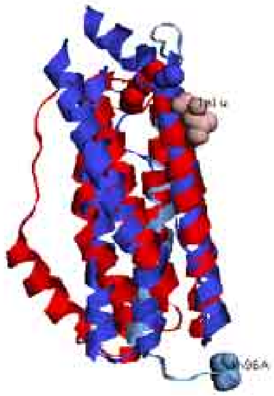
146	1alu:_<br>(158)<br>1jaf:A<br>(128)	INTERLEUKIN-6<br>CYTOCHROME C'	**126**	36	0.002	0.21	2.35	73	5.8% : 5.5%<br>(4/69 : 4/73)	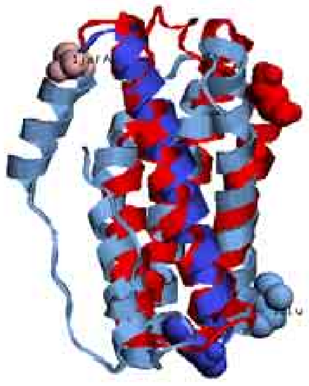

147	1alu:_ (158)	INTERLEUKIN-6	**77**	39	0.0003	0.20	2.11	59	7.1% : 8.5% (4/56 : 5/59)	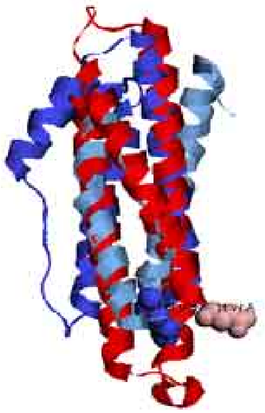
	1lvf:A (106)	syntaxin 6								
148	1moj:A (180)	Dps-like ferritin	**57**	33	0.024	0.33	3.15	115	12.4% : 5.2% (11/89 : 6/115)	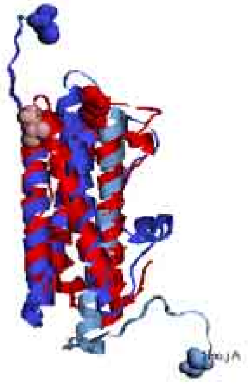
	1alu_ (158)	INTERLEUKIN-6								
149	1alu:_ (158)	INTERLEUKIN-6	**54**	36	0.002	0.28	3.06	85	12.9% : 10.6% (8/62 : 9/85)	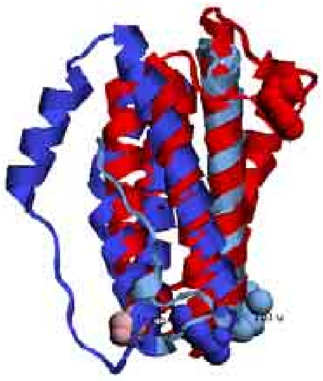
	1nog:A (149)	conserved hypothetical protein TA0546								

150	1alu:_ (158)	INTERLEUKIN-6	**118**	44	1E-05	0.33	3.33	112	7.4% : 8.0% (5/68 : 9/112)	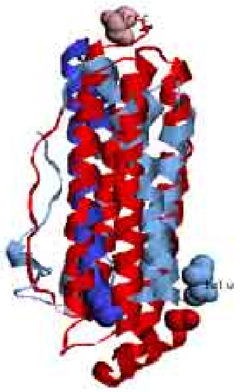
	1rcc_ (171)	L FERRITIN								
151	1alu:_ (158)	INTERLEUKIN-6	**100**	35	0.003	0.33	3.36	109	10.4% : 4.6% (7/67 : 5/109)	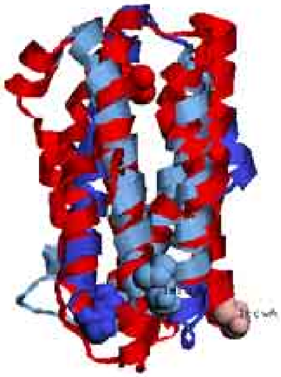
	1rcw:A (213)	CT610								
152	1rfy:A (89)	Transcriptional repressor traM	**53**	33	0.013	0.41	1.74	59	5.4% : 6.8% (3/56 : 4/59)	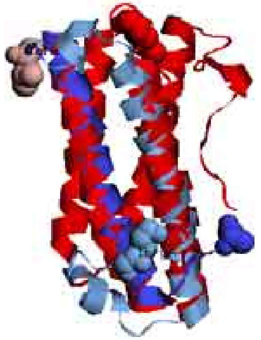
	1alu_ (158)	INTERLEUKIN-6								

153	1alu:_ (158)	INTERLEUKIN-6	**51**	37	0.001	0.35	3.46	103	7.9% : 10.7% (5/63 : 11/103)	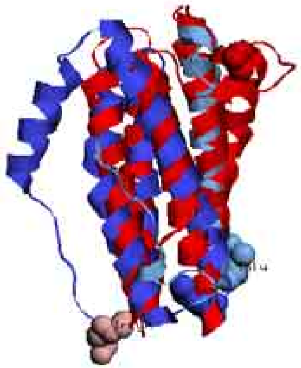
	1rty:A (159)	yvqk protein								
154	1alu:_ (158)	INTERLEUKIN-6	**123**	43	2E-05	0.40	3.48	114	11.9% : 9.6% (10/84 : 11/114)	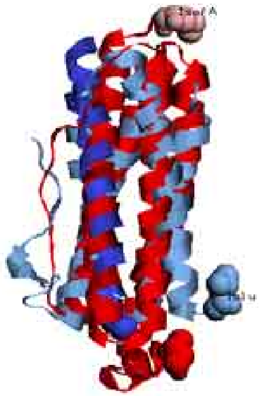
	1sof:A (155)	Bacterioferritin								
155	1alu:_ (158)	INTERLEUKIN-6	**98**	49	4E-07	0.26	3.13	102	6.0% : 5.9% (3/50 : 6/102)	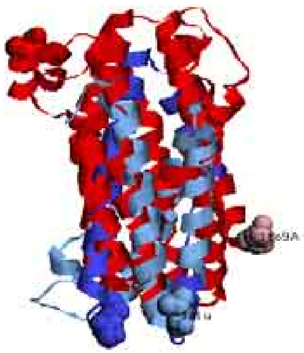
	1to9:A (225)	THI-4 protein								

156	1alu:_ (158)	INTERLEUKIN-6	**121**	37	0.001	0.37	2.61	107	6.1% : 8.4% (4/66 : 9/107)	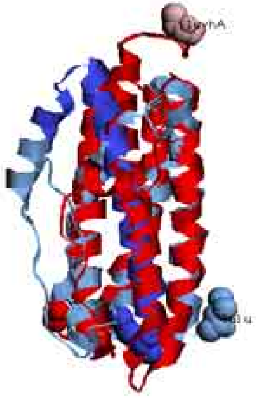
	1uvh:A (157)	STARVATION-INDUCED DNA PROTECTING PROTEIN								
157	1vjx:A (148)	putative ferritin-like diiron-carboxylate pro	**36**	41	7E-05	0.34	2.95	103	10.7% : 9.7% (9/84 : 10/103)	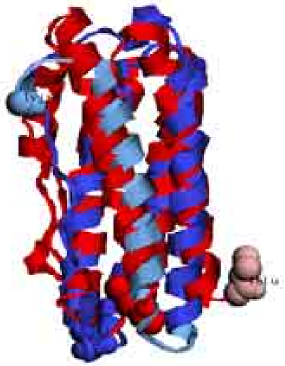
	1alu_ (158)	INTERLEUKIN-6								
158	1alu:_ (158)	INTERLEUKIN-6	**53**	37	0.0008	0.29	2.95	93	7.4% : 12.9% (4/54 : 12/93)	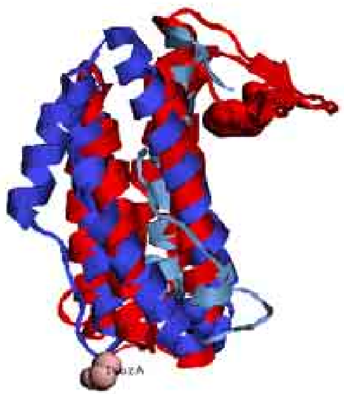
	1woz:A (159)	177aa long conserved hypothetical protein (ST								

159	1alu:_ (158)	INTERLEUKIN-6	**53**	40	0.0001	0.28	3.32	94	11.1% : 9.6% (7/63 : 9/94)	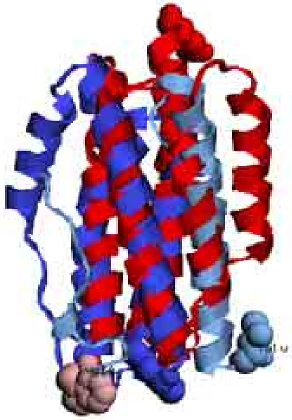
	1wy1:A (142)	hypothetical protein PH0671								
160	1alu:_ (158)	INTERLEUKIN-6	**99**	36	0.002	0.24	2.70	77	13.2% : 7.8% (10/76 : 6/77)	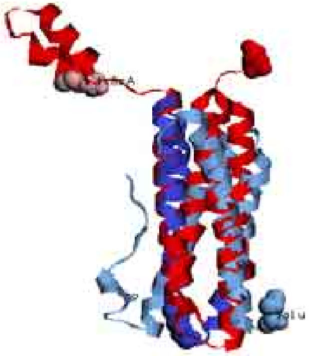
	1x8z:A (151)	invertase/pectin methylesterase inhibitor fam								
161	1alu:_ (158)	INTERLEUKIN-6	**56**	43	2E-05	0.35	2.90	97	12.3% : 9.3% (8/65 : 9/97)	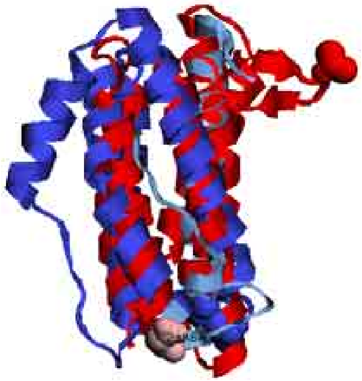
	2ah6:A (152)	BH1595, unknown conserved protein								

162	1alu:_<br>(158)	INTERLEUKIN-6	**47**	34	0.012	0.22	3.15	76	8.3% : 9.2%<br>(6/72 : 7/76)	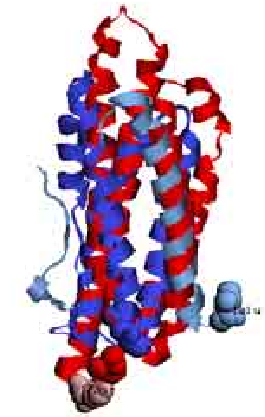
	2asr_<br>(142)	ASPARTATE RECEPTOR								
163	2c41:A<br>(155)	DPS FAMILY DNA-BINDING STRESS RESPONSE PROTEI	**38**	35	0.005	0.42	3.17	119	5.6% : 5.0%<br>(3/54 : 6/119)	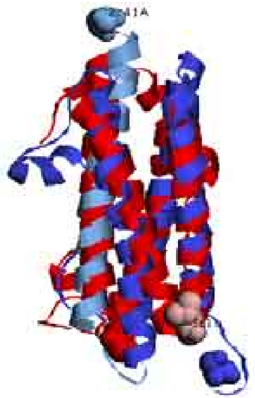
	1alu_<br>(158)	INTERLEUKIN-6								
164	1alu:_<br>(158)	INTERLEUKIN-6	**113**	50	1E-07	0.34	2.62	109	11.5% : 4.6%<br>(9/78 : 5/109)	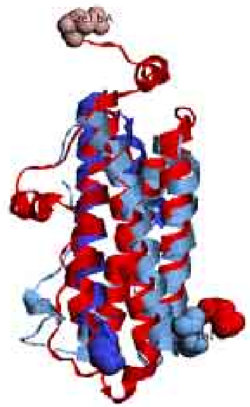
	2clb:A<br>(169)	DPS-LIKE PROTEIN								

165	1alu:_ (158)	INTERLEUKIN-6	**49**	45	5E-06	0.40	3.18	113	7.8% : 8.0% (4/51 : 9/113)	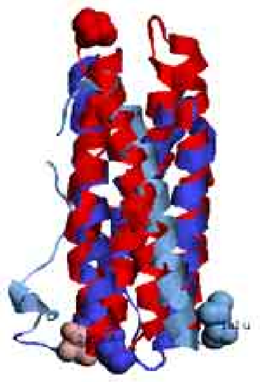
	2d5k:A (148)	Dps family protein								
166	1alu:_ (158)	INTERLEUKIN-6	**117**	48	6E-07	0.32	3.37	107	9.2% : 10.3% (7/76 : 11/107)	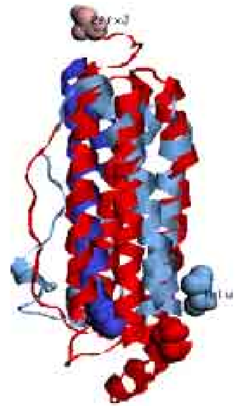
	2ffx:J (173)	ferritin light chain								
167	1alu:_ (158)	INTERLEUKIN-6	**46**	43	2E-05	0.39	3.46	114	3.2% : 7.9% (2/62 : 9/114)	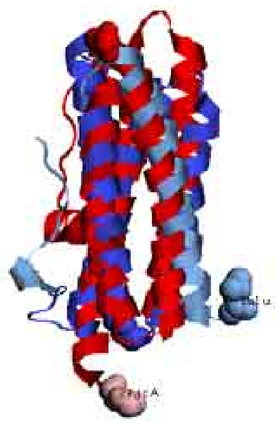
	2fjc:A (151)	Antigen TpF1								

168	1alu:_<br>(158)<br>2fzf:A<br>(158)	INTERLEUKIN-6<br>hypothetical protein	**120**	34	0.007	0.42	3.15	113	10.5% : 7.1%<br>(6/57 : 8/113)	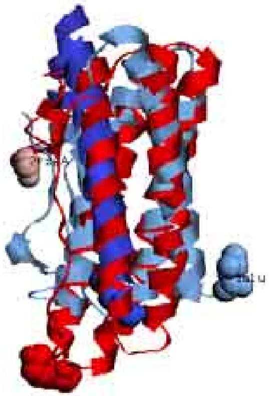
169	1alu:_<br>(158)<br>2gs4:A<br>(159)	INTERLEUKIN-6<br>Protein yciF	**47**	54	1E-08	0.43	3.46	121	8.2% : 7.4%<br>(5/61 : 9/121)	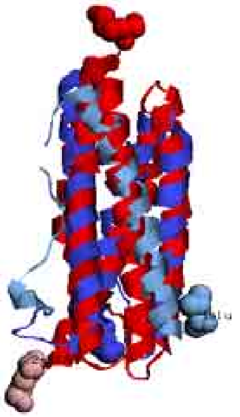
170	1alu:_<br>(158)<br>2gsc:A<br>(114)	INTERLEUKIN-6<br>conserved hypothetical protein	**75**	35	0.003	0.32	2.44	75	4.2% : 6.7%<br>(2/48 : 5/75)	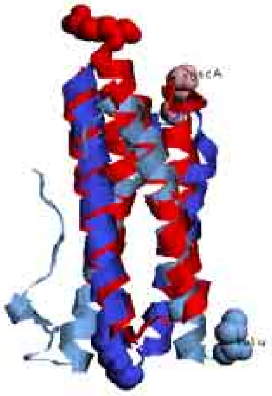

171	1alu:_ (158)	INTERLEUKIN-6	**38**	51	5E-08	0.35	3.41	116	6.7% : 7.8% (6/89 : 9/116)	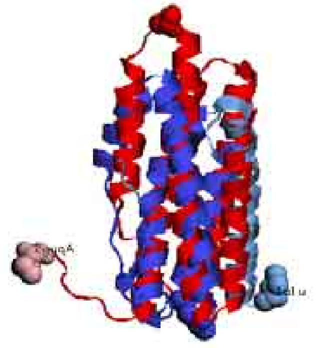
	2gyq:A (162)	ycfI, putative structural protein								
172	2hjd:A (89)	Quorum-sensing antiactivator	**55**	34	0.006	0.40	1.37	58	7.5% : 6.9% (4/53 : 4/58)	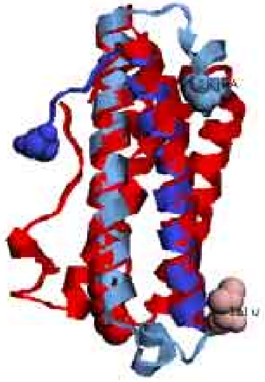
	1alu_ (158)	INTERLEUKIN-6								
173	1alu:_ (158)	INTERLEUKIN-6	**129**	49	3E-07	0.41	3.20	106	3.8% : 7.5% (2/53 : 8/106)	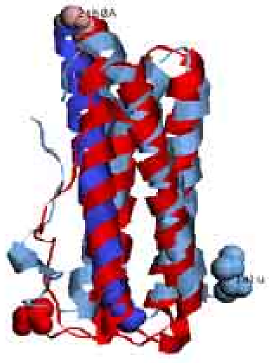
	2ib0:A (142)	CONSERVED HYPOTHETICAL ALANINE RICH PROTEIN								

174	1amf:_<br>(231)<br>2b4l:A<br>(268)	MOLYBDATE TRANSPORT PROTEIN MODA<br>Glycine betaine-binding protein	**69**	32	0.046	0.37	3.43	151	11.7% : 13.9%<br>(16/137 : 21/151)	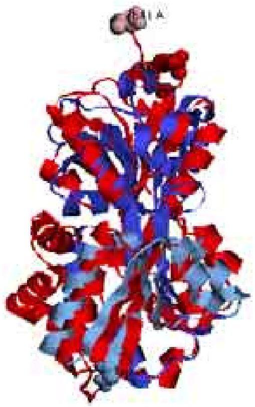
175	1nps:A<br>(87)<br>1amm_<br>(174)	DEVELOPMENT-SPECIFIC PROTEIN S<br>GAMMA B-CRYSTALLIN	**47**	34	0.006	0.30	1.68	80	23.0% : 28.8%<br>(17/74 : 23/80)	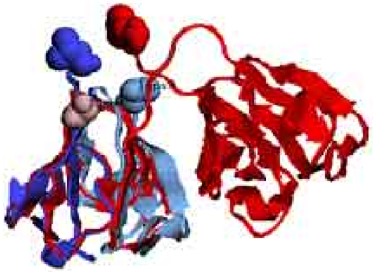
176	1amx:_<br>(150)<br>1u5x:A<br>(132)	COLLAGEN ADHESIN<br>Tumor necrosis factor ligand superfamily memb	**77**	32	0.026	0.31	3.27	82	6.0% : 7.3%<br>(3/50 : 6/82)	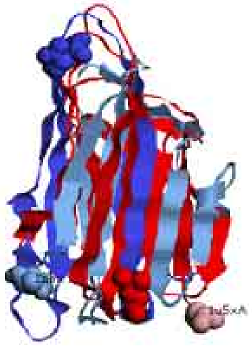

177	1aoh:A (143)	CELLULOSOME-INTEGRATING PROTEIN CIPA	**96**	32	0.026	0.20	2.98	56	9.6% : 7.1% (5/52 : 4/56)	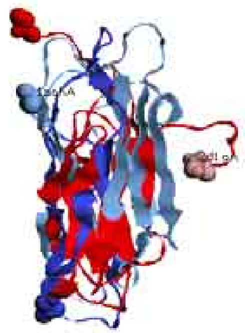
	2dlg:A (102)	Filamin-B								
178	1ap9:_ (218)	BACTERIORHODOPSIN	**122**	31	0.089	0.25	3.20	96	7.7% : 4.2% (6/78 : 4/96)	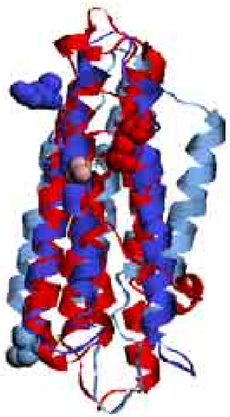
	1qkr:A (181)	VINCULIN								
179	1apc:_ (106)	CYTOCHROME B562	**40**	32	0.043	0.28	3.44	59	2.3% : 10.2% (1/43 : 6/59)	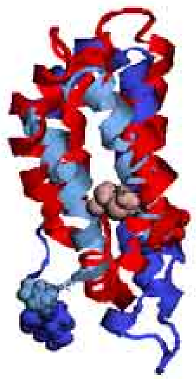
	1y6d:A (114)	Phosphorelay protein luxU								

180	1apn:A (226)<br>1avb:A (226)	CONCANAVALIN A<br>ARCELIN-1	**117**	106	2E-24	0.86	1.86	208	36.9% : 38.5% (38/103 : 80/208)	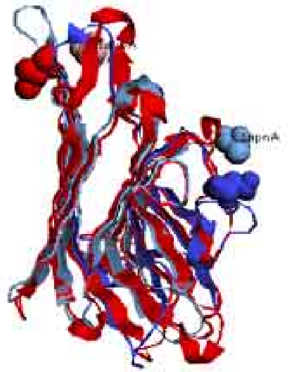
181	1apn:A (226)<br>1ax0_ (239)	CONCANAVALIN A<br>LECTIN	**117**	119	2E-28	0.85	1.67	211	44.0% : 41.7% (48/109 : 88/211)	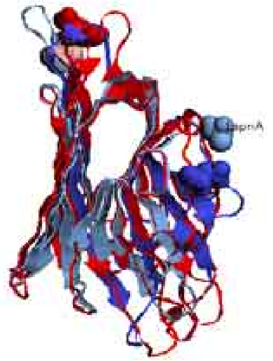
182	1apn:A (226)<br>1bjq:A (252)	CONCANAVALIN A<br>LECTIN	**117**	117	1E-27	0.75	1.53	208	44.4% : 43.3% (48/108 : 90/208)	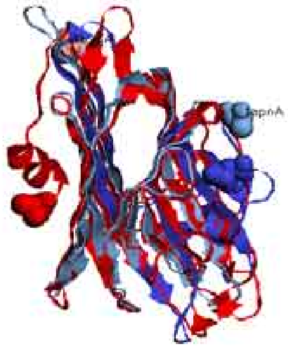

183	1apn:A (226)	CONCANAVALIN A	**117**	104	8E-24	0.73	1.66	164	42.6% : 41.5% (43/101 : 68/164)	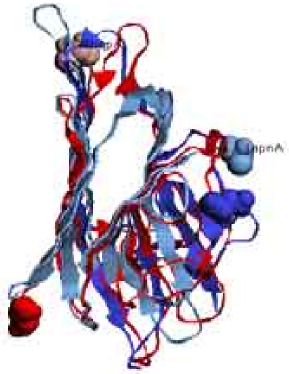
	1bqp:A (181)	PROTEIN (LECTIN)								
184	1apn:A (226)	CONCANAVALIN A	**117**	122	3E-29	0.82	1.77	205	46.3% : 46.3% (50/108 : 95/205)	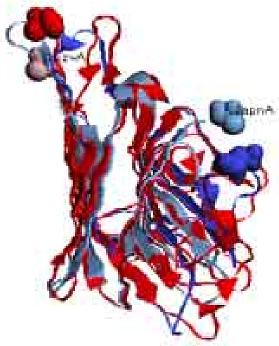
	1bzw:A (232)	PROTEIN (PEANUT LECTIN)								
185	1apn:A (226)	CONCANAVALIN A	**117**	117	8E-28	0.83	1.46	208	52.9% : 49.0% (55/104 : 102/208)	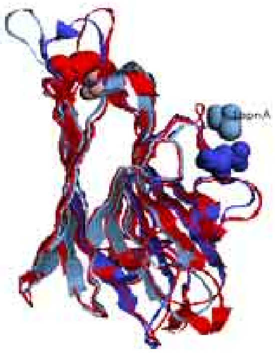
	1dbn:A (239)	PROTEIN (LEUKOAGGLUTININ)								

186	1apn:A (226)	CONCANAVALIN A	**117**	58	6E-10	0.63	1.86	172	40.8% : 41.3% (40/98 : 71/172)	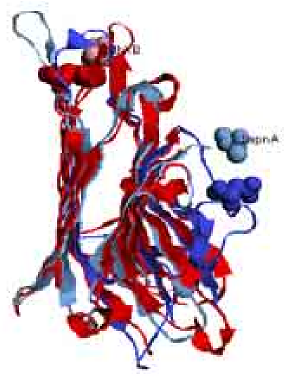
	1dhk:B (197)	BEAN LECTIN-LIKE INHIBITOR								
187	1apn:A (226)	CONCANAVALIN A	**117**	120	1E-28	0.84	1.35	207	48.1% : 47.3% (50/104 : 98/207)	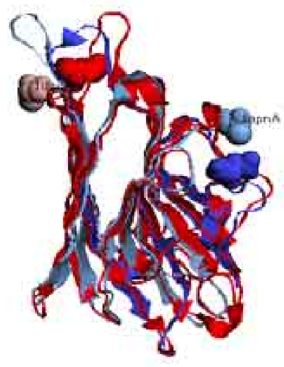
	1dzq:A (235)	ULEX EUROPAEUS LECTIN II (UEA-II)								
188	1apn:A (226)	CONCANAVALIN A	**118**	95	8E-21	0.80	1.66	204	48.6% : 45.6% (52/107 : 93/204)	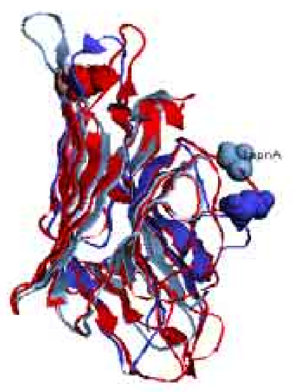
	1f9k:A (234)	ACIDIC LECTIN								

189	1apn:A (226)	CONCANAVALIN A	117	110	2E-25	0.79	1.62	201	50.9% : 47.3% (55/108 : 95/201)	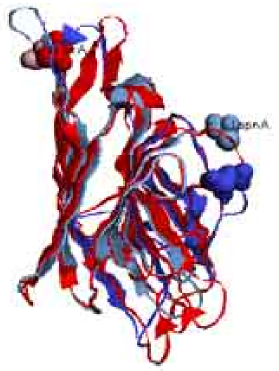
	1fat:A (233)	PHYTOHEMAGGLUTININ-L								
190	1apn:A (226)	CONCANAVALIN A	117	117	1E-27	0.83	1.53	208	46.3% : 46.6% (50/108 : 97/208)	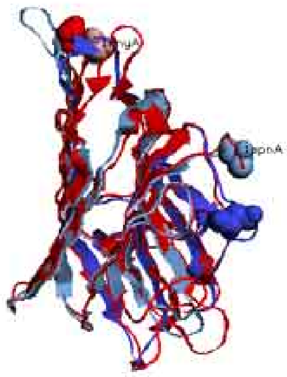
	1fny:A (235)	BARK AGGLUTININ I,POLYPEPTIDE A								
191	1apn:A (226)	CONCANAVALIN A	117	106	2E-24	0.78	1.39	203	44.8% : 41.4% (47/105 : 84/203)	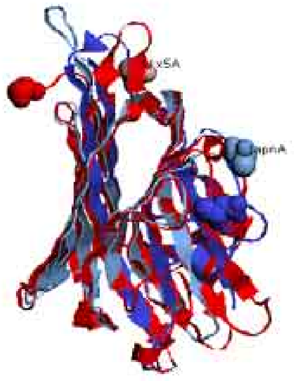
	1fx5:A (241)	ANTI-H(O) LECTIN I								

192	1apn:A (226)	CONCANAVALIN A	**117**	113	2E-26	0.75	1.42	206	46.7% : 43.7% (50/107 : 90/206)	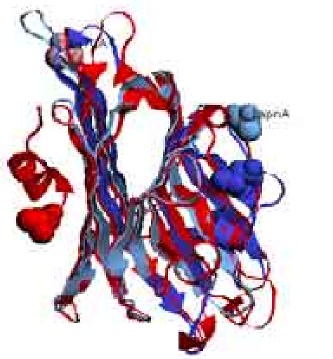
	1g7y:A (252)	STEM/LEAF LECTIN DB58								
193	1g86:A (141)	CHARCOT-LEYDEN CRYSTAL PROTEIN	**31**	37	0.0007	0.21	2.42	89	10.5% : 11.2% (6/57 : 10/89)	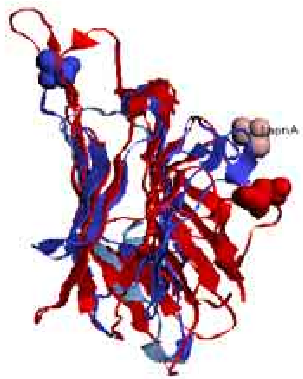
	1apn:A (226)	CONCANAVALIN A								
194	1apn:A (226)	CONCANAVALIN A	**117**	116	2E-27	0.79	1.45	207	47.2% : 46.4% (51/108 : 96/207)	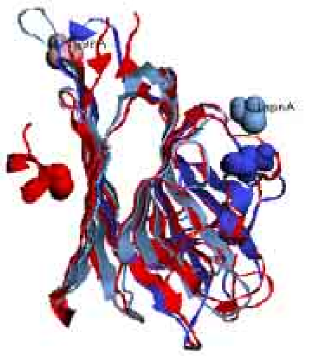
	1g9f:A (242)	LECTIN								

195	1apn:A (226)	CONCANAVALIN A	**118**	103	2E-23	0.76	1.45	197	47.1% : 45.7% (49/104 : 90/197)	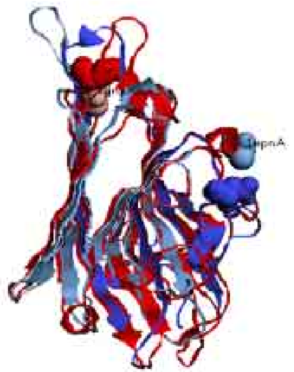
	1gnz:A (236)	LECTIN								
196	1apn:A (226)	CONCANAVALIN A	**113**	99	2E-22	0.79	1.67	205	45.2% : 43.9% (47/104 : 90/205)	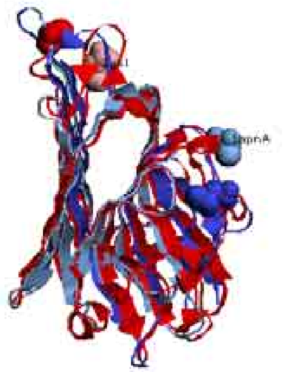
	1gsl_ (243)	GRIFFONIA SIMPLICIFOLIA LECTIN 4								
197	1apn:A (226)	CONCANAVALIN A	**109**	35	0.006	0.58	2.84	170	15.9% : 17.6% (13/82 : 30/170)	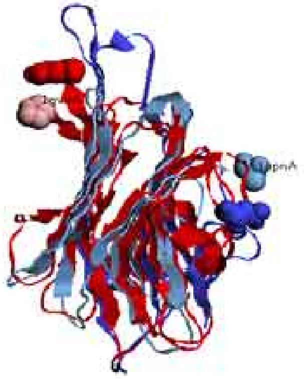
	1gv9:A (224)	P58/ERGIC-53								

198	1apn:A (226)	CONCANAVALIN A	**117**	98	8E-22	0.83	1.52	205	31.4% : 36.1% (33/105 : 74/205)	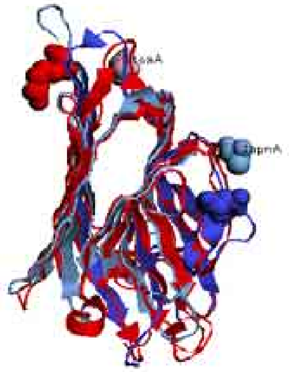
	1ioa:A (228)	ARCELIN-5A								
199	1is3:A (134)	CONGERIN II	**42**	45	3E-06	0.29	2.10	89	9.4% : 10.1% (6/64 : 9/89)	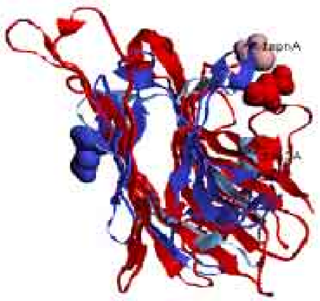
	1apn:A (226)	CONCANAVALIN A								
200	* 1mve:A (243)	Truncated 1,3-1,4-beta-D-glucanase	**52**	37	0.002	0.22	3.11	131	8.2% : 10.7% (8/97 : 14/131)	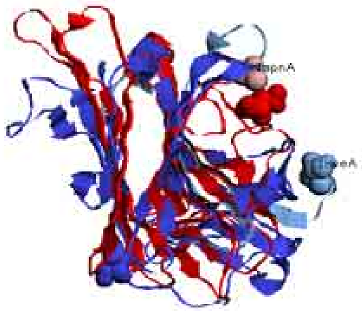
	* 1apn:A (226)	CONCANAVALIN A								

201	1apn:A (226)	CONCANAVALIN A	**117**	131	5E-32	0.84	1.56	210	46.2% : 49.5% (49/106 : 104/210)	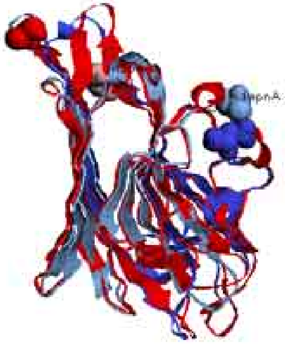
	1n3o:A (241)	lectin PAL								
202	1apn:A (226)	CONCANAVALIN A	**117**	126	3E-30	0.87	1.42	211	45.4% : 44.1% (49/108 : 93/211)	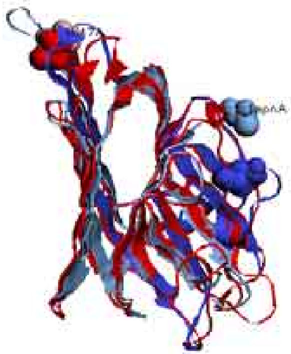
	1n47:A (233)	Isolectin B4								
203	1apn:A (226)	CONCANAVALIN A	**110**	40	0.0002	0.35	3.25	130	22.6% : 7.7% (14/62 : 10/130)	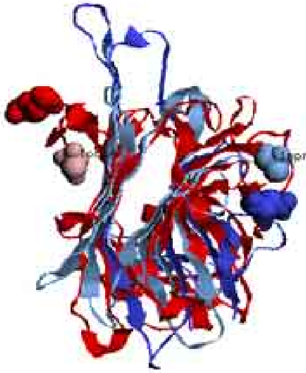
	1oq1:A (223)	Protein yesU								

204	1sla:A<br>(134)	BOVINE GALECTIN-1	**43**	37	0.0008	0.29	2.17	90	14.1% : 10.0%<br>(9/64 : 9/90)	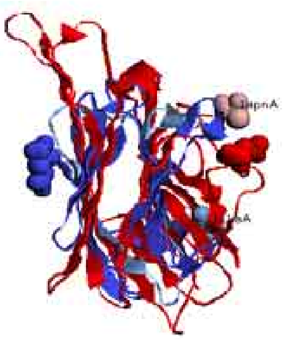
	1apn:A<br>(226)	CONCANAVALIN A								
205	1apn:A<br>(226)	CONCANAVALIN A	**118**	104	8E-24	0.77	1.49	202	46.7% : 45.0%<br>(50/107 : 91/202)	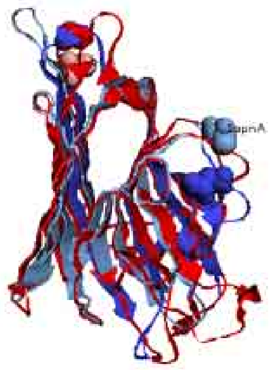
	1wbf:A<br>(237)	PROTEIN (AGGLUTININ)								
206	1apn:A<br>(226)	CONCANAVALIN A	**111**	56	3E-09	0.54	2.46	163	17.2% : 16.6%<br>(15/87 : 27/163)	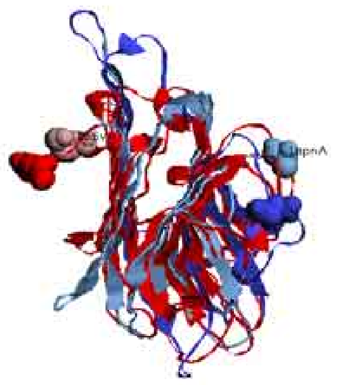
	2a6v:A<br>(218)	Emp46p								

207	1apn:A (226)	CONCANAVALIN A	111	46	4E-06	0.48	2.18	159	19.5% : 18.9% (16/82 : 30/159)	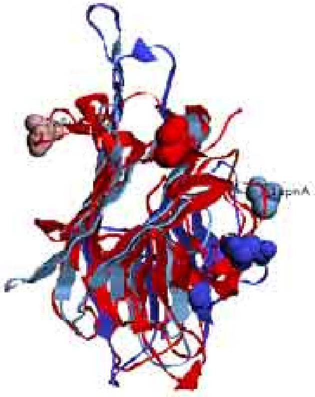
	2a6y:A (232)	Emp47p (form1)								
208	1apn:A (226)	CONCANAVALIN A	117	96	2E-21	0.71	1.46	161	39.2% : 40.4% (40/102 : 65/161)	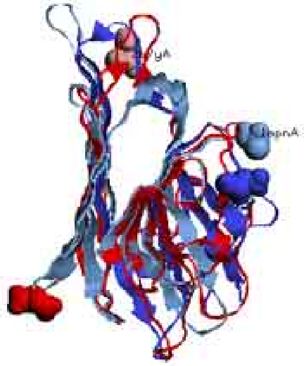
	2b7y:A (181)	Favin beta chain								
209	1apn:A (226)	CONCANAVALIN A	117	153	2E-38	0.88	1.16	212	61.7% : 58.5% (66/107 : 124/212)	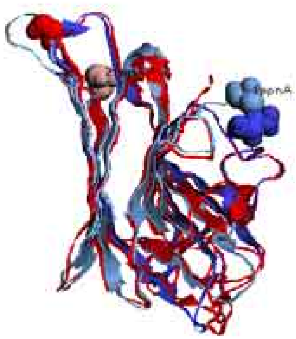
	2fmd:A (233)	Lectin								

210	* 1ark:_ (60)	NEBULIN	**46**	32	0.027	0.67	1.37	53	26.3% : 30.2% (10/38 : 16/53)	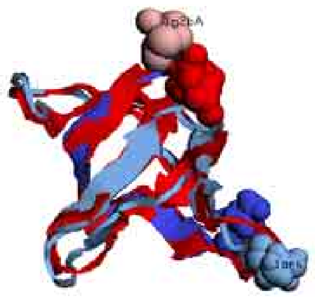
	* 1g2b:A (62)	SPECTRIN ALPHA CHAIN								
211	1au1:A (166)	INTERFERON-BETA	**35**	32	0.037	0.25	3.13	101	7.8% : 6.9% (6/77 : 7/101)	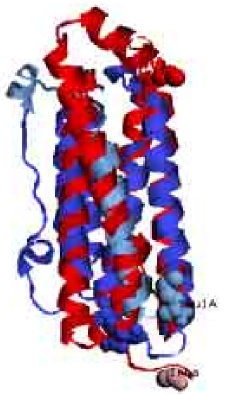
	1fha_ (173)	FERRITIN								
212	1au1:A (166)	INTERFERON-BETA	**36**	34	0.007	0.25	3.07	101	7.7% : 5.0% (6/78 : 5/101)	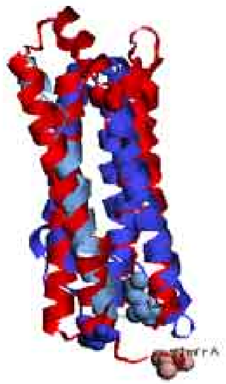
	1mfr:A (171)	M FERRITIN								

213	1au1:A (166)	INTERFERON-BETA	**139**	34	0.007	0.26	3.29	133	10.6% : 10.5% (11/104 : 14/133)	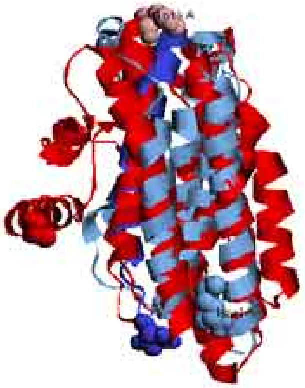
	1otk:A (244)	Phenylacetic acid degradation protein paaC								
214	1au1:A (166)	INTERFERON-BETA	**37**	36	0.003	0.21	2.87	130	6.7% : 7.7% (7/104 : 10/130)	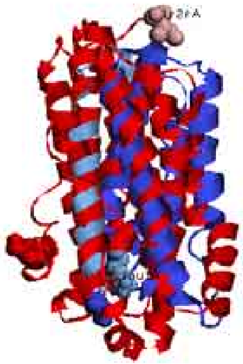
	1r2f:A (283)	PROTEIN (RIBONUCLEOTIDE REDUCTASE R2)								
215	1t6i:A (111)	Superoxide dismutase [Ni]	**65**	39	0.0002	0.23	2.22	65	3.7% : 3.1% (2/54 : 2/65)	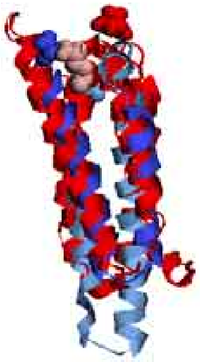
	1au1:A (166)	INTERFERON-BETA								

216	1tqg:A (105)	Chemotaxis protein cheA	**31**	37	0.001	0.25	1.31	56	5.3% : 7.1% (2/38 : 4/56)	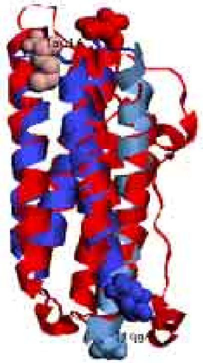
	1au1:A (166)	INTERFERON-BETA								
217	1umn:A (151)	DPS-LIKE PEROXIDE RESISTANCE PROTEIN	**76**	35	0.003	0.24	3.23	81	7.8% : 16.0% (4/51 : 13/81)	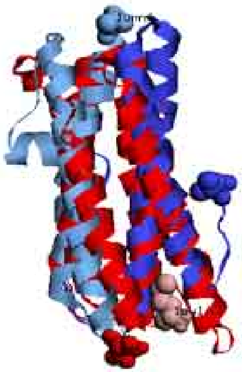
	1au1:A (166)	INTERFERON-BETA								
218	1au1:A (166)	INTERFERON-BETA	**36**	34	0.012	0.22	2.86	132	6.2% : 6.1% (4/65 : 8/132)	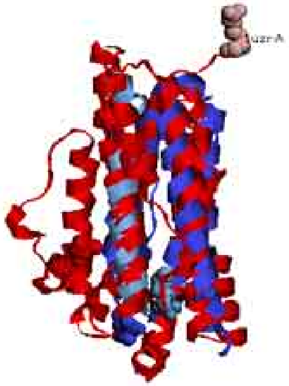
	1uzr:A (282)	RIBONUCLEOTIDE REDUCTASE R2-2 SMALL SUBUNIT								

219	1au1:A (166)	INTERFERON-BETA	**135**	41	8E-05	0.36	3.25	105	8.5% : 6.7% (6/71 : 7/105)	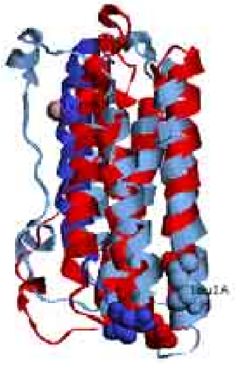
	1vjx:A (148)	putative ferritin-like diiron-carboxylate pro								
220	1wvt:A (148)	hypothetical protein ST2180	**39**	34	0.006	0.26	3.17	82	8.1% : 3.7% (5/62 : 3/82)	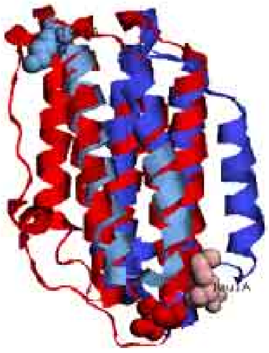
	1au1:A (166)	INTERFERON-BETA								
221	1au1:A (166)	INTERFERON-BETA	**107**	32	0.037	0.20	2.29	100	3.8% : 4.0% (2/53 : 4/100)	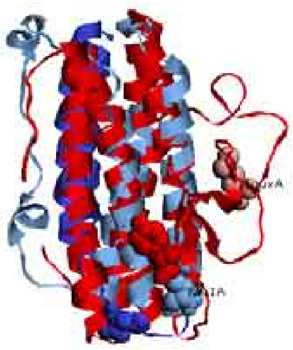
	1yux:A (201)	Nigerythrin								

222	1zuj:A (168)	hypothetical protein Llacc01001955	**57**	31	0.064	0.27	2.42	93	12.8% : 9.7% (6/47 : 9/93)	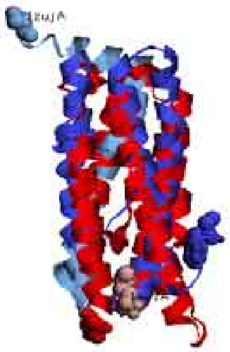
	1au1:A (166)	INTERFERON-BETA								
223	2ah6:A (152)	BH1595, unknown conserved protein	**39**	34	0.006	0.27	2.96	88	7.5% : 6.8% (5/67 : 6/88)	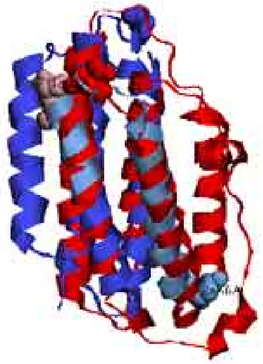
	1au1:A (166)	INTERFERON-BETA								
224	2c41:A (155)	DPS FAMILY DNA-BINDING STRESS RESPONSE PROTEI	**77**	36	0.002	0.29	3.05	87	3.6% : 9.2% (2/56 : 8/87)	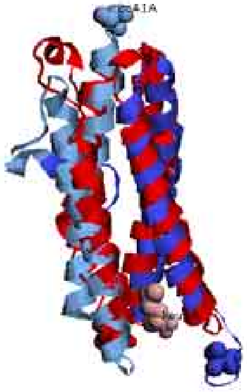
	1au1:A (166)	INTERFERON-BETA								

225	1au1:A (166)	INTERFERON-BETA	139	32	0.037	0.20	2.14	84	4.3% : 7.1% (2/46 : 6/84)	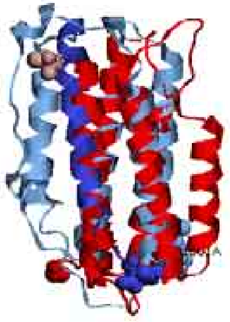
	2g2d:A (160)	ATP:cobalamin adenosyltransferase								
226	2gyq:A (162)	ycfI, putative structural protein	132	36	0.002	0.30	2.62	111	6.7% : 10.8% (4/60 : 12/111)	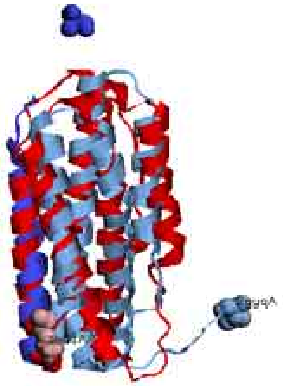
	1au1:A (166)	INTERFERON-BETA								
227	1au1:A (166)	INTERFERON-BETA	113	38	0.0007	0.28	3.13	107	9.9% : 6.5% (8/81 : 7/107)	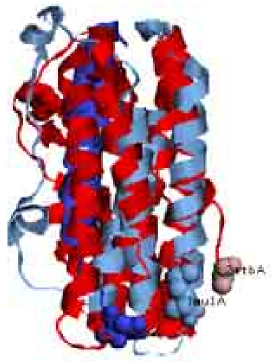
	2itb:A (199)	TRNA-(Ms(2)io(6)a)-hydroxylase, putative								

228	1au1:A (166)	INTERFERON-BETA	**118**	33	0.021	0.22	2.77	94	15.7% : 6.4% (8/51 : 6/94)	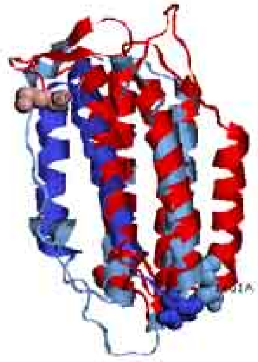
	2nt8:A (181)	Cobalamin adenosyltransferase								
229	1aue:A (92)	FKBP-RAPAMYCIN ASSOCIATED PROTEIN	**66**	35	0.005	0.26	3.42	59	3.6% : 6.8% (2/55 : 4/59)	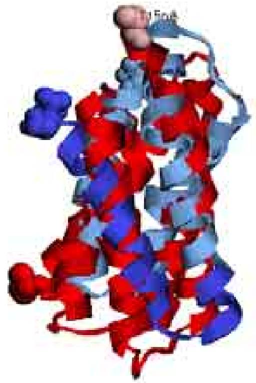
	1i5n:A (128)	CHEMOTAXIS PROTEIN CHEA								
230	1aue:A (92)	FKBP-RAPAMYCIN ASSOCIATED PROTEIN	**38**	36	0.002	0.44	3.10	65	2.2% : 3.1% (1/46 : 2/65)	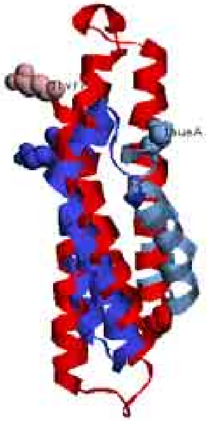
	1lvf:A (106)	syntaxin 6								

231	1aue:A (92)	FKBP-RAPAMYCIN ASSOCIATED PROTEIN	**38**	34	0.01	0.51	3.19	57	4.7% : 3.5% (2/43 : 2/57)	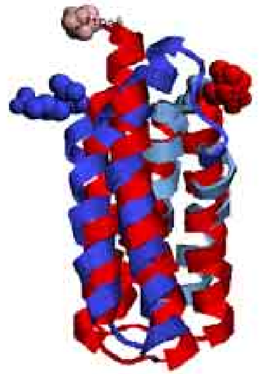
	1tqg:A (105)	Chemotaxis protein cheA								
232	1aue:A (92)	FKBP-RAPAMYCIN ASSOCIATED PROTEIN	**72**	36	0.002	0.30	3.37	72	2.7% : 8.3% (1/37 : 6/72)	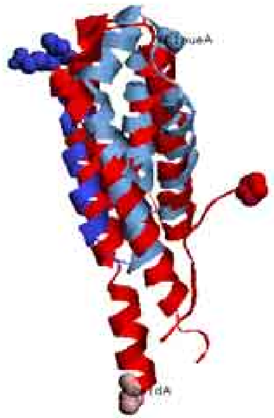
	2etd:A (145)	lemA protein								
233	* 2d80:A (318)	PHB depolymerase	**208**	34	0.019	0.43	2.64	163	13.4% : 14.7% (13/97 : 24/163)	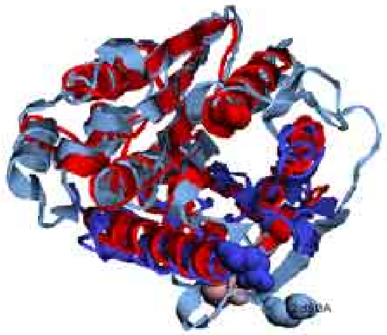
	* 1auo:A (218)	CARBOXYLESTERASE								

234	1dgl:A<br>(237)<br><br>1avb:A<br>(226)	LECTIN<br><br>ARCELIN-1	**123**	115	4E-27	0.80	1.47	206	37.6% : 37.4%<br>(38/101 : 77/206)	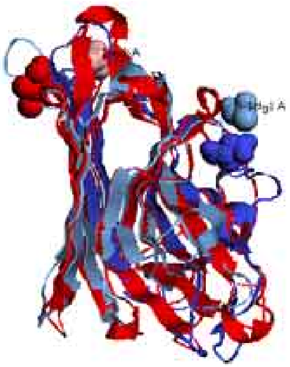
235	1mvq:A<br>(236)<br><br>1avb:A<br>(226)	lectin, isoform 1<br><br>ARCELIN-1	**123**	119	4E-28	0.80	1.49	206	37.6% : 39.3%<br>(38/101 : 81/206)	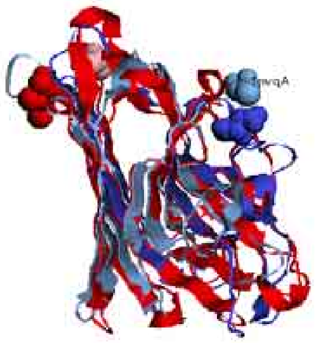
236	*<br>1avd:A<br>(123)<br><br>*<br>1swf:A<br>(117)	AVIDIN<br><br>CIRCULARLY PERMUTED CORE-STREPTAVIDIN E51/A46	**41**	44	1E-05	0.58	1.42	98	32.8% : 34.7%<br>(22/67 : 34/98)	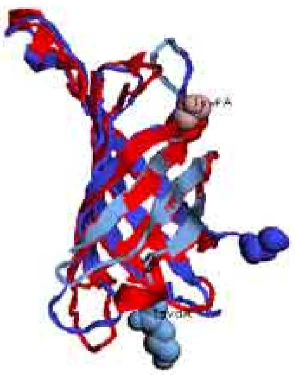

237	* 2c4i:A (245)		175	97	2E-21	0.34	0.58	116	97.9% : 98.3% (94/96 : 114/116)	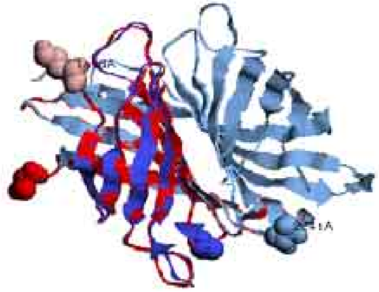
	* 1avd:A (123)	AVIDIN								
238	1avo:B (140)	11S REGULATOR	105	36	0.002	0.29	3.16	96	5.8% : 14.6% (5/86 : 14/96)	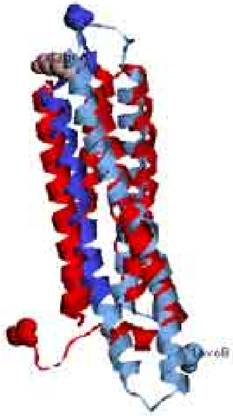
	1jgc:A (160)	bacterioferritin								
239	1qsd:A (102)	PROTEIN (BETA-TUBULIN BINDING POST-CHAPERONIN	44	48	7E-07	0.26	2.62	72	7.2% : 8.3% (5/69 : 6/72)	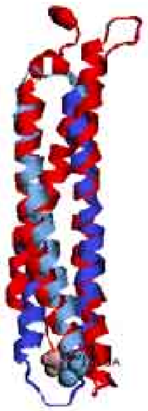
	1avo:B (140)	11S REGULATOR								

240	1xou:B (84)	Z5138 gene product	**49**	41	6E-05	0.38	3.47	74	12.3% : 9.5% (7/57 : 7/74)	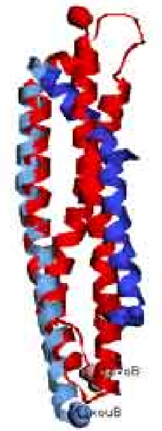
	1avo:B (140)	11S REGULATOR								
241	2fup:A (129)	hypothetical protein PA3352	**33**	46	2E-06	0.34	2.44	70	6.3% : 8.6% (4/64 : 6/70)	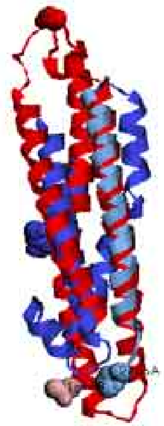
	1avo:B (140)	11S REGULATOR								
242	* 1awo:_ (62)	ABL TYROSINE KINASE	**47**	32	0.027	0.50	1.90	48	29.7% : 29.2% (11/37 : 14/48)	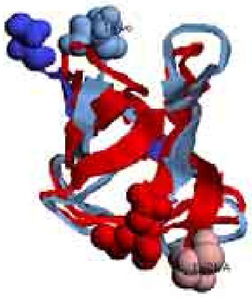
	* 1g2b:A (62)	SPECTRIN ALPHA CHAIN								

243	* 1awo:_ (57)	ABL TYROSINE KINASE	17	34	0.008	0.49	2.03	50	27.5% : 32.0% (11/40 : 16/50)	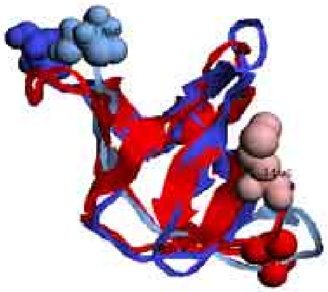
	* 1tuc_ (65)	ALPHA-SPECTRIN								
244	* 1aww:_ (67)	BRUTON'S TYROSINE KINASE	50	37	0.0007	0.57	1.62	54	33.3% : 31.5% (13/39 : 17/54)	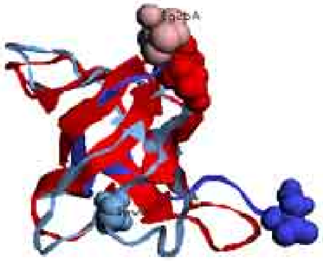
	* 1g2b:A (62)	SPECTRIN ALPHA CHAIN								
245	* 1aww:_ (67)	BRUTON'S TYROSINE KINASE	20	37	0.001	0.46	1.64	52	27.5% : 32.7% (11/40 : 17/52)	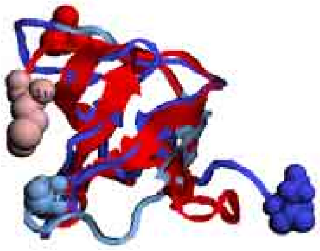
	* 1tuc_ (63)	ALPHA-SPECTRIN								

246	1dgl:A (237)	LECTIN	**123**	151	6E-38	0.91	1.42	225	42.5% : 40.9% (48/113 : 92/225)	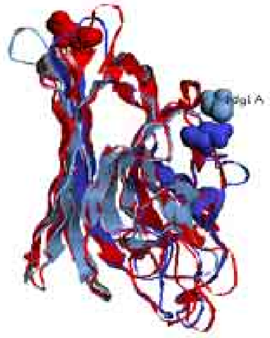
	1ax0_ (239)	LECTIN								
247	1mvq:A (236)	lectin, isoform 1	**123**	158	4E-40	0.89	1.29	222	43.8% : 43.2% (49/112 : 96/222)	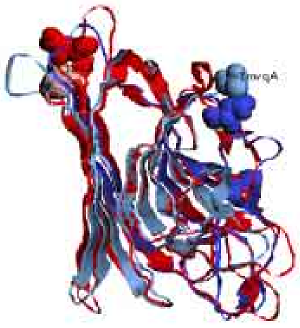
	1ax0_ (239)	LECTIN								
248	1ax8:_ (131)	OBESITY PROTEIN	**86**	32	0.03	0.26	3.28	79	9.1% : 8.9% (6/66 : 7/79)	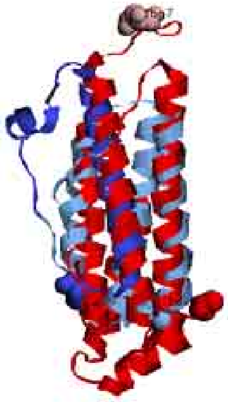
	1bg7_ (154)	FERRITIN								

249	1ax8:_ (131)	OBESITY PROTEIN	93	31	0.052	0.33	2.87	95	9.5% : 11.6% (7/74 : 11/95)	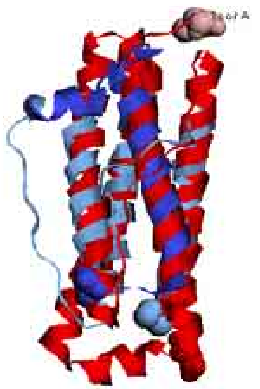
	1sof:A (155)	Bacterioferritin								
250	1ax8:_ (131)	OBESITY PROTEIN	97	33	0.018	0.28	2.59	65	6.8% : 7.7% (3/44 : 5/65)	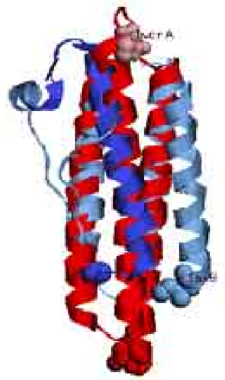
	1wcr:A (103)	PTS SYSTEM, N,N'-DIACETYLCHITOBIOSE-SPECIFIC								
251	1ax8:_ (131)	OBESITY PROTEIN	80	32	0.036	0.27	3.39	83	9.1% : 12.0% (5/55 : 10/83)	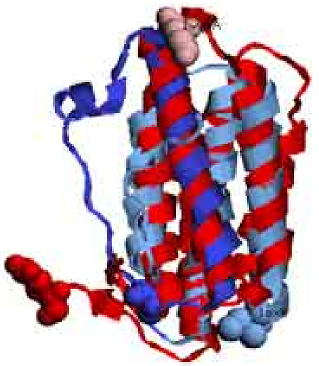
	1wvt:A (148)	hypothetical protein ST2180								

252	1ax8:_<br>(131)<br><br>1wy1:A<br>(142)	OBESITY PROTEIN<br><br>hypothetical protein PH0671	**103**	35	0.004	0.35	2.78	87	5.0% : 16.1%<br>(3/60 : 14/87)	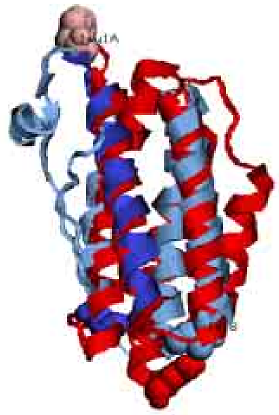
253	1ax8:_<br>(131)<br><br>2ah6:A<br>(152)	OBESITY PROTEIN<br><br>BH1595, unknown conserved protein	**30**	31	0.074	0.20	2.63	75	7.9% : 9.3%<br>(5/63 : 7/75)	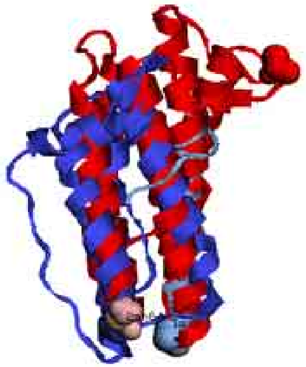
254	1ax8:_<br>(131)<br><br>2g2d:A<br>(160)	OBESITY PROTEIN<br><br>ATP:cobalamin adenosyltransferase	**104**	35	0.004	0.28	2.73	82	8.3% : 14.6%<br>(5/60 : 12/82)	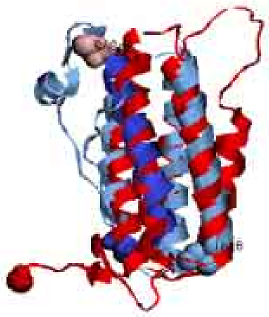

255	1ax8:_ (131)<br>2gs4:A (159)	OBESITY PROTEIN<br>Protein yciF	**97**	38	0.0003	0.30	2.11	86	10.9% : 11.6% (5/46 : 10/86)	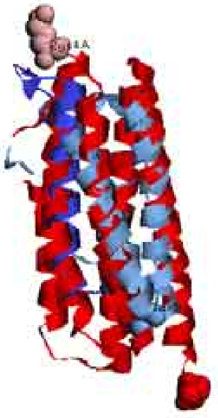
256	1ax8:_ (131)<br>2gyq:A (162)	OBESITY PROTEIN<br>ycfI, putative structural protein	**88**	38	0.0006	0.28	2.41	85	7.5% : 8.2% (6/80 : 7/85)	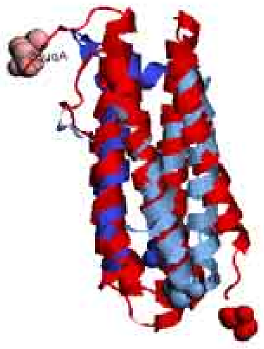
257	1ax8:_ (131)<br>2nt8:A (181)	OBESITY PROTEIN<br>Cobalamin adenosyltransferase	**82**	34	0.007	0.23	3.12	79	14.0% : 10.1% (6/43 : 8/79)	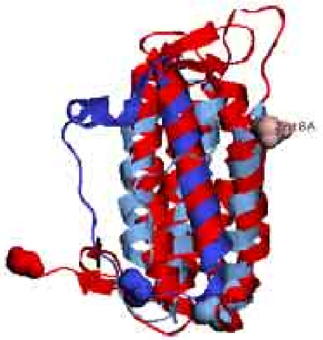

<table>
<tr><td rowspan="2">258</td><td>*
1byh:_
(214)</td><td>HYBRID</td><td rowspan="2">**59**</td><td rowspan="2">171</td><td rowspan="2">6E-44</td><td rowspan="2">0.60</td><td rowspan="2">0.74</td><td rowspan="2">205</td><td rowspan="2">100.0% : 96.1%
(156/156 : 197/205)</td><td rowspan="2">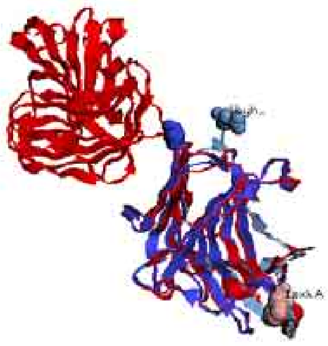</td></tr>
<tr><td>*
1axk:A
(393)</td><td>GLUXYN-1</td></tr>
<tr><td rowspan="2">259</td><td>*
1dyp:A
(271)</td><td>KAPPA-CARRAGEENASE</td><td rowspan="2">**84**</td><td rowspan="2">73</td><td rowspan="2">4E-14</td><td rowspan="2">0.29</td><td rowspan="2">1.60</td><td rowspan="2">166</td><td rowspan="2">23.6% : 23.5%
(30/127 : 39/166)</td><td rowspan="2">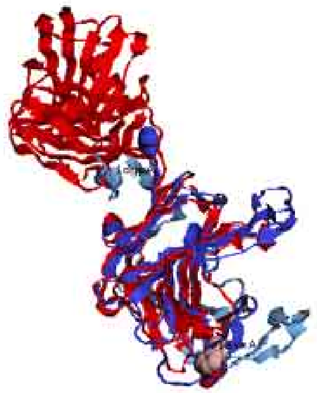</td></tr>
<tr><td>*
1axk:A
(393)</td><td>GLUXYN-1</td></tr>
<tr><td rowspan="2">260</td><td>*
1axk:A
(393)</td><td>GLUXYN-1</td><td rowspan="2">**327**</td><td rowspan="2">37</td><td rowspan="2">0.004</td><td rowspan="2">0.24</td><td rowspan="2">2.84</td><td rowspan="2">143</td><td rowspan="2">11.7% : 11.2%
(13/111 : 16/143)</td><td rowspan="2">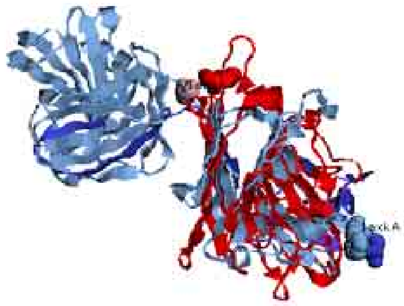</td></tr>
<tr><td>*
1dzq:A
(235)</td><td>ULEX EUROPAEUS LECTIN II (UEA-II)</td></tr>
</table>

261	* 1axk:A (393)	GLUXYN-1	**327**	39	0.001	0.21	2.87	141	8.1% : 10.6% (9/111 : 15/141)	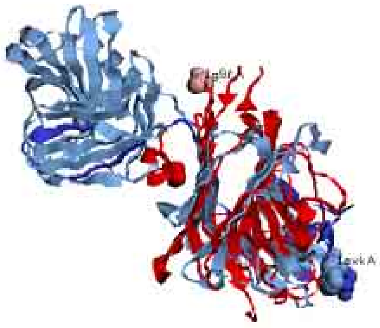
	* 1g9f:A (242)	LECTIN								
262	* 1gbg:_ (214)	(1,3-1,4)-BETA-D-GLUCAN 4 GLUCANOHYDROLASE	**59**	185	2E-48	0.60	0.53	205	78.8% : 74.1% (123/156 : 152/205)	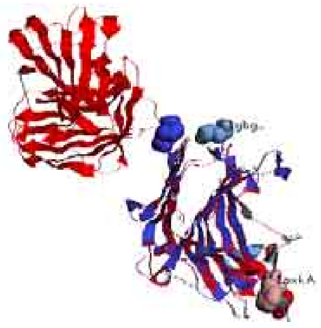
	* 1axk:A (393)	GLUXYN-1								
263	* 1o4y:A (270)	beta-agarase A	**86**	55	8E-09	0.29	1.98	175	21.7% : 20.6% (30/138 : 36/175)	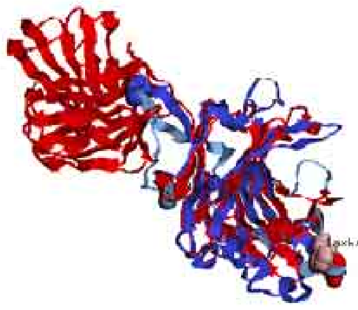
	* 1axk:A (393)	GLUXYN-1								

264	* 1axk:A (393)	GLUXYN-1	**315**	49	1E-06	0.24	1.93	174	23.3% : 22.4% (31/133 : 39/174)	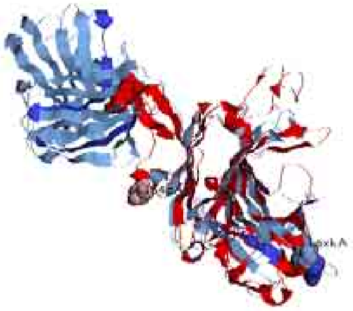
	* 1o4z:A (295)	beta-agarase B								
265	* 1axk:A (393)	GLUXYN-1	**320**	49	6E-07	0.23	3.15	133	7.6% : 11.3% (7/92 : 15/133)	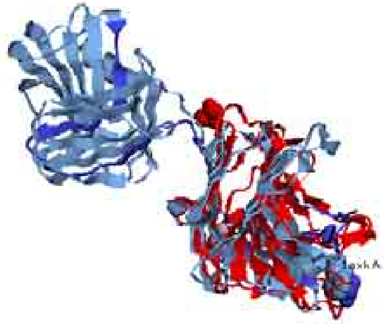
	* 1uai:A (223)	polyguluronate lyase								
266	* 1g2b:A (63)	SPECTRIN ALPHA CHAIN	**22**	36	0.002	0.62	1.49	51	31.6% : 31.4% (12/38 : 16/51)	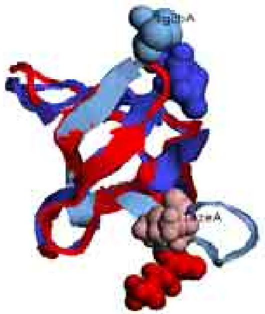
	* 1aze:A (56)	GRB2								

267	* 1tuc:_ (63)	ALPHA-SPECTRIN	**53**	38	0.0006	0.38	1.65	48	40.0% : 37.5% (16/40 : 18/48)	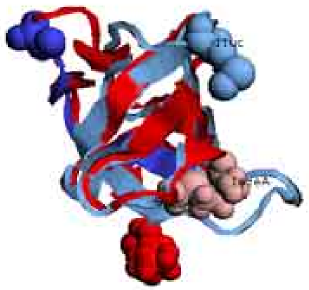
	* 1aze:A (56)	GRB2								
268	1hcf:X (101)	BDNF/NT-3 GROWTH FACTORS RECEPTOR	**44**	37	0.0008	0.22	3.11	51	8.8% : 9.8% (3/34 : 5/51)	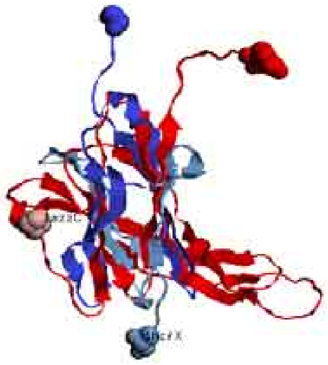
	1azz:C (137)	ECOTIN								
269	* 1g2b:A (63)	SPECTRIN ALPHA CHAIN	**19**	41	6E-05	0.72	1.32	55	32.5% : 32.7% (13/40 : 18/55)	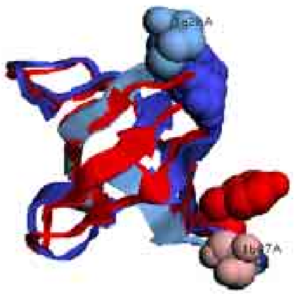
	* 1b07:A (58)	PROTEIN (PROTO-ONCOGENE CRK (CRK))								

270	* 1b07:A (58)	PROTEIN (PROTO-ONCOGENE CRK (CRK))	**15**	35	0.003	0.62	1.45	53	35.0% : 35.8% (14/40 : 19/53)	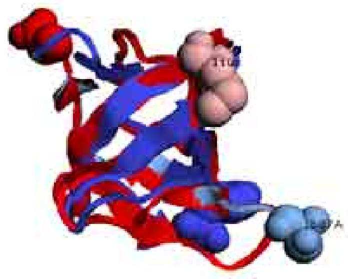
	* 1tuc_ (65)	ALPHA-SPECTRIN								
271	1b5l:_ (153)	INTERFERON TAU	**111**	36	0.002	0.32	3.16	102	7.1% : 4.9% (6/84 : 5/102)	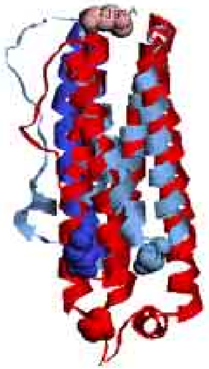
	1jgc:A (160)	bacterioferritin								
272	1ji4:A (144)	NEUTROPHIL-ACTIVATING PROTEIN A	**43**	37	0.0008	0.37	3.37	98	9.9% : 8.2% (7/71 : 8/98)	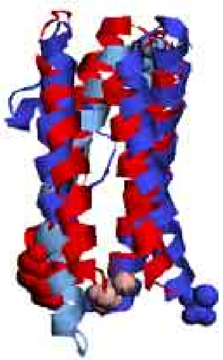
	1b5l_ (153)	INTERFERON TAU								

273	1b5l:_ (153)	INTERFERON TAU	**42**	35	0.004	0.37	3.16	105	7.4% : 10.5% (4/54 : 11/105)	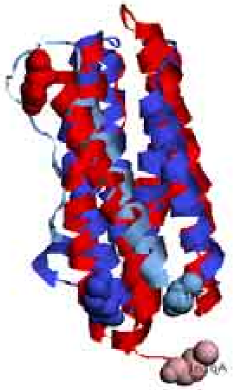
	1n1q:A (149)	DPS Protein								
274	1b5l:_ (153)	INTERFERON TAU	**48**	35	0.005	0.41	3.23	109	11.1% : 10.1% (6/54 : 11/109)	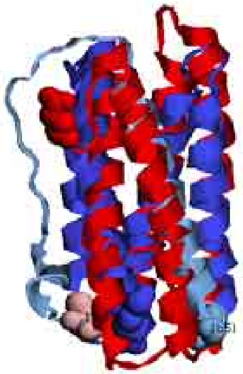
	1qgh:A (150)	NON-HEME IRON-CONTAINING FERRITIN								
275	1rcc:_ (171)	L FERRITIN	**49**	37	0.0009	0.31	3.47	104	4.4% : 10.6% (3/68 : 11/104)	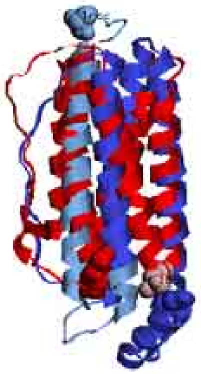
	1b5l_ (153)	INTERFERON TAU								

276	1b5l:_ (153)	INTERFERON TAU	**47**	35	0.003	0.33	2.65	98	12.3% : 7.1% (9/73 : 7/98)	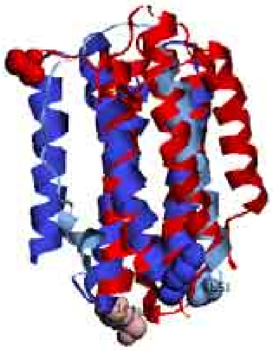
	1rty:A (159)	yvqk protein								
277	1b5l:_ (153)	INTERFERON TAU	**96**	39	0.0003	0.21	3.47	97	8.8% : 9.3% (7/80 : 9/97)	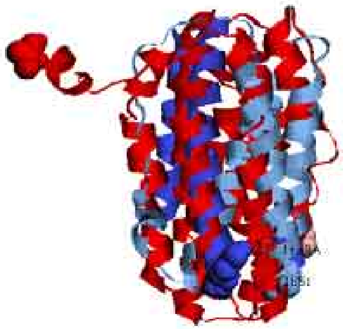
	1to9:A (225)	THI-4 protein								
278	1b5l:_ (153)	INTERFERON TAU	**122**	42	2E-05	0.23	2.77	128	4.5% : 6.3% (4/89 : 8/128)	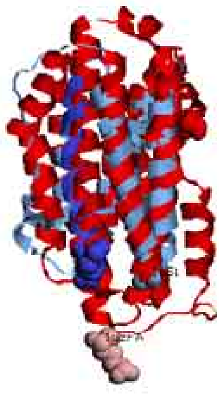
	1uzr:A (282)	RIBONUCLEOTIDE REDUCTASE R2-2 SMALL SUBUNIT								

279	1b5l:_ (153)	INTERFERON TAU	123	45	5E-06	0.36	2.85	103	5.8% : 11.7% (3/52 : 12/103)	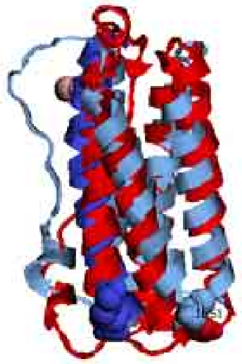
	1vjx:A (148)	putative ferritin-like diiron-carboxylate pro								
280	1b5l:_ (153)	INTERFERON TAU	50	40	0.0001	0.32	3.15	98	13.9% : 12.2% (10/72 : 12/98)	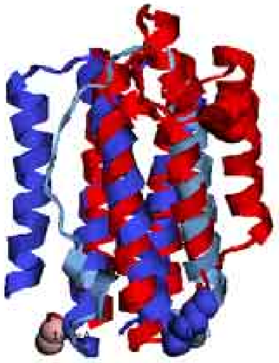
	1woz:A (159)	177aa long conserved hypothetical protein (ST								
281	1b5l:_ (153)	INTERFERON TAU	51	36	0.002	0.39	2.60	100	7.8% : 8.0% (5/64 : 8/100)	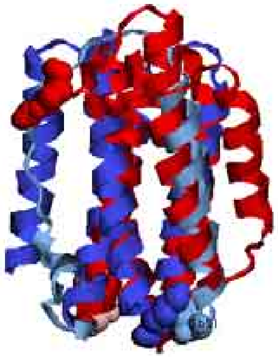
	1wvt:A (148)	hypothetical protein ST2180								

282	1b5l:_ (153)	INTERFERON TAU	108	36	0.002	0.43	3.37	118	5.2% : 11.0% (5/97 : 13/118)	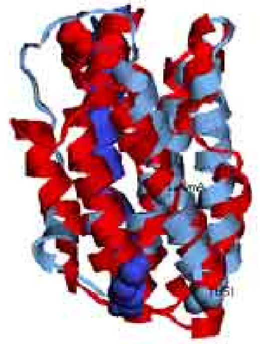
	1wwm:A (180)	hypothetical protein TT2028								
283	1b5l:_ (153)	INTERFERON TAU	32	38	0.0004	0.23	3.37	99	9.9% : 6.1% (8/81 : 6/99)	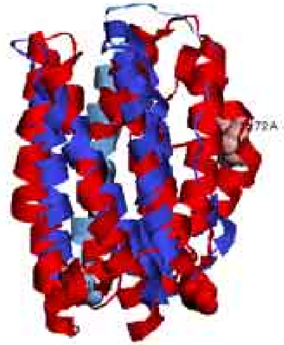
	1z72:A (217)	transcriptional regulator, putative								
284	1b5l:_ (153)	INTERFERON TAU	52	38	0.0004	0.35	2.52	95	8.8% : 8.4% (6/68 : 8/95)	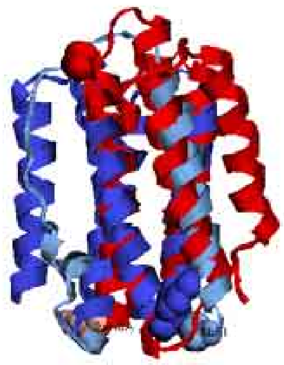
	2ah6:A (152)	BH1595, unknown conserved protein								

285	1b5l:_ (153)	INTERFERON TAU	110	37	0.001	0.33	3.04	100	6.7% : 12.0% (3/45 : 12/100)	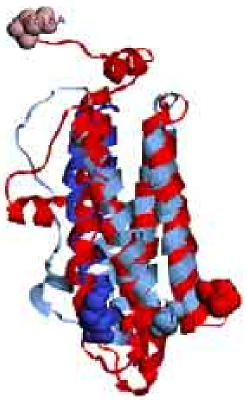
	2clb:A (169)	DPS-LIKE PROTEIN								
286	1b5l:_ (153)	INTERFERON TAU	44	35	0.004	0.36	2.75	107	7.9% : 12.1% (5/63 : 13/107)	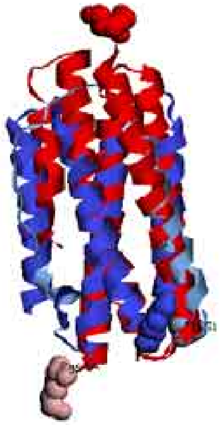
	2gs4:A (159)	Protein yciF								
287	1b5l:_ (153)	INTERFERON TAU	37	38	0.0004	0.32	3.42	115	5.2% : 13.9% (3/58 : 16/115)	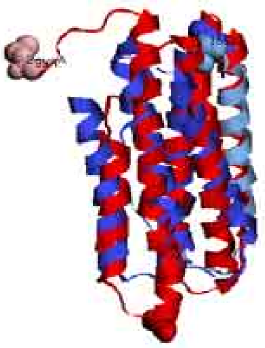
	2gyq:A (162)	ycfI, putative structural protein								

288	1b5l:_ (153)	INTERFERON TAU	**123**	35	0.004	0.42	2.78	108	7.8% : 6.5% (4/51 : 7/108)	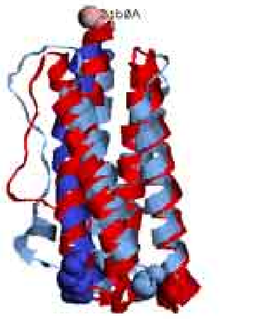
	2ib0:A (142)	CONSERVED HYPOTHETICAL ALANINE RICH PROTEIN								
289	1b5l:_ (153)	INTERFERON TAU	**101**	47	1E-06	0.33	3.48	115	3.6% : 7.0% (3/84 : 8/115)	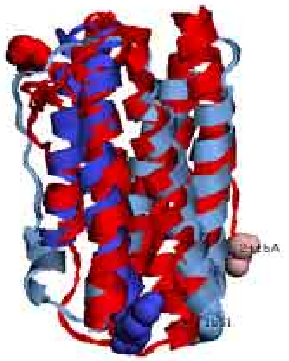
	2itb:A (199)	TRNA-(Ms(2)io(6)a)-hydroxylase, putative								
290	1b64:_ (91)	ELONGATION FACTOR 1-BETA	**35**	31	0.053	0.22	2.83	53	10.9% : 9.4% (5/46 : 5/53)	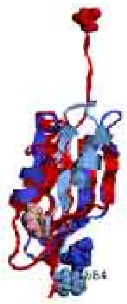
	1xn9:A (101)	30S ribosomal protein S24e								

291	1b64:_ (91)	ELONGATION FACTOR 1-BETA	**35**	31	0.053	0.22	2.54	51	13.6% : 13.7% (6/44 : 7/51)	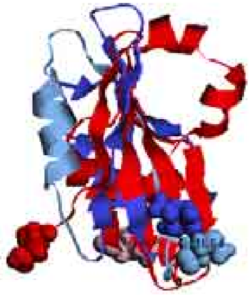
	1ywx:A (102)	30S ribosomal protein S24e								
292	1bgd:_ (158)	GRANULOCYTE COLONY-STIMULATING FACTOR	**130**	35	0.005	0.26	2.83	81	16.0% : 8.6% (8/50 : 7/81)	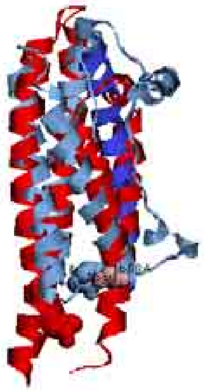
	1b68:A (139)	APOLIPOPROTEIN E								
293	1n1q:A (149)	DPS Protein	**92**	45	3E-06	0.28	2.67	83	13.8% : 6.0% (8/58 : 5/83)	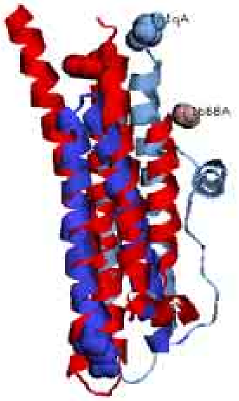
	1b68:A (139)	APOLIPOPROTEIN E								

294	1b68:A (139)	APOLIPOPROTEIN E	**33**	47	1E-06	0.27	3.03	93	4.3% : 6.5% (3/69 : 6/93)	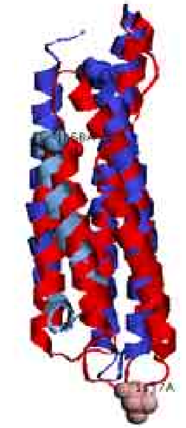
	1sj7:A (167)	Talin 1								
295	1x8z:A (151)	invertase/pectin methylesterase inhibitor fam	**64**	37	0.0007	0.34	2.92	91	11.1% : 11.0% (9/81 : 10/91)	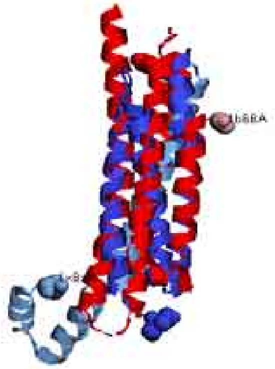
	1b68:A (139)	APOLIPOPROTEIN E								
296	1b68:A (139)	APOLIPOPROTEIN E	**111**	41	5E-05	0.52	3.49	101	14.6% : 7.9% (12/82 : 8/101)	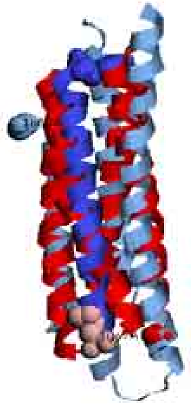
	1yo7:A (120)	Regulatory protein rop								

297	2gsc:A (114)	conserved hypothetical protein	**90**	44	1E-05	0.36	3.29	92	9.3% : 9.8% (5/54 : 9/92)	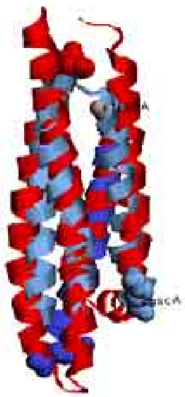
	1b68:A (139)	APOLIPOPROTEIN E								
298	2gyq:A (162)	ycfI, putative structural protein	**137**	42	3E-05	0.22	2.46	82	9.3% : 6.1% (5/54 : 5/82)	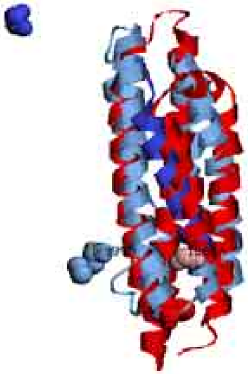
	1b68:A (139)	APOLIPOPROTEIN E								
299	2ieq:A (89)	Spike glycoprotein	**35**	45	4E-06	0.25	2.37	61	6.3% : 8.2% (3/48 : 5/61)	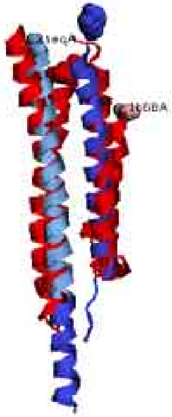
	1b68:A (139)	APOLIPOPROTEIN E								

300	1bgc:_ (159)	GRANULOCYTE COLONY-STIMULATING FACTOR	51	40	0.0001	0.30	2.69	112	5.1% : 8.0% (3/59 : 9/112)	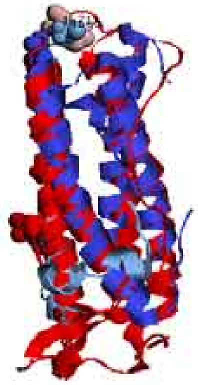
	1b71:A (191)	PROTEIN (RUBRERYTHRIN)								
301	1cd9:A (171)	PROTEIN (GRANULOCYTE COLONY-STIMULATING FACTO	53	36	0.003	0.22	2.89	96	8.6% : 8.3% (5/58 : 8/96)	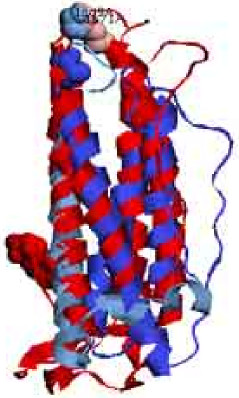
	1b71:A (191)	PROTEIN (RUBRERYTHRIN)								
302	1cnt:1 (152)	CILIARY NEUROTROPHIC FACTOR	33	41	7E-05	0.29	2.47	113	5.5% : 3.5% (3/55 : 4/113)	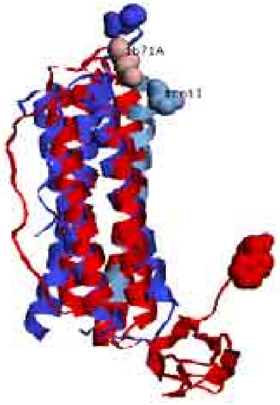
	1b71:A (191)	PROTEIN (RUBRERYTHRIN)								

303	1i1r:B (167)	VIRAL IL-6	**53**	33	0.015	0.22	2.96	99	11.3% : 4.0% (6/53 : 4/99)	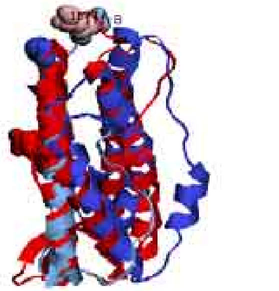
	1b71:A (191)	PROTEIN (RUBRERYTHRIN)								
304	1bbh:A (131)	CYTOCHROME C'	**96**	34	0.009	0.23	3.35	70	9.4% : 12.9% (5/53 : 9/70)	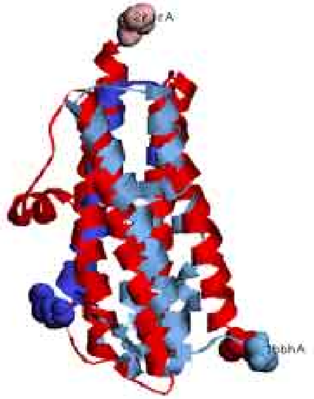
	2fjc:A (151)	Antigen TpF1								
305	2g2d:A (160)	ATP:cobalamin adenosyltransferase	**93**	36	0.003	0.27	2.94	86	9.3% : 9.3% (4/43 : 8/86)	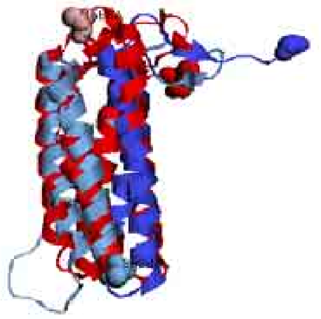
	1bbh:A (131)	CYTOCHROME C'								

306	1bgc:_ (159)	GRANULOCYTE COLONY-STIMULATING FACTOR	**52**	50	1E-07	0.42	2.50	117	6.0% : 12.0% (5/83 : 14/117)	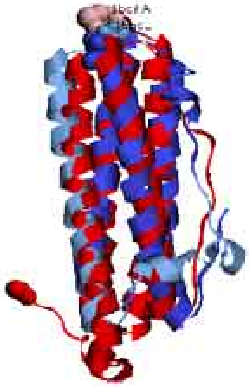
	1bcf:A (157)	BACTERIOFERRITIN								
307	1bgd:_ (158)	GRANULOCYTE COLONY-STIMULATING FACTOR	**51**	45	5E-06	0.45	2.42	120	11.1% : 10.0% (7/63 : 12/120)	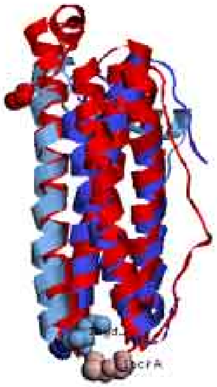
	1bcf:A (157)	BACTERIOFERRITIN								
308	1cd9:A (171)	PROTEIN (GRANULOCYTE COLONY-STIMULATING FACTO	**55**	52	5E-08	0.39	2.74	119	8.9% : 10.9% (8/90 : 13/119)	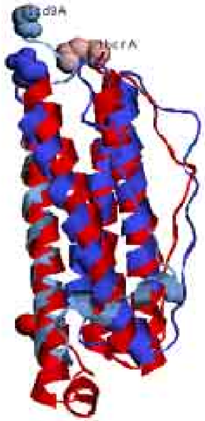
	1bcf:A (157)	BACTERIOFERRITIN								

309	1bcf:A (157)	BACTERIOFERRITIN	122	46	2E-06	0.34	2.88	116	9.8% : 8.6% (9/92 : 10/116)	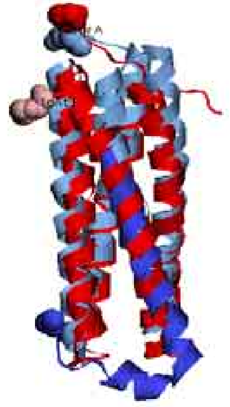
	1cnt:1 (152)	CILIARY NEUROTROPHIC FACTOR								
310	1emr:A (159)	LEUKEMIA INHIBITORY FACTOR	51	37	0.001	0.38	3.42	119	5.6% : 9.2% (5/89 : 11/119)	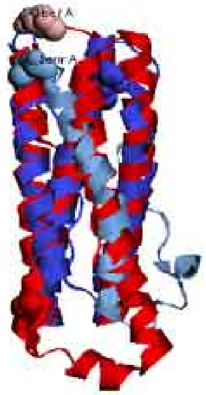
	1bcf:A (157)	BACTERIOFERRITIN								
311	1f1m:A (162)	OUTER SURFACE PROTEIN C	128	36	0.002	0.22	1.81	82	3.1% : 8.5% (2/65 : 7/82)	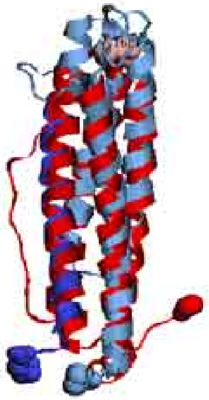
	1bcf:A (157)	BACTERIOFERRITIN								

312	1f45:B (137)	INTERLEUKIN-12 ALPHA CHAIN	103	36	0.002	0.31	2.77	93	5.3% : 7.5% (3/57 : 7/93)	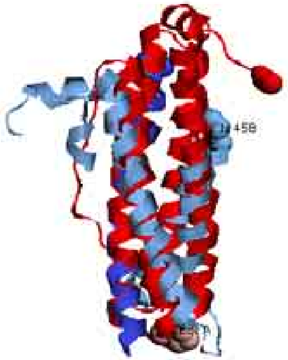
	1bcf:A (157)	BACTERIOFERRITIN								
313	1bcf:A (157)	BACTERIOFERRITIN	100	40	0.0002	0.30	2.75	110	8.8% : 4.5% (7/80 : 5/110)	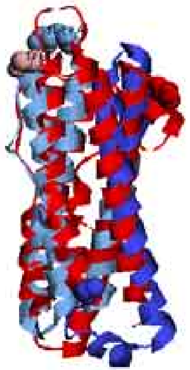
	1f6f:A (185)	PLACENTAL LACTOGEN								
314	1ggq:A (162)	OUTER SURFACE PROTEIN C	129	34	0.008	0.22	1.93	82	10.5% : 7.3% (6/57 : 6/82)	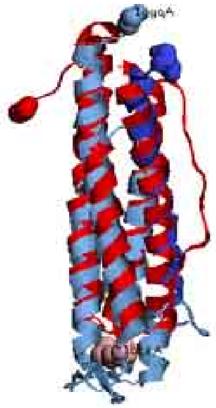
	1bcf:A (157)	BACTERIOFERRITIN								

315	1i1r:B (167)	VIRAL IL-6	**130**	33	0.015	0.34	3.42	108	0.0% : 6.5% (0/49 : 7/108)	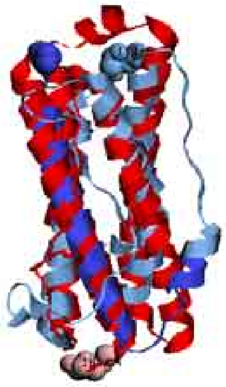
	1bcf:A (157)	BACTERIOFERRITIN								
316	1lvf:A (106)	syntaxin 6	**41**	44	6E-06	0.36	2.91	85	6.0% : 3.5% (5/84 : 3/85)	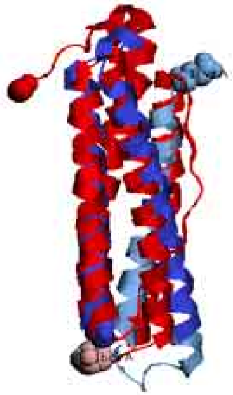
	1bcf:A (157)	BACTERIOFERRITIN								
317	1yjg:A (157)	Surface protein VspA	**122**	47	1E-06	0.24	1.92	83	3.9% : 8.4% (3/77 : 7/83)	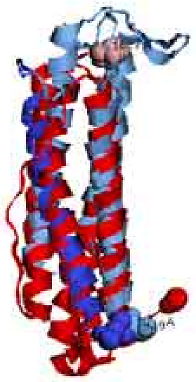
	1bcf:A (157)	BACTERIOFERRITIN								

318	1z7c:A (165)	Chorionic somatomammotropin hormone	**48**	32	0.052	0.38	3.49	111	8.6% : 8.1% (6/70 : 9/111)	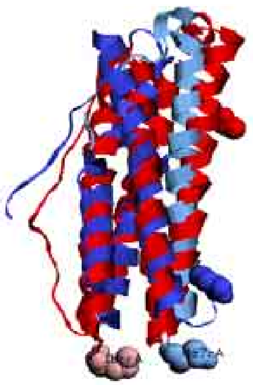
	1bcf:A (157)	BACTERIOFERRITIN								
319	1bcf:A (157)	BACTERIOFERRITIN	**87**	35	0.004	0.39	2.03	96	8.8% : 10.4% (8/91 : 10/96)	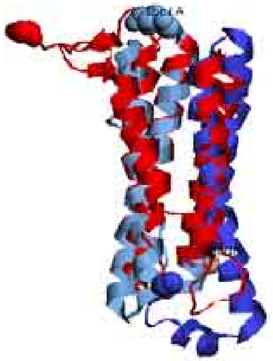
	2g2d:A (160)	ATP:cobalamin adenosyltransferase								
320	2huj:A (125)	Lin2004 protein	**90**	45	3E-06	0.29	3.20	77	5.7% : 2.6% (3/53 : 2/77)	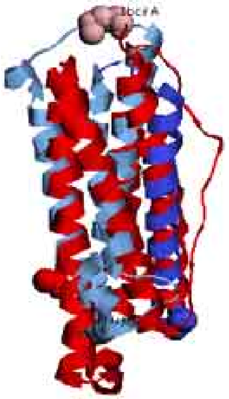
	1bcf:A (157)	BACTERIOFERRITIN								

321	* 1bed:_ (181)	DSBA OXIDOREDUCTASE	**98**	97	1E-21	0.88	1.95	172	43.3% : 39.0% (39/90 : 67/172)	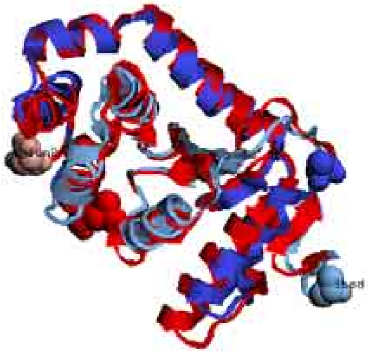
	* 1un2:A (186)	THIOL-DISULFIDE INTERCHANGE PROTEIN								
322	1bg7:_ (154)	FERRITIN	**41**	40	0.0002	0.33	2.29	100	3.9% : 7.0% (2/51 : 7/100)	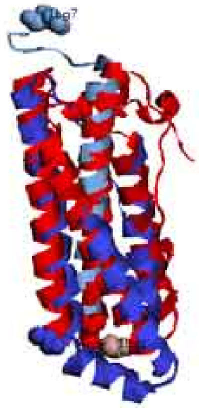
	1bgc_ (159)	GRANULOCYTE COLONY-STIMULATING FACTOR								
323	1bg7:_ (154)	FERRITIN	**84**	37	0.0008	0.29	2.68	82	6.6% : 8.5% (4/61 : 7/82)	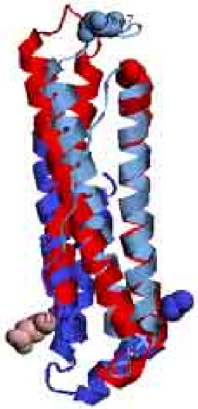
	1lvf:A (106)	syntaxin 6								

324	1bg7:_ (154)	FERRITIN	**81**	38	0.0006	0.31	1.95	59	7.7% : 15.3% (4/52 : 9/59)	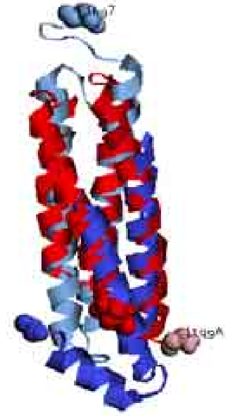
	1tqg:A (105)	Chemotaxis protein cheA								
325	1bgc:_ (159)	GRANULOCYTE COLONY-STIMULATING FACTOR	**113**	51	6E-08	0.39	2.95	115	9.3% : 9.6% (5/54 : 11/115)	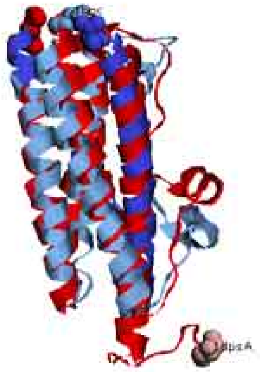
	1dps:A (159)	DPS								
326	1bgc:_ (159)	GRANULOCYTE COLONY-STIMULATING FACTOR	**55**	53	1E-08	0.41	3.11	115	5.5% : 11.3% (4/73 : 13/115)	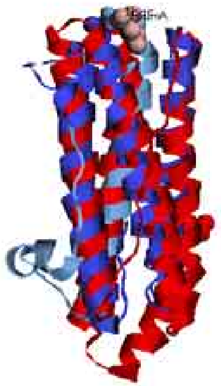
	1eum:A (161)	FERRITIN 1								

327	1bgc:_ (159)	GRANULOCYTE COLONY-STIMULATING FACTOR	**49**	49	2E-07	0.36	2.86	114	8.3% : 7.0% (5/60 : 8/114)	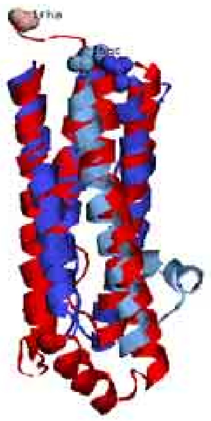
	1fha_ (173)	FERRITIN								
328	1bgc:_ (159)	GRANULOCYTE COLONY-STIMULATING FACTOR	**47**	55	6E-09	0.36	2.76	112	11.1% : 9.8% (9/81 : 11/112)	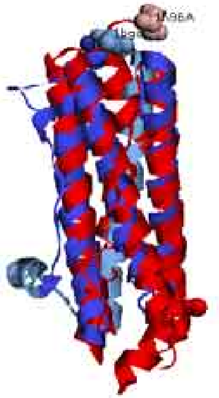
	1h96:A (167)	FERRITIN LIGHT CHAIN 1								
329	1bgc:_ (159)	GRANULOCYTE COLONY-STIMULATING FACTOR	**69**	40	0.0001	0.27	3.13	74	13.0% : 5.4% (7/54 : 4/74)	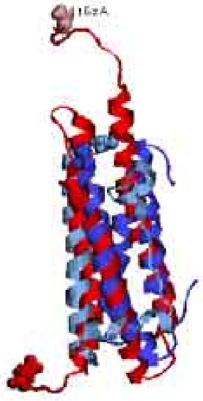
	1i6z:A (135)	BAG-FAMILY MOLECULAR CHAPERONE REGULATOR-1								

330	1bgc:_ (159)	GRANULOCYTE COLONY-STIMULATING FACTOR	**51**	52	2E-08	0.41	3.44	116	12.9% : 6.0% (11/85 : 7/116)	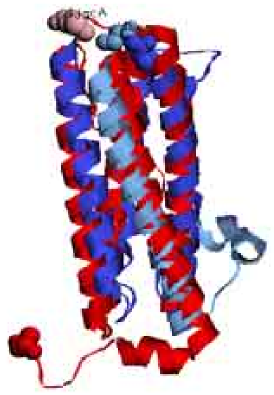
	1jgc:A (160)	bacterioferritin								
331	1bgc:_ (159)	GRANULOCYTE COLONY-STIMULATING FACTOR	**132**	43	1E-05	0.31	2.37	99	3.6% : 12.1% (2/56 : 12/99)	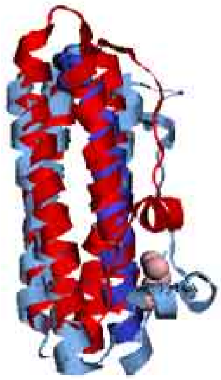
	1ji4:A (144)	NEUTROPHIL-ACTIVATING PROTEIN A								
332	1jig:A (146)	Dlp-2	**33**	39	0.0002	0.34	2.79	98	13.8% : 13.3% (12/87 : 13/98)	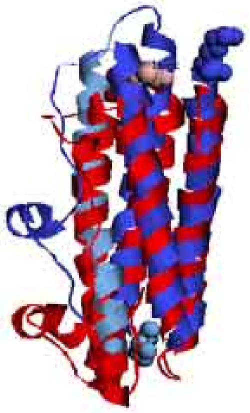
	1bgc_ (159)	GRANULOCYTE COLONY-STIMULATING FACTOR								

333	1bgc:_ (159)	GRANULOCYTE COLONY-STIMULATING FACTOR	**48**	46	3E-06	0.25	2.96	84	5.4% : 4.8% (3/56 : 4/84)	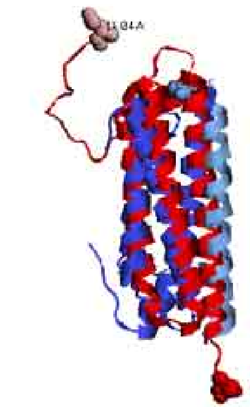
	1k04:A (142)	FOCAL ADHESION KINASE 1								
334	1bgc:_ (159)	GRANULOCYTE COLONY-STIMULATING FACTOR	**54**	52	4E-08	0.37	3.12	113	9.1% : 9.7% (8/88 : 11/113)	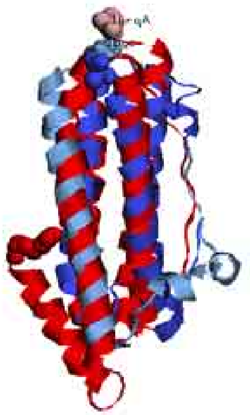
	1krq:A (164)	ferritin								
335	1bgc:_ (159)	GRANULOCYTE COLONY-STIMULATING FACTOR	**88**	39	0.0003	0.33	1.80	62	7.3% : 8.1% (4/55 : 5/62)	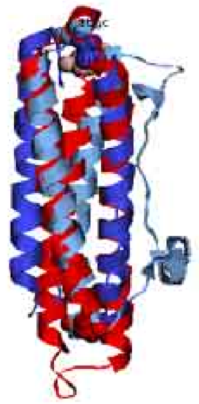
	1lvf:A (106)	syntaxin 6								

336	1bgc:_ (159)	GRANULOCYTE COLONY-STIMULATING FACTOR	**46**	51	5E-08	0.36	3.35	115	3.6% : 6.1% (2/56 : 7/115)	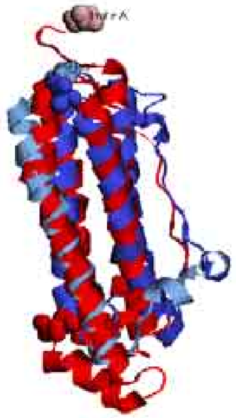
	1mfr:A (171)	M FERRITIN								
337	1bgc:_ (159)	GRANULOCYTE COLONY-STIMULATING FACTOR	**106**	52	4E-08	0.25	3.10	100	13.6% : 13.0% (8/59 : 13/100)	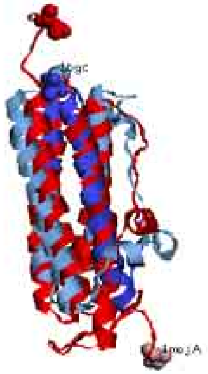
	1moj:A (180)	Dps-like ferritin								
338	1n1q:A (149)	DPS Protein	**36**	44	1E-05	0.37	2.91	101	11.6% : 8.9% (10/86 : 9/101)	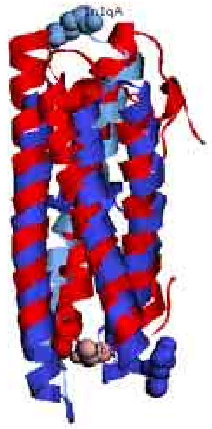
	1bgc_ (159)	GRANULOCYTE COLONY-STIMULATING FACTOR								

339	1bgc:_ (159)	GRANULOCYTE COLONY-STIMULATING FACTOR	**49**	53	2E-08	0.35	2.35	111	0.0% : 10.8% (0/51 : 12/111)	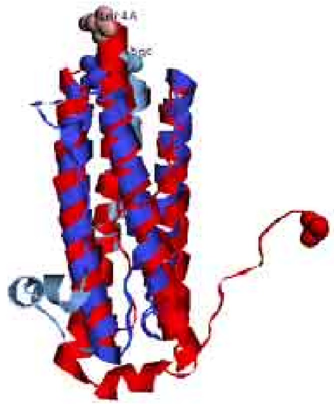
	1nf4:A (169)	bacterioferritin								
340	1bgc:_ (159)	GRANULOCYTE COLONY-STIMULATING FACTOR	**37**	46	3E-06	0.36	2.55	86	12.7% : 3.5% (8/63 : 3/86)	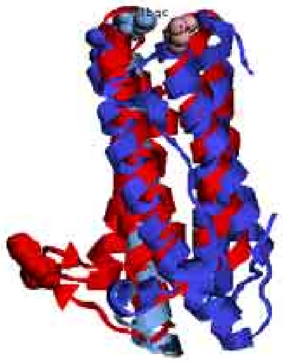
	1nog:A (149)	conserved hypothetical protein TA0546								
341	1o9r:A (162)	AGROBACTERIUM TUMEFACIENS DPS	**45**	53	2E-08	0.38	2.58	116	3.5% : 6.9% (2/57 : 8/116)	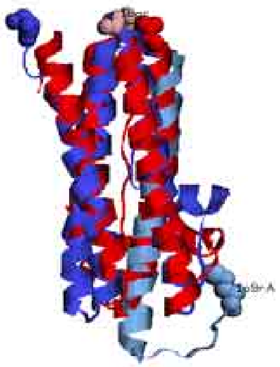
	1bgc_ (159)	GRANULOCYTE COLONY-STIMULATING FACTOR								

342	1bgc:_ (159)	GRANULOCYTE COLONY-STIMULATING FACTOR	122	34	0.007	0.34	1.74	60	16.4% : 6.7% (9/55 : 4/60)	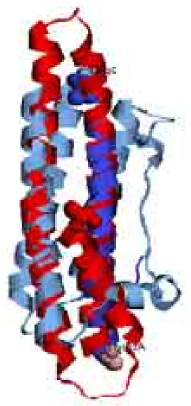
	1qsd:A (102)	PROTEIN (BETA-TUBULIN BINDING POST-CHAPERONIN								
343	1r03:A (170)	mitochondrial ferritin	122	45	5E-06	0.30	2.83	103	8.5% : 7.8% (5/59 : 8/103)	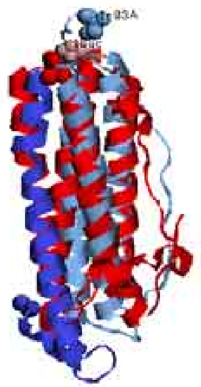
	1bgc_ (159)	GRANULOCYTE COLONY-STIMULATING FACTOR								
344	1bgc:_ (159)	GRANULOCYTE COLONY-STIMULATING FACTOR	47	53	2E-08	0.37	3.34	115	8.1% : 7.0% (7/86 : 8/115)	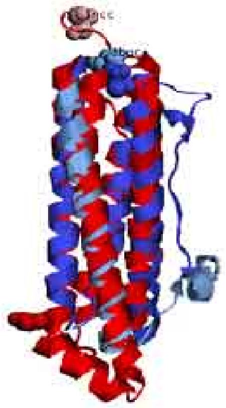
	1rcc_ (171)	L FERRITIN								

345	1bgc:_ (159)	GRANULOCYTE COLONY-STIMULATING FACTOR	**58**	49	4E-07	0.28	3.45	107	12.7% : 8.4% (10/79 : 9/107)	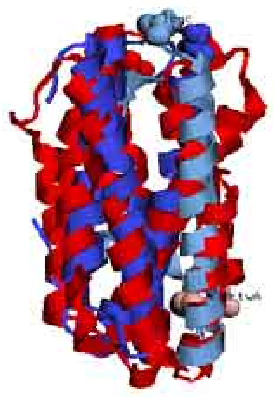
	1rtw:A (206)	transcriptional activator, putative								
346	1rty:A (159)	yvqk protein	**34**	35	0.006	0.31	3.07	97	7.6% : 15.5% (5/66 : 15/97)	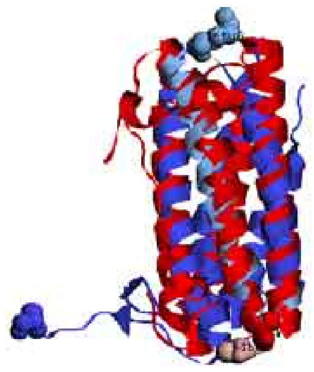
	1bgc_ (159)	GRANULOCYTE COLONY-STIMULATING FACTOR								
347	1bgc:_ (159)	GRANULOCYTE COLONY-STIMULATING FACTOR	**54**	57	8E-10	0.35	3.34	102	9.0% : 10.8% (7/78 : 11/102)	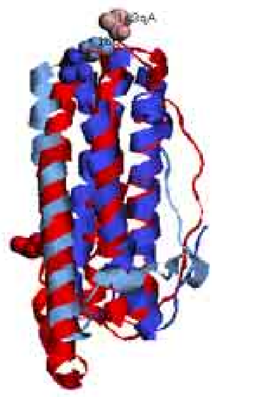
	1s3q:A (162)	ferritin								

348	1sof:A (155)	Bacterioferritin	113	48	7E-07	0.42	3.41	117	9.4% : 10.3% (8/85 : 12/117)	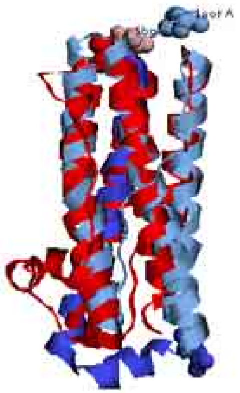
	1bgc_ (159)	GRANULOCYTE COLONY-STIMULATING FACTOR								
349	1umn:A (151)	DPS-LIKE PEROXIDE RESISTANCE PROTEIN	31	47	1E-06	0.34	2.73	102	11.6% : 13.7% (10/86 : 14/102)	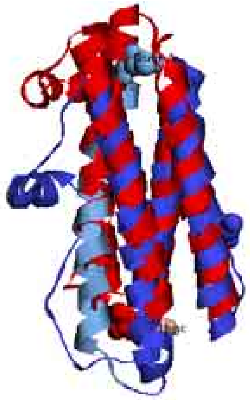
	1bgc_ (159)	GRANULOCYTE COLONY-STIMULATING FACTOR								
350	1bgc:_ (159)	GRANULOCYTE COLONY-STIMULATING FACTOR	55	48	7E-07	0.21	2.40	80	9.8% : 15.0% (5/51 : 12/80)	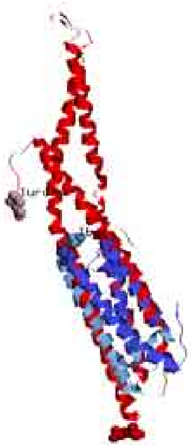
	1uru:A (217)	AMPHIPHYSIN								

351	1bgc:_ (159)	GRANULOCYTE COLONY-STIMULATING FACTOR	59	41	8E-05	0.46	2.25	110	9.0% : 9.1% (8/89 : 10/110)	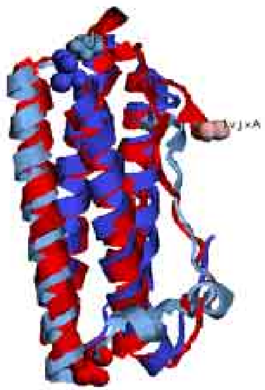
	1vjx:A (148)	putative ferritin-like diiron-carboxylate pro								
352	1bgc:_ (159)	GRANULOCYTE COLONY-STIMULATING FACTOR	52	56	2E-09	0.38	3.08	114	8.3% : 8.8% (7/84 : 10/114)	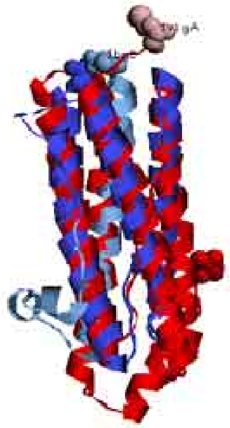
	1vlg:A (164)	ferritin								
353	1bgc:_ (159)	GRANULOCYTE COLONY-STIMULATING FACTOR	34	44	6E-06	0.30	2.82	93	13.6% : 9.7% (9/66 : 9/93)	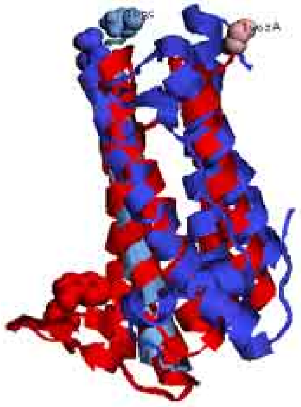
	1woz:A (159)	177aa long conserved hypothetical protein (ST								

354	1bgc:_<br>(159)<br>1wy1:A<br>(142)	GRANULOCYTE COLONY-STIMULATING FACTOR<br>hypothetical protein PH0671	**35**	37	0.001	0.37	2.36	95	9.4% : 9.5%<br>(6/64 : 9/95)	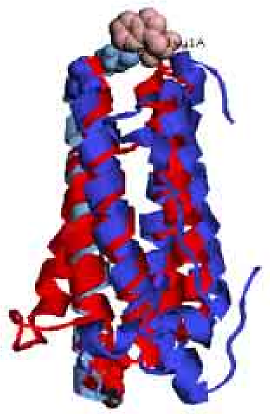
355	1bgc:_<br>(159)<br>1yo7:A<br>(120)	GRANULOCYTE COLONY-STIMULATING FACTOR<br>Regulatory protein rop	**59**	34	0.007	0.36	1.90	77	9.6% : 7.8%<br>(7/73 : 6/77)	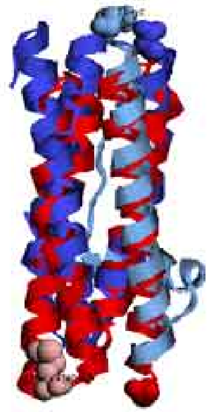
356	1bgc:_<br>(159)<br>1zs3:A<br>(171)	GRANULOCYTE COLONY-STIMULATING FACTOR<br>Lactococcus lactis MG1363 DpsA	**112**	38	0.0005	0.31	2.87	114	11.4% : 8.8%<br>(10/88 : 10/114)	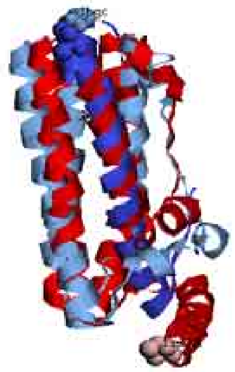

357	1bgc:_ (159)	GRANULOCYTE COLONY-STIMULATING FACTOR	**138**	38	0.0005	0.22	2.85	83	7.5% : 9.6% (5/67 : 8/83)	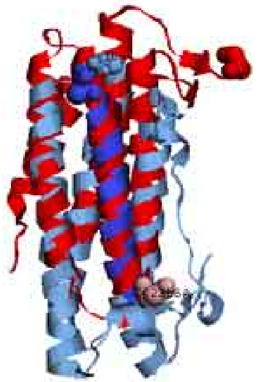
	2ah6:A (152)	BH1595, unknown conserved protein								
358	2c2f:A (178)	DNA-BINDING STRESS RESPONSE PROTEIN	**141**	34	0.01	0.30	2.48	106	13.8% : 12.3% (8/58 : 13/106)	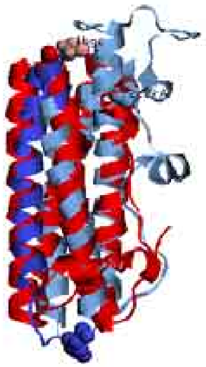
	1bgc_ (159)	GRANULOCYTE COLONY-STIMULATING FACTOR								
359	2c41:A (155)	DPS FAMILY DNA-BINDING STRESS RESPONSE PROTEI	**34**	44	7E-06	0.45	3.05	118	7.0% : 12.7% (4/57 : 15/118)	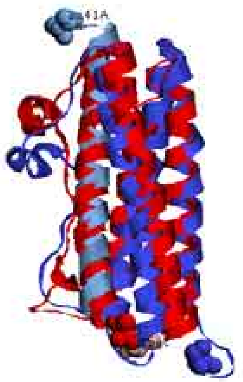
	1bgc_ (159)	GRANULOCYTE COLONY-STIMULATING FACTOR								

360	2clb:A (169)	DPS-LIKE PROTEIN	**48**	42	3E-05	0.34	2.77	117	11.1% : 6.8% (9/81 : 8/117)	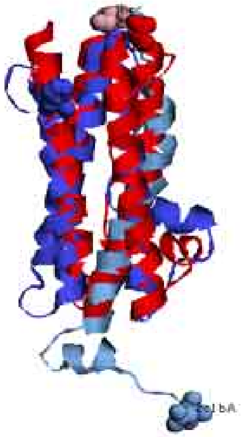
	1bgc_ (159)	GRANULOCYTE COLONY-STIMULATING FACTOR								
361	2d5k:A (148)	Dps family protein	**33**	40	0.0001	0.45	2.65	115	6.6% : 12.2% (6/91 : 14/115)	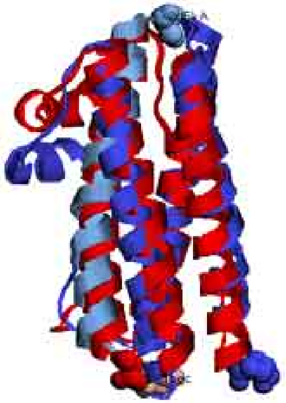
	1bgc_ (159)	GRANULOCYTE COLONY-STIMULATING FACTOR								
362	1bgc:_ (159)	GRANULOCYTE COLONY-STIMULATING FACTOR	**100**	38	0.0007	0.20	3.33	99	10.3% : 9.1% (9/87 : 9/99)	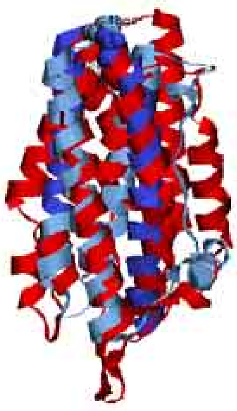
	2f2g:A (215)	SEED MATURATION PROTEIN PM36 HOMOLOG								

363	2ffx:J (173)	ferritin light chain	**123**	50	1E-07	0.33	3.29	111	1.8% : 10.8% (1/57 : 12/111)	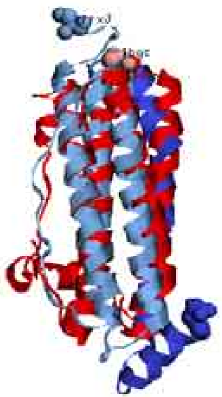
	1bgc_ (159)	GRANULOCYTE COLONY-STIMULATING FACTOR								
364	2fjc:A (151)	Antigen TpF1	**36**	40	0.0001	0.38	2.98	102	7.8% : 15.7% (7/90 : 16/102)	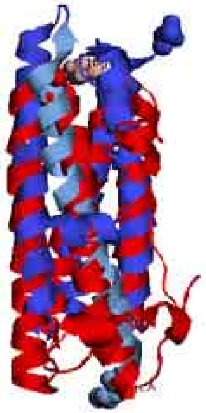
	1bgc_ (159)	GRANULOCYTE COLONY-STIMULATING FACTOR								
365	1bgc:_ (159)	GRANULOCYTE COLONY-STIMULATING FACTOR	**51**	43	2E-05	0.45	2.30	121	6.7% : 5.8% (6/89 : 7/121)	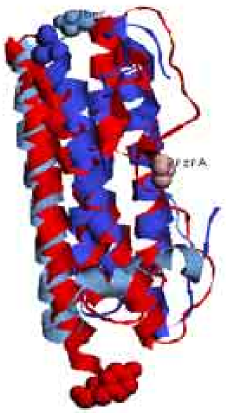
	2fzf:A (158)	hypothetical protein								

366	1bgc:_ (159)	GRANULOCYTE COLONY-STIMULATING FACTOR	35	36	0.002	0.31	2.34	95	13.3% : 9.5% (8/60 : 9/95)	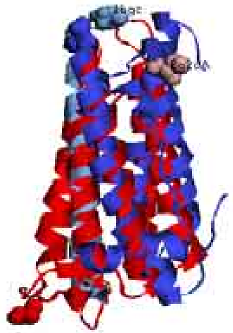
	2g2d:A (160)	ATP:cobalamin adenosyltransferase								
367	1bgc:_ (159)	GRANULOCYTE COLONY-STIMULATING FACTOR	127	42	4E-05	0.43	2.48	115	7.7% : 6.1% (5/65 : 7/115)	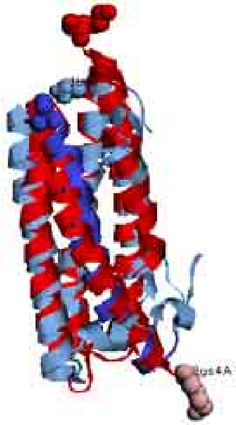
	2gs4:A (159)	Protein yciF								
368	1bgc:_ (159)	GRANULOCYTE COLONY-STIMULATING FACTOR	120	41	5E-05	0.43	2.58	119	8.5% : 8.4% (4/47 : 10/119)	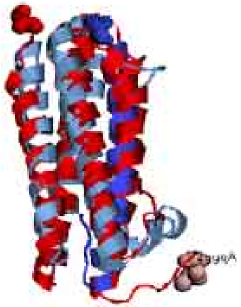
	2gyq:A (162)	ycfI, putative structural protein								

369	1bgc:_<br>(159)	GRANULOCYTE COLONY-STIMULATING FACTOR	**58**	43	2E-05	0.47	2.30	113	7.1% : 7.1%<br>(6/84 : 8/113)	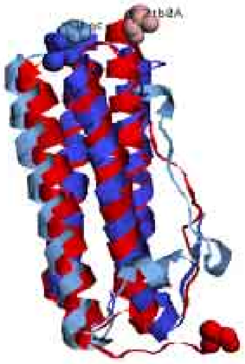
	2ib0:A<br>(142)	CONSERVED HYPOTHETICAL ALANINE RICH PROTEIN								
370	1bgc:_<br>(159)	GRANULOCYTE COLONY-STIMULATING FACTOR	**37**	53	2E-08	0.25	2.79	103	12.0% : 10.7%<br>(9/75 : 11/103)	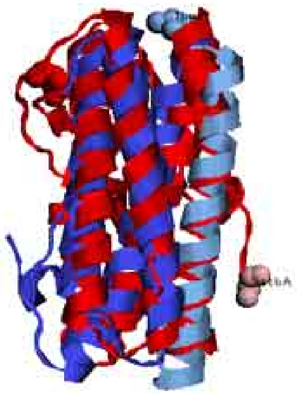
	2itb:A<br>(199)	TRNA-(Ms(2)io(6)a)-hydroxylase, putative								
371	1dps:A<br>(159)	DPS	**49**	46	2E-06	0.36	2.92	115	8.6% : 9.6%<br>(8/93 : 11/115)	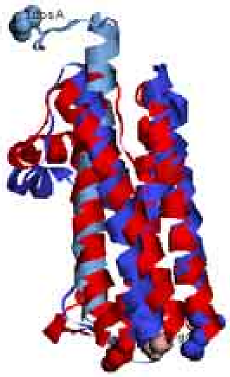
	1bgd_<br>(158)	GRANULOCYTE COLONY-STIMULATING FACTOR								

372	1bgd:_ (158)	GRANULOCYTE COLONY-STIMULATING FACTOR	**54**	49	3E-07	0.44	3.18	118	10.3% : 10.2% (9/87 : 12/118)	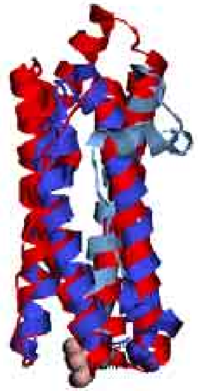
	1eum:A (161)	FERRITIN 1								
373	1bgd:_ (158)	GRANULOCYTE COLONY-STIMULATING FACTOR	**46**	49	3E-07	0.38	3.43	116	6.6% : 6.9% (4/61 : 8/116)	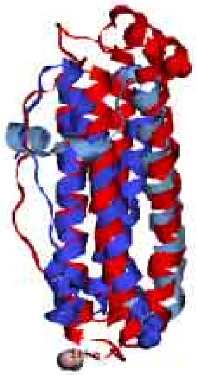
	1fha_ (173)	FERRITIN								
374	1h96:A (167)	FERRITIN LIGHT CHAIN 1	**123**	41	0.0001	0.36	3.35	113	7.3% : 12.4% (6/82 : 14/113)	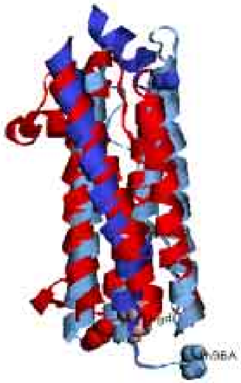
	1bgd_ (158)	GRANULOCYTE COLONY-STIMULATING FACTOR								

375	1bgd:_ (158)	GRANULOCYTE COLONY-STIMULATING FACTOR	**83**	34	0.008	0.31	3.19	64	5.1% : 12.5% (3/59 : 8/64)	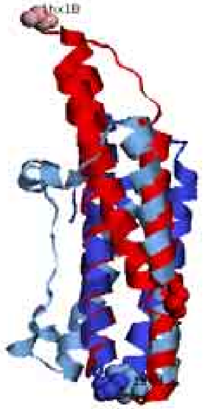
	1hx1:B (112)	BAG-FAMILY MOLECULAR CHAPERONE REGULATOR-1								
376	1bgd:_ (158)	GRANULOCYTE COLONY-STIMULATING FACTOR	**52**	48	5E-07	0.43	3.39	118	9.9% : 5.1% (9/91 : 6/118)	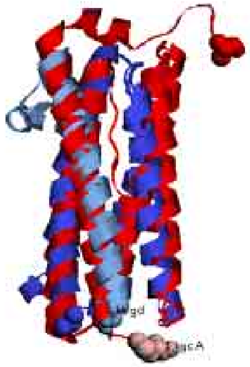
	1jgc:A (160)	bacterioferritin								
377	1bgd:_ (158)	GRANULOCYTE COLONY-STIMULATING FACTOR	**130**	38	0.0004	0.35	3.48	111	4.5% : 7.2% (4/88 : 8/111)	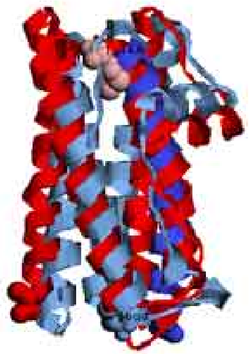
	1ji4:A (144)	NEUTROPHIL-ACTIVATING PROTEIN A								

378	1bgd:_ (158)	GRANULOCYTE COLONY-STIMULATING FACTOR	**130**	37	0.001	0.37	3.30	112	14.9% : 10.7% (7/47 : 12/112)	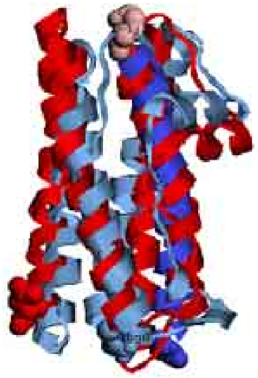
	1ji5:A (142)	Dlp-1								
379	1bgd:_ (158)	GRANULOCYTE COLONY-STIMULATING FACTOR	**128**	42	5E-05	0.37	3.17	110	6.0% : 11.8% (3/50 : 13/110)	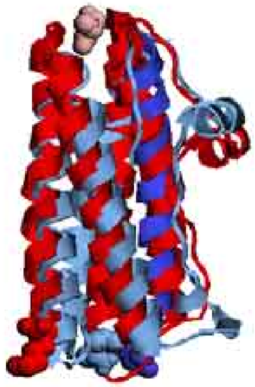
	1jig:A (146)	Dlp-2								
380	1bgd:_ (158)	GRANULOCYTE COLONY-STIMULATING FACTOR	**50**	41	5E-05	0.23	2.49	80	6.9% : 5.0% (4/58 : 4/80)	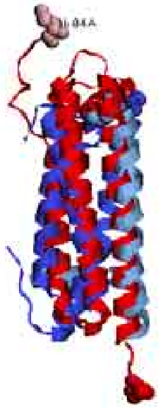
	1k04:A (142)	FOCAL ADHESION KINASE 1								

381	1bgd:_ (158)	GRANULOCYTE COLONY-STIMULATING FACTOR	**53**	48	7E-07	0.40	3.25	117	7.4% : 7.7% (4/54 : 9/117)	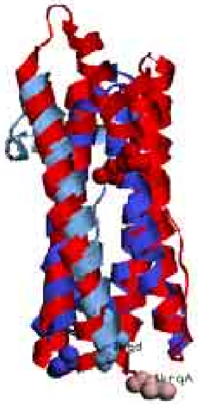
	1krq:A (164)	ferritin								
382	1bgd:_ (158)	GRANULOCYTE COLONY-STIMULATING FACTOR	**45**	44	8E-06	0.38	3.33	116	4.8% : 5.2% (3/62 : 6/116)	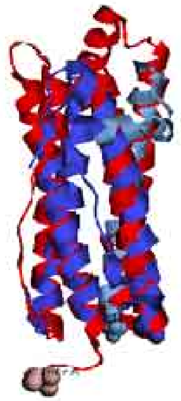
	1mfr:A (171)	M FERRITIN								
383	1bgd:_ (158)	GRANULOCYTE COLONY-STIMULATING FACTOR	**105**	41	5E-05	0.25	1.86	95	9.1% : 11.6% (5/55 : 11/95)	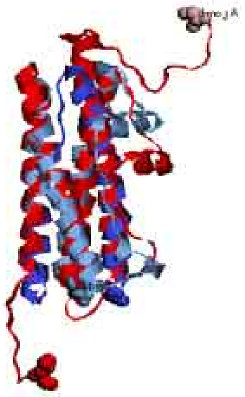
	1moj:A (180)	Dps-like ferritin								

384	1n1q:A (149)	DPS Protein	**37**	38	0.0005	0.39	2.98	102	8.0% : 5.9% (7/87 : 6/102)	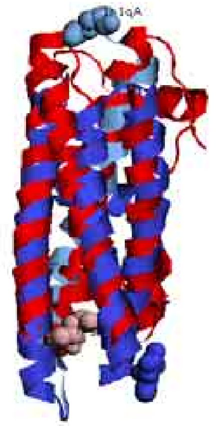
	1bgd_ (158)	GRANULOCYTE COLONY-STIMULATING FACTOR								
385	1bgd:_ (158)	GRANULOCYTE COLONY-STIMULATING FACTOR	**48**	49	2E-07	0.33	2.39	106	2.0% : 10.4% (1/51 : 11/106)	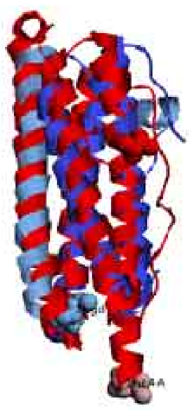
	1nf4:A (169)	bacterioferritin								
386	1bgd:_ (158)	GRANULOCYTE COLONY-STIMULATING FACTOR	**116**	46	3E-06	0.37	3.19	116	7.3% : 6.9% (7/96 : 8/116)	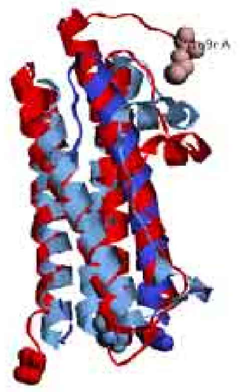
	1o9r:A (162)	AGROBACTERIUM TUMEFACIENS DPS								

387	1bgd:_ (158)	GRANULOCYTE COLONY-STIMULATING FACTOR	**131**	40	0.0001	0.27	2.79	91	9.1% : 9.9% (5/55 : 9/91)	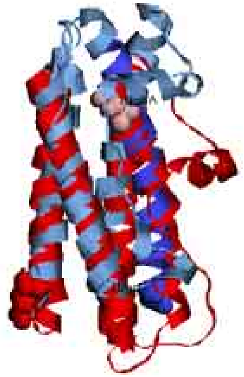
	1qgh:A (150)	NON-HEME IRON-CONTAINING FERRITIN								
388	1bgd:_ (158)	GRANULOCYTE COLONY-STIMULATING FACTOR	**46**	46	3E-06	0.27	3.46	94	7.1% : 6.4% (4/56 : 6/94)	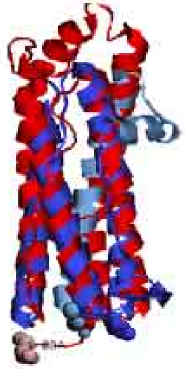
	1r03:A (170)	mitochondrial ferritin								
389	1bgd:_ (158)	GRANULOCYTE COLONY-STIMULATING FACTOR	**46**	44	8E-06	0.40	3.38	118	5.4% : 6.8% (3/56 : 8/118)	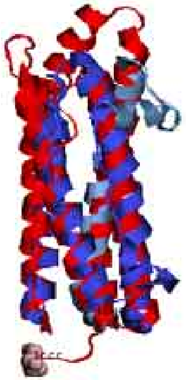
	1rcc_ (171)	L FERRITIN								

390	1bgd:_ (158)	GRANULOCYTE COLONY-STIMULATING FACTOR	**33**	36	0.003	0.34	2.67	97	6.2% : 12.4% (4/65 : 12/97)	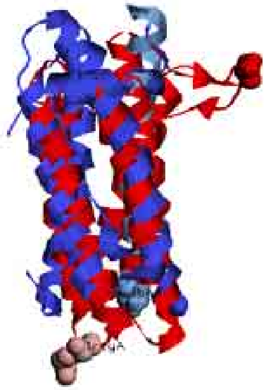
	1rty:A (159)	yvqk protein								
391	1bgd:_ (158)	GRANULOCYTE COLONY-STIMULATING FACTOR	**53**	51	5E-08	0.39	3.40	107	9.6% : 10.3% (8/83 : 11/107)	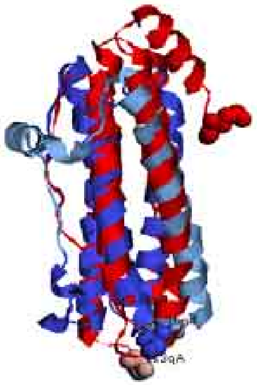
	1s3q:A (162)	ferritin								
392	1bgd:_ (158)	GRANULOCYTE COLONY-STIMULATING FACTOR	**51**	47	1E-06	0.46	2.46	120	8.7% : 7.5% (8/92 : 9/120)	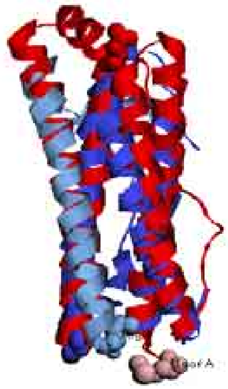
	1sof:A (155)	Bacterioferritin								

393	1bgd:_ (158)	GRANULOCYTE COLONY-STIMULATING FACTOR	**128**	46	2E-06	0.34	3.37	102	9.1% : 10.8% (8/88 : 11/102)	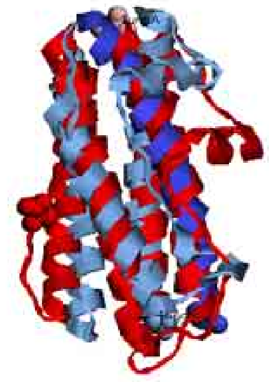
	1umn:A (151)	DPS-LIKE PEROXIDE RESISTANCE PROTEIN								
394	1bgd:_ (158)	GRANULOCYTE COLONY-STIMULATING FACTOR	**120**	35	0.005	0.34	3.15	111	12.5% : 7.2% (11/88 : 8/111)	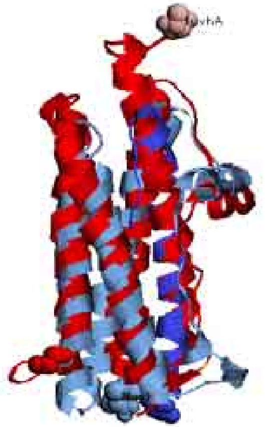
	1uvh:A (157)	STARVATION-INDUCED DNA PROTECTING PROTEIN								
395	1bgd:_ (158)	GRANULOCYTE COLONY-STIMULATING FACTOR	**58**	33	0.014	0.46	2.91	111	5.5% : 9.0% (3/55 : 10/111)	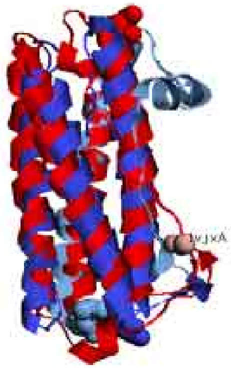
	1vjx:A (148)	putative ferritin-like diiron-carboxylate pro								

396	1bgd:_<br>(158)	GRANULOCYTE COLONY-STIMULATING FACTOR	**51**	50	1E-07	0.40	3.50	116	5.7% : 7.8%<br>(5/87 : 9/116)	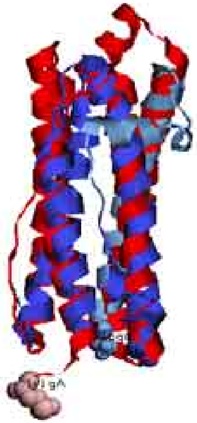
	1vlg:A<br>(164)	ferritin								
397	1bgd:_<br>(158)	GRANULOCYTE COLONY-STIMULATING FACTOR	**88**	35	0.006	0.27	3.04	63	3.7% : 7.9%<br>(2/54 : 5/63)	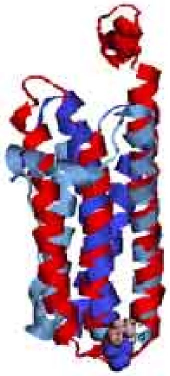
	1w9r:A<br>(119)	CHOLINE BINDING PROTEIN A								
398	1bgd:_<br>(158)	GRANULOCYTE COLONY-STIMULATING FACTOR	**117**	33	0.014	0.36	2.51	61	7.7% : 8.2%<br>(4/52 : 5/61)	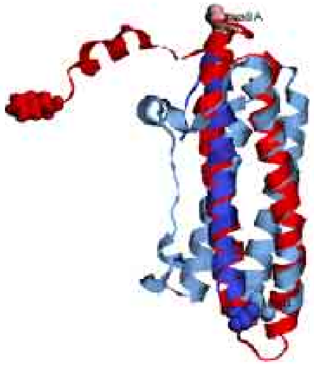
	1wa8:A<br>(99)	ESAT-6 LIKE PROTEIN ESXB								

399	1bgd:_ (158)	GRANULOCYTE COLONY-STIMULATING FACTOR	**138**	34	0.007	0.21	2.65	82	9.1% : 4.9% (6/66 : 4/82)	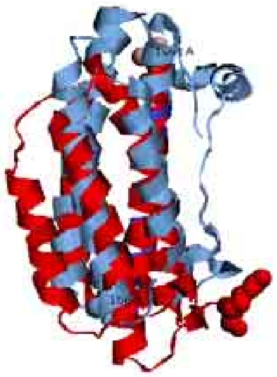
	1wvt:A (148)	hypothetical protein ST2180								
400	1bgd:_ (158)	GRANULOCYTE COLONY-STIMULATING FACTOR	**36**	37	0.001	0.33	3.22	116	10.5% : 8.6% (9/86 : 10/116)	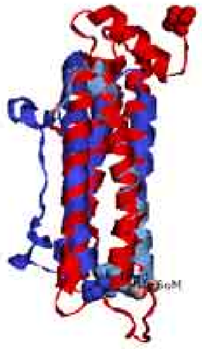
	1z6o:M (191)	Ferritin heavy chain								
401	1bgd:_ (158)	GRANULOCYTE COLONY-STIMULATING FACTOR	**52**	42	5E-05	0.21	3.41	85	12.2% : 9.4% (9/74 : 8/85)	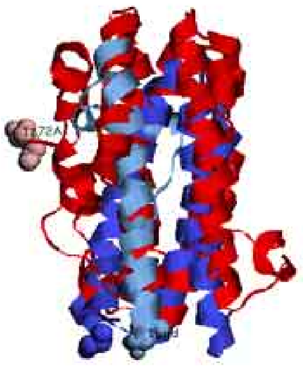
	1z72:A (217)	transcriptional regulator, putative								

402	1bgd:_ (158)	GRANULOCYTE COLONY-STIMULATING FACTOR	**109**	41	5E-05	0.31	3.19	111	13.3% : 8.1% (12/90 : 9/111)	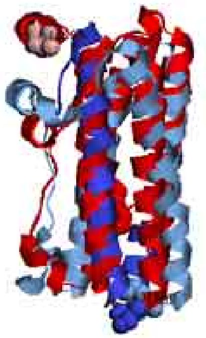
	1zs3:A (171)	Lactococcus lactis MG1363 DpsA								
403	1bgd:_ (158)	GRANULOCYTE COLONY-STIMULATING FACTOR	**114**	35	0.004	0.30	3.11	112	12.2% : 8.0% (11/90 : 9/112)	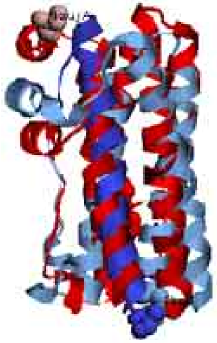
	1zuj:A (168)	hypothetical protein Llacc01001955								
404	2c41:A (155)	DPS FAMILY DNA-BINDING STRESS RESPONSE PROTEI	**36**	37	0.001	0.46	3.28	122	7.3% : 12.3% (4/55 : 15/122)	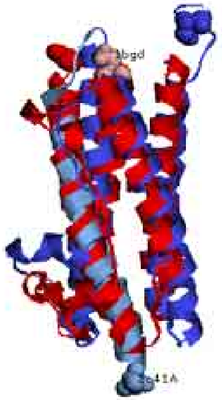
	1bgd_ (158)	GRANULOCYTE COLONY-STIMULATING FACTOR								

405	2d5k:A (148)	Dps family protein	**34**	37	0.001	0.36	2.99	100	10.6% : 11.0% (5/47 : 11/100)	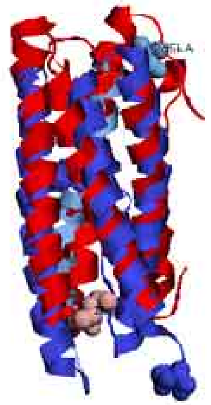
	1bgd_ (158)	GRANULOCYTE COLONY-STIMULATING FACTOR								
406	1bgd:_ (158)	GRANULOCYTE COLONY-STIMULATING FACTOR	**45**	49	2E-07	0.32	3.23	109	11.1% : 8.3% (9/81 : 9/109)	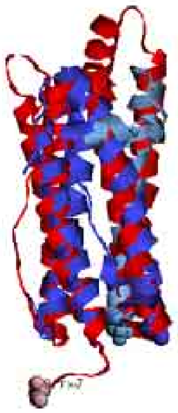
	2ffx:J (173)	ferritin light chain								
407	1bgd:_ (158)	GRANULOCYTE COLONY-STIMULATING FACTOR	**125**	42	3E-05	0.37	2.97	102	13.8% : 11.8% (12/87 : 12/102)	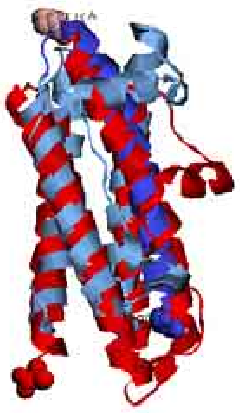
	2fjc:A (151)	Antigen TpF1								

408	1bgd:_ (158)	GRANULOCYTE COLONY-STIMULATING FACTOR	50	37	0.0008	0.42	2.10	114	7.3% : 6.1% (6/82 : 7/114)	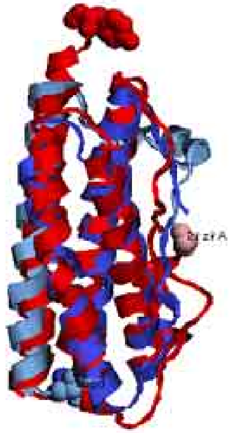
	2fzf:A (158)	hypothetical protein								
409	1bgd:_ (158)	GRANULOCYTE COLONY-STIMULATING FACTOR	35	34	0.007	0.33	3.12	94	12.3% : 6.4% (8/65 : 6/94)	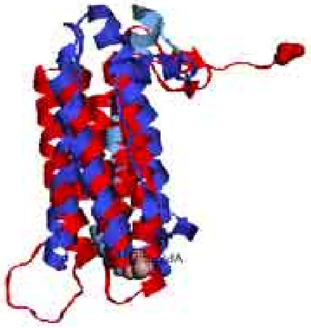
	2g2d:A (160)	ATP:cobalamin adenosyltransferase								
410	1bgd:_ (158)	GRANULOCYTE COLONY-STIMULATING FACTOR	126	36	0.002	0.41	2.42	114	8.5% : 5.3% (5/59 : 6/114)	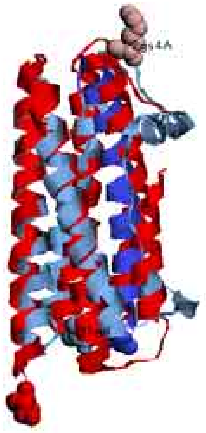
	2gs4:A (159)	Protein yciF								

411	1bgd:_ (158)	GRANULOCYTE COLONY-STIMULATING FACTOR	47	39	0.0003	0.36	2.89	112	2.2% : 10.7% (1/46 : 12/112)	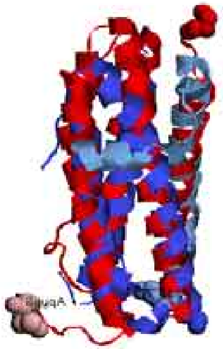
	2gyq:A (162)	ycfI, putative structural protein								
412	1bgd:_ (158)	GRANULOCYTE COLONY-STIMULATING FACTOR	57	43	2E-05	0.49	2.31	115	7.1% : 7.8% (6/85 : 9/115)	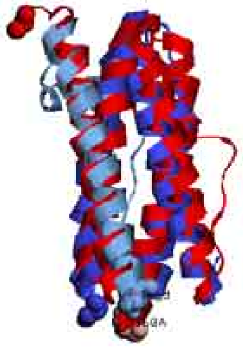
	2ib0:A (142)	CONSERVED HYPOTHETICAL ALANINE RICH PROTEIN								
413	1bgd:_ (158)	GRANULOCYTE COLONY-STIMULATING FACTOR	36	50	1E-07	0.27	2.91	108	10.5% : 9.3% (8/76 : 10/108)	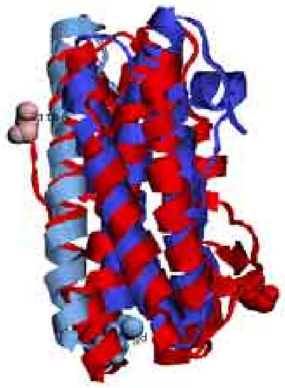
	2itb:A (199)	TRNA-(Ms(2)io(6)a)-hydroxylase, putative								

414	* 1jjz:A (29)	KALATA B1	**6**	33	0.019	0.47	2.40	27	50.0% : 55.6% (11/22 : 15/27)	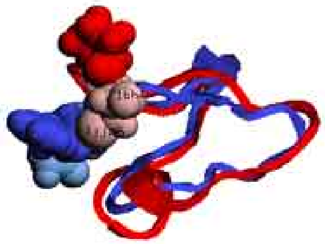
	* 1bh4_ (30)	CIRCULIN A								
415	* 1bh4:_ (30)	CIRCULIN A	**20**	31	0.066	0.82	2.28	27	8.3% : 55.6% (1/12 : 15/27)	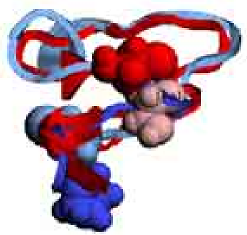
	* 1kal_ (29)	KALATA B1								
416	1bha:_ (67)	BACTERIORHODOPSIN	**43**	33	0.012	0.55	3.03	47	10.9% : 4.3% (5/46 : 2/47)	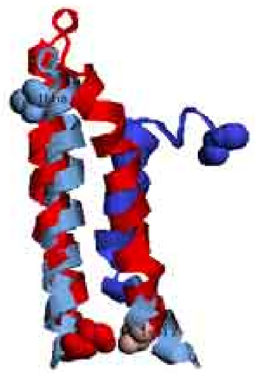
	1s5l:Z (117)	Photosystem II reaction center Z protein								

417	1dgl:A (237)	LECTIN	**123**	150	1E-37	0.78	1.26	219	43.8% : 42.5% (49/112 : 93/219)	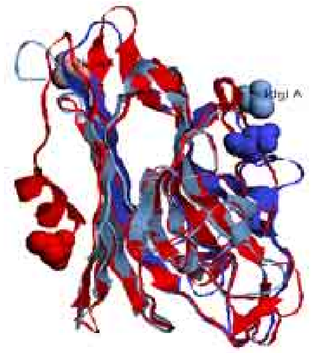
	1bjq:A (252)	LECTIN								
418	1mvq:A (236)	lectin, isoform 1	**123**	149	3E-37	0.79	1.28	219	48.7% : 46.1% (55/113 : 101/219)	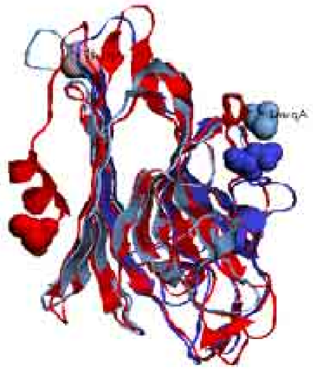
	1bjq:A (252)	LECTIN								
419	1bkz:A (135)	GALECTIN-7	**40**	36	0.002	0.28	2.20	88	7.4% : 12.5% (4/54 : 11/88)	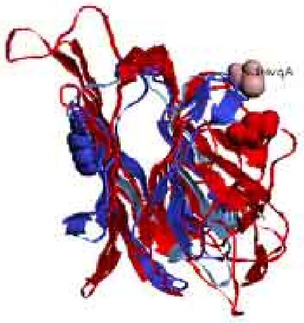
	1mvq:A (236)	lectin, isoform 1								

420	1bl8:A (97)	PROTEIN (POTASSIUM CHANNEL PROTEIN)	**62**	34	0.009	0.22	1.73	61	3.6% : 6.6% (2/56 : 4/61)	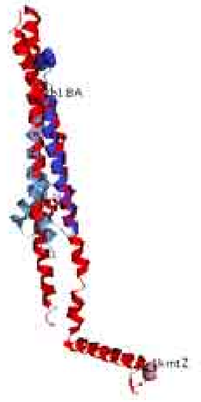
	1kmi:Z (178)	Chemotaxis protein cheZ								
421	1bmf:G (124)	BOVINE MITOCHONDRIAL F1-ATPASE	**65**	38	0.0004	0.76	2.69	93	3.7% : 6.5% (3/81 : 6/93)	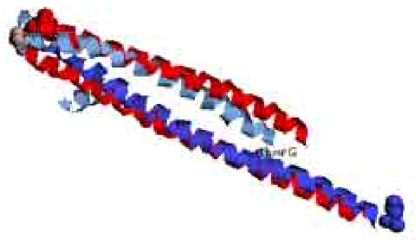
	1qbz:A (103)	PROTEIN (SIV GP41 ECTODOMAIN)								
422	2crb:A (97)	nuclear receptor binding factor 2	**54**	34	0.009	0.29	2.53	63	6.8% : 3.2% (4/59 : 2/63)	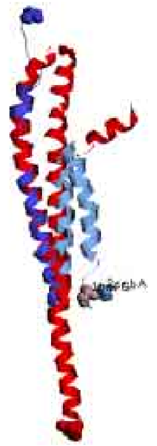
	1bmf:G (124)	BOVINE MITOCHONDRIAL F1-ATPASE								

423	1boo:A (283)	PROTEIN (N-4 CYTOSINE-SPECIFIC METHYLTRANSFER	**123**	33	0.041	0.25	2.96	139	7.4% : 15.8% (7/94 : 22/139)	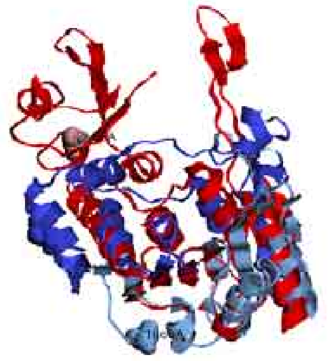
	2b25:A (258)	hypothetical protein								
424	2gb4:A (233)	Thiopurine S-methyltransferase	**89**	37	0.002	0.30	2.38	137	8.4% : 8.8% (7/83 : 12/137)	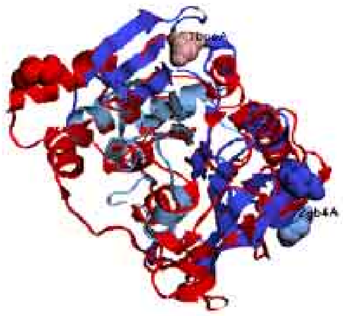
	1boo:A (283)	PROTEIN (N-4 CYTOSINE-SPECIFIC METHYLTRANSFER								
425	1dgl:A (237)	LECTIN	**123**	111	9E-26	0.78	1.46	171	44.1% : 40.4% (45/102 : 69/171)	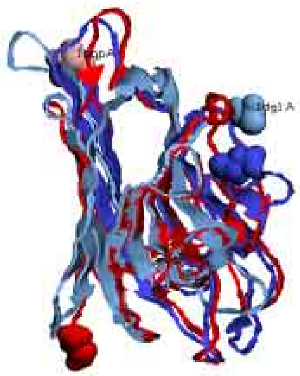
	1bqp:A (181)	PROTEIN (LECTIN)								

426	1mvq:A (236)	lectin, isoform 1	**123**	119	4E-28	0.76	1.30	168	42.2% : 41.7% (43/102 : 70/168)	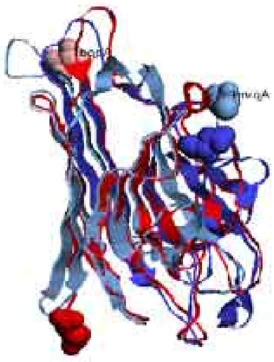
	1bqp:A (181)	PROTEIN (LECTIN)								
427	* 1g2b:A (63)	SPECTRIN ALPHA CHAIN	**22**	38	0.0004	0.64	0.74	51	35.1% : 33.3% (13/37 : 17/51)	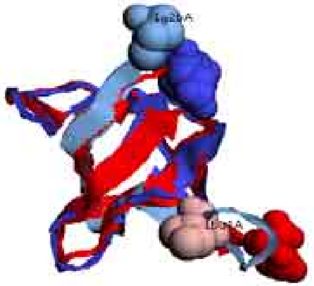
	* 1bu1:A (57)	PROTEIN (HEMOPOIETIC CELL KINASE)								
428	1buy:A (166)	PROTEIN (ERYTHROPOIETIN)	**117**	35	0.006	0.30	3.45	111	9.3% : 9.0% (8/86 : 10/111)	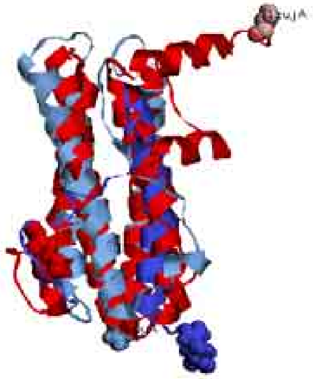
	1zuj:A (168)	hypothetical protein Llacc01001955								

429	1buy:A (166)	PROTEIN (ERYTHROPOIETIN)	**127**	33	0.026	0.32	3.07	100	9.4% : 11.0% (8/85 : 11/100)	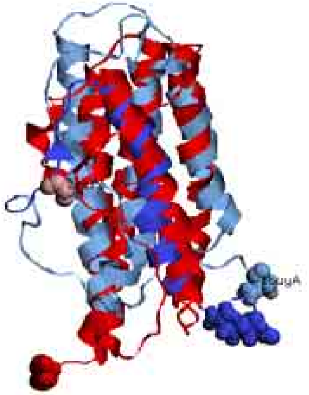
	2fzf:A (158)	hypothetical protein								
430	* 1byh:_ (214)	HYBRID	**59**	200	9E-53	0.75	0.95	211	100.0% : 100.0% (156/156 : 211/211)	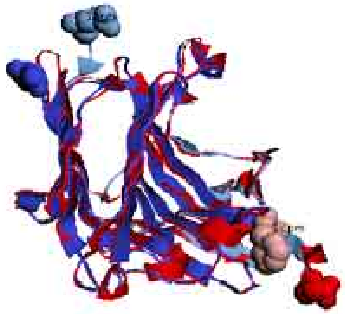
	* 1cpm_ (214)	CIRCULARLY PERMUTED								
431	* 1byh:_ (214)	HYBRID	**109**	37	0.001	0.32	3.12	134	12.7% : 9.7% (8/63 : 13/134)	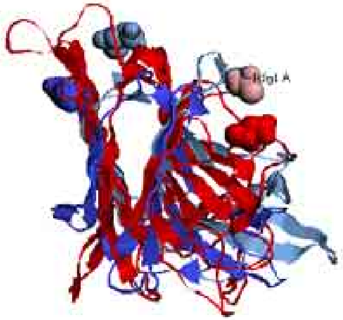
	* 1dgl:A (237)	LECTIN								

432	* 1mve:A (243)<br>* 1byh_ (214)	Truncated 1,3-1,4-beta-D-glucanase<br>HYBRID	**181**	95	8E-21	0.50	1.47	189	33.1% : 29.6% (48/145 : 56/189)	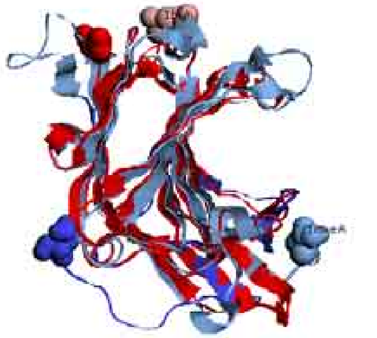
433	* 1byh:_ (214)<br>* 1s2b:A (201)	HYBRID<br>Scytalidopepsin B	**54**	38	0.0007	0.25	3.39	127	6.8% : 5.5% (7/103 : 7/127)	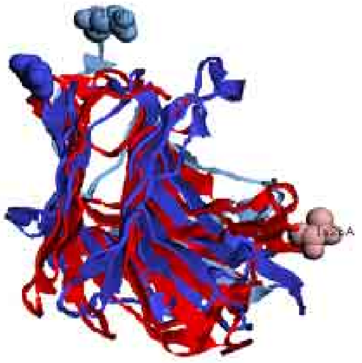
434	1gmi:A (136)<br>1byn:A (128)	PROTEIN KINASE C, EPSILON TYPE<br>PROTEIN (SYNAPTOTAGMIN I)	**120**	38	0.0006	0.21	1.63	103	21.7% : 22.3% (20/92 : 23/103)	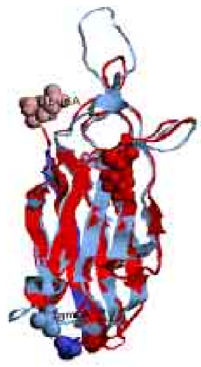

435	1dgl:A (237)	LECTIN	**123**	147	9E-37	0.86	1.58	217	40.5% : 43.8% (47/116 : 95/217)	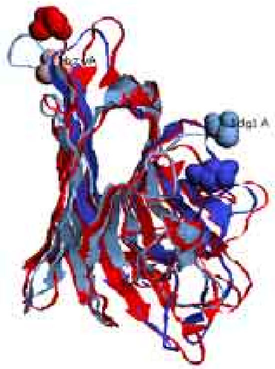
	1bzw:A (232)	PROTEIN (PEANUT LECTIN)								
436	1mvq:A (236)	lectin, isoform 1	**123**	161	8E-41	0.86	1.56	216	39.7% : 43.5% (46/116 : 94/216)	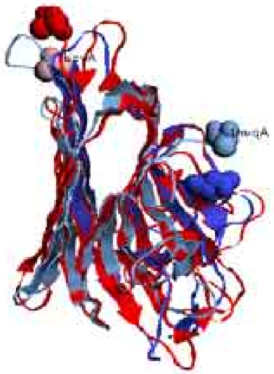
	1bzw:A (232)	PROTEIN (PEANUT LECTIN)								
437	1c02:A (166)	PHOSPHOTRANSFERASE YPD1P	**84**	32	0.037	0.24	3.25	81	6.9% : 6.2% (4/58 : 5/81)	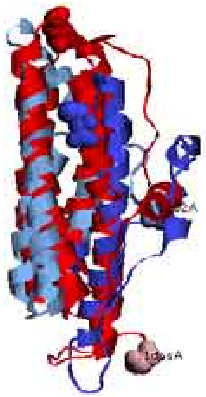
	1dps:A (159)	DPS								

438	1g33:A (73)	PARVALBUMIN ALPHA	**35**	35	0.004	0.48	2.12	62	21.1% : 14.5% (12/57 : 9/62)	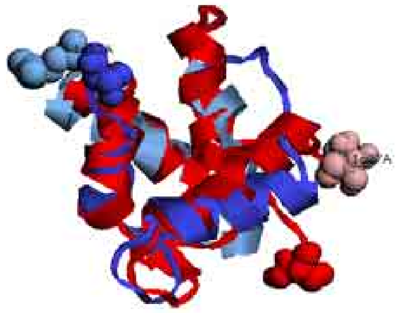
	1c07:A (95)	PROTEIN (EPIDERMAL GROWTH FACTOR RECEPTOR PAT								
439	1c1f:A (135)	PROTEIN (CONGERIN I)	**39**	35	0.004	0.31	2.77	96	7.2% : 7.3% (5/69 : 7/96)	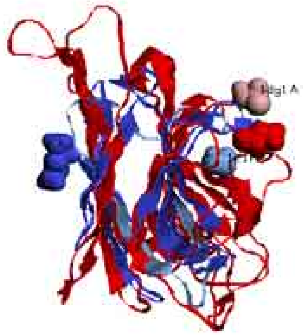
	1dgl:A (237)	LECTIN								
440	1c1y:A (167)	RAS-RELATED PROTEIN RAP-1A	**60**	49	3E-07	0.25	2.63	120	12.5% : 19.2% (10/80 : 23/120)	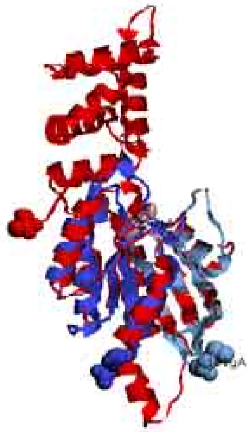
	1puj:A (262)	conserved hypothetical protein ylqF								

441	1c7v:A (68)	CALCIUM VECTOR PROTEIN	**33**	36	0.002	0.40	1.79	57	42.6% : 29.8% (23/54 : 17/57)	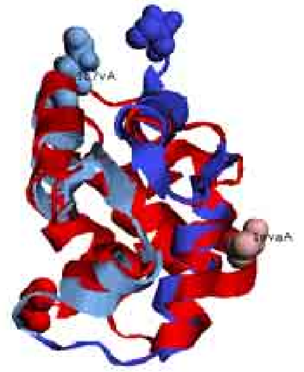
	1pva:A (109)	PARVALBUMIN								
442	1g5v:A (56)	SURVIVAL MOTOR NEURON PROTEIN 1	**19**	33	0.018	0.42	1.78	46	2.9% : 6.5% (1/34 : 3/46)	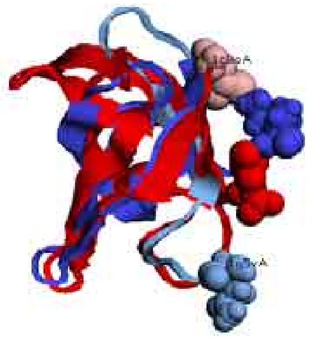
	1c9o:A (66)	COLD-SHOCK PROTEIN								
443	1j27:A (98)	hypothetical protein TT1725	**52**	36	0.002	0.30	3.47	74	8.2% : 9.5% (5/61 : 7/74)	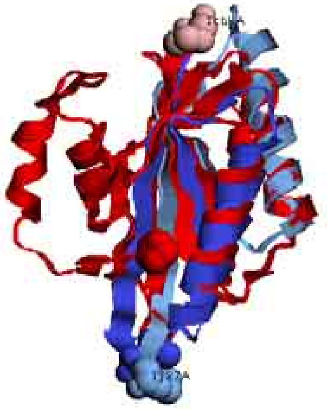
	1cbk:A (160)	PROTEIN (7,8-DIHYDRO-6-HYDROXYMETHYLPTERIN-PY								

444	1cxz:B (86)	PROTEIN (PKN)	**51**	36	0.002	0.48	2.02	64	5.6% : 7.8% (3/54 : 5/64)	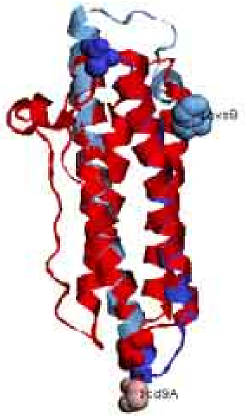
	1cd9:A (171)	PROTEIN (GRANULOCYTE COLONY-STIMULATING FACTO								
445	1cd9:A (171)	PROTEIN (GRANULOCYTE COLONY-STIMULATING FACTO	**59**	55	4E-09	0.40	3.45	123	7.4% : 8.9% (7/94 : 11/123)	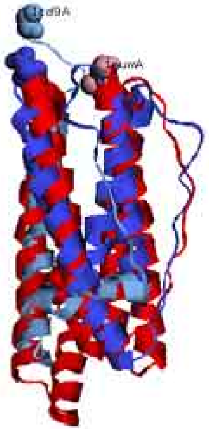
	1eum:A (161)	FERRITIN 1								
446	1h96:A (167)	FERRITIN LIGHT CHAIN 1	**118**	48	6E-07	0.34	3.50	115	9.7% : 12.2% (9/93 : 14/115)	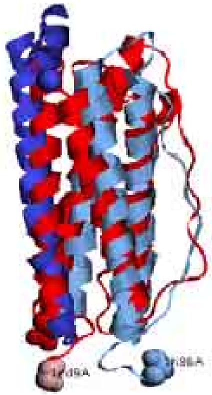
	1cd9:A (171)	PROTEIN (GRANULOCYTE COLONY-STIMULATING FACTO								

447	1cd9:A<br>(171)<br>1ji4:A<br>(144)	PROTEIN (GRANULOCYTE COLONY-STIMULATING FACTO<br>NEUTROPHIL-ACTIVATING PROTEIN A	**63**	38	0.0005	0.40	3.26	115	9.1% : 7.8%<br>(8/88 : 9/115)	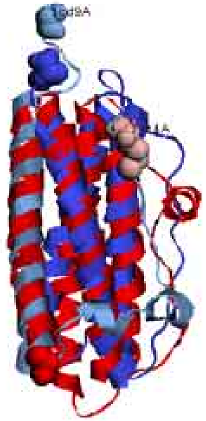
448	1cd9:A<br>(171)<br>1ji5:A<br>(142)	PROTEIN (GRANULOCYTE COLONY-STIMULATING FACTO<br>Dlp-1	**148**	33	0.022	0.30	3.08	99	11.6% : 9.1%<br>(10/86 : 9/99)	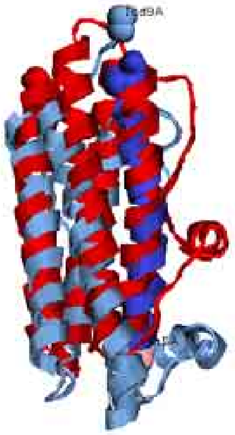
449	1cd9:A<br>(171)<br>1jig:A<br>(146)	PROTEIN (GRANULOCYTE COLONY-STIMULATING FACTO<br>Dlp-2	**139**	37	0.001	0.38	2.53	109	12.0% : 7.3%<br>(11/92 : 8/109)	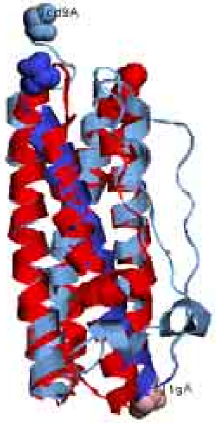

450	1cd9:A (171)	PROTEIN (GRANULOCYTE COLONY-STIMULATING FACTO	**58**	51	6E-08	0.38	3.40	122	9.6% : 9.8% (9/94 : 12/122)	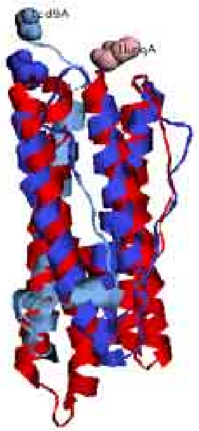
	1krq:A (164)	ferritin								
451	1cd9:A (171)	PROTEIN (GRANULOCYTE COLONY-STIMULATING FACTO	**121**	41	6E-05	0.30	2.85	111	10.9% : 14.4% (7/64 : 16/111)	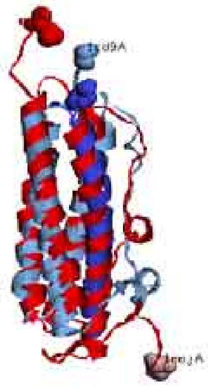
	1moj:A (180)	Dps-like ferritin								
452	1cd9:A (171)	PROTEIN (GRANULOCYTE COLONY-STIMULATING FACTO	**59**	37	0.001	0.34	3.00	107	16.3% : 15.9% (14/86 : 17/107)	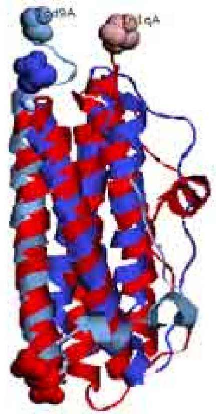
	1n1q:A (149)	DPS Protein								

453	1o9r:A (162)	AGROBACTERIUM TUMEFACIENS DPS	**42**	42	3E-05	0.38	3.08	115	6.8% : 7.0% (4/59 : 8/115)	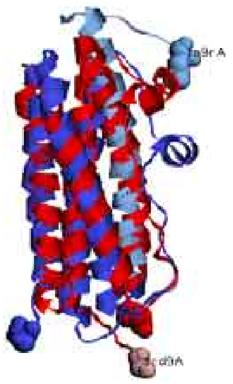
	1cd9:A (171)	PROTEIN (GRANULOCYTE COLONY-STIMULATING FACTO								
454	1cd9:A (171)	PROTEIN (GRANULOCYTE COLONY-STIMULATING FACTO	**143**	37	0.001	0.33	2.77	107	10.2% : 10.3% (9/88 : 11/107)	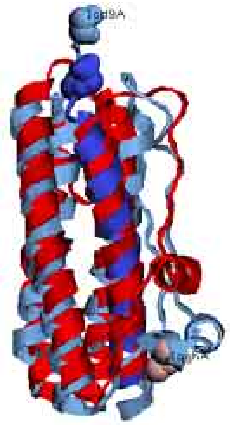
	1qgh:A (150)	NON-HEME IRON-CONTAINING FERRITIN								
455	1cd9:A (171)	PROTEIN (GRANULOCYTE COLONY-STIMULATING FACTO	**58**	54	1E-08	0.35	3.15	108	7.8% : 11.1% (6/77 : 12/108)	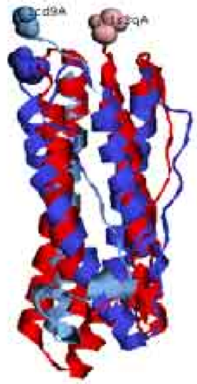
	1s3q:A (162)	ferritin								

456	1cd9:A (171)	PROTEIN (GRANULOCYTE COLONY-STIMULATING FACTO	**55**	45	3E-06	0.40	2.75	119	8.9% : 8.4% (8/90 : 10/119)	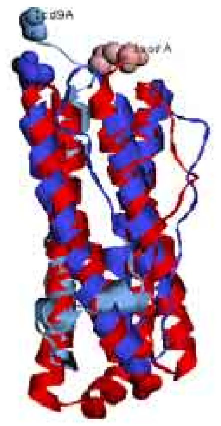
	1sof:A (155)	Bacterioferritin								
457	1cd9:A (171)	PROTEIN (GRANULOCYTE COLONY-STIMULATING FACTO	**145**	43	2E-05	0.30	2.65	98	14.8% : 10.2% (13/88 : 10/98)	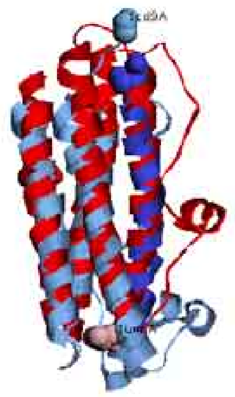
	1umn:A (151)	DPS-LIKE PEROXIDE RESISTANCE PROTEIN								
458	1cd9:A (171)	PROTEIN (GRANULOCYTE COLONY-STIMULATING FACTO	**134**	36	0.002	0.34	3.27	106	9.5% : 11.3% (6/63 : 12/106)	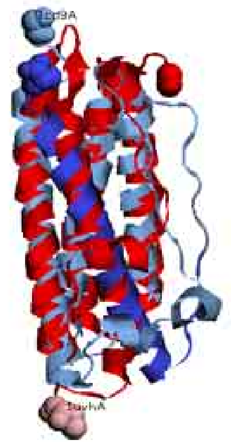
	1uvh:A (157)	STARVATION-INDUCED DNA PROTECTING PROTEIN								

459	1cd9:A (171)	PROTEIN (GRANULOCYTE COLONY-STIMULATING FACTO	**62**	34	0.011	0.41	2.25	108	5.1% : 9.3% (3/59 : 10/108)	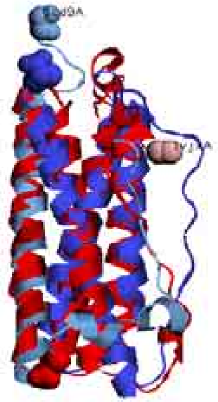
	1vjx:A (148)	putative ferritin-like diiron-carboxylate pro								
460	1cd9:A (171)	PROTEIN (GRANULOCYTE COLONY-STIMULATING FACTO	**54**	54	1E-08	0.38	3.30	122	6.4% : 7.4% (6/94 : 9/122)	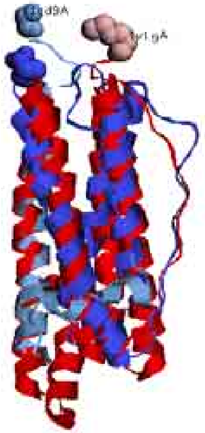
	1vlg:A (164)	ferritin								
461	1cd9:A (171)	PROTEIN (GRANULOCYTE COLONY-STIMULATING FACTO	**35**	42	4E-05	0.24	3.08	115	5.3% : 8.7% (3/57 : 10/115)	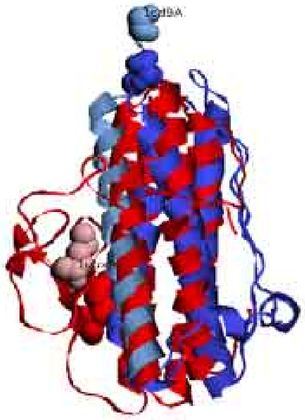
	1yux:A (201)	Nigerythrin								

462	1cd9:A (171)	PROTEIN (GRANULOCYTE COLONY-STIMULATING FACTO	**123**	34	0.008	0.27	2.72	109	10.5% : 8.3% (9/86 : 9/109)	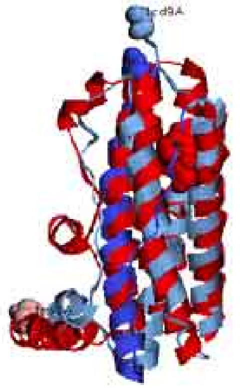
	1zs3:A (171)	Lactococcus lactis MG1363 DpsA								
463	1cd9:A (171)	PROTEIN (GRANULOCYTE COLONY-STIMULATING FACTO	**117**	38	0.0006	0.30	3.48	114	7.9% : 11.4% (5/63 : 13/114)	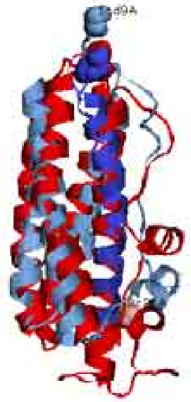
	2c2f:A (178)	DNA-BINDING STRESS RESPONSE PROTEIN								
464	1cd9:A (171)	PROTEIN (GRANULOCYTE COLONY-STIMULATING FACTO	**138**	39	0.0004	0.34	3.25	107	7.3% : 11.2% (6/82 : 12/107)	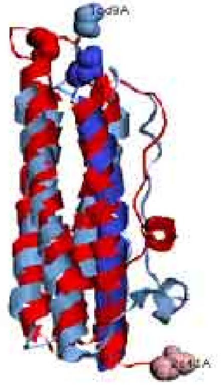
	2c41:A (155)	DPS FAMILY DNA-BINDING STRESS RESPONSE PROTEI								

465	2clb:A (169)	DPS-LIKE PROTEIN	**44**	42	4E-05	0.34	2.59	118	13.8% : 4.2% (12/87 : 5/118)	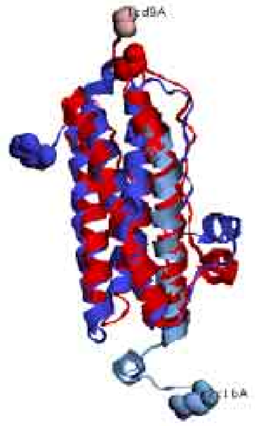
	1cd9:A (171)	PROTEIN (GRANULOCYTE COLONY-STIMULATING FACTO								
466	1cd9:A (171)	PROTEIN (GRANULOCYTE COLONY-STIMULATING FACTO	**60**	38	0.0006	0.40	2.93	116	13.3% : 11.2% (12/90 : 13/116)	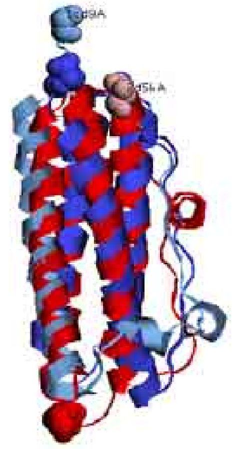
	2d5k:A (148)	Dps family protein								
467	1cd9:A (171)	PROTEIN (GRANULOCYTE COLONY-STIMULATING FACTO	**48**	44	1E-05	0.31	3.26	116	2.9% : 8.6% (2/68 : 10/116)	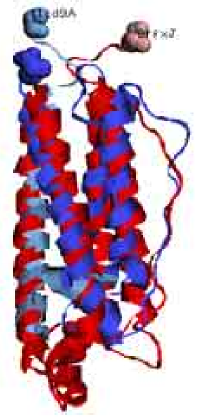
	2ffx:J (173)	ferritin light chain								

468	1cd9:A<br>(171)<br><br>2fjc:A<br>(151)	PROTEIN (GRANULOCYTE COLONY-STIMULATING FACTO<br><br>Antigen TpF1	**141**	38	0.0004	0.37	2.78	107	17.8% : 8.4%<br>(16/90 : 9/107)	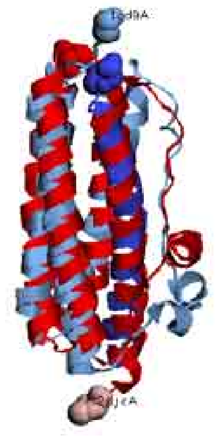
469	1cd9:A<br>(171)<br><br>2fzf:A<br>(158)	PROTEIN (GRANULOCYTE COLONY-STIMULATING FACTO<br><br>hypothetical protein	**55**	41	6E-05	0.40	2.95	117	6.8% : 7.7%<br>(6/88 : 9/117)	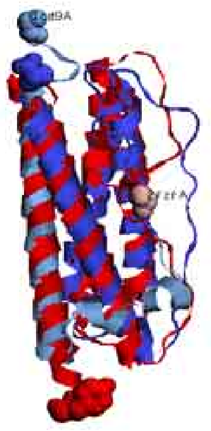
470	1cd9:A<br>(171)<br><br>2ib0:A<br>(142)	PROTEIN (GRANULOCYTE COLONY-STIMULATING FACTO<br><br>CONSERVED HYPOTHETICAL ALANINE RICH PROTEIN	**62**	37	0.001	0.48	2.72	123	6.5% : 6.5%<br>(6/92 : 8/123)	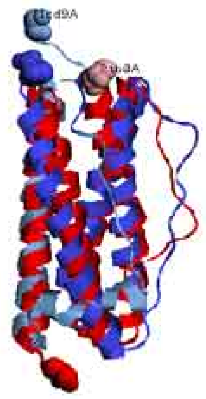

471	1cd9:A (171)	PROTEIN (GRANULOCYTE COLONY-STIMULATING FACTO	**126**	36	0.003	0.20	3.02	105	12.8% : 11.4% (11/86 : 12/105)	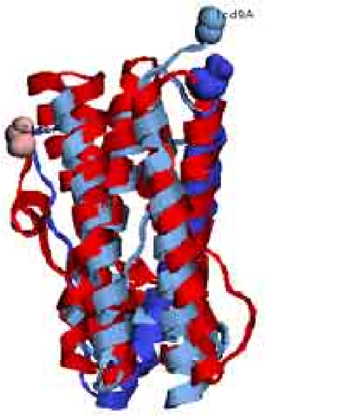
	2itb:A (199)	TRNA-(Ms(2)io(6)a)-hydroxylase, putative								
472	1ce3:A (54)	PROTEIN (API)	**27**	34	0.007	0.72	2.00	44	67.9% : 68.2% (19/28 : 30/44)	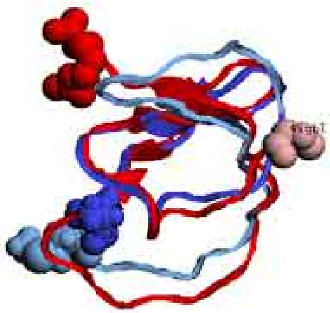
	4sgb:I (51)	POTATO INHIBITOR, PCI-1								
473	1cgn:_ (125)	CYTOCHROME C	**75**	34	0.01	0.25	2.66	67	2.1% : 13.4% (1/48 : 9/67)	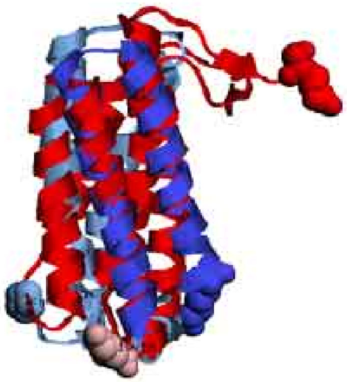
	1wvt:A (148)	hypothetical protein ST2180								

474	1cgn:_ (125)	CYTOCHROME C	**60**	37	0.0008	0.21	2.51	63	16.0% : 11.1% (8/50 : 7/63)	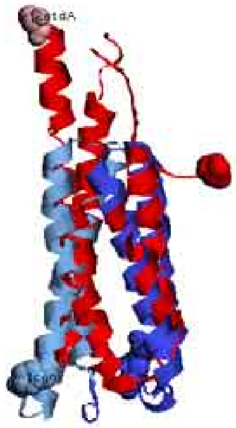
	2etd:A (145)	lemA protein								
475	1cgn:_ (125)	CYTOCHROME C	**88**	43	2E-05	0.22	3.22	69	18.0% : 7.2% (9/50 : 5/69)	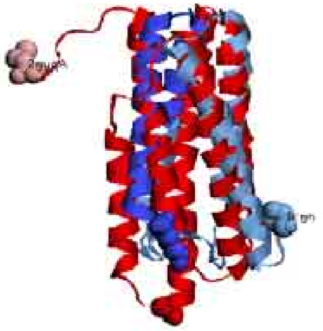
	2gyq:A (162)	ycfI, putative structural protein								
476	1clf:_ (55)	FERREDOXIN	**29**	32	0.046	0.54	2.91	41	30.6% : 36.6% (11/36 : 15/41)	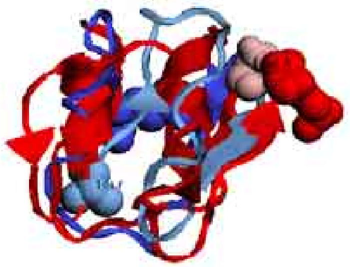
	1dwl:A (59)	FERREDOXIN I								

477	1jgc:A (160)	bacterioferritin	**34**	43	2E-05	0.40	2.74	115	5.3% : 8.7% (3/57 : 10/115)	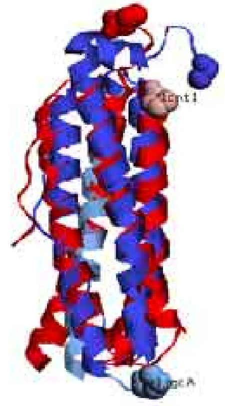
	1cnt:1 (152)	CILIARY NEUROTROPHIC FACTOR								
478	1cnt:1 (152)	CILIARY NEUROTROPHIC FACTOR	**124**	35	0.004	0.39	3.41	108	7.4% : 8.3% (4/54 : 9/108)	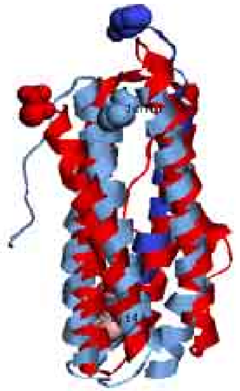
	1ji4:A (144)	NEUTROPHIL-ACTIVATING PROTEIN A								
479	1cnt:1 (152)	CILIARY NEUROTROPHIC FACTOR	**121**	36	0.002	0.45	3.29	111	11.2% : 9.0% (10/89 : 10/111)	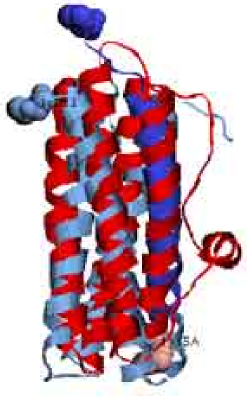
	1ji5:A (142)	Dlp-1								

480	1cnt:1 (152)	CILIARY NEUROTROPHIC FACTOR	**118**	37	0.001	0.50	3.44	115	13.6% : 10.4% (12/88 : 12/115)	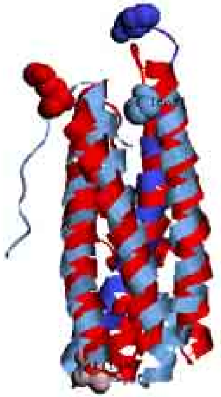
	1jig:A (146)	Dlp-2								
481	1cnt:1 (152)	CILIARY NEUROTROPHIC FACTOR	**119**	42	3E-05	0.42	2.48	87	6.9% : 9.2% (4/58 : 8/87)	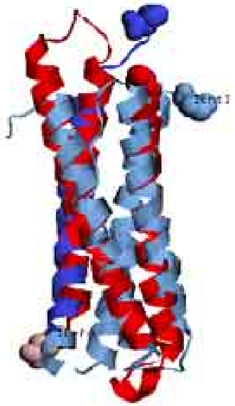
	1lvf:A (106)	syntaxin 6								
482	1cnt:1 (152)	CILIARY NEUROTROPHIC FACTOR	**114**	41	6E-05	0.51	3.46	116	10.0% : 8.6% (9/90 : 10/116)	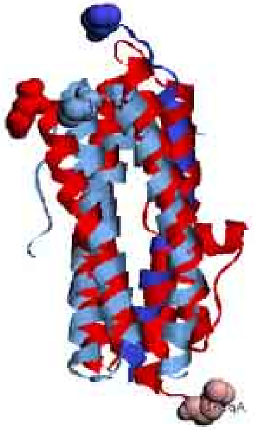
	1n1q:A (149)	DPS Protein								

483	1cnt:1 (152)	CILIARY NEUROTROPHIC FACTOR	**60**	62	4E-11	0.21	2.64	82	9.2% : 9.8% (7/76 : 8/82)	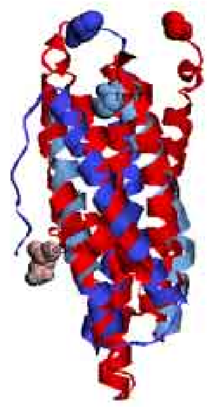
	1o5h:A (200)	Formiminotetrahydrofolate cyclodeaminase								
484	1qgh:A (150)	NON-HEME IRON-CONTAINING FERRITIN	**113**	42	3E-05	0.37	3.05	109	5.5% : 9.2% (3/55 : 10/109)	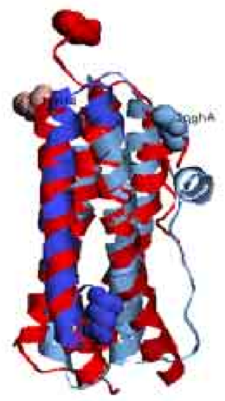
	1cnt:1 (152)	CILIARY NEUROTROPHIC FACTOR								
485	1cnt:1 (152)	CILIARY NEUROTROPHIC FACTOR	**34**	40	0.0002	0.42	3.06	120	10.9% : 9.2% (10/92 : 11/120)	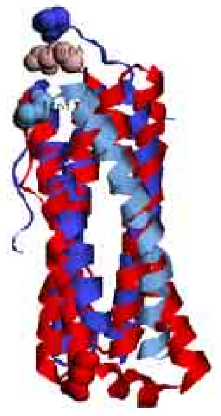
	1sof:A (155)	Bacterioferritin								

486	1umn:A (151)	DPS-LIKE PEROXIDE RESISTANCE PROTEIN	114	44	1E-05	0.39	3.17	112	10.8% : 10.7% (10/93 : 12/112)	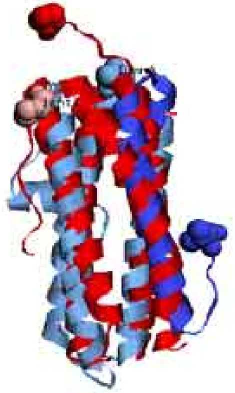
	1cnt:1 (152)	CILIARY NEUROTROPHIC FACTOR								
487	1vjx:A (148)	putative ferritin-like diiron-carboxylate pro	114	38	0.0005	0.38	3.42	107	14.0% : 12.1% (8/57 : 13/107)	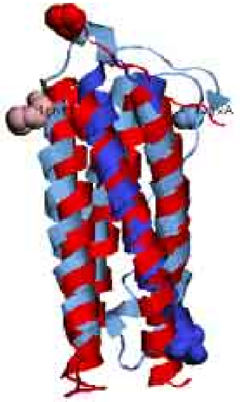
	1cnt:1 (152)	CILIARY NEUROTROPHIC FACTOR								
488	1woz:A (159)	177aa long conserved hypothetical protein (ST	47	34	0.01	0.25	3.31	82	4.9% : 13.4% (3/61 : 11/82)	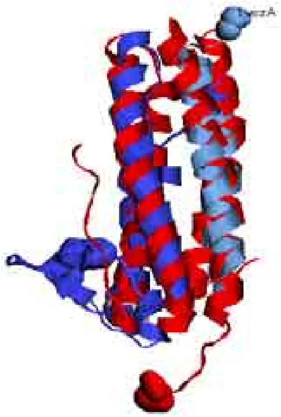
	1cnt:1 (152)	CILIARY NEUROTROPHIC FACTOR								

489	1wvt:A (148)	hypothetical protein ST2180	**40**	39	0.0003	0.30	2.91	90	5.8% : 7.8% (3/52 : 7/90)	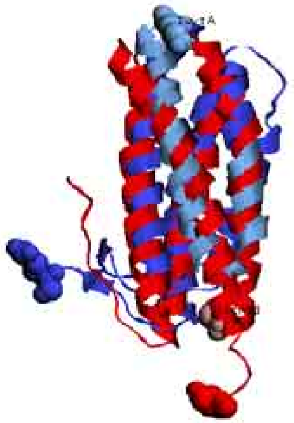
	1cnt:1 (152)	CILIARY NEUROTROPHIC FACTOR								
490	1cnt:1 (152)	CILIARY NEUROTROPHIC FACTOR	**88**	37	0.001	0.27	1.99	80	7.7% : 10.0% (6/78 : 8/80)	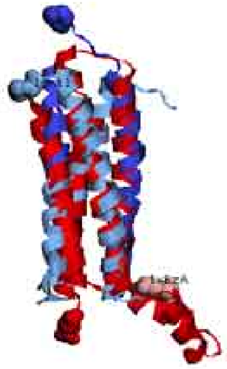
	1x8z:A (151)	invertase/pectin methylesterase inhibitor fam								
491	1cnt:1 (152)	CILIARY NEUROTROPHIC FACTOR	**38**	38	0.0004	0.36	3.25	112	8.0% : 7.1% (7/87 : 8/112)	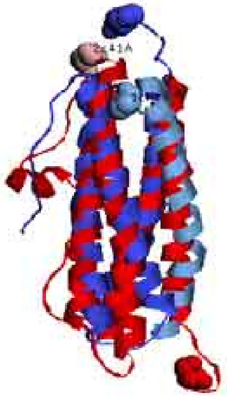
	2c41:A (155)	DPS FAMILY DNA-BINDING STRESS RESPONSE PROTEI								

492	2clb:A (169)	DPS-LIKE PROTEIN	**55**	39	0.0003	0.33	2.99	104	9.6% : 9.6% (5/52 : 10/104)	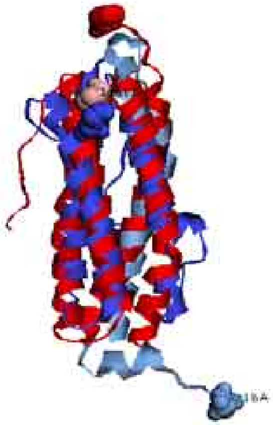
	1cnt:1 (152)	CILIARY NEUROTROPHIC FACTOR								
493	1cnt:1 (152)	CILIARY NEUROTROPHIC FACTOR	**115**	40	0.0001	0.52	3.50	118	13.5% : 7.6% (12/89 : 9/118)	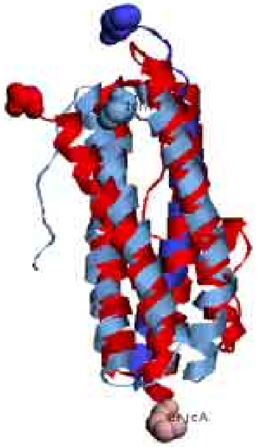
	2fjc:A (151)	Antigen TpF1								
494	1cnt:1 (152)	CILIARY NEUROTROPHIC FACTOR	**36**	37	0.001	0.47	2.77	123	8.7% : 10.6% (8/92 : 13/123)	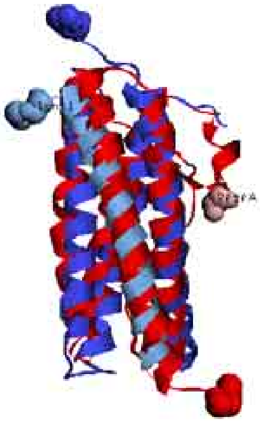
	2fzf:A (158)	hypothetical protein								

495	1cnt:1 (152)	CILIARY NEUROTROPHIC FACTOR	**43**	42	4E-05	0.44	2.83	111	5.9% : 6.3% (5/85 : 7/111)	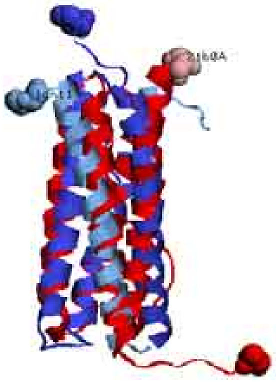
	2ib0:A (142)	CONSERVED HYPOTHETICAL ALANINE RICH PROTEIN								
496	* 1dyp:A (271)	KAPPA-CARRAGEENASE	**84**	64	2E-11	0.34	1.62	162	24.6% : 24.7% (31/126 : 40/162)	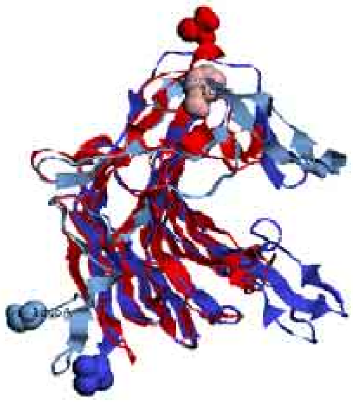
	* 1cpm_ (214)	CIRCULARLY PERMUTED								
497	* 1gbg:_ (214)	(1,3-1,4)-BETA-D-GLUCAN 4 GLUCANOHYDROLASE	**59**	223	9E-60	0.75	0.96	211	78.8% : 75.4% (123/156 : 159/211)	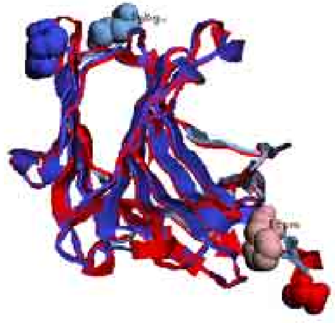
	* 1cpm_ (214)	CIRCULARLY PERMUTED								

498	* 1gmm:A (126)	CBM6	**34**	34	0.006	0.27	3.45	80	3.3% : 2.5% (2/60 : 2/80)	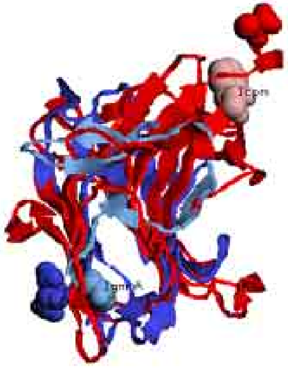
	* 1cpm_ (214)	CIRCULARLY PERMUTED								
499	* 1gv9:A (224)	P58/ERGIC-53	**47**	38	0.001	0.23	2.93	143	8.3% : 9.8% (10/121 : 14/143)	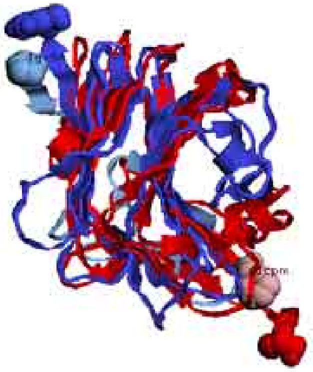
	* 1cpm_ (214)	CIRCULARLY PERMUTED								
500	* 1cpm:_ (214)	CIRCULARLY PERMUTED	**164**	38	0.0007	0.25	2.89	138	9.8% : 13.0% (11/112 : 18/138)	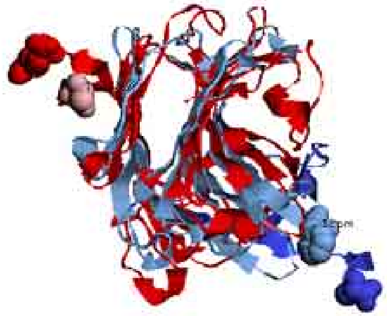
	* 1oq1:A (223)	Protein yesU								

501	* 1cpm:_ (214)	CIRCULARLY PERMUTED	158	36	0.004	0.29	2.87	148	8.3% : 8.1% (10/121 : 12/148)	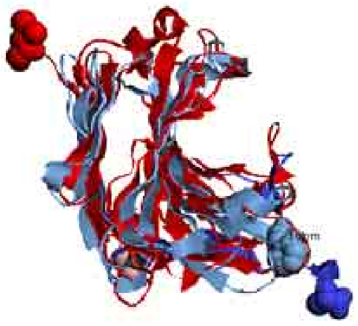
	* 2cws:A (227)	alginate lyase A1-II'								
502	1cpq:_ (129)	CYTOCHROME C'	68	42	3E-05	0.23	3.32	98	12.5% : 11.2% (11/88 : 11/98)	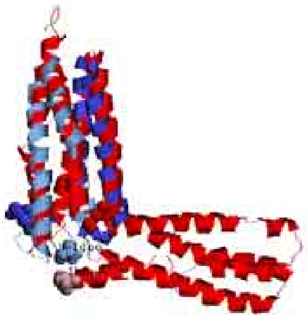
	1h6g:A (255)	ALPHA-1 CATENIN								
503	1cpq:_ (129)	CYTOCHROME C'	92	35	0.003	0.21	2.85	87	12.5% : 10.3% (9/72 : 9/87)	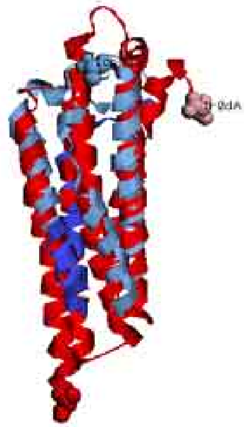
	1r0d:A (194)	Huntingtin Interacting Protein 12								

504	1cpq:_ (129)	CYTOCHROME C'	71	37	0.0009	0.47	3.34	74	13.9% : 16.2% (10/72 : 12/74)	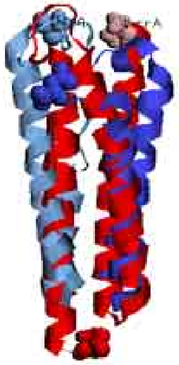
	1wcr:A (103)	PTS SYSTEM, N,N'-DIACETYLCHITOBIOSE-SPECIFIC								
505	1cpq:_ (129)	CYTOCHROME C'	78	32	0.026	0.24	3.31	69	4.3% : 2.9% (2/47 : 2/69)	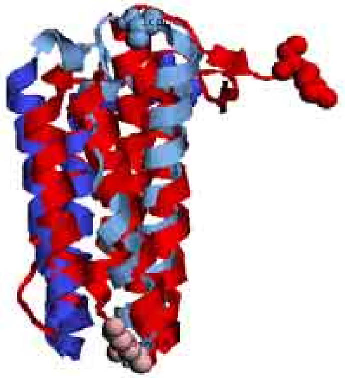
	1wvt:A (148)	hypothetical protein ST2180								
506	1cpq:_ (129)	CYTOCHROME C'	65	34	0.01	0.41	2.85	94	4.4% : 10.6% (4/91 : 10/94)	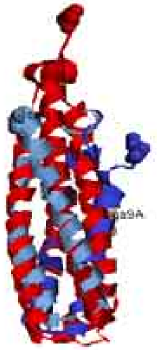
	1ya9:A (169)	Apolipoprotein E								

507	1cpq:_ (129)	CYTOCHROME C'	41	34	0.01	0.33	2.88	78	8.3% : 7.7% (4/48 : 6/78)	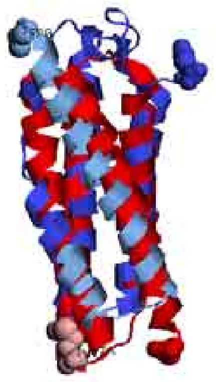
	1yo7:A (120)	Regulatory protein rop								
508	1cpq:_ (129)	CYTOCHROME C'	67	32	0.037	0.36	3.44	73	6.9% : 5.5% (5/72 : 4/73)	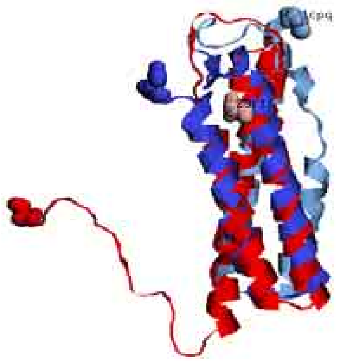
	2dl1:A (116)	Spartin								
509	1cpq:_ (129)	CYTOCHROME C'	93	34	0.009	0.24	3.21	71	17.0% : 14.1% (9/53 : 10/71)	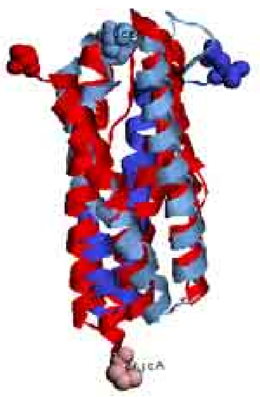
	2fjc:A (151)	Antigen TpF1								

510	1cpq:_ (129)	CYTOCHROME C'	**48**	33	0.021	0.23	2.10	61	13.0% : 8.2% (6/46 : 5/61)	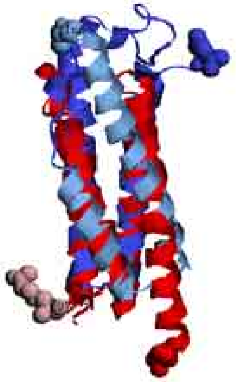
	2gsc:A (114)	conserved hypothetical protein								
511	1csm:A (253)	CHORISMATE MUTASE	**154**	33	0.025	0.44	2.80	123	6.7% : 19.5% (7/105 : 24/123)	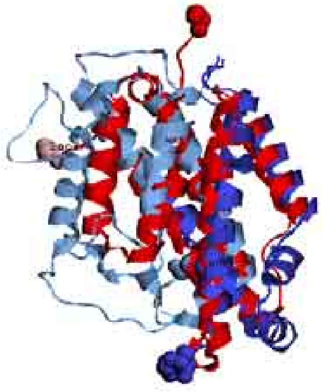
	2ao2:A (165)	Chorismate mutase								
512	1g4y:B (82)	CALCIUM-ACTIVATED POTASSIUM CHANNEL RSK2	**26**	37	0.0007	0.44	1.89	59	7.7% : 6.8% (3/39 : 4/59)	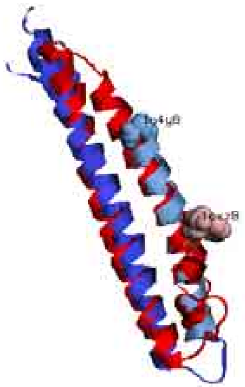
	1cxz:B (86)	PROTEIN (PKN)								

513	2j0n:A (171)	INVASIN IPAD	**118**	36	0.002	0.53	2.34	67	9.4% : 7.5% (6/64 : 5/67)	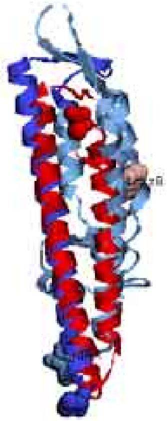
	1cxz:B (86)	PROTEIN (PKN)								
514	1ihn:A (113)	hypothetical protein MTH938	**82**	33	0.015	0.22	3.11	51	18.2% : 2.0% (6/33 : 1/51)	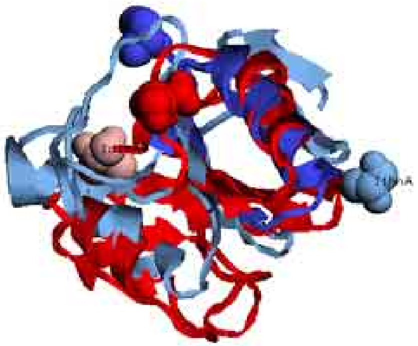
	1d1z:A (101)	SAP SH2 DOMAIN								
515	1d5c:A (162)	RAB6 GTPASE	**59**	40	0.0002	0.25	2.93	119	9.5% : 16.0% (8/84 : 19/119)	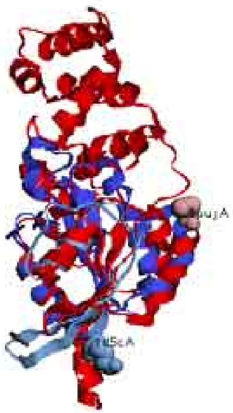
	1puj:A (262)	conserved hypothetical protein ylqF								

516	1d5w:A (123)	TRANSCRIPTIONAL REGULATORY PROTEIN FIXJ	**59**	32	0.026	0.28	3.31	86	9.2% : 15.1% (7/76 : 13/86)	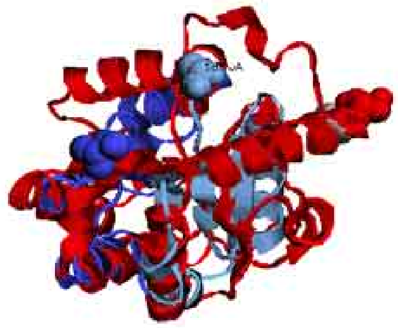
	2bfw:A (196)	GLGA GLYCOGEN SYNTHASE								
517	1f07:A (321)	COENZYME F420-DEPENDENT N5,N10-METHYLENETETRA	**190**	34	0.027	0.28	3.32	174	6.1% : 11.5% (7/115 : 20/174)	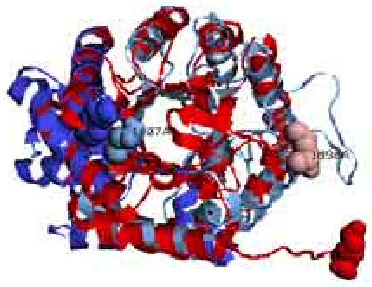
	1d9e:A (268)	3-DEOXY-D-MANNO-OCTULOSONATE 8-PHOSPHATE SYNT								
518	1dgl:A (237)	LECTIN	**123**	145	4E-36	0.91	1.31	225	46.5% : 46.2% (53/114 : 104/225)	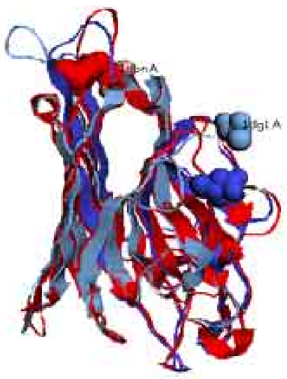
	1dbn:A (239)	PROTEIN (LEUKOAGGLUTININ)								

519	1mvq:A (236)	lectin, isoform 1	**123**	165	5E-42	0.92	1.36	225	50.9% : 50.7% (58/114 : 114/225)	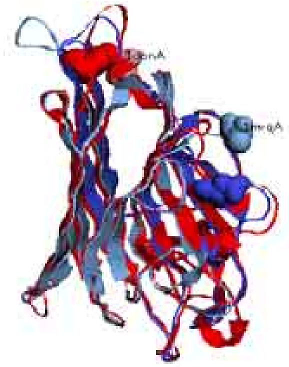
	1dbn:A (239)	PROTEIN (LEUKOAGGLUTININ)								
520	1dct:A (324)	PROTEIN (MODIFICATION METHYLASE HAEIII)	**282**	34	0.019	0.31	2.91	151	17.6% : 17.9% (22/125 : 27/151)	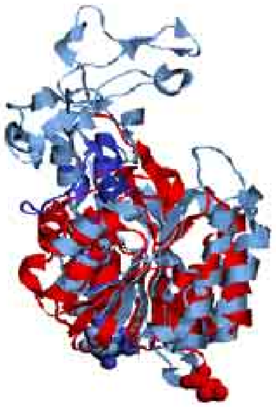
	1wy7:A (197)	hypothetical protein PH1948								
521	1df6:A (30)	CYCLOVIOLACIN O1	**26**	31	0.066	0.27	1.24	23	54.5% : 47.8% (12/22 : 11/23)	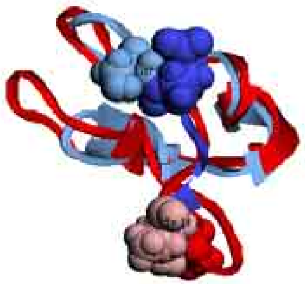
	1r1f:A (37)	Palicourein								

522	*<br>1df8:A<br>(118)<br>*<br>1swf:A<br>(117)	PROTEIN (STREPTAVIDIN)<br>CIRCULARLY PERMUTED CORE-STREPTAVIDIN E51/A46	38	196	8E-52	0.73	0.67	109	100.0% : 98.2%<br>(81/81 : 107/109)	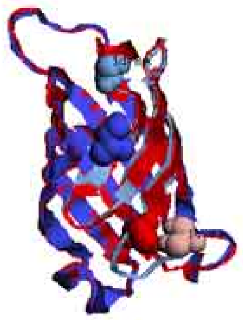
523	1dgl:A<br>(237)<br>1dhk:B<br>(197)	LECTIN<br>BEAN LECTIN-LIKE INHIBITOR	123	55	8E-09	0.63	1.78	176	39.8% : 39.8%<br>(39/98 : 70/176)	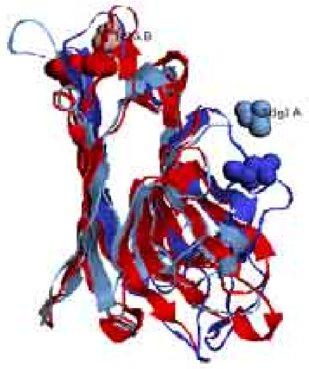
524	1dgl:A<br>(237)<br>1dzq:A<br>(235)	LECTIN<br>ULEX EUROPAEUS LECTIN II (UEA-II)	123	142	3E-35	0.89	1.44	220	42.1% : 42.3%<br>(48/114 : 93/220)	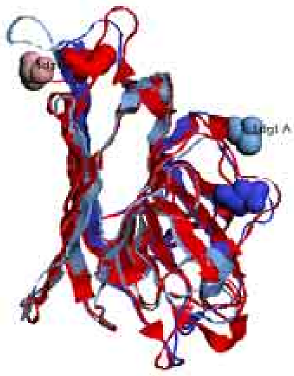

525	1dgl:A (237)	LECTIN	**124**	124	9E-30	0.83	1.51	214	46.8% : 44.9% (52/111 : 96/214)	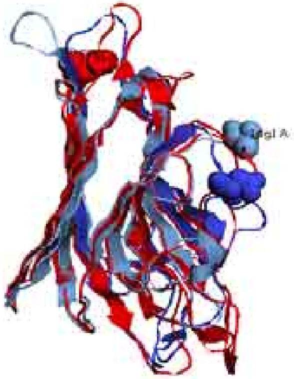
	1f9k:A (234)	ACIDIC LECTIN								
526	1dgl:A (237)	LECTIN	**123**	144	7E-36	0.86	1.45	217	51.3% : 45.6% (58/113 : 99/217)	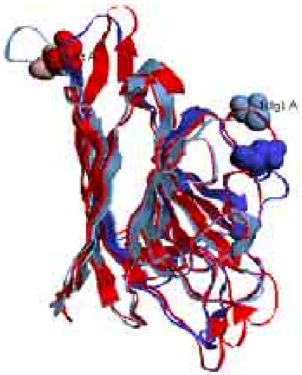
	1fat:A (233)	PHYTOHEMAGGLUTININ-L								
527	1dgl:A (237)	LECTIN	**123**	154	1E-38	0.91	1.29	224	45.2% : 46.0% (52/115 : 103/224)	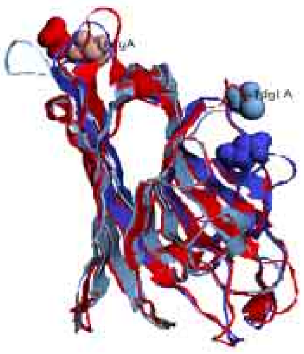
	1fny:A (235)	BARK AGGLUTININ I,POLYPEPTIDE A								

528	1dgl:A (237)	LECTIN	123	132	3E-32	0.82	1.71	215	41.3% : 40.0% (45/109 : 86/215)	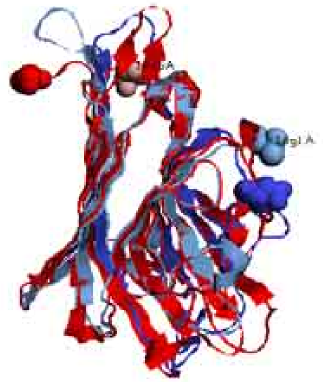
	1fx5:A (241)	ANTI-H(O) LECTIN I								
529	1g7y:A (252)	STEM/LEAF LECTIN DB58	116	141	1E-34	0.83	1.45	224	44.7% : 41.5% (51/114 : 93/224)	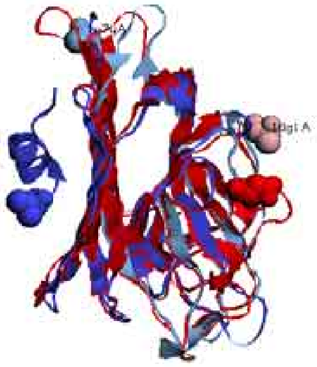
	1dgl:A (237)	LECTIN								
530	1g9f:A (242)	LECTIN	114	141	9E-35	0.83	1.35	219	44.1% : 44.7% (49/111 : 98/219)	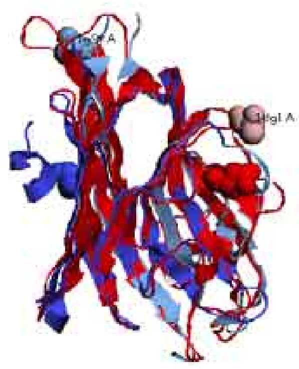
	1dgl:A (237)	LECTIN								

531	1gbg:_ (214)	(1,3-1,4)-BETA-D-GLUCAN 4 GLUCANOHYDROLASE	**108**	33	0.021	0.36	3.18	140	11.5% : 10.0% (7/61 : 14/140)	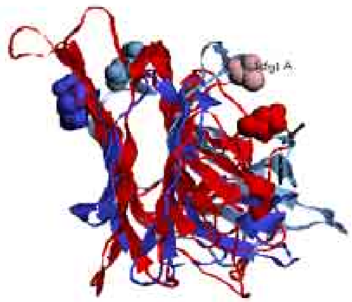
	1dgl:A (237)	LECTIN								
532	1dgl:A (237)	LECTIN	**125**	135	6E-33	0.84	1.53	215	45.5% : 43.7% (51/112 : 94/215)	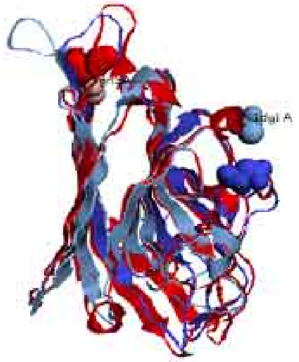
	1gnz:A (236)	LECTIN								
533	1gsl:_ (243)	GRIFFONIA SIMPLICIFOLIA LECTIN 4	**122**	148	5E-37	0.85	1.50	219	44.6% : 41.6% (50/112 : 91/219)	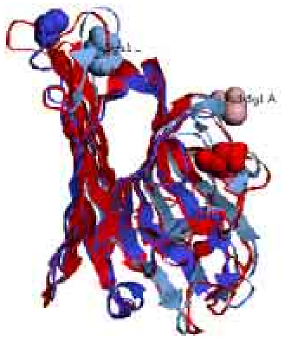
	1dgl:A (237)	LECTIN								

534	1dgl:A (237)	LECTIN	**115**	44	1E-05	0.50	2.06	163	18.0% : 16.6% (16/89 : 27/163)	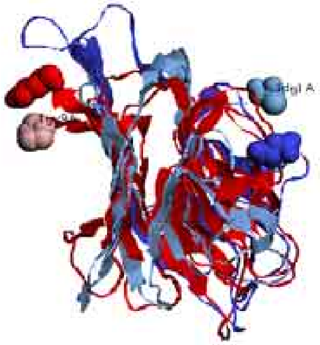
	1gv9:A (224)	P58/ERGIC-53								
535	1dgl:A (237)	LECTIN	**123**	121	7E-29	0.85	1.48	214	34.3% : 34.6% (35/102 : 74/214)	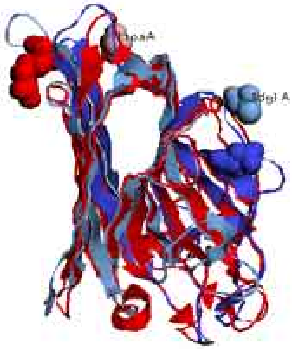
	1ioa:A (228)	ARCELIN-5A								
536	1is3:A (134)	CONGERIN II	**38**	36	0.002	0.30	2.07	94	7.6% : 10.6% (5/66 : 10/94)	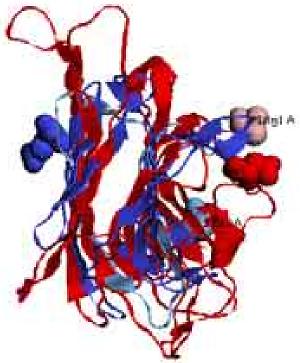
	1dgl:A (237)	LECTIN								

537	1dgl:A (237)<br>1kgy:A (181)	LECTIN<br>EPHRIN TYPE-B RECEPTOR 2	**118**	31	0.099	0.23	3.25	102	10.9% : 4.9% (6/55 : 5/102)	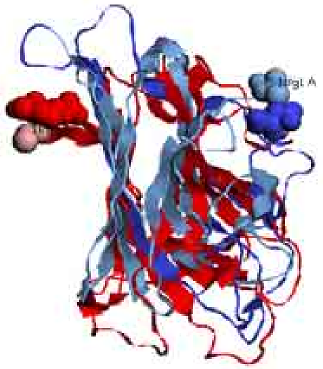
538	1n3o:A (241)<br>1dgl:A (237)	lectin PAL<br>LECTIN	**121**	168	5E-43	0.90	1.34	225	46.1% : 49.8% (53/115 : 112/225)	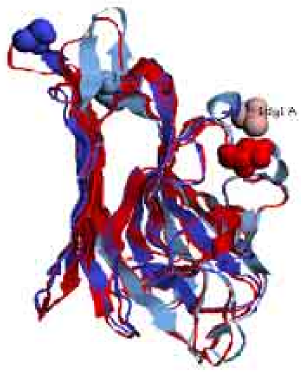
539	1dgl:A (237)<br>1n47:A (233)	LECTIN<br>Isolectin B4	**123**	148	5E-37	0.91	1.19	223	47.7% : 46.2% (53/111 : 103/223)	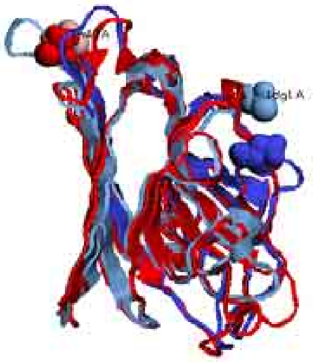

540	1dgl:A (237)	LECTIN	116	35	0.01	0.35	2.71	134	20.3% : 7.5% (12/59 : 10/134)	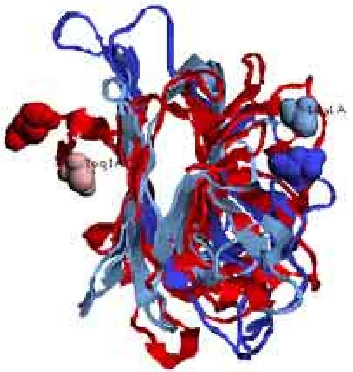
	1oq1:A (223)	Protein yesU								
541	1sla:A (134)	BOVINE GALECTIN-1	36	35	0.004	0.29	2.14	92	16.7% : 12.0% (11/66 : 11/92)	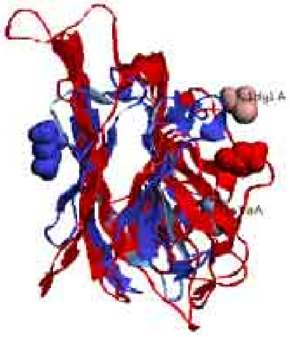
	1dgl:A (237)	LECTIN								
542	1vav:A (222)	Alginate lyase PA1167	105	39	0.0004	0.28	3.42	123	6.2% : 8.1% (4/65 : 10/123)	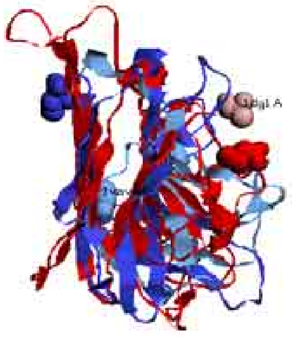
	1dgl:A (237)	LECTIN								

543	1wbf:A<br>(237)<br><br>1dgl:A<br>(237)	PROTEIN (AGGLUTININ)<br><br>LECTIN	**115**	125	3E-30	0.84	1.39	218	42.0% : 42.7%<br>(47/112 : 93/218)	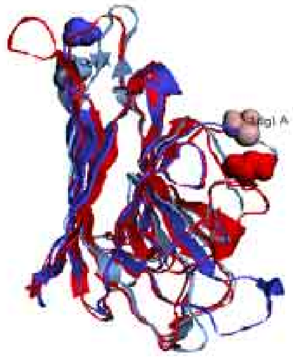
544	1dgl:A<br>(237)<br><br>2a6v:A<br>(218)	LECTIN<br><br>Emp46p	**117**	51	1E-07	0.51	2.59	162	16.5% : 16.7%<br>(14/85 : 27/162)	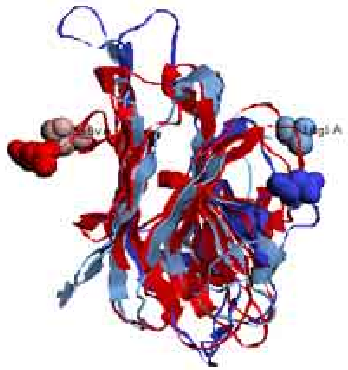
545	1dgl:A<br>(237)<br><br>2a6y:A<br>(232)	LECTIN<br><br>Emp47p (form1)	**117**	45	6E-06	0.43	2.14	156	16.9% : 18.6%<br>(14/83 : 29/156)	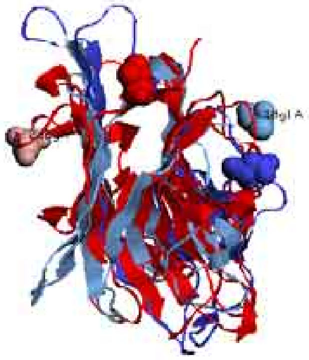

546	1dgl:A (237)	LECTIN	123	115	4E-27	0.76	1.42	169	42.2% : 41.4% (43/102 : 70/169)	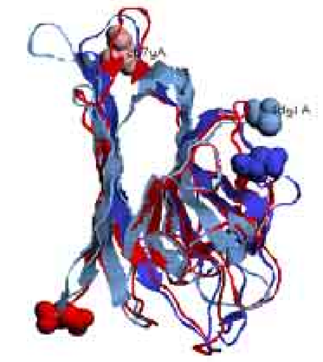
	2b7y:A (181)	Favin beta chain								
547	1dgl:A (237)	LECTIN	123	216	2E-57	0.96	0.71	228	62.9% : 60.5% (73/116 : 138/228)	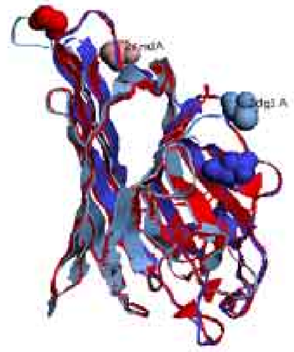
	2fmd:A (233)	Lectin								
548	1mvq:A (236)	lectin, isoform 1	123	61	9E-11	0.64	1.70	176	41.8% : 43.2% (41/98 : 76/176)	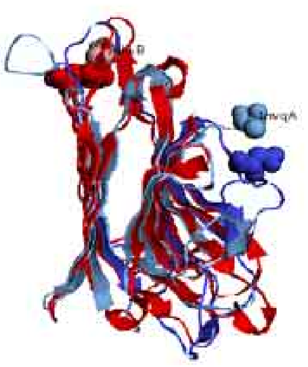
	1dhk:B (197)	BEAN LECTIN-LIKE INHIBITOR								

549	1dhk:B (197)	BEAN LECTIN-LIKE INHIBITOR	**44**	35	0.006	0.20	2.89	108	18.3% : 18.5% (17/93 : 20/108)	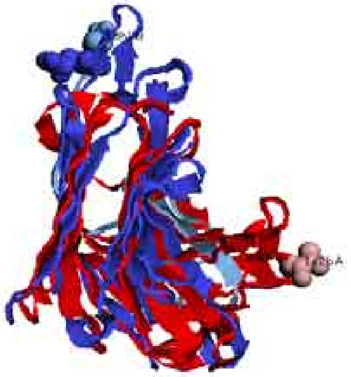
	1s2b:A (201)	Scytalidopepsin B								
550	1dil:_ (381)	SIALIDASE	**235**	38	0.002	0.28	2.80	187	11.5% : 10.7% (20/174 : 20/187)	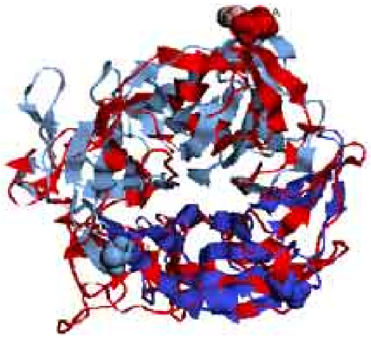
	2g8s:A (347)	Glucose/sorbosone dehydrogenases								
551	1dl3:A (194)	PROTEIN (PHOSPHORIBOSYLANTRANILATE ISOMERASE)	**54**	46	3E-06	0.39	2.80	148	14.1% : 10.8% (20/142 : 16/148)	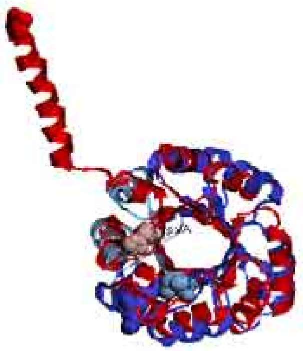
	1vpx:A (208)	PROTEIN (Transaldolase (EC 2.2.1.2))								

552	1dlb:A (64)	HIV-1 ENVELOPE GLYCOPROTEIN GP41	**31**	41	6E-05	0.40	2.44	53	6.5% : 7.5% (2/31 : 4/53)	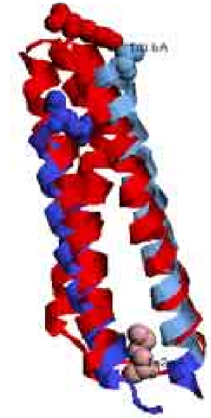
	1e2a:A (102)	ENZYME IIA								
553	1dps:A (159)	DPS	**47**	34	0.01	0.39	3.20	116	9.9% : 8.6% (9/91 : 10/116)	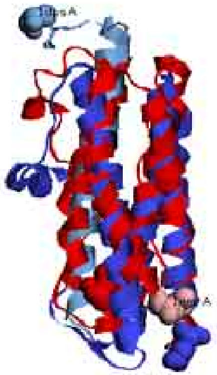
	1emr:A (159)	LEUKEMIA INHIBITORY FACTOR								
554	1f6f:A (185)	PLACENTAL LACTOGEN	**137**	34	0.014	0.33	3.27	110	7.9% : 9.1% (6/76 : 10/110)	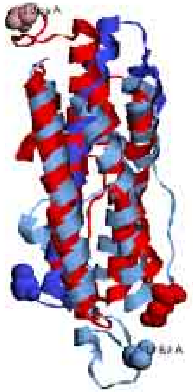
	1dps:A (159)	DPS								

555	1i1r:B (167)	VIRAL IL-6	**121**	34	0.011	0.36	3.02	109	5.9% : 11.0% (3/51 : 12/109)	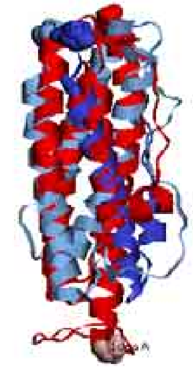
	1dps:A (159)	DPS								
556	1dps:A (159)	DPS	**47**	36	0.002	0.31	3.21	95	5.2% : 5.3% (3/58 : 5/95)	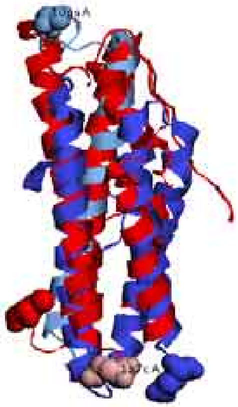
	1z7c:A (165)	Chorionic somatomammotropin hormone								
557	1sr4:A (167)	Cytolethal distending toxin subunit A	**67**	38	0.0004	0.32	1.54	103	7.3% : 23.3% (7/96 : 24/103)	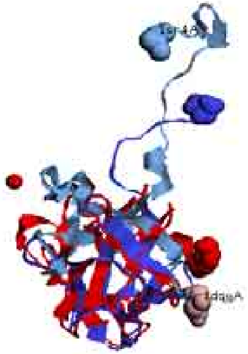
	1dqg:A (134)	MANNOSE RECEPTOR								

558	2brq:A (94)	FILAMIN A	**42**	35	0.005	0.34	3.04	66	14.0% : 12.1% (8/57 : 8/66)	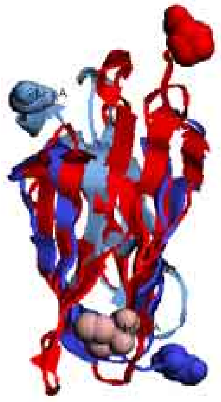
	1dqt:A (117)	CYTOTOXIC T LYMPHOCYTE ASSOCIATED ANTIGEN 4								
559	1ds6:A (181)	RAS-RELATED C3 BOTULINUM TOXIN SUBSTRATE 2	**60**	37	0.001	0.20	2.61	121	11.4% : 17.4% (10/88 : 21/121)	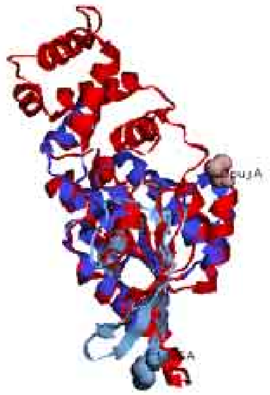
	1puj:A (262)	conserved hypothetical protein ylqF								
560	1duc:_ (118)	DEOXYURIDINE 5'-TRIPHOSPHATE NUCLEODITOHYDROL	**50**	33	0.012	0.21	3.24	65	7.7% : 12.3% (4/52 : 8/65)	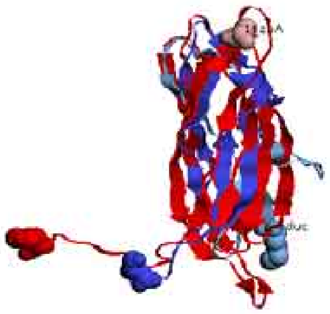
	1tza:A (132)	apaG protein								

561	1eg2:A (271)	MODIFICATION METHYLASE RSRI	**156**	37	0.002	0.37	3.03	134	14.1% : 15.7% (11/78 : 21/134)	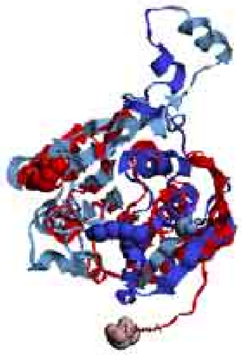
	1dus:A (194)	MJ0882								
562	1g60:A (239)	Adenine-specific Methyltransferase MboIIA	**144**	43	3E-05	0.34	2.60	120	13.4% : 15.8% (9/67 : 19/120)	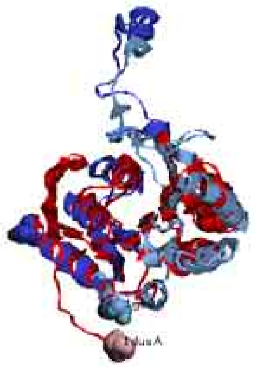
	1dus:A (194)	MJ0882								
563	1dxe:A (253)	2-DEHYDRO-3-DEOXY-GALACTARATE ALDOLASE	**151**	37	0.002	0.38	2.46	149	11.8% : 11.4% (17/144 : 17/149)	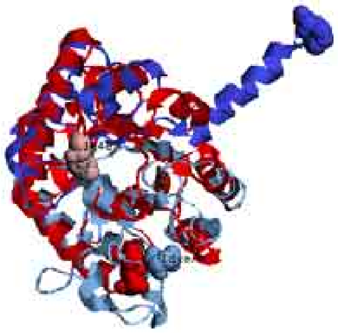
	1g4e:A (221)	THIAMIN PHOSPHATE SYNTHASE								

564	1dxe:A (253)	2-DEHYDRO-3-DEOXY-GALACTARATE ALDOLASE	130	34	0.012	0.46	2.52	155	6.5% : 15.5% (10/154 : 24/155)	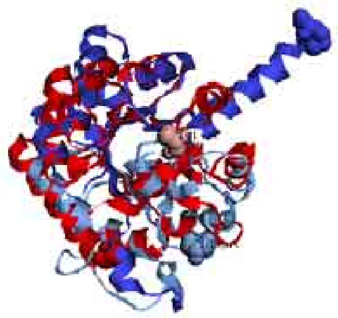
	1vlw:A (205)	2-dehydro-3-deoxyphosphogluconate aldolase/4-								
565	1j27:A (98)	hypothetical protein TT1725	53	33	0.021	0.24	1.65	66	6.7% : 9.1% (4/60 : 6/66)	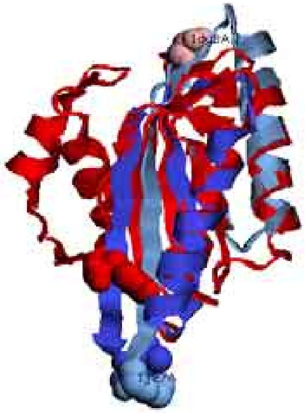
	1dy3:A (158)	7,8-DIHYDRO-6-HYDROXYMETHYLPTERIN-PYROPHOSPHO								
566	2fhm:A (91)	Probable acylphosphatase	37	30	0.09	0.23	2.94	65	5.2% : 10.8% (3/58 : 7/65)	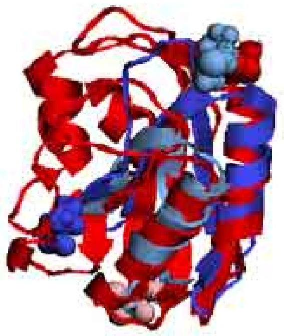
	1dy3:A (158)	7,8-DIHYDRO-6-HYDROXYMETHYLPTERIN-PYROPHOSPHO								

567	* 1dyp:A (271)	KAPPA-CARRAGEENASE	**78**	75	7E-15	0.36	2.10	174	18.2% : 20.1% (24/132 : 35/174)	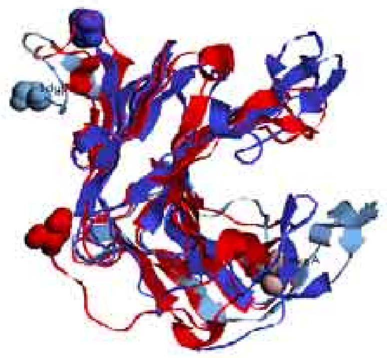
	* 1mve:A (243)	Truncated 1,3-1,4-beta-D-glucanase								
568	* 1dzb:A (223)	SCFV FRAGMENT 1F9	**117**	185	4E-48	0.95	1.06	215	26.0% : 62.8% (47/181 : 135/215)	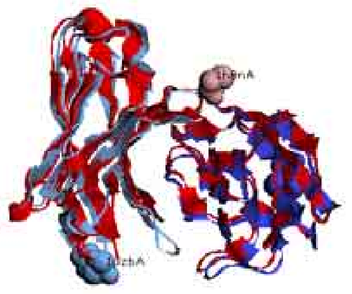
	* 1h8n:A (218)	MUTANT AL2 6E7S9G								
569	1jp5:A (233)	single-chain Fv fragment 1696	**114**	160	1E-40	0.88	1.14	214	28.2% : 51.4% (55/195 : 110/214)	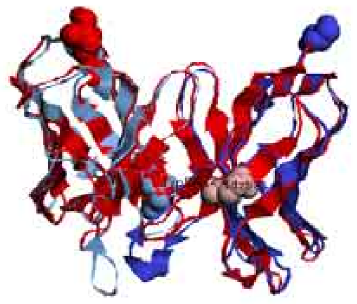
	1dzb:A (223)	SCFV FRAGMENT 1F9								

570	1dzb:A (223)	SCFV FRAGMENT 1F9	**117**	169	2E-43	0.88	1.68	214	22.1% : 48.1% (42/190 : 103/214)	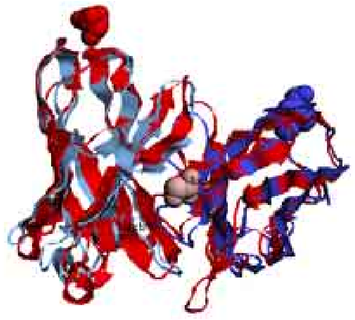
	1x9q:A (232)	4m5.3 anti-fluorescein single chain antibody								
571	1dzb:A (223)	SCFV FRAGMENT 1F9	**116**	173	1E-44	0.87	1.45	216	26.7% : 56.5% (51/191 : 122/216)	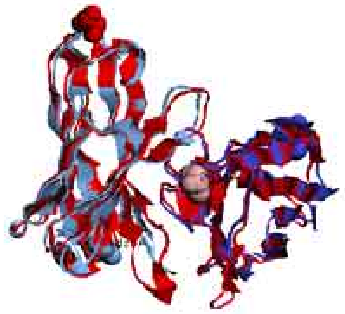
	2gjj:A (237)	A21 single-chain antibody fragment against er								
572	1mvq:A (236)	lectin, isoform 1	**123**	147	1E-36	0.89	1.42	219	46.5% : 46.1% (53/114 : 101/219)	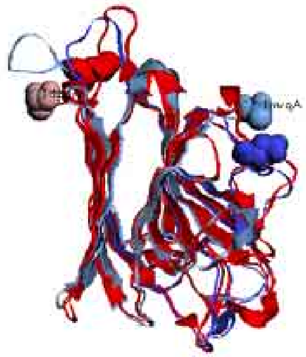
	1dzq:A (235)	ULEX EUROPAEUS LECTIN II (UEA-II)								

573	1e0s:A (173)	ADP-RIBOSYLATION FACTOR 6	**65**	44	7E-06	0.23	3.30	125	15.3% : 16.8% (13/85 : 21/125)	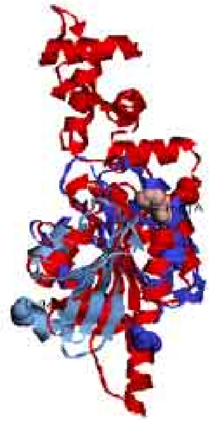
	1puj:A (262)	conserved hypothetical protein ylqF								
574	1e1a:A (312)	DIISOPROPYLFLUOROPHOSPHATASE	**135**	60	3E-10	0.33	3.49	188	12.5% : 13.8% (22/176 : 26/188)	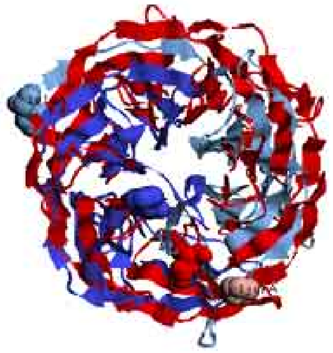
	1jof:A (365)	CARBOXY-CIS,CIS-MUCONATE CYCLASE								
575	1e1a:A (312)	DIISOPROPYLFLUOROPHOSPHATASE	**158**	48	1E-06	0.46	2.71	213	14.0% : 13.1% (25/179 : 28/213)	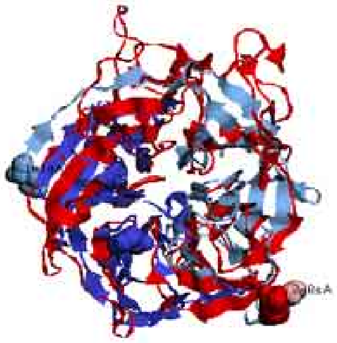
	2g8s:A (347)	Glucose/sorbosone dehydrogenases								

576	1zke:A (81)	Hypothetical protein HP1531	**46**	34	0.006	0.28	1.57	53	13.5% : 7.5% (7/52 : 4/53)	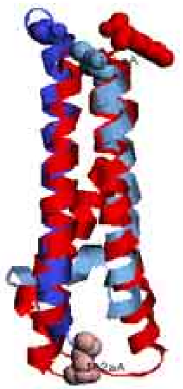
	1e2a:A (102)	ENZYME IIA								
577	2g38:A (77)	PE FAMILY PROTEIN	**42**	44	6E-06	0.27	2.36	54	9.1% : 11.1% (4/44 : 6/54)	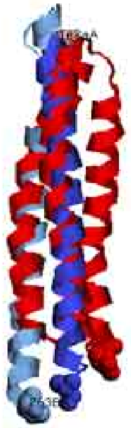
	1e2a:A (102)	ENZYME IIA								
578	2gyq:A (162)	ycfI, putative structural protein	**99**	43	2E-05	0.44	3.43	87	7.9% : 8.0% (6/76 : 7/87)	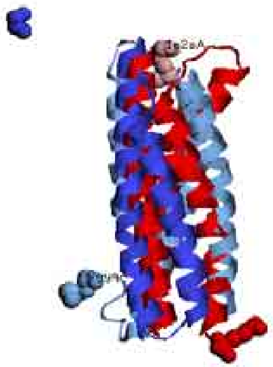
	1e2a:A (102)	ENZYME IIA								

579	*<br>1e6g:A<br>(59)<br><br>*<br>1g2b:A<br>(63)	SPECTRIN ALPHA CHAIN<br><br>SPECTRIN ALPHA CHAIN	**47**	90	9E-20	0.59	0.45	52	92.3% : 90.4%<br>(36/39 : 47/52)	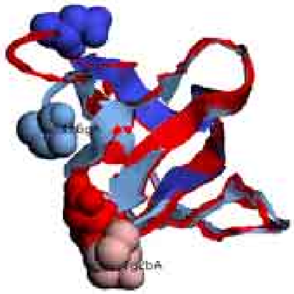
580	*<br>1e6g:A<br>(59)<br><br>*<br>1tuc_<br>(65)	SPECTRIN ALPHA CHAIN<br><br>ALPHA-SPECTRIN	**18**	90	9E-20	0.66	1.65	55	90.0% : 85.5%<br>(36/40 : 47/55)	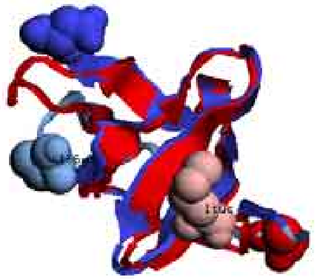
581	1ojh:A<br>(52)<br><br>1ecm:A<br>(91)	NBLA<br><br>ENDO-OXABICYCLIC TRANSITION STATE ANALOGUE	**16**	34	0.006	0.28	2.31	39	12.5% : 12.8%<br>(4/32 : 5/39)	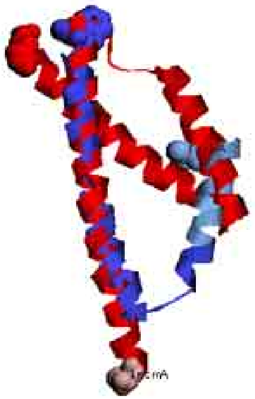

582	1eg2:A (271)	MODIFICATION METHYLASE RSRI	**135**	32	0.056	0.24	3.08	125	13.5% : 15.2% (10/74 : 19/125)	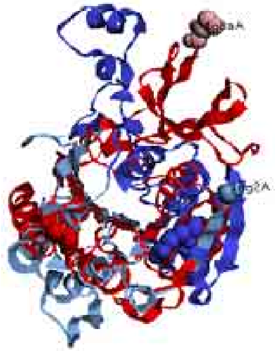
	1g8a:A (227)	FIBRILLARIN-LIKE PRE-RRNA PROCESSING PROTEIN								
583	1eg2:A (271)	MODIFICATION METHYLASE RSRI	**135**	44	1E-05	0.23	3.04	118	15.5% : 16.1% (11/71 : 19/118)	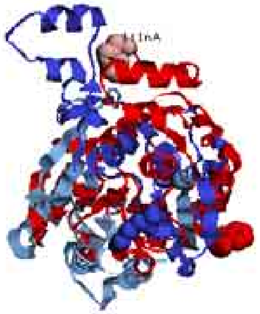
	1i1n:A (224)	PROTEIN-L-ISOASPARTATE O-METHYLTRANSFERASE								
584	1eg2:A (271)	MODIFICATION METHYLASE RSRI	**130**	52	5E-08	0.24	3.03	116	14.9% : 15.5% (11/74 : 18/116)	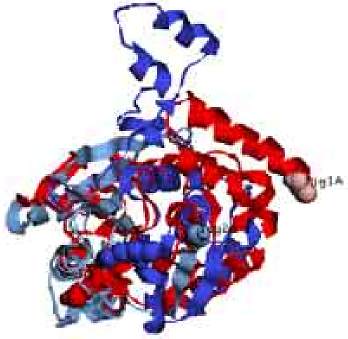
	1jg1:A (215)	protein-L-isoaspartate O-methyltransferase								

585	1eg2:A (271)	MODIFICATION METHYLASE RSRI	**187**	38	0.001	0.22	3.25	128	10.2% : 8.6% (6/59 : 11/128)	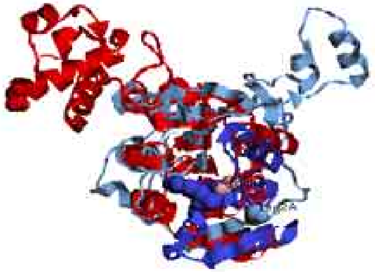
	1qyr:A (252)	High level Kasugamycin resistance protein								
586	1eg2:A (271)	MODIFICATION METHYLASE RSRI	**189**	43	3E-05	0.27	2.67	138	17.4% : 17.4% (16/92 : 24/138)	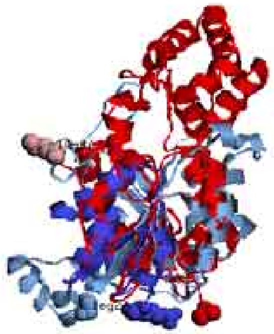
	1wg8:A (281)	predicted S-adenosylmethionine-dependent meth								
587	1eg2:A (271)	MODIFICATION METHYLASE RSRI	**163**	43	3E-05	0.36	3.48	143	10.1% : 11.2% (8/79 : 16/143)	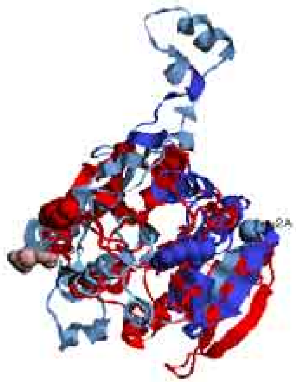
	2bzg:A (231)	THIOPURINE S-METHYLTRANSFERASE								

588	1eg2:A (271)	MODIFICATION METHYLASE RSRI	**194**	43	3E-05	0.52	2.65	131	10.4% : 17.6% (8/77 : 23/131)	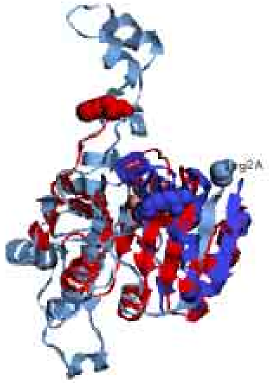
	2esr:A (160)	Methyltransferase								
589	1eg2:A (271)	MODIFICATION METHYLASE RSRI	**167**	37	0.002	0.36	2.45	117	10.8% : 17.1% (7/65 : 20/117)	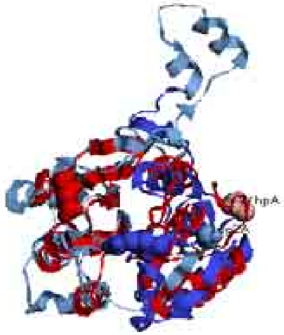
	2fhp:A (183)	methylase, putative								
590	1eg2:A (271)	MODIFICATION METHYLASE RSRI	**161**	40	0.0002	0.42	3.43	153	6.9% : 7.2% (6/87 : 11/153)	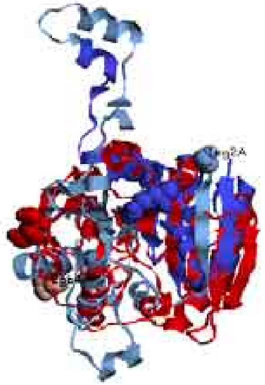
	2gb4:A (233)	Thiopurine S-methyltransferase								

591	1eg2:A (271)	MODIFICATION METHYLASE RSRI	**139**	38	0.001	0.23	3.20	142	12.1% : 13.4% (11/91 : 19/142)	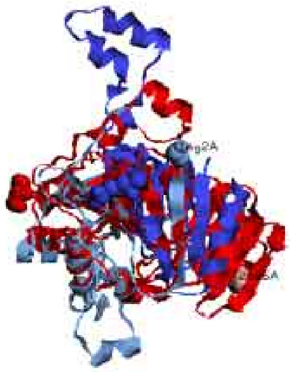
	2o05:A (286)	Spermidine synthase								
592	2a10:A (104)	Carbon dioxide concentrating mechanism protei	**35**	31	0.052	0.24	3.07	66	11.1% : 16.7% (6/54 : 11/66)	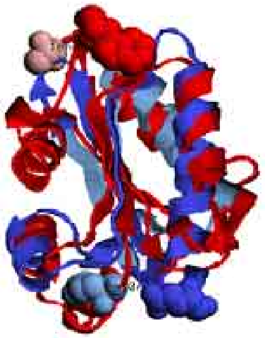
	1ehw:A (143)	NUCLEOSIDE DIPHOSPHATE KINASE								
593	1eix:A (231)	OROTIDINE 5'-MONOPHOSPHATE DECARBOXYLASE	**166**	38	0.0006	0.24	3.08	147	9.8% : 11.6% (13/132 : 17/147)	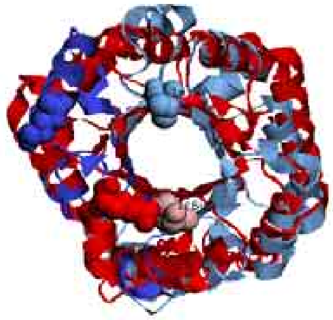
	1f6y:A (259)	5-METHYLTETRAHYDROFOLATE CORRINOID/IRON SULFU								

594	1ek0:A (170)	PROTEIN (GTP-BINDING PROTEIN YPT51)	61	45	5E-06	0.23	3.08	123	14.8% : 18.7% (13/88 : 23/123)	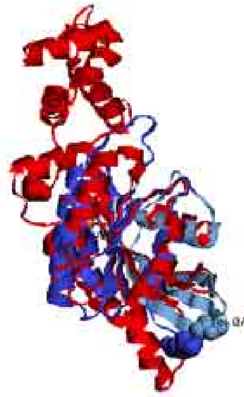
	1puj:A (262)	conserved hypothetical protein ylqF								
595	1nps:A (87)	DEVELOPMENT-SPECIFIC PROTEIN S	47	34	0.009	0.30	1.69	80	21.9% : 28.8% (16/73 : 23/80)	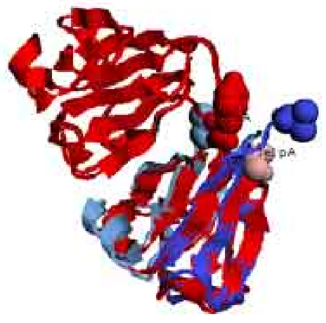
	1elp:A (174)	GAMMA-D CRYSTALLIN								
596	1emr:A (159)	LEUKEMIA INHIBITORY FACTOR	48	39	0.0003	0.36	3.43	109	4.3% : 10.1% (4/92 : 11/109)	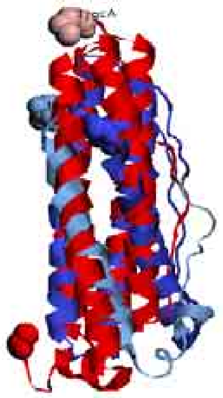
	1jgc:A (160)	bacterioferritin								

597	1emr:A (159)	LEUKEMIA INHIBITORY FACTOR	**49**	42	3E-05	0.36	3.46	115	12.2% : 11.3% (12/98 : 13/115)	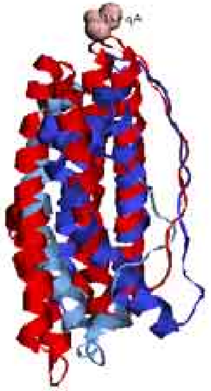
	1krq:A (164)	ferritin								
598	1emr:A (159)	LEUKEMIA INHIBITORY FACTOR	**116**	31	0.059	0.38	3.41	124	9.6% : 8.1% (9/94 : 10/124)	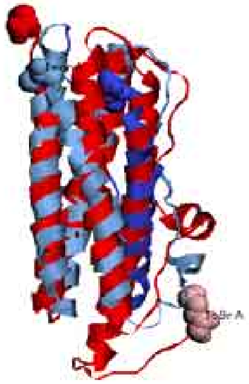
	1o9r:A (162)	AGROBACTERIUM TUMEFACIENS DPS								
599	1uvh:A (157)	STARVATION-INDUCED DNA PROTECTING PROTEIN	**45**	35	0.004	0.40	3.20	118	12.2% : 11.9% (12/98 : 14/118)	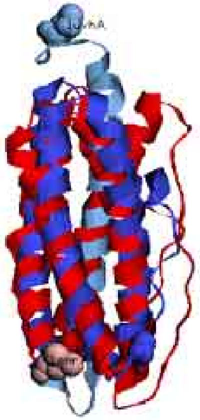
	1emr:A (159)	LEUKEMIA INHIBITORY FACTOR								

600	1emr:A (159)	LEUKEMIA INHIBITORY FACTOR	**129**	43	2E-05	0.46	3.46	114	11.9% : 12.3% (10/84 : 14/114)	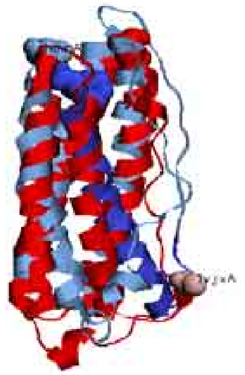
	1vjx:A (148)	putative ferritin-like diiron-carboxylate pro								
601	1yjg:A (157)	Surface protein VspA	**71**	34	0.01	0.22	2.37	81	10.3% : 4.9% (6/58 : 4/81)	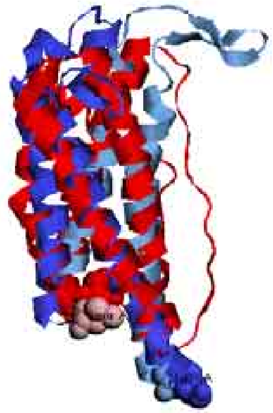
	1emr:A (159)	LEUKEMIA INHIBITORY FACTOR								
602	1emr:A (159)	LEUKEMIA INHIBITORY FACTOR	**113**	33	0.024	0.37	3.45	120	9.7% : 7.5% (9/93 : 9/120)	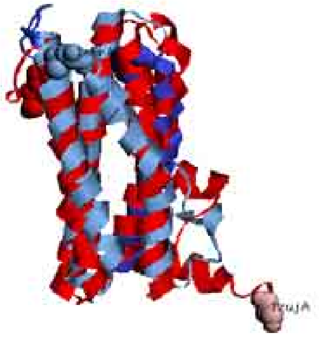
	1zuj:A (168)	hypothetical protein Llacc01001955								

603	1emr:A (159)	LEUKEMIA INHIBITORY FACTOR	**44**	31	0.084	0.26	3.07	83	3.4% : 8.4% (2/59 : 7/83)	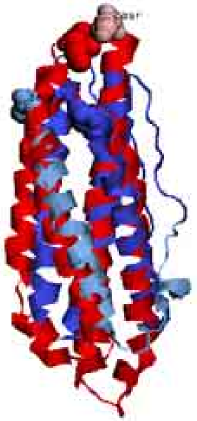
	2asr_ (142)	ASPARTATE RECEPTOR								
604	1emr:A (159)	LEUKEMIA INHIBITORY FACTOR	**54**	37	0.001	0.40	2.69	109	9.8% : 6.4% (9/92 : 7/109)	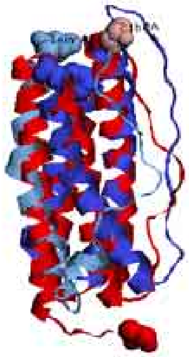
	2ib0:A (142)	CONSERVED HYPOTHETICAL ALANINE RICH PROTEIN								
605	* 1emy:_ (153)	MYOGLOBIN	**50**	36	0.002	0.31	3.35	75	11.5% : 9.3% (6/52 : 7/75)	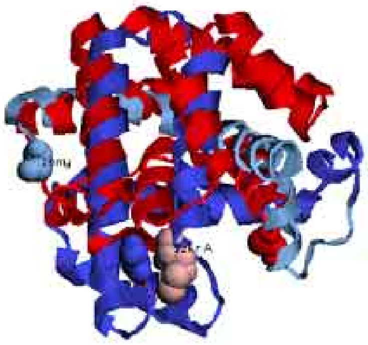
	* 1zkr:A (146)	Major allergen I polypeptide, fused chain 1,								

606	1v8e:A (217)	putative glycerophosphoryl diester phosphodie	**114**	33	0.03	0.32	2.81	130	14.5% : 13.1% (16/110 : 17/130)	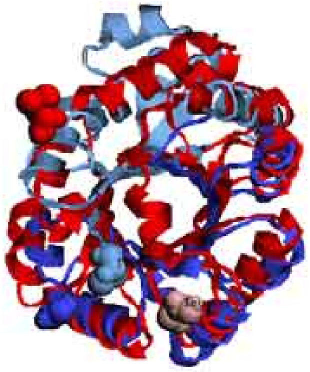
	1eua:A (213)	KDPG ALDOLASE								
607	1ggq:A (162)	OUTER SURFACE PROTEIN C	**44**	36	0.002	0.27	2.12	87	4.6% : 9.2% (3/65 : 8/87)	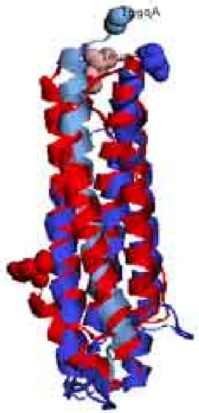
	1eum:A (161)	FERRITIN 1								
608	1i1r:B (167)	VIRAL IL-6	**133**	34	0.013	0.33	3.29	108	5.9% : 5.6% (4/68 : 6/108)	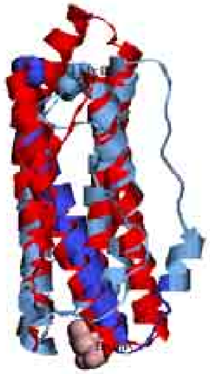
	1eum:A (161)	FERRITIN 1								

609	1yjg:A (157)	Surface protein VspA	**124**	46	2E-06	0.23	2.37	83	3.3% : 4.8% (2/60 : 4/83)	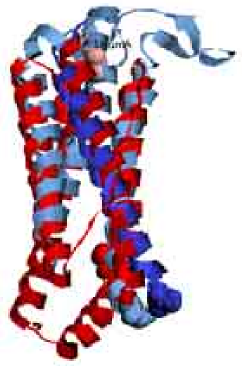
	1eum:A (161)	FERRITIN 1								
610	1evs:A (164)	ONCOSTATIN M	**137**	32	0.03	0.37	3.32	109	4.6% : 6.4% (4/87 : 7/109)	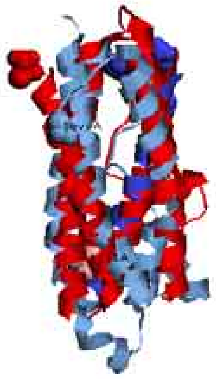
	1ji4:A (144)	NEUTROPHIL-ACTIVATING PROTEIN A								
611	1evs:A (164)	ONCOSTATIN M	**140**	39	0.0003	0.32	3.37	103	7.7% : 6.8% (4/52 : 7/103)	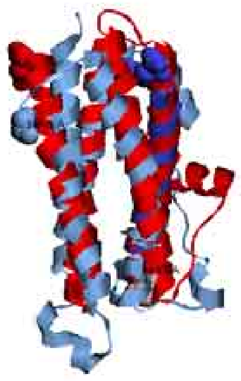
	1ji5:A (142)	Dlp-1								

612	1evs:A (164)	ONCOSTATIN M	**136**	38	0.0004	0.39	3.46	110	7.1% : 6.4% (6/85 : 7/110)	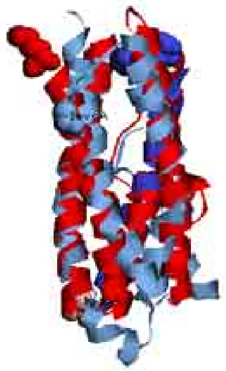
	1jig:A (146)	Dlp-2								
613	1evs:A (164)	ONCOSTATIN M	**53**	35	0.004	0.20	2.86	80	14.3% : 13.8% (8/56 : 11/80)	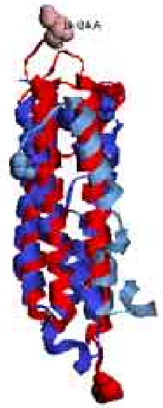
	1k04:A (142)	FOCAL ADHESION KINASE 1								
614	1evs:A (164)	ONCOSTATIN M	**139**	40	0.0002	0.29	3.42	102	8.7% : 9.8% (8/92 : 10/102)	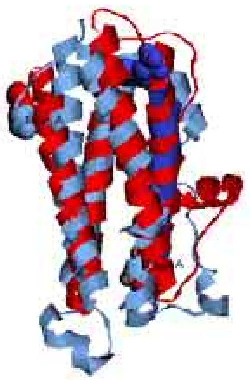
	1qgh:A (150)	NON-HEME IRON-CONTAINING FERRITIN								

615	1evs:A (164)	ONCOSTATIN M	138	39	0.0003	0.32	3.10	104	7.1% : 6.7% (6/85 : 7/104)	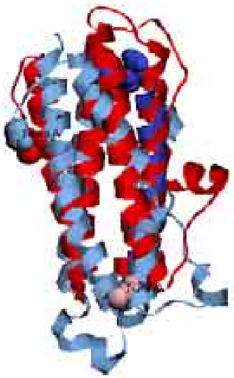
	1umn:A (151)	DPS-LIKE PEROXIDE RESISTANCE PROTEIN								
616	1evs:A (164)	ONCOSTATIN M	135	31	0.061	0.29	2.74	100	17.2% : 9.0% (10/58 : 9/100)	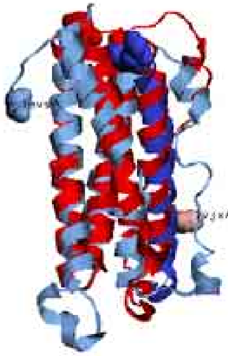
	1vjx:A (148)	putative ferritin-like diiron-carboxylate pro								
617	1evs:A (164)	ONCOSTATIN M	111	43	2E-05	0.26	3.43	98	3.5% : 12.2% (2/57 : 12/98)	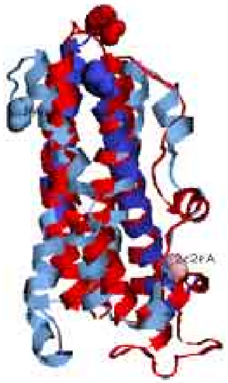
	2c2f:A (178)	DNA-BINDING STRESS RESPONSE PROTEIN								

618	1evs:A<br>(164)<br>2c41:A<br>(155)	ONCOSTATIN M<br>DPS FAMILY DNA-BINDING STRESS RESPONSE PROTEI	**134**	40	0.0002	0.41	2.69	117	9.4% : 8.5%<br>(9/96 : 10/117)	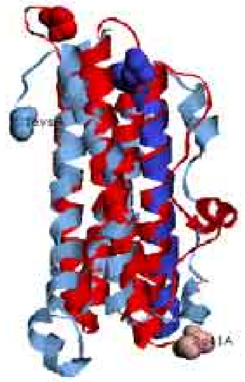
619	1evs:A<br>(164)<br>2clb:A<br>(169)	ONCOSTATIN M<br>DPS-LIKE PROTEIN	**119**	36	0.002	0.26	3.42	98	8.5% : 8.2%<br>(7/82 : 8/98)	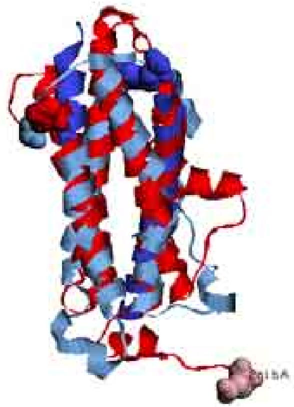
620	1evs:A<br>(164)<br>2d5k:A<br>(148)	ONCOSTATIN M<br>Dps family protein	**134**	36	0.002	0.40	2.82	114	7.0% : 5.3%<br>(6/86 : 6/114)	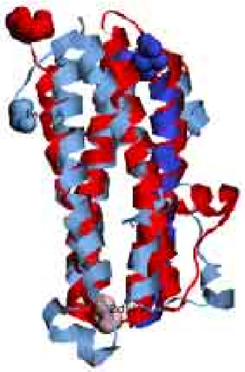

621	1evs:A (164)	ONCOSTATIN M	**127**	36	0.002	0.24	3.19	97	11.4% : 10.3% (9/79 : 10/97)	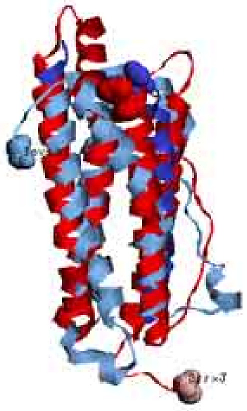
	2ffx:J (173)	ferritin light chain								
622	1evs:A (164)	ONCOSTATIN M	**133**	37	0.0008	0.39	3.36	111	7.3% : 6.3% (6/82 : 7/111)	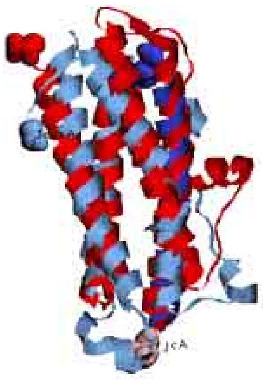
	2fjc:A (151)	Antigen TpF1								
623	1eye:A (257)	DIHYDROPTEROATE SYNTHASE I	**111**	33	0.044	0.27	2.56	147	12.1% : 15.6% (17/141 : 23/147)	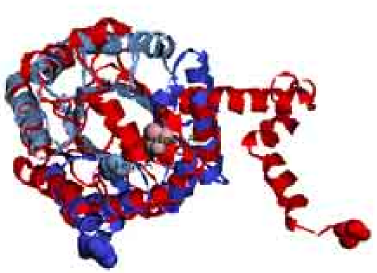
	1m1b:A (291)	PHOSPHOENOLPYRUVATE PHOSPHOMUTASE								

624	1eye:A (257)<br>1o66:A (250)	DIHYDROPTEROATE SYNTHASE I<br>3-methyl-2-oxobutanoate hydroxymethyltransfer	**128**	34	0.021	0.26	3.34	126	19.8% : 11.9% (20/101 : 15/126)	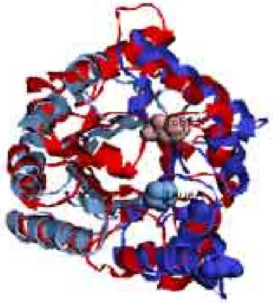
625	1eye:A (257)<br>1pyf:A (311)	DIHYDROPTEROATE SYNTHASE I<br>IolS protein	**185**	35	0.007	0.23	3.40	155	9.6% : 14.8% (13/136 : 23/155)	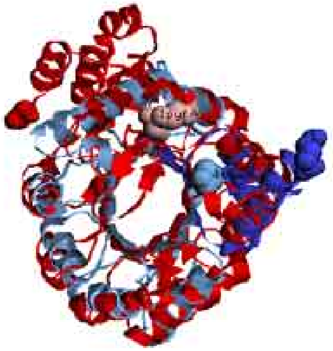
626	1f07:A (321)<br>1o60:A (270)	COENZYME F420-DEPENDENT N5,N10-METHYLENETETRA<br>2-dehydro-3-deoxyphosphooctonate aldolase	**191**	33	0.056	0.28	3.40	171	8.5% : 9.9% (10/118 : 17/171)	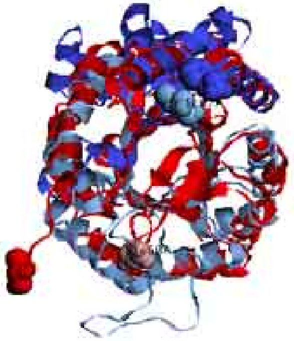

627	1f1m:A<br>(162)<br>1jgc:A<br>(160)	OUTER SURFACE PROTEIN C<br>bacterioferritin	**128**	37	0.001	0.21	1.91	82	7.3% : 11.0%<br>(4/55 : 9/82)	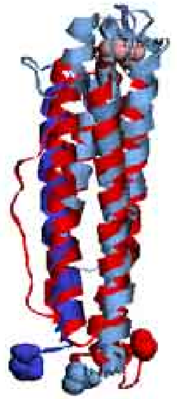
628	1f1m:A<br>(162)<br>1krq:A<br>(164)	OUTER SURFACE PROTEIN C<br>ferritin	**128**	36	0.003	0.20	2.22	82	4.7% : 6.1%<br>(3/64 : 5/82)	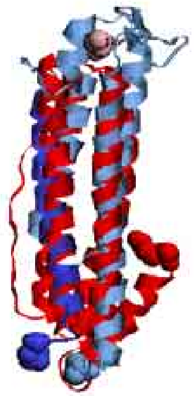
629	1f1m:A<br>(162)<br>1mfr:A<br>(171)	OUTER SURFACE PROTEIN C<br>M FERRITIN	**37**	41	9E-05	0.23	2.23	84	12.7% : 6.0%<br>(10/79 : 5/84)	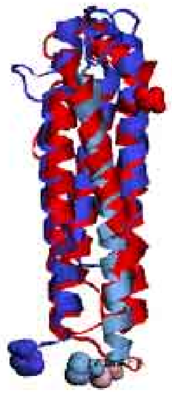

630	1f1m:A (162)	OUTER SURFACE PROTEIN C	**38**	38	0.0004	0.24	2.21	84	11.5% : 8.3% (7/61 : 7/84)	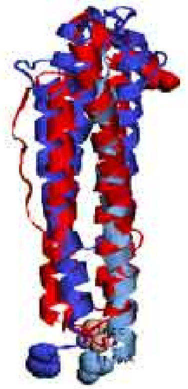
	1rcc_ (171)	L FERRITIN								
631	1f1m:A (162)	OUTER SURFACE PROTEIN C	**99**	40	0.0002	0.36	3.07	100	16.7% : 10.0% (8/48 : 10/100)	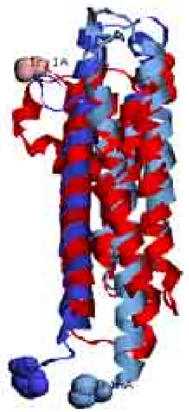
	1rj1:A (148)	invertase inhibitor								
632	1f1m:A (162)	OUTER SURFACE PROTEIN C	**128**	34	0.012	0.23	1.83	83	6.6% : 6.0% (4/61 : 5/83)	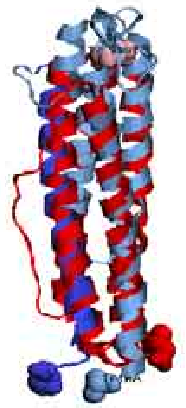
	1sof:A (155)	Bacterioferritin								

633	1f1m:A (162)	OUTER SURFACE PROTEIN C	**127**	36	0.002	0.21	2.30	82	1.9% : 9.8% (1/52 : 8/82)	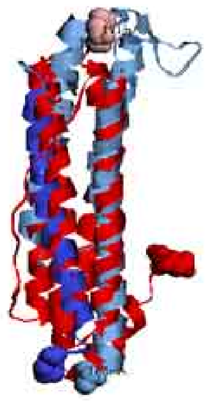
	1vlg:A (164)	ferritin								
634	1g60:A (239)	Adenine-specific Methyltransferase MboIIA	**163**	44	2E-05	0.41	2.09	127	11.0% : 17.3% (9/82 : 22/127)	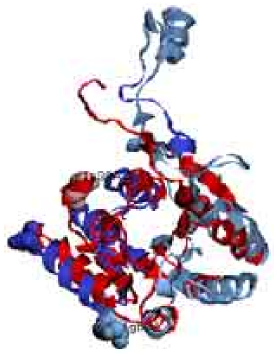
	1f38:A (186)	PRECORRIN-8W DECARBOXYLASE								
635	* 1h8n:A (218)	MUTANT AL2 6E7S9G	**108**	101	8E-23	0.80	2.01	208	24.6% : 51.4% (43/175 : 107/208)	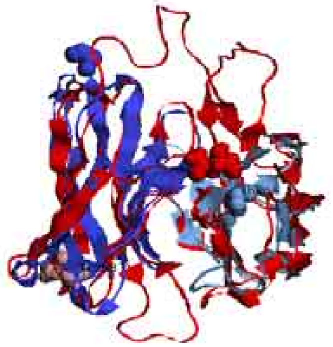
	* 1f3r:B (257)	FV ANTIBODY FRAGMENT								

636	1f3r:B (257)	FV ANTIBODY FRAGMENT	**139**	102	4E-23	0.78	2.17	214	28.3% : 44.4% (49/173 : 95/214)	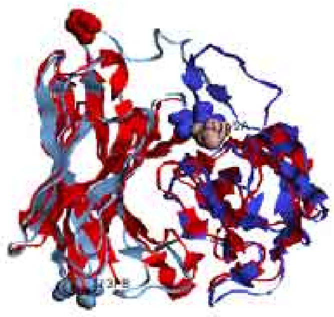
	1jp5:A (233)	single-chain Fv fragment 1696								
637	1f3r:B (257)	FV ANTIBODY FRAGMENT	**138**	88	7E-19	0.72	2.38	205	23.2% : 48.3% (41/177 : 99/205)	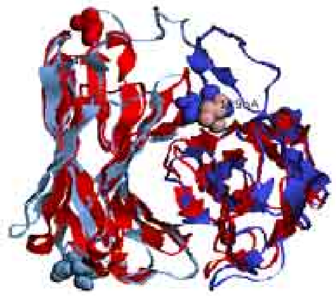
	1x9q:A (232)	4m5.3 anti-fluorescein single chain antibody								
638	1f3r:B (257)	FV ANTIBODY FRAGMENT	**137**	104	1E-23	0.73	2.09	209	23.7% : 49.8% (42/177 : 104/209)	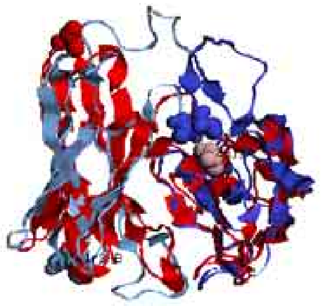
	2gjj:A (237)	A21 single-chain antibody fragment against er								

639	1f45:B (137)	INTERLEUKIN-12 ALPHA CHAIN	**85**	33	0.021	0.27	3.44	79	3.4% : 7.6% (2/59 : 6/79)	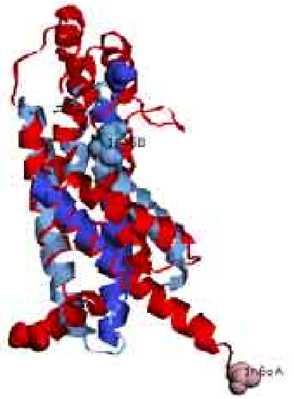
	1h6o:A (197)	TELOMERIC REPEAT BINDING FACTOR 1								
640	1f45:B (137)	INTERLEUKIN-12 ALPHA CHAIN	**101**	36	0.001	0.29	2.98	90	7.4% : 11.1% (4/54 : 10/90)	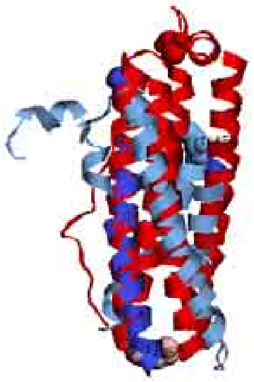
	1jgc:A (160)	bacterioferritin								
641	1f45:B (137)	INTERLEUKIN-12 ALPHA CHAIN	**108**	33	0.015	0.34	2.84	91	11.1% : 8.8% (6/54 : 8/91)	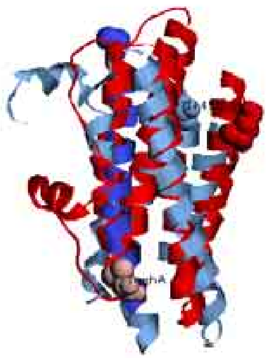
	1qgh:A (150)	NON-HEME IRON-CONTAINING FERRITIN								

642	1f45:B (137)<br>1vjx:A (148)	INTERLEUKIN-12 ALPHA CHAIN<br>putative ferritin-like diiron-carboxylate pro	**107**	36	0.001	0.35	3.34	96	4.3% : 11.5%<br>(2/47 : 11/96)	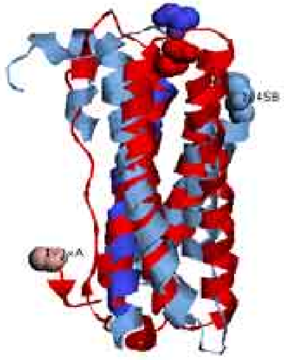
643	1f45:B (137)<br>2c41:A (155)	INTERLEUKIN-12 ALPHA CHAIN<br>DPS FAMILY DNA-BINDING STRESS RESPONSE PROTEI	**103**	33	0.018	0.36	2.79	95	9.6% : 8.4%<br>(5/52 : 8/95)	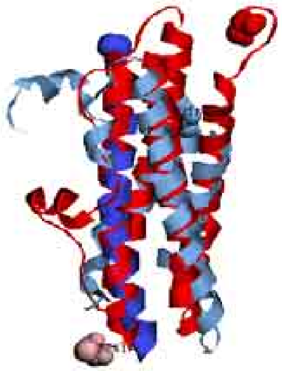
644	1f4m:A (56)<br>1wrd:A (98)	ROP ALA2ILE2-6<br>Target of Myb protein 1	**25**	38	0.0004	0.36	1.76	47	7.0% : 4.3%<br>(3/43 : 2/47)	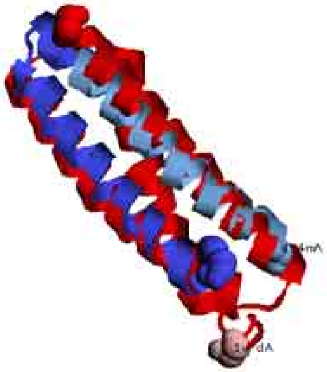

645	1f4m:A (56)	ROP ALA2ILE2-6	**24**	42	2E-05	0.50	2.48	45	16.7% : 13.3% (5/30 : 6/45)	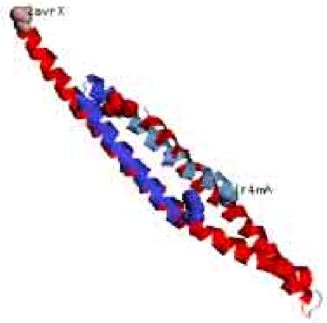
	2avr:X (109)	adhesion A								
646	1f51:E (119)	SPORULATION INITIATION PHOSPHOTRANSFERASE F	**40**	34	0.007	0.20	3.34	84	11.1% : 16.7% (9/81 : 14/84)	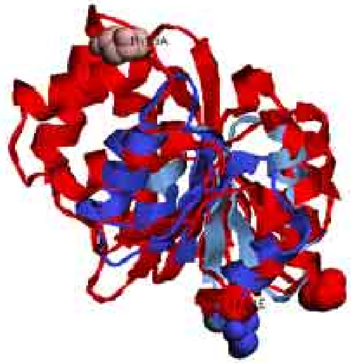
	1h1d:A (215)	CATECHOL-O-METHYLTRANSFERASE								
647	1fha:_ (173)	FERRITIN	**109**	35	0.006	0.30	3.20	116	8.0% : 6.9% (6/75 : 8/116)	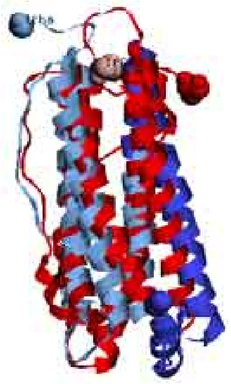
	1f6f:A (185)	PLACENTAL LACTOGEN								

648	1h96:A (167)	FERRITIN LIGHT CHAIN 1	**117**	44	1E-05	0.33	3.32	122	13.0% : 8.2% (7/54 : 10/122)	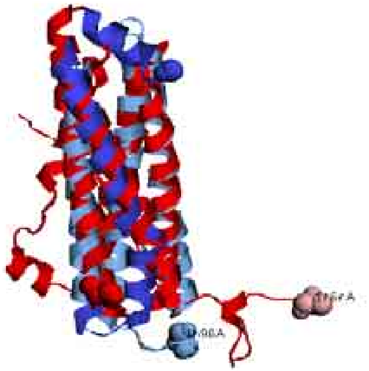
	1f6f:A (185)	PLACENTAL LACTOGEN								
649	1jgc:A (160)	bacterioferritin	**105**	46	2E-06	0.33	3.18	116	11.1% : 7.8% (6/54 : 9/116)	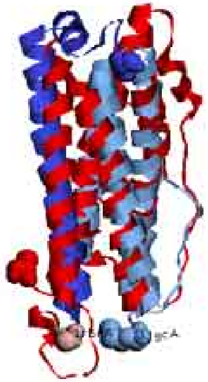
	1f6f:A (185)	PLACENTAL LACTOGEN								
650	1f6f:A (185)	PLACENTAL LACTOGEN	**155**	38	0.0008	0.31	3.05	107	3.5% : 8.4% (3/86 : 9/107)	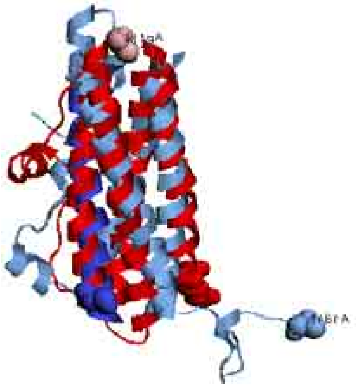
	1jig:A (146)	Dlp-2								

651	1f6f:A (185)	PLACENTAL LACTOGEN	**131**	33	0.021	0.29	2.61	115	9.0% : 11.3% (7/78 : 13/115)	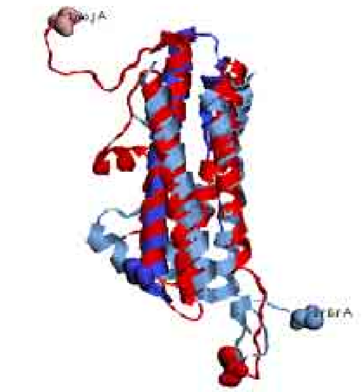
	1moj:A (180)	Dps-like ferritin								
652	1f6f:A (185)	PLACENTAL LACTOGEN	**152**	37	0.001	0.35	3.04	111	10.7% : 14.4% (6/56 : 16/111)	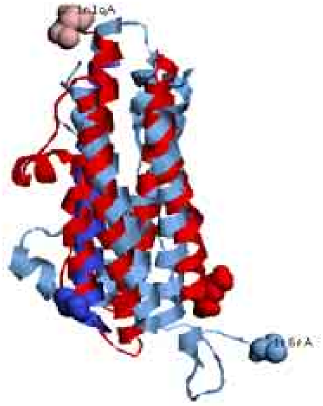
	1n1q:A (149)	DPS Protein								
653	1nf4:A (169)	bacterioferritin	**106**	39	0.0004	0.29	2.85	114	5.6% : 7.9% (4/71 : 9/114)	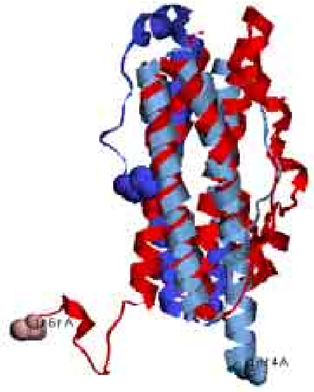
	1f6f:A (185)	PLACENTAL LACTOGEN								

654	1f6f:A<br>(185)<br>1o9r:A<br>(162)	PLACENTAL LACTOGEN<br>AGROBACTERIUM TUMEFACIENS DPS	**142**	33	0.03	0.34	3.40	113	6.8% : 8.0%<br>(4/59 : 9/113)	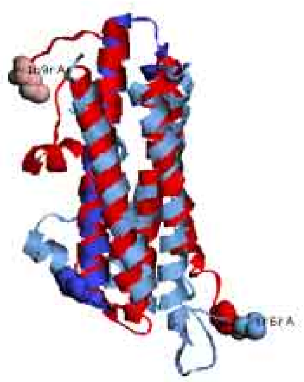
655	1f6f:A<br>(185)<br>1qgh:A<br>(150)	PLACENTAL LACTOGEN<br>NON-HEME IRON-CONTAINING FERRITIN	**158**	32	0.061	0.27	3.17	106	3.9% : 5.7%<br>(2/51 : 6/106)	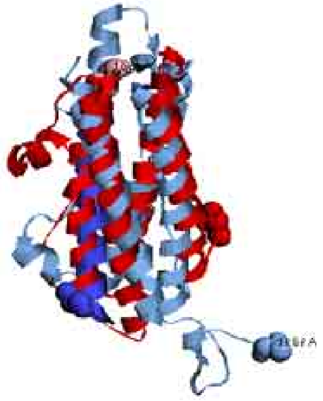
656	1rcc:_<br>(171)<br>1f6f:A<br>(185)	L FERRITIN<br>PLACENTAL LACTOGEN	**116**	42	4E-05	0.31	3.09	121	7.1% : 7.4%<br>(7/98 : 9/121)	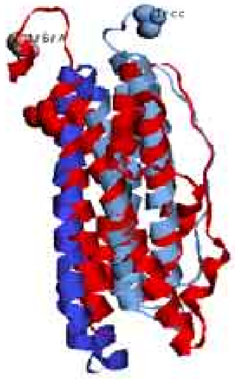

657	1sof:A (155)	Bacterioferritin	100	42	4E-05	0.33	3.07	113	4.3% : 4.4% (4/94 : 5/113)	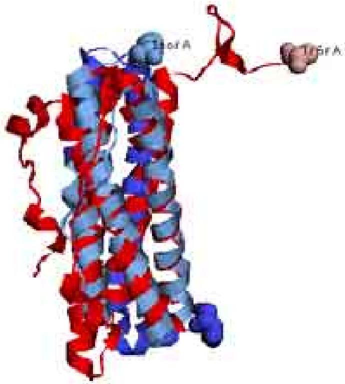
	1f6f:A (185)	PLACENTAL LACTOGEN								
658	1vlg:A (164)	ferritin	106	38	0.0006	0.31	3.29	116	10.6% : 4.3% (10/94 : 5/116)	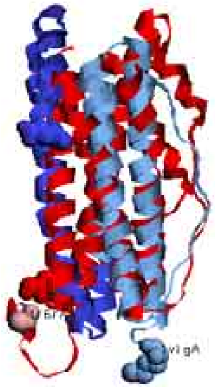
	1f6f:A (185)	PLACENTAL LACTOGEN								
659	1f6f:A (185)	PLACENTAL LACTOGEN	135	31	0.073	0.29	2.89	113	5.4% : 6.2% (3/56 : 7/113)	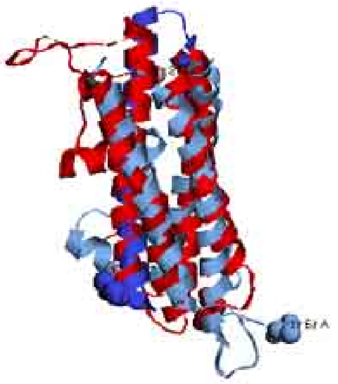
	2c2f:A (178)	DNA-BINDING STRESS RESPONSE PROTEIN								

660	1f6f:A (185)<br>2fjc:A (151)	PLACENTAL LACTOGEN<br>Antigen TpF1	**150**	35	0.007	0.36	3.01	112	9.8% : 8.0% (5/51 : 9/112)	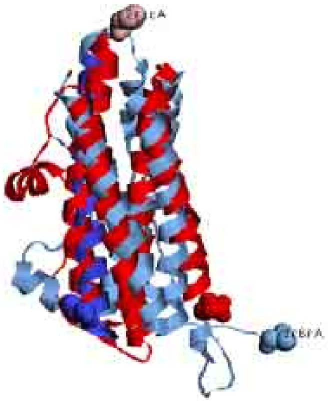
661	2fzf:A (158)<br>1f6f:A (185)	hypothetical protein<br>PLACENTAL LACTOGEN	**108**	36	0.002	0.35	2.56	117	8.3% : 10.3% (5/60 : 12/117)	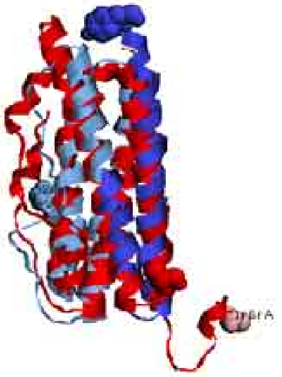
662	2ib0:A (142)<br>1f6f:A (185)	CONSERVED HYPOTHETICAL ALANINE RICH PROTEIN<br>PLACENTAL LACTOGEN	**85**	36	0.002	0.29	3.15	100	6.1% : 12.0% (3/49 : 12/100)	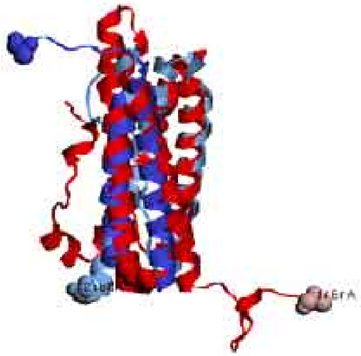

663	1v93:A<br>(292)	5,10-Methylenetetrahydrofolate reductase	**231**	34	0.021	0.22	3.26	156	14.2% : 12.2%<br>(22/155 : 19/156)	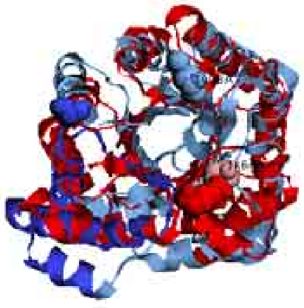
	1f6y:A<br>(259)	5-METHYLTETRAHYDROFOLATE CORRINOID/IRON SULFU								
664	1z69:A<br>(327)	Coenzyme F420-dependent N(5),N(10)-methylenet	**240**	38	0.001	0.26	2.91	161	10.5% : 8.1%<br>(16/153 : 13/161)	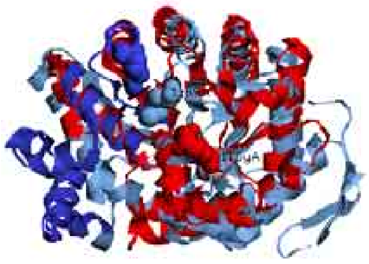
	1f6y:A<br>(259)	5-METHYLTETRAHYDROFOLATE CORRINOID/IRON SULFU								
665	1mvq:A<br>(236)	lectin, isoform 1	**124**	130	1E-31	0.80	1.34	210	48.2% : 44.8%<br>(53/110 : 94/210)	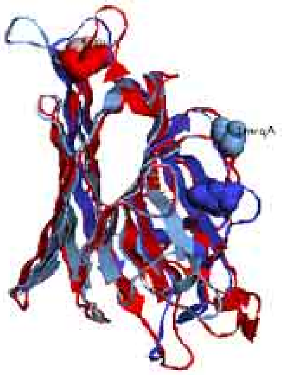
	1f9k:A<br>(234)	ACIDIC LECTIN								

666	1mvq:A (236)	lectin, isoform 1	**123**	142	4E-35	0.86	1.39	216	52.6% : 48.6% (60/114 : 105/216)	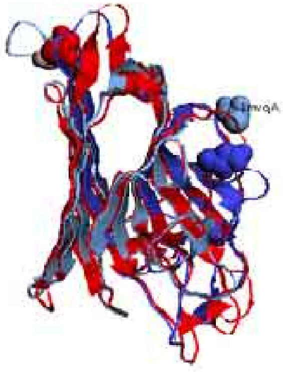
	1fat:A (233)	PHYTOHEMAGGLUTININ-L								
667	1fha:_ (173)	FERRITIN	**80**	55	5E-09	0.25	3.43	89	7.3% : 9.0% (6/82 : 8/89)	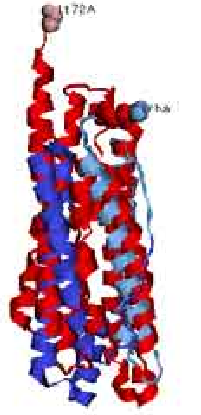
	1t72:A (215)	Phosphate transport system protein phoU homol								
668	1fha:_ (173)	FERRITIN	**94**	40	0.0001	0.36	2.43	98	8.4% : 6.1% (8/95 : 6/98)	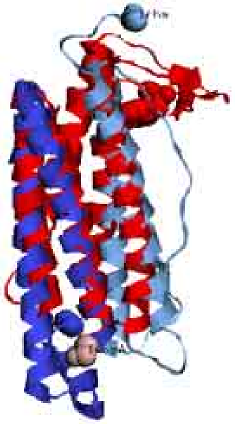
	1woz:A (159)	177aa long conserved hypothetical protein (ST								

669	1fha:_ (173)	FERRITIN	**43**	40	0.0002	0.21	2.94	82	5.9% : 7.3% (4/68 : 6/82)	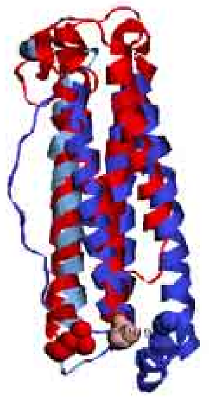
	1yjg:A (157)	Surface protein VspA								
670	2g2d:A (160)	ATP:cobalamin adenosyltransferase	**85**	34	0.012	0.36	2.38	98	5.3% : 12.2% (5/94 : 12/98)	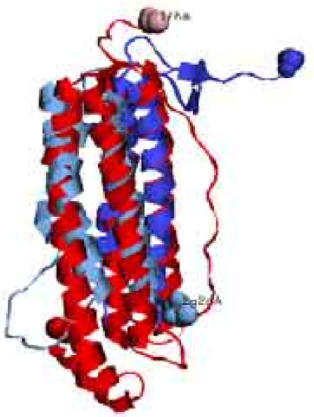
	1fha_ (173)	FERRITIN								
671	1fio:A (191)	SSO1 PROTEIN	**159**	47	2E-06	0.35	3.40	119	3.3% : 9.2% (3/90 : 11/119)	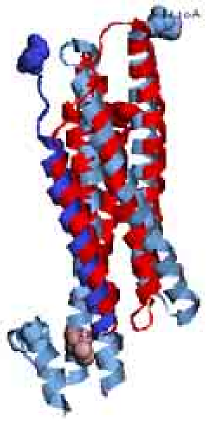
	1sj7:A (167)	Talin 1								

672	2caz:A (58)	SUPPRESSOR PROTEIN STP22 OF TEMPERATURE-SENSI	**29**	34	0.007	0.31	1.73	48	13.3% : 4.2% (6/45 : 2/48)	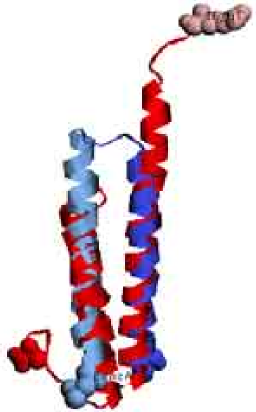
	1fjg:T (99)	30S RIBOSOMAL PROTEIN S20								
673	2caz:C (64)	PROTEIN SRN2	**37**	36	0.002	0.26	1.46	46	9.4% : 6.5% (3/32 : 3/46)	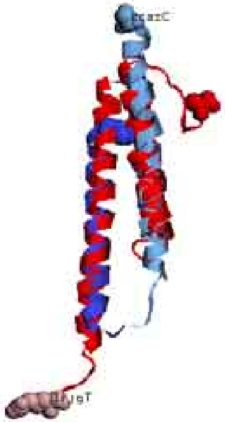
	1fjg:T (99)	30S RIBOSOMAL PROTEIN S20								
674	1xjc:A (146)	MobB protein homolog	**119**	37	0.0008	0.22	3.33	79	18.6% : 13.9% (11/59 : 11/79)	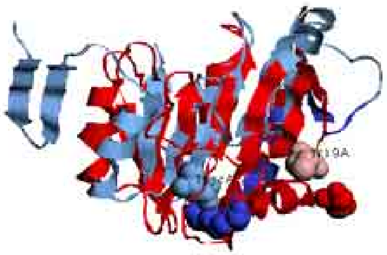
	1fl9:A (157)	HYPOTHETICAL PROTEIN HI0065								

675	1y5e:A (157)	Molybdenum cofactor biosynthesis protein B	**79**	30	0.098	0.31	3.48	85	21.1% : 10.6% (16/76 : 9/85)	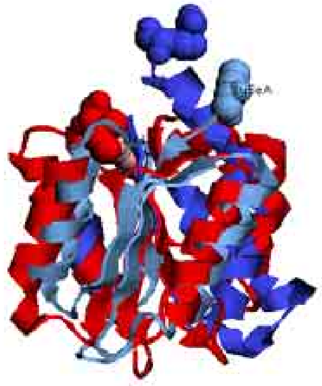
	1fla_ (138)	FLAVODOXIN								
676	1mvq:A (236)	lectin, isoform 1	**123**	161	5E-41	0.90	1.27	223	48.2% : 48.0% (55/114 : 107/223)	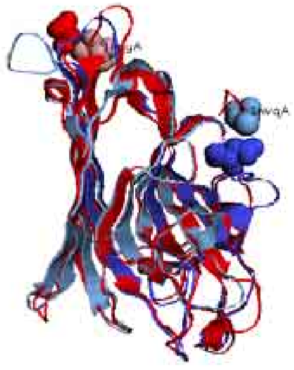
	1fny:A (235)	BARK AGGLUTININ I,POLYPEPTIDE A								
677	1zld:A (103)	Ptr necrosis toxin	**65**	37	0.001	0.32	2.94	66	20.0% : 12.1% (11/55 : 8/66)	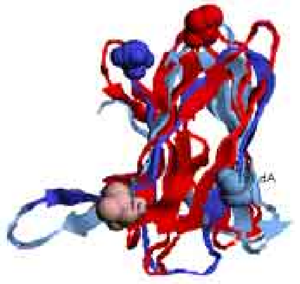
	1fo0:B (116)	PROTEIN (BM3.3 T CELL RECEPTOR BETA-CHAIN)								

678	1fp3:A (402)	N-ACYL-D-GLUCOSAMINE 2-EPIMERASE	**322**	35	0.014	0.21	3.42	245	11.7% : 13.5% (21/179 : 33/245)	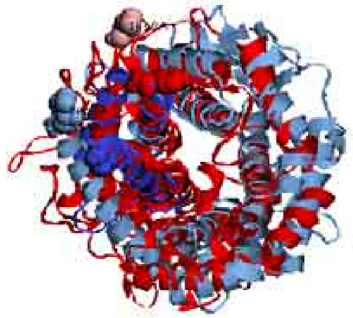
	1ks8:A (433)	Endo-b-1,4-glucanase								
679	1fp3:A (402)	N-ACYL-D-GLUCOSAMINE 2-EPIMERASE	**81**	69	8E-13	0.30	3.27	287	11.4% : 12.5% (30/263 : 36/287)	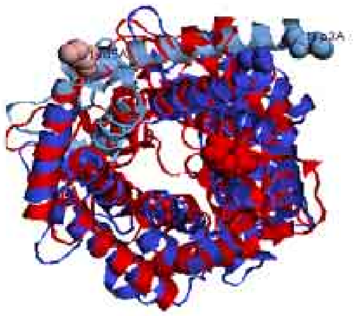
	1vd5:A (377)	unsaturated glucuronyl hydrolase								
680	1fp3:A (402)	N-ACYL-D-GLUCOSAMINE 2-EPIMERASE	**123**	62	1E-10	0.36	3.01	239	9.7% : 7.5% (22/226 : 18/239)	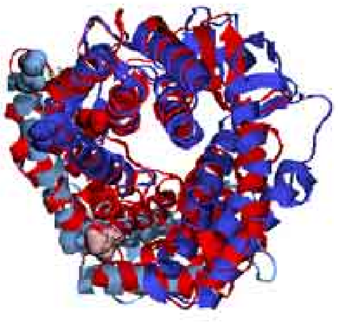
	1wu4:A (375)	xylanase Y								

681	* 1fw8:A (415)	PHOSPHOGLYCERATE KINASE	**348**	305	6E-84	0.54	1.81	410	64.2% : 62.0% (219/341 : 254/410)	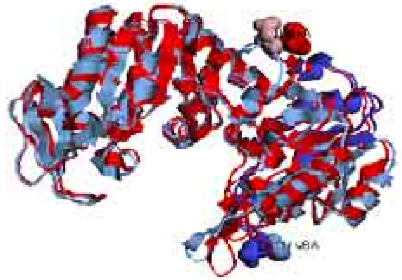
	* 1hdi:A (413)	PHOSPHOGLYCERATE KINASE								
682	* 1fw8:A (415)	PHOSPHOGLYCERATE KINASE	**342**	267	1E-72	0.55	2.26	403	64.3% : 61.5% (214/333 : 248/403)	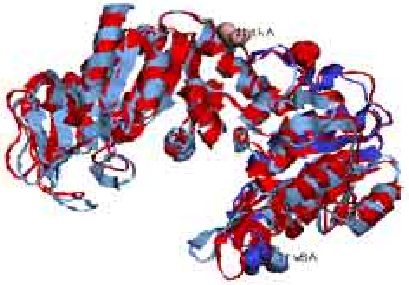
	* 1ltk:A (417)	PHOSPHOGLYCERATE KINASE								
683	* 1fw8:A (415)	PHOSPHOGLYCERATE KINASE	**347**	238	7E-64	0.53	1.67	389	50.6% : 51.2% (162/320 : 199/389)	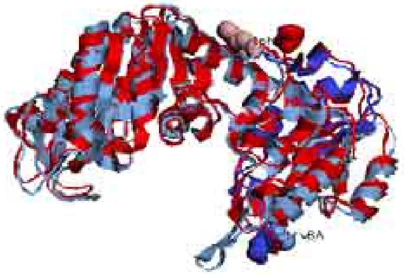
	* 1php_ (394)	3-PHOSPHOGLYCERATE KINASE								

684	* 1fw8:A (415)	PHOSPHOGLYCERATE KINASE	345	318	4E-88	0.56	2.37	413	100.0% : 99.5% (341/341 : 411/413)	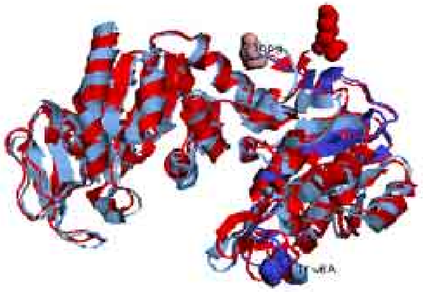
	* 1qpg_ (415)	3-PHOSPHOGLYCERATE KINASE								
685	* 1fw8:A (415)	PHOSPHOGLYCERATE KINASE	349	191	8E-50	0.50	1.40	376	41.8% : 42.3% (130/311 : 159/376)	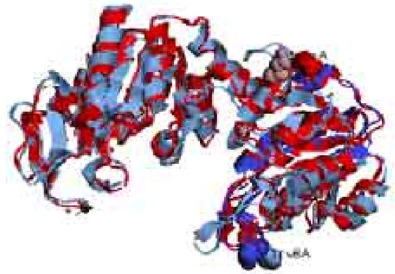
	* 1v6s:A (390)	Phosphoglycerate kinase								
686	* 1fw8:A (415)	PHOSPHOGLYCERATE KINASE	348	168	7E-43	0.50	1.96	373	38.9% : 41.3% (119/306 : 154/373)	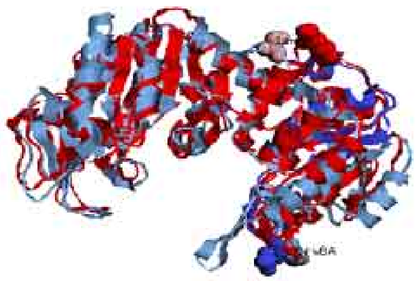
	* 1zmr:A (386)	Phosphoglycerate kinase								

687	* 1fw8:A (415)	PHOSPHOGLYCERATE KINASE	**349**	154	1E-38	0.42	3.41	355	33.8% : 34.9% (100/296 : 124/355)	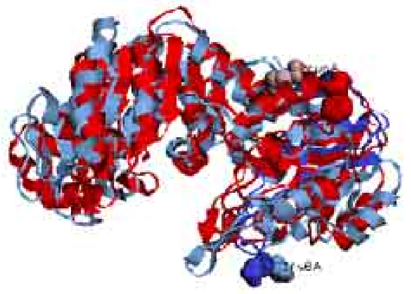
	* 2cun:A (405)	Phosphoglycerate kinase								
688	1fwn:A (257)	2-DEHYDRO-3-DEOXYPHOSPHOOCTONATE ALDOLASE	**166**	33	0.036	0.22	3.20	155	9.9% : 9.0% (13/131 : 14/155)	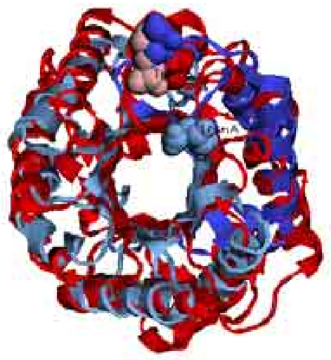
	1gzj:A (304)	ENDO TYPE CELLULASE ENGI								
689	1rhc:A (330)	F420-dependent alcohol dehydrogenase	**229**	35	0.014	0.29	2.92	172	6.9% : 14.0% (11/159 : 24/172)	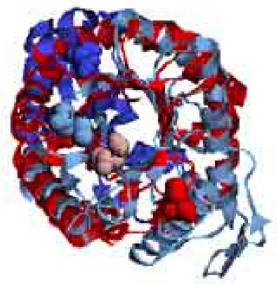
	1fwn:A (257)	2-DEHYDRO-3-DEOXYPHOSPHOOCTONATE ALDOLASE								

690	1v5x:A (200)	Phosphoribosylanthranilate isomerase	**128**	32	0.039	0.24	2.56	132	10.7% : 12.9% (14/131 : 17/132)	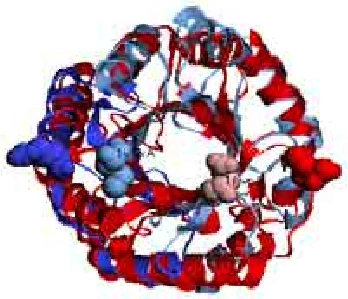
	1fwn:A (257)	2-DEHYDRO-3-DEOXYPHOSPHOOCTONATE ALDOLASE								
691	1fwn:A (257)	2-DEHYDRO-3-DEOXYPHOSPHOOCTONATE ALDOLASE	**168**	34	0.012	0.24	3.03	167	11.0% : 10.8% (12/109 : 18/167)	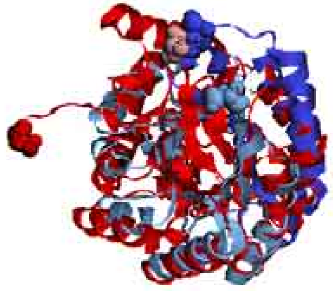
	2b4g:A (312)	dihydroorotate dehydrogenase								
692	1mvq:A (236)	lectin, isoform 1	**123**	136	2E-33	0.83	1.73	215	42.2% : 40.9% (46/109 : 88/215)	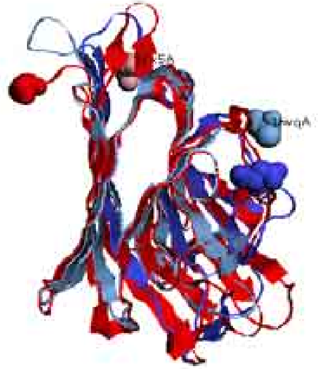
	1fx5:A (241)	ANTI-H(O) LECTIN I								

693	1fxk:A (107)	PREFOLDIN	**77**	40	0.0001	0.52	3.02	61	8.9% : 8.2% (4/45 : 5/61)	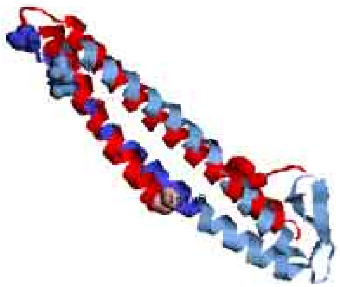
	1g4y:B (82)	CALCIUM-ACTIVATED POTASSIUM CHANNEL RSK2								
694	1fxk:A (107)	PREFOLDIN	**82**	34	0.007	0.38	2.12	52	13.7% : 15.4% (7/51 : 8/52)	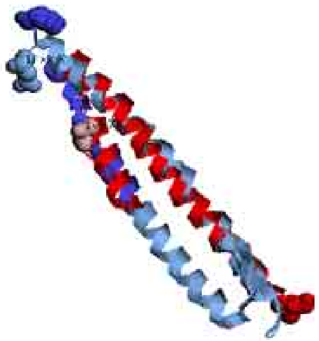
	1vp7:A (68)	exodeoxyribonuclease VII small subunit								
695	1fxk:C (133)	PROTEIN (PREFOLDIN)	**72**	32	0.037	0.24	2.39	74	2.9% : 6.8% (2/69 : 5/74)	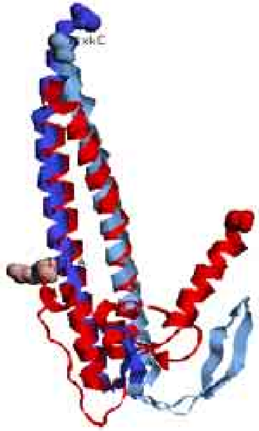
	1tjl:A (145)	DnaK suppressor protein								

696	1fxk:C (133)	PROTEIN (PREFOLDIN)	86	32	0.044	0.25	1.59	58	9.8% : 12.1% (4/41 : 7/58)	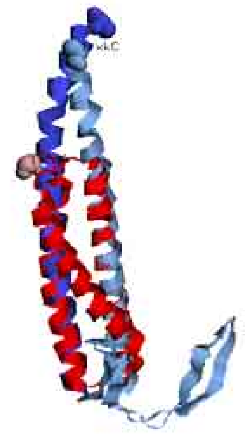
	1wrd:A (98)	Target of Myb protein 1								
697	1fxk:C (133)	PROTEIN (PREFOLDIN)	86	44	8E-06	0.52	3.14	77	8.2% : 10.4% (4/49 : 8/77)	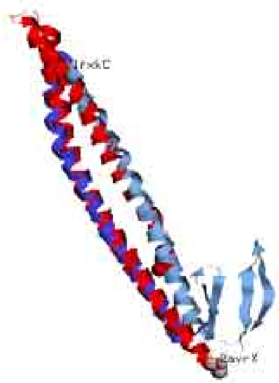
	2avr:X (109)	adhesion A								
698	1fxk:C (133)	PROTEIN (PREFOLDIN)	69	36	0.001	0.54	1.76	80	4.1% : 3.8% (3/74 : 3/80)	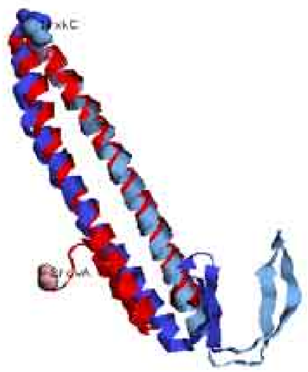
	2fcw:A (106)	Alpha-2-macroglobulin receptor-associated pro								

699	1fzq:A (176)	ADP-RIBOSYLATION FACTOR-LIKE PROTEIN 3	**69**	53	1E-08	0.23	2.58	123	16.9% : 21.1% (14/83 : 26/123)	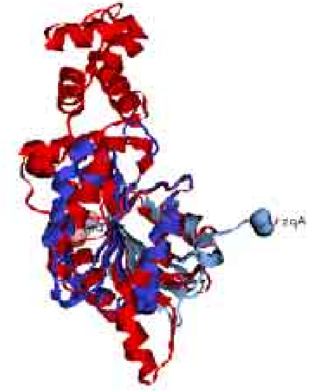
	1puj:A (262)	conserved hypothetical protein ylqF								
700	1g16:A (160)	RAS-RELATED PROTEIN SEC4	**54**	44	9E-06	0.24	2.79	119	16.5% : 23.5% (14/85 : 28/119)	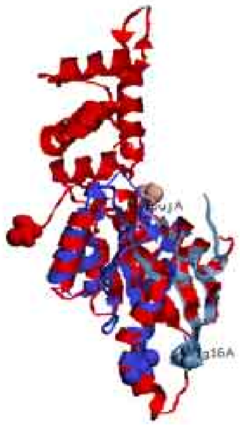
	1puj:A (262)	conserved hypothetical protein ylqF								
701	1g1k:A (143)	SCAFFOLDING PROTEIN	**61**	32	0.037	0.26	3.11	82	13.3% : 11.0% (10/75 : 9/82)	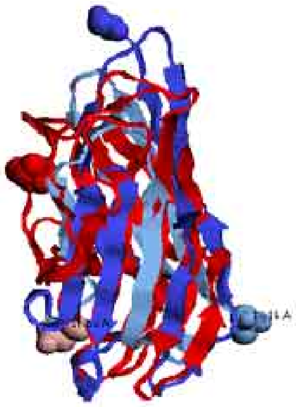
	1nbc:A (155)	CELLULOSOMAL SCAFFOLDING PROTEIN A								

702	1g1k:A (143)<br>1nqd:A (112)	SCAFFOLDING PROTEIN<br>class 1 collagenase	**51**	31	0.075	0.27	3.05	65	10.3% : 18.5% (6/58 : 12/65)	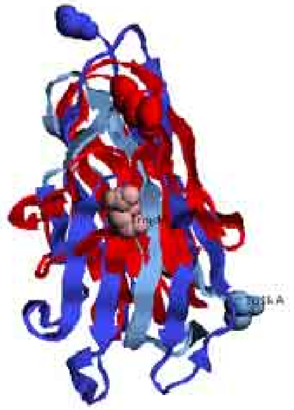
703	* 1g2b:A (62)<br>* 1gl5:A (67)	SPECTRIN ALPHA CHAIN<br>TYROSINE-PROTEIN KINASE TEC	**20**	39	0.0003	0.47	1.90	52	27.5% : 28.8% (11/40 : 15/52)	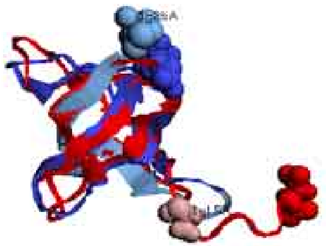
704	* 1g2b:A (63)<br>* 1h3h:A (60)	SPECTRIN ALPHA CHAIN<br>GRB2-RELATED ADAPTOR PROTEIN 2	**19**	35	0.004	0.62	1.52	52	27.5% : 30.8% (11/40 : 16/52)	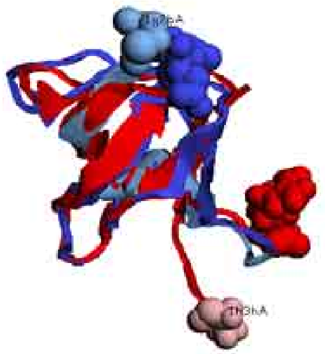

705	* 1g2b:A (62)	SPECTRIN ALPHA CHAIN	16	34	0.011	0.39	2.99	48	37.5% : 33.3% (12/32 : 16/48)	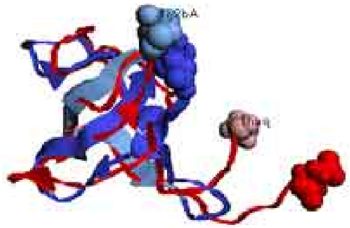
	* 1hsq_ (71)	PHOSPHOLIPASE C-GAMMA (SH3 DOMAIN)								
706	* 1g2b:A (63)	SPECTRIN ALPHA CHAIN	22	31	0.065	0.59	1.19	50	27.0% : 28.0% (10/37 : 14/50)	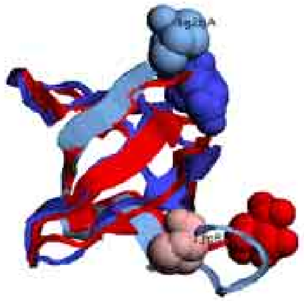
	* 1jo8:A (58)	ACTIN BINDING PROTEIN								
707	* 1g2b:A (62)	SPECTRIN ALPHA CHAIN	17	31	0.065	0.54	1.52	52	28.2% : 26.9% (11/39 : 14/52)	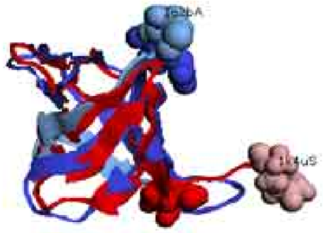
	* 1k4u:S (62)	PHAGOCYTE NADPH OXIDASE SUBUNIT P67PHOX								

708	* 1k76:A (60)	SEX MUSCLE ABNORMAL PROTEIN 5	**45**	42	2E-05	0.73	1.92	56	33.3% : 32.1% (13/39 : 18/56)	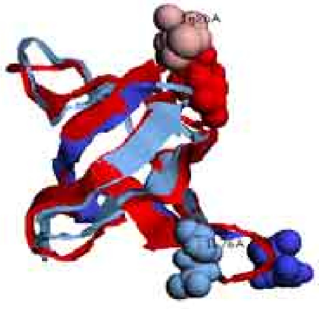
	* 1g2b:A (63)	SPECTRIN ALPHA CHAIN								
709	* 1g2b:A (62)	SPECTRIN ALPHA CHAIN	**15**	36	0.002	0.51	1.34	47	45.7% : 40.4% (16/35 : 19/47)	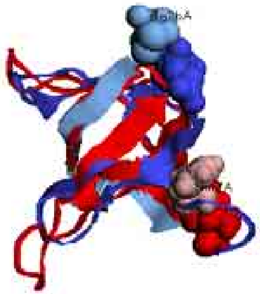
	* 1nm7:A (69)	Peroxisomal Membrane Protein PAS20								
710	* 1g2b:A (63)	SPECTRIN ALPHA CHAIN	**19**	36	0.002	0.53	1.91	42	26.7% : 31.0% (8/30 : 13/42)	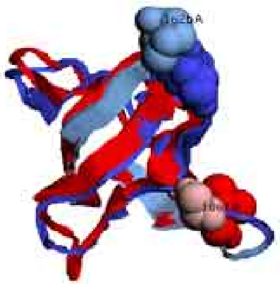
	* 1oot:A (58)	Hypothetical 40.4 kDa protein in PES4-HIS2 in								

711	* 1tuc:_ (65)	ALPHA-SPECTRIN	30	98	5E-22	0.75	1.34	52	100.0% : 100.0% (30/30 : 52/52)	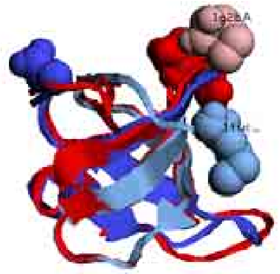
	* 1g2b:A (63)	SPECTRIN ALPHA CHAIN								
712	* 1g2b:A (62)	SPECTRIN ALPHA CHAIN	16	32	0.046	0.44	1.47	49	24.3% : 30.6% (9/37 : 15/49)	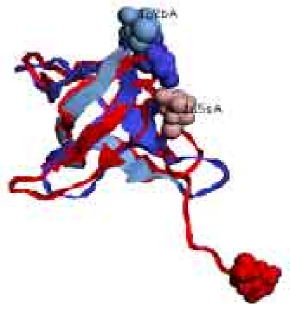
	* 1u5s:A (71)	Cytoplasmic protein NCK2								
713	* 1uhf:A (69)	INTERSECTIN 2	48	33	0.016	0.50	1.71	53	28.2% : 34.0% (11/39 : 18/53)	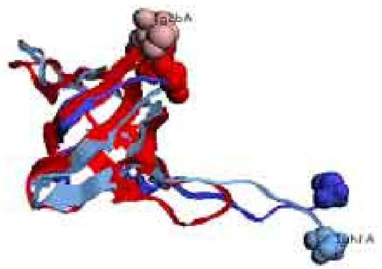
	* 1g2b:A (63)	SPECTRIN ALPHA CHAIN								

714	* 1g2b:A (63)	SPECTRIN ALPHA CHAIN	17	38	0.0006	0.57	1.19	52	32.5% : 30.8% (13/40 : 16/52)	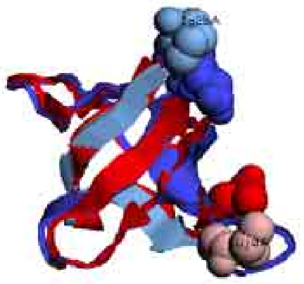
	* 1uj0:A (58)	signal transducing adaptor molecule (SH3 doma								
715	* 1g2b:A (63)	SPECTRIN ALPHA CHAIN	15	40	0.0002	0.52	1.32	50	34.2% : 36.0% (13/38 : 18/50)	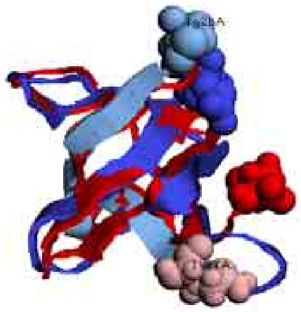
	* 1w1f:A (60)	TYROSINE-PROTEIN KINASE LYN								
716	* 1g2b:A (63)	SPECTRIN ALPHA CHAIN	18	33	0.019	0.66	1.23	51	37.8% : 37.3% (14/37 : 19/51)	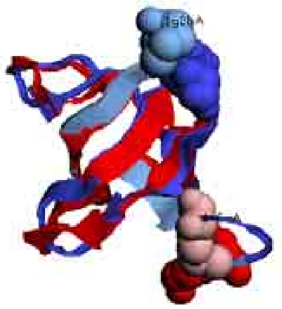
	* 1w6x:A (56)	NEUTROPHIL CYTOSOL FACTOR 4								

717	* 1g2b:A (63)	SPECTRIN ALPHA CHAIN	**15**	31	0.055	0.45	1.68	52	20.0% : 21.2% (8/40 : 11/52)	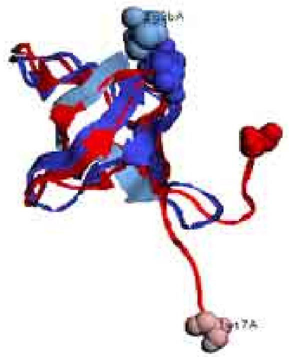
	* 1wi7:A (68)	SH3-domain kinase binding protein 1								
718	* 1g2b:A (62)	SPECTRIN ALPHA CHAIN	**15**	34	0.011	0.48	1.00	52	27.5% : 26.9% (11/40 : 14/52)	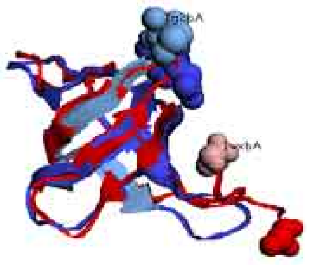
	* 1wxb:A (68)	Epidermal growth factor receptor pathway subs								
719	* 1wxt:A (68)	Hypothetical protein FLJ21522	**48**	32	0.032	0.59	1.48	55	31.6% : 29.1% (12/38 : 16/55)	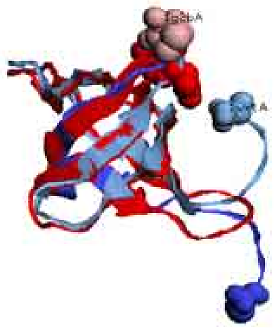
	* 1g2b:A (63)	SPECTRIN ALPHA CHAIN								

720	* 1wyx:A (68)	CRK-associated substrate	**47**	33	0.016	0.59	1.48	55	40.0% : 41.8% (16/40 : 23/55)	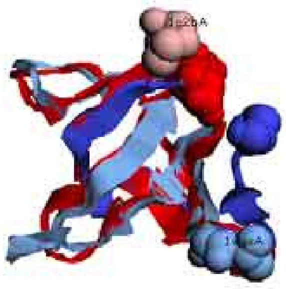
	* 1g2b:A (63)	SPECTRIN ALPHA CHAIN								
721	* 1x2k:A (68)	Osteoclast stimulating factor 1	**48**	37	0.001	0.49	1.25	53	27.5% : 30.2% (11/40 : 16/53)	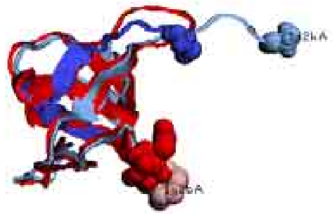
	* 1g2b:A (63)	SPECTRIN ALPHA CHAIN								
722	* 1g2b:A (63)	SPECTRIN ALPHA CHAIN	**15**	33	0.013	0.39	1.80	51	24.4% : 27.5% (10/41 : 14/51)	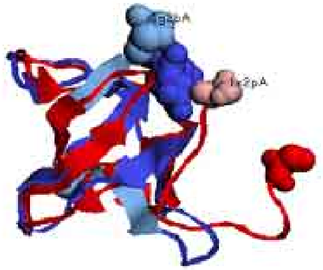
	* 1x2p:A (68)	Protein arginine N-methyltransferase 2								

723	* 1ynz:A (57)	Pin3p	**43**	34	0.009	0.70	1.35	53	31.6% : 34.0% (12/38 : 18/53)	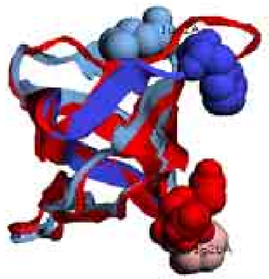
	* 1g2b:A (63)	SPECTRIN ALPHA CHAIN								
724	* 1z9z:A (60)	Cytoskeleton assembly control protein SLA1	**46**	34	0.011	0.63	0.99	52	36.8% : 34.6% (14/38 : 18/52)	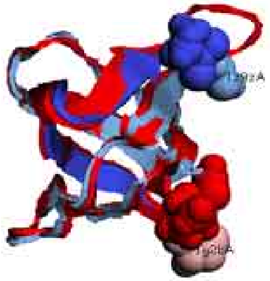
	* 1g2b:A (63)	SPECTRIN ALPHA CHAIN								
725	* 1zsg:A (65)	Rho guanine nucleotide exchange factor 7	**49**	33	0.019	0.56	2.00	54	22.5% : 25.9% (9/40 : 14/54)	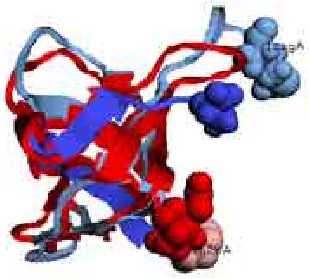
	* 1g2b:A (63)	SPECTRIN ALPHA CHAIN								

726	* 2a08:A (59)	Hypothetical 41.8 kDa protein in SPO13-ARG4 i	**46**	36	0.002	0.65	1.25	53	30.0% : 34.0% (12/40 : 18/53)	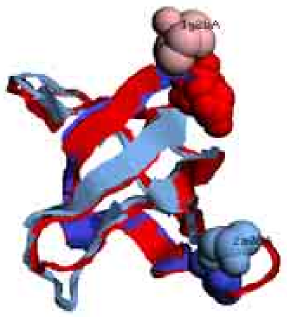
	* 1g2b:A (63)	SPECTRIN ALPHA CHAIN								
727	* 2ak5:A (60)	Rho guanine nucleotide exchange factor 7	**47**	32	0.032	0.56	1.03	51	23.7% : 25.5% (9/38 : 13/51)	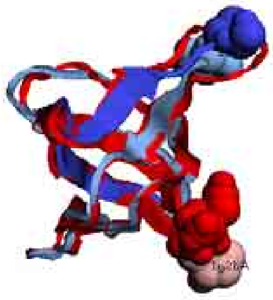
	* 1g2b:A (63)	SPECTRIN ALPHA CHAIN								
728	* 1g2b:A (62)	SPECTRIN ALPHA CHAIN	**18**	40	0.0001	0.53	1.51	50	34.2% : 36.0% (13/38 : 18/50)	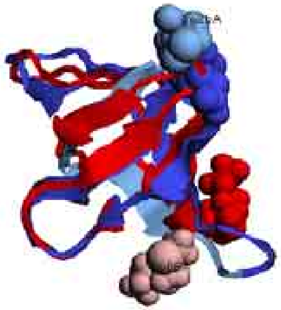
	* 2b86:A (67)	Cytoplasmic protein NCK2								

729	* 1g2b:A (63)	SPECTRIN ALPHA CHAIN	**21**	34	0.009	0.60	1.22	50	24.3% : 26.0% (9/37 : 13/50)	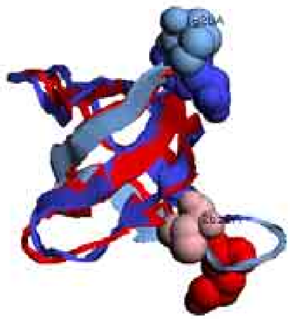
	* 2bz8:A (56)	SH3-DOMAIN KINASE BINDING PROTEIN 1								
730	* 1g2b:A (63)	SPECTRIN ALPHA CHAIN	**15**	34	0.006	0.43	1.46	51	38.5% : 39.2% (15/39 : 20/51)	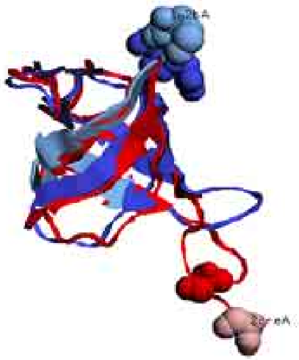
	* 2cre:A (71)	HEF-like protein								
731	* 1g2b:A (63)	SPECTRIN ALPHA CHAIN	**15**	31	0.065	0.44	1.02	49	25.0% : 26.5% (9/36 : 13/49)	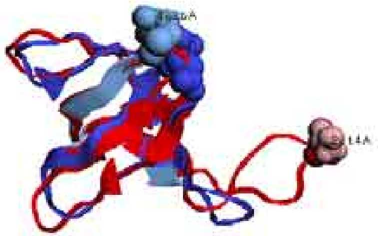
	* 2ct4:A (70)	Cdc42-interacting protein 4								

732	* 1g2b:A (63)	SPECTRIN ALPHA CHAIN	**15**	31	0.078	0.51	1.74	55	27.9% : 29.1% (12/43 : 16/55)	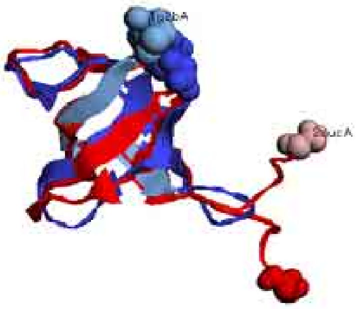
	* 2cuc:A (70)	SH3 domain containing ring finger 2								
733	* 2d8j:A (77)	fyn-related kinase	**57**	37	0.0009	0.54	1.61	54	28.9% : 29.6% (11/38 : 16/54)	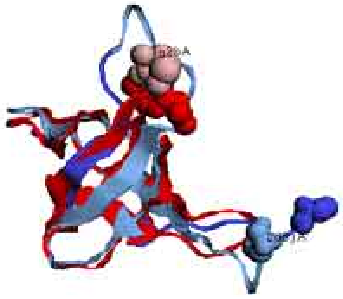
	* 1g2b:A (62)	SPECTRIN ALPHA CHAIN								
734	* 2da9:A (70)	SH3-domain kinase binding protein 1	**50**	34	0.009	0.56	1.66	55	30.0% : 32.7% (12/40 : 18/55)	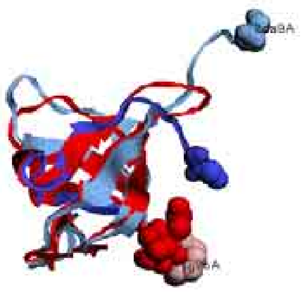
	* 1g2b:A (63)	SPECTRIN ALPHA CHAIN								

735	* 1g2b:A (63)	SPECTRIN ALPHA CHAIN	15	38	0.0004	0.41	1.49	52	32.5% : 34.6% (13/40 : 18/52)	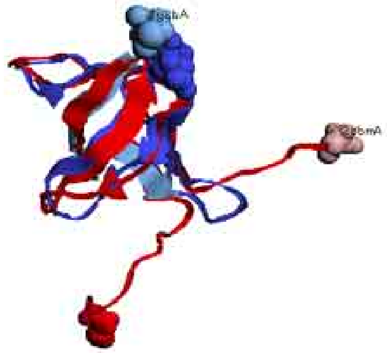
	* 2dbm:A (73)	SH3-containing GRB2-like protein 2								
736	* 1g2b:A (63)	SPECTRIN ALPHA CHAIN	15	33	0.013	0.42	1.96	51	27.5% : 29.4% (11/40 : 15/51)	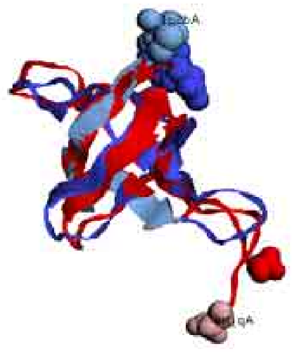
	* 2djq:A (68)	SH3 domain containing ring finger 2								
737	* 1g2b:A (63)	SPECTRIN ALPHA CHAIN	15	32	0.027	0.45	1.59	52	20.0% : 23.1% (8/40 : 12/52)	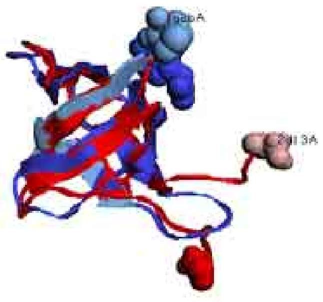
	* 2dl3:A (68)	Sorbin and SH3 domain-containing protein 1								

738	* 2dl4:A (68)	Protein Stac	**48**	44	6E-06	0.54	1.45	53	39.5% : 41.5% (15/38 : 22/53)	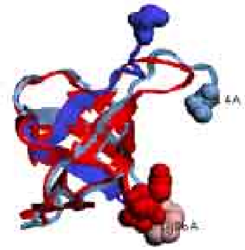
	* 1g2b:A (63)	SPECTRIN ALPHA CHAIN								
739	* 2dl7:A (73)	KIAA0769 protein	**52**	32	0.046	0.49	1.52	51	35.0% : 35.3% (14/40 : 18/51)	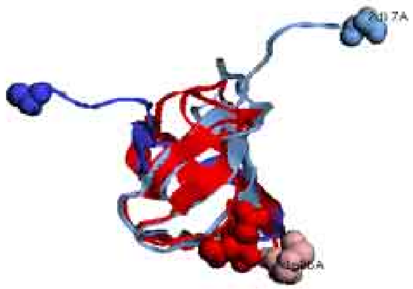
	* 1g2b:A (63)	SPECTRIN ALPHA CHAIN								
740	* 1g2b:A (63)	SPECTRIN ALPHA CHAIN	**15**	35	0.005	0.43	1.94	53	31.7% : 30.2% (13/41 : 16/53)	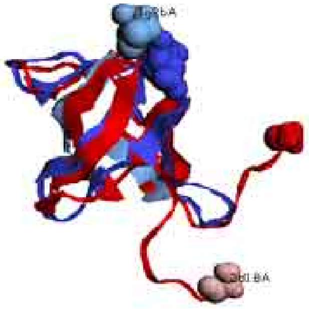
	* 2dl8:A (72)	SLIT-ROBO Rho GTPase-activating protein 2								

741	* 2dlm:A (68)	Vinexin	**48**	35	0.003	0.49	1.58	51	32.4% : 37.3% (12/37 : 19/51)	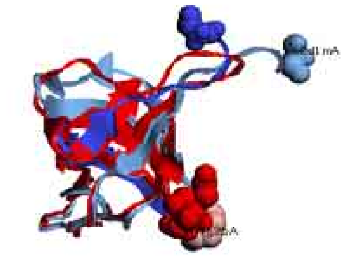
	* 1g2b:A (63)	SPECTRIN ALPHA CHAIN								
742	* 1g2b:A (63)	SPECTRIN ALPHA CHAIN	**14**	33	0.016	0.36	1.05	48	22.2% : 27.1% (8/36 : 13/48)	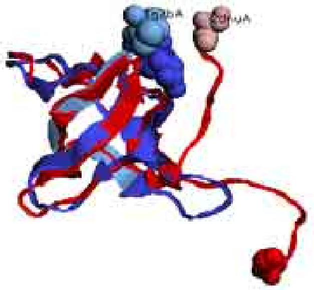
	* 2dnu:A (71)	SH3 multiple domains 1								
743	* 1g2b:A (63)	SPECTRIN ALPHA CHAIN	**19**	35	0.003	0.61	1.55	53	30.0% : 32.1% (12/40 : 17/53)	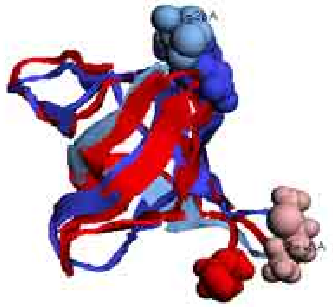
	* 2ew3:A (60)	SH3-containing GRB2-like protein 3								

744	* 1g2b:A (63)	SPECTRIN ALPHA CHAIN	**21**	33	0.016	0.57	1.43	52	23.1% : 26.9% (9/39 : 14/52)	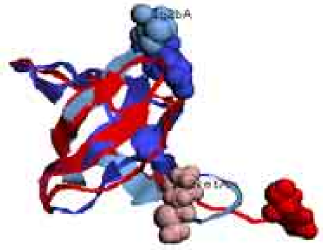
	* 2fei:A (60)	CD2-associated protein								
745	* 1g2b:A (63)	SPECTRIN ALPHA CHAIN	**16**	37	0.001	0.60	1.12	51	38.5% : 37.3% (15/39 : 19/51)	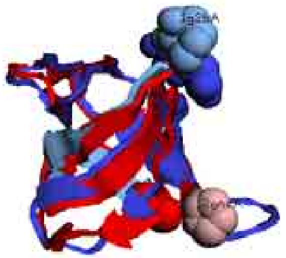
	* 2gnc:A (55)	SLIT-ROBO Rho GTPase-activating protein 1								
746	* 2j6f:A (57)	CD2-ASSOCIATED PROTEIN	**51**	40	0.0001	0.26	1.72	44	21.1% : 22.7% (8/38 : 10/44)	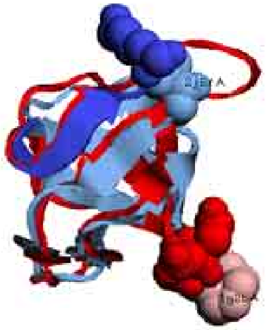
	* 1g2b:A (63)	SPECTRIN ALPHA CHAIN								

747	1xx1:A (285)	Sphingomyelinase I	145	37	0.002	0.32	3.27	145	11.9% : 11.7% (15/126 : 17/145)	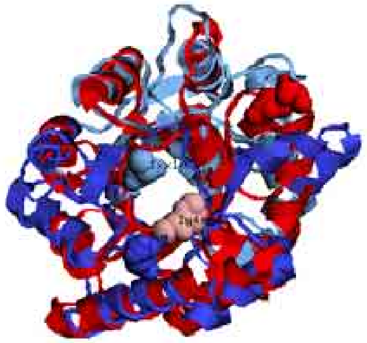
	1g4e:A (221)	THIAMIN PHOSPHATE SYNTHASE								
748	1g4e:A (221)	THIAMIN PHOSPHATE SYNTHASE	97	35	0.005	0.25	2.42	133	19.8% : 7.5% (26/131 : 10/133)	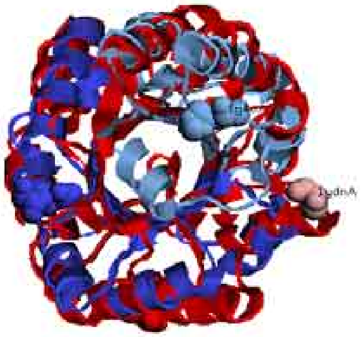
	1ydn:A (283)	HYDROXYMETHYLGLUTARYL-COA LYASE								
749	1ggq:A (162)	OUTER SURFACE PROTEIN C	133	36	0.002	0.55	2.44	64	12.5% : 4.7% (7/56 : 3/64)	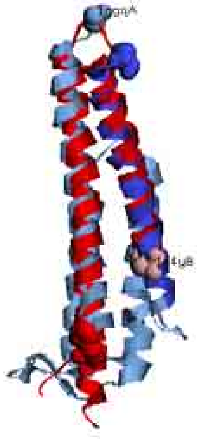
	1g4y:B (82)	CALCIUM-ACTIVATED POTASSIUM CHANNEL RSK2								

750	1g4y:B (82)	CALCIUM-ACTIVATED POTASSIUM CHANNEL RSK2	**26**	35	0.004	0.41	2.23	55	3.3% : 5.5% (1/30 : 3/55)	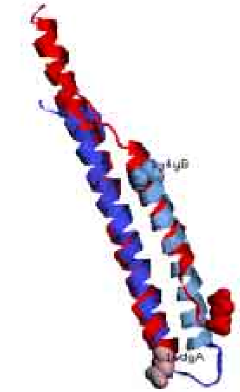
	1wdg:A (82)	E2 glycoprotein								
751	1z0p:A (74)	hypothetical protein SPy1572	**39**	34	0.006	0.45	1.94	54	3.2% : 1.9% (1/31 : 1/54)	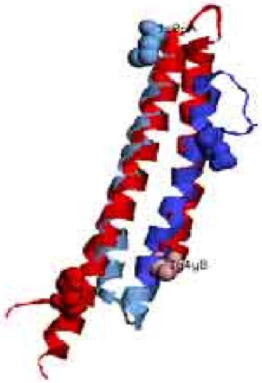
	1g4y:B (82)	CALCIUM-ACTIVATED POTASSIUM CHANNEL RSK2								
752	1g4y:B (82)	CALCIUM-ACTIVATED POTASSIUM CHANNEL RSK2	**37**	42	3E-05	0.23	2.45	55	10.2% : 5.5% (5/49 : 3/55)	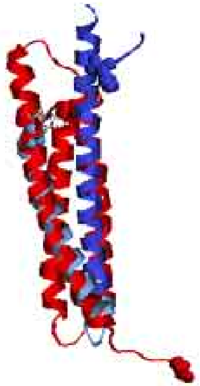
	2dnx:A (130)	Syntaxin-12								

753	1g4y:B (82)	CALCIUM-ACTIVATED POTASSIUM CHANNEL RSK2	**45**	40	0.0001	0.27	1.53	42	10.0% : 7.1% (4/40 : 3/42)	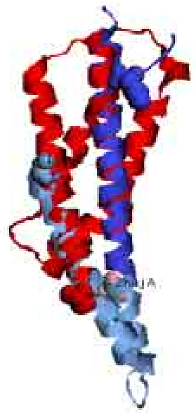
	2huj:A (125)	Lin2004 protein								
754	1g4y:B (82)	CALCIUM-ACTIVATED POTASSIUM CHANNEL RSK2	**27**	40	0.0002	0.60	2.69	68	5.2% : 7.4% (3/58 : 5/68)	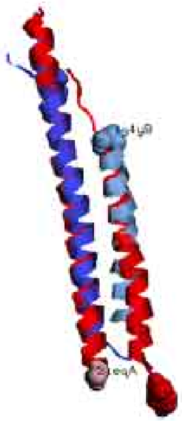
	2ieq:A (89)	Spike glycoprotein								
755	1g55:A (315)	DNA CYTOSINE METHYLTRANSFERASE DNMT2	**276**	52	6E-08	0.27	3.03	135	14.8% : 13.3% (17/115 : 18/135)	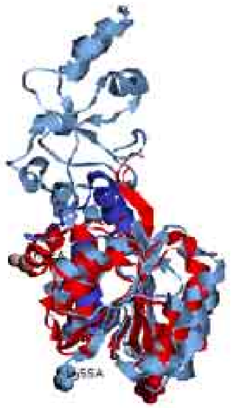
	1ne2:A (179)	hypothetical protein ta1320								

756	1g55:A (315)	DNA CYTOSINE METHYLTRANSFERASE DNMT2	258	35	0.009	0.20	3.38	137	15.8% : 15.3% (18/114 : 21/137)	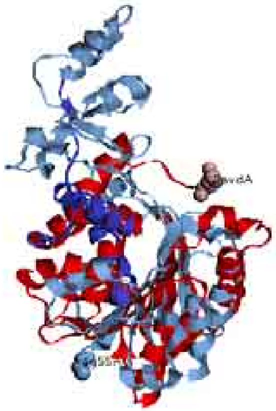
	2avd:A (219)	Catechol-O-methyltransferase								
757	1g5v:A (56)	SURVIVAL MOTOR NEURON PROTEIN 1	17	33	0.018	0.41	1.72	47	2.9% : 8.5% (1/35 : 4/47)	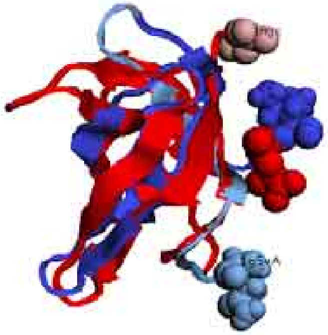
	1mjc_ (69)	MAJOR COLD-SHOCK PROTEIN 7.4								
758	1g5z:A (164)	OUTER SURFACE PROTEIN C	127	32	0.043	0.20	2.31	83	8.6% : 6.0% (5/58 : 5/83)	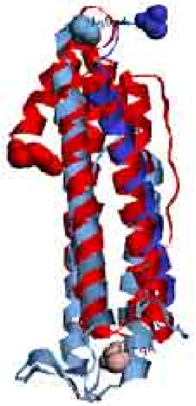
	1krq:A (164)	ferritin								

759	2crb:A (97)<br>1g5z:A (164)	nuclear receptor binding factor 2<br>OUTER SURFACE PROTEIN C	**53**	34	0.007	0.31	2.87	69	7.7% : 17.4%<br>(4/52 : 12/69)	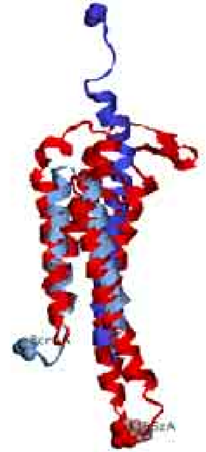
760	1g60:A (239)<br>1i9g:A (264)	Adenine-specific Methyltransferase MboIIA<br>HYPOTHETICAL PROTEIN RV2118C	**100**	43	3E-05	0.24	2.07	128	19.0% : 18.8%<br>(15/79 : 24/128)	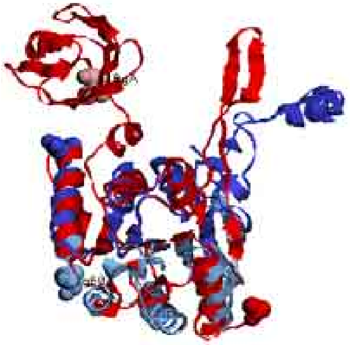
761	1g60:A (239)<br>1im8:A (225)	Adenine-specific Methyltransferase MboIIA<br>YecO	**157**	37	0.002	0.33	2.17	127	10.8% : 14.2%<br>(9/83 : 18/127)	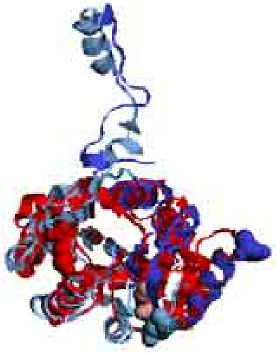

762	1g60:A (239)	Adenine-specific Methyltransferase MboIIA	**179**	47	2E-06	0.51	2.76	149	10.4% : 10.7% (10/96 : 16/149)	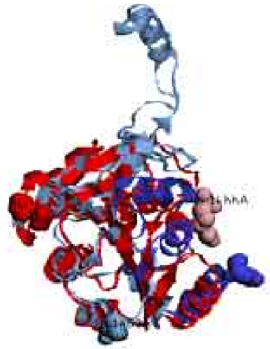
	1khh:A (193)	Guanidinoacetate methyltransferase								
763	1g60:A (239)	Adenine-specific Methyltransferase MboIIA	**135**	42	5E-05	0.24	1.77	135	11.7% : 14.1% (9/77 : 19/135)	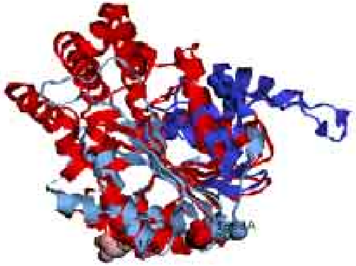
	1kpi:A (291)	CYCLOPROPANE-FATTY-ACYL-PHOSPHOLIPID SYNTHASE								
764	1g60:A (239)	Adenine-specific Methyltransferase MboIIA	**147**	45	8E-06	0.26	1.76	135	8.9% : 14.1% (7/79 : 19/135)	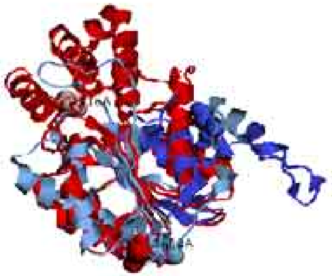
	1l1e:A (272)	mycolic acid synthase								

765	1g60:A (239)	Adenine-specific Methyltransferase MboIIA	**172**	42	7E-05	0.32	2.34	138	6.9% : 13.0% (5/72 : 18/138)	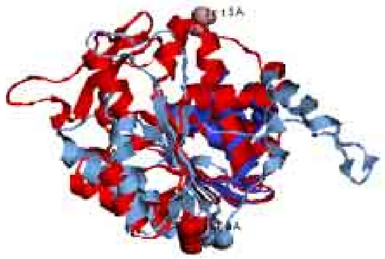
	1ri1:A (252)	mRNA CAPPING ENZYME								
766	1g60:A (239)	Adenine-specific Methyltransferase MboIIA	**134**	44	2E-05	0.25	1.69	135	10.5% : 14.1% (8/76 : 19/135)	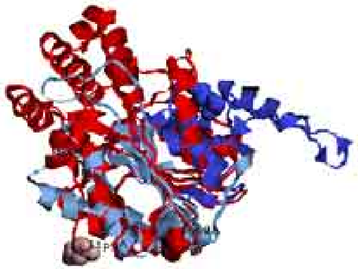
	1tpy:A (285)	methoxy mycolic acid synthase 2								
767	1g60:A (239)	Adenine-specific Methyltransferase MboIIA	**159**	44	1E-05	0.36	2.01	131	7.7% : 12.2% (6/78 : 16/131)	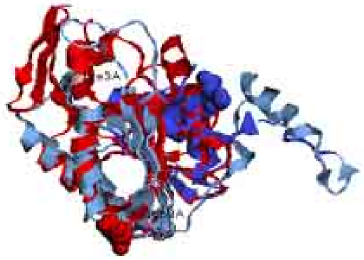
	1ve3:A (214)	hypothetical protein PH0226								

768	1g60:A (239)	Adenine-specific Methyltransferase MboIIA	**181**	36	0.005	0.33	3.22	136	11.5% : 14.7% (10/87 : 20/136)	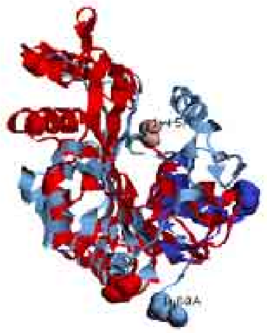
	1vl5:A (231)	unknown conserved protein BH2331								
769	1g60:A (239)	Adenine-specific Methyltransferase MboIIA	**177**	45	6E-06	0.34	2.50	142	14.3% : 16.9% (12/84 : 24/142)	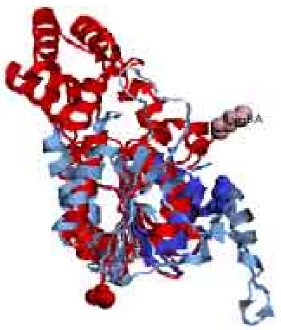
	1wg8:A (281)	predicted S-adenosylmethionine-dependent meth								
770	1g60:A (239)	Adenine-specific Methyltransferase MboIIA	**150**	49	5E-07	0.44	2.29	140	7.1% : 19.3% (5/70 : 27/140)	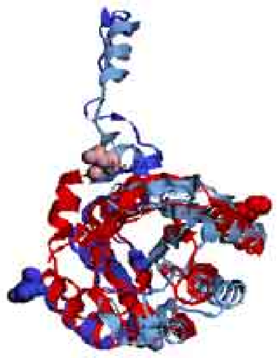
	1wy7:A (197)	hypothetical protein PH1948								

771	1g60:A<br>(239)<br>1wzn:A<br>(245)	Adenine-specific Methyltransferase MboIIA<br>SAM-dependent methyltransferase	**155**	47	3E-06	0.27	2.32	127	7.1% : 14.2%<br>(6/84 : 18/127)	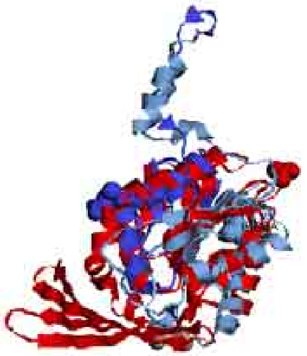
772	1g60:A<br>(239)<br>1xdz:A<br>(239)	Adenine-specific Methyltransferase MboIIA<br>Methyltransferase gidB	**126**	34	0.012	0.29	2.73	135	10.8% : 10.4%<br>(9/83 : 14/135)	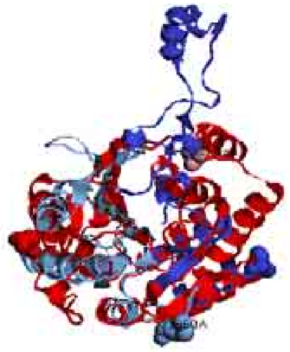
773	1g60:A<br>(239)<br>1xj5:A<br>(290)	Adenine-specific Methyltransferase MboIIA<br>Spermidine synthase 1	**117**	35	0.01	0.20	2.67	127	11.7% : 12.6%<br>(11/94 : 16/127)	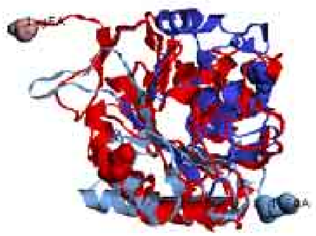

774	1g60:A (239)<br>1xq1:A (221)	Adenine-specific Methyltransferase MboIIA<br>PUTATIVE TROPINONE REDUCATSE	**189**	31	0.1	0.28	2.63	124	7.9% : 8.9% (7/89 : 11/124)	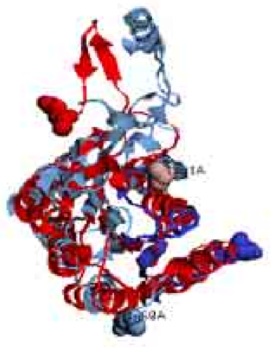
775	1g60:A (239)<br>1xtp:A (248)	Adenine-specific Methyltransferase MboIIA<br>LMAJ004091AAA	**111**	37	0.002	0.25	2.45	125	9.9% : 14.4% (8/81 : 18/125)	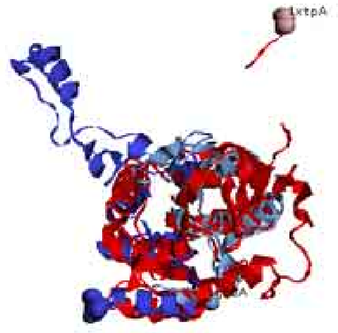
776	1g60:A (239)<br>1xxl:A (234)	Adenine-specific Methyltransferase MboIIA<br>YcgJ protein	**180**	41	0.0001	0.35	2.04	140	10.6% : 16.4% (9/85 : 23/140)	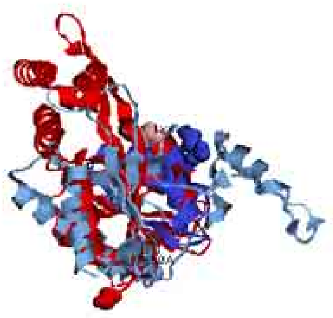

777	1g60:A (239)	Adenine-specific Methyltransferase MboIIA	**159**	33	0.024	0.29	2.39	131	10.5% : 13.7% (8/76 : 18/131)	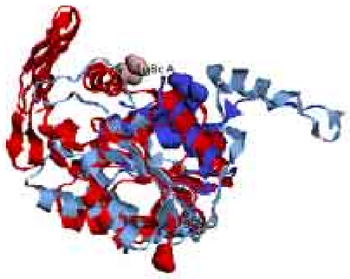
	1y8c:A (246)	S-adenosylmethionine-dependent methyltransfer								
778	1g60:A (239)	Adenine-specific Methyltransferase MboIIA	**128**	37	0.001	0.26	2.11	121	11.0% : 14.0% (8/73 : 17/121)	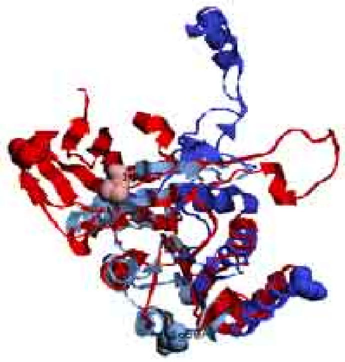
	1yb2:A (233)	hypothetical protein Ta0852								
779	1g60:A (239)	Adenine-specific Methyltransferase MboIIA	**165**	50	3E-07	0.32	2.39	125	7.4% : 12.8% (6/81 : 16/125)	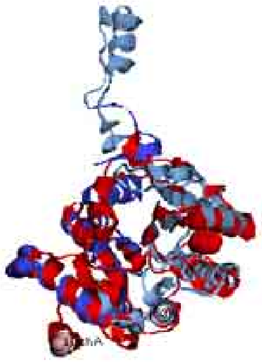
	1yzh:A (204)	tRNA (guanine-N(7)-)-methyltransferase								

780	1g60:A (239)	Adenine-specific Methyltransferase MboIIA	**143**	47	2E-06	0.38	2.79	149	11.6% : 11.4% (11/95 : 17/149)	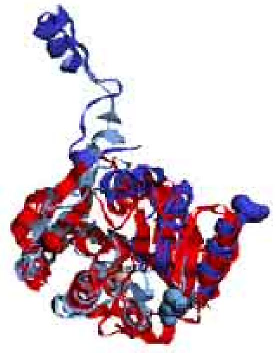
	1zx0:A (229)	Guanidinoacetate N-methyltransferase								
781	1g60:A (239)	Adenine-specific Methyltransferase MboIIA	**138**	33	0.029	0.35	2.40	133	11.2% : 12.8% (11/98 : 17/133)	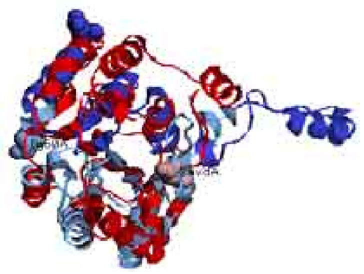
	2avd:A (219)	Catechol-O-methyltransferase								
782	1g60:A (239)	Adenine-specific Methyltransferase MboIIA	**105**	33	0.034	0.27	2.90	133	7.5% : 13.5% (6/80 : 18/133)	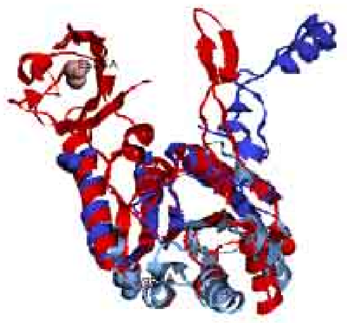
	2b25:A (258)	hypothetical protein								

783	1g60:A (239)	Adenine-specific Methyltransferase MboIIA	**102**	48	9E-07	0.24	2.61	133	8.0% : 11.3% (7/88 : 15/133)	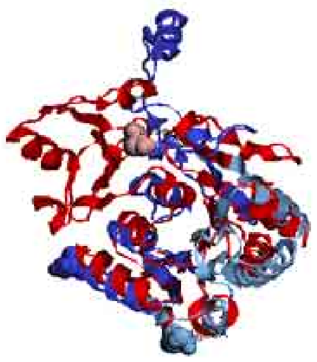
	2b9e:A (276)	NOL1/NOP2/Sun domain family, member 5 isoform								
784	1g60:A (239)	Adenine-specific Methyltransferase MboIIA	**182**	47	2E-06	0.58	2.62	132	9.5% : 15.9% (8/84 : 21/132)	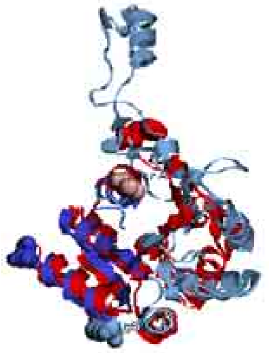
	2esr:A (160)	Methyltransferase								
785	1g60:A (239)	Adenine-specific Methyltransferase MboIIA	**136**	35	0.006	0.35	2.32	138	11.6% : 14.5% (10/86 : 20/138)	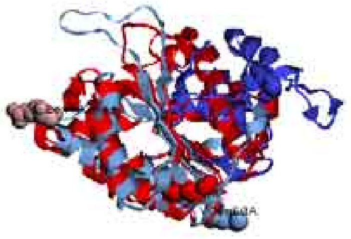
	2ex4:A (222)	adrenal gland protein AD-003								

786	1g60:A (239)	Adenine-specific Methyltransferase MboIIA	**166**	47	2E-06	0.36	2.17	135	9.7% : 11.9% (9/93 : 16/135)	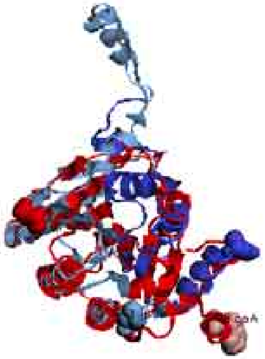
	2fca:A (205)	tRNA (guanine-N(7)-)-methyltransferase								
787	1g60:A (239)	Adenine-specific Methyltransferase MboIIA	**156**	42	8E-05	0.39	2.10	116	6.1% : 18.1% (2/33 : 21/116)	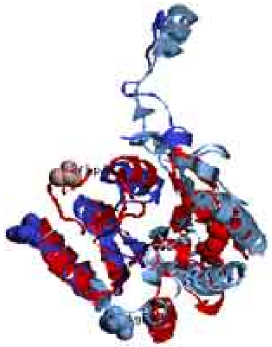
	2fhp:A (183)	methylase, putative								
788	1g60:A (239)	Adenine-specific Methyltransferase MboIIA	**144**	45	8E-06	0.27	1.96	139	9.9% : 15.1% (8/81 : 21/139)	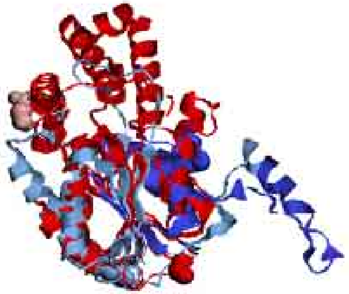
	2fk7:A (278)	methoxy mycolic acid synthase 4								

789	1g60:A (239)	Adenine-specific Methyltransferase MboIIA	**158**	43	2E-05	0.44	1.93	126	5.6% : 18.3% (4/71 : 23/126)	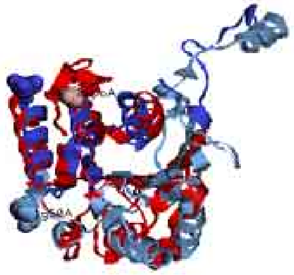
	2fpo:A (178)	methylase yhhF								
790	1g60:A (239)	Adenine-specific Methyltransferase MboIIA	**135**	33	0.034	0.43	3.13	149	9.9% : 16.8% (8/81 : 25/149)	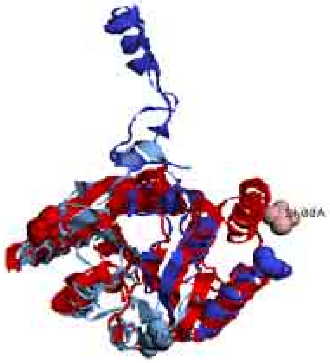
	2h00:A (226)	methyltransferase 10 domain containing protei								
791	1g60:A (239)	Adenine-specific Methyltransferase MboIIA	**161**	47	2E-06	0.43	2.25	124	15.9% : 17.7% (11/69 : 22/124)	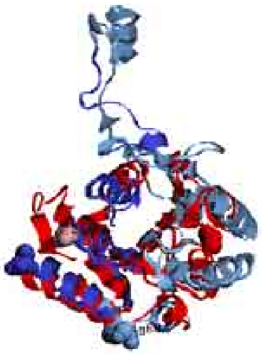
	2ift:A (177)	Putative methylase HI0767								

792	1g60:A (239)	Adenine-specific Methyltransferase MboIIA	**193**	32	0.059	0.26	3.09	124	11.9% : 11.3% (10/84 : 14/124)	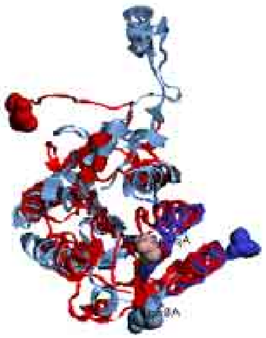
	2nwq:A (230)	Probable short-chain dehydrogenase								
793	1g60:A (239)	Adenine-specific Methyltransferase MboIIA	**129**	36	0.004	0.26	2.24	136	10.0% : 13.2% (9/90 : 18/136)	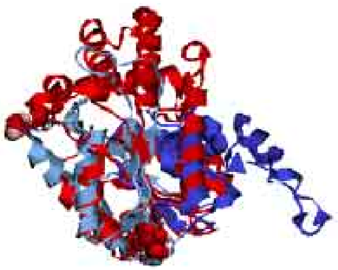
	2o57:A (282)	putative sarcosine dimethylglycine methyltran								
794	1g7y:A (252)	STEM/LEAF LECTIN DB58	**116**	139	3E-34	0.83	1.51	224	45.6% : 42.9% (52/114 : 96/224)	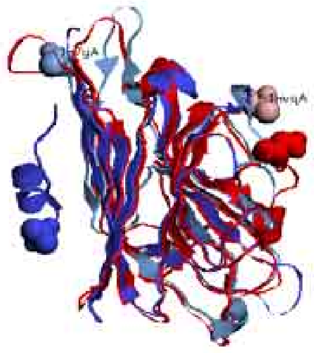
	1mvq:A (236)	lectin, isoform 1								

795	1g86:A (141)	CHARCOT-LEYDEN CRYSTAL PROTEIN	**40**	41	7E-05	0.27	2.62	94	5.5% : 9.6% (3/55 : 9/94)	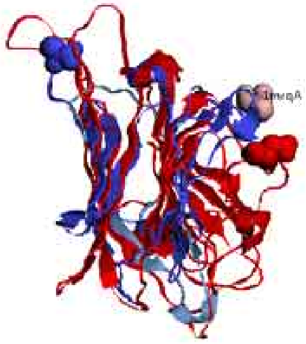
	1mvq:A (236)	lectin, isoform 1								
796	1v70:A (105)	probable antibiotics synthesis protein	**67**	32	0.036	0.20	3.44	54	8.5% : 9.3% (4/47 : 5/54)	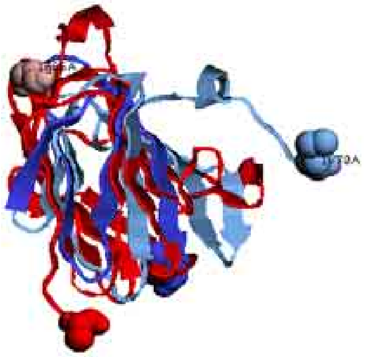
	1g86:A (141)	CHARCOT-LEYDEN CRYSTAL PROTEIN								
797	1g9f:A (242)	LECTIN	**114**	147	1E-36	0.80	1.25	215	48.6% : 47.9% (54/111 : 103/215)	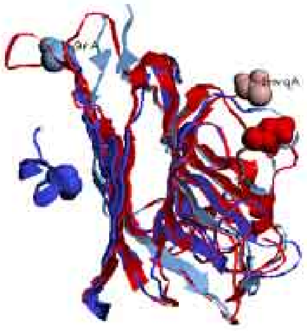
	1mvq:A (236)	lectin, isoform 1								

798	1g9o:A<br>(91)<br><br>2hga:A<br>(103)	NHE-RF<br><br>Conserved protein MTH1368	**25**	33	0.022	0.38	2.23	74	23.6% : 21.6%<br>(13/55 : 16/74)	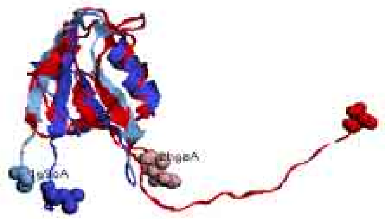
799	*<br>1mve:A<br>(243)<br><br>*<br>1gbg_<br>(214)	Truncated 1,3-1,4-beta-D-glucanase<br><br>(1,3-1,4)-BETA-D-GLUCAN 4 GLUCANOHYDROLASE	**181**	105	6E-24	0.51	1.50	190	34.5% : 30.0%<br>(50/145 : 57/190)	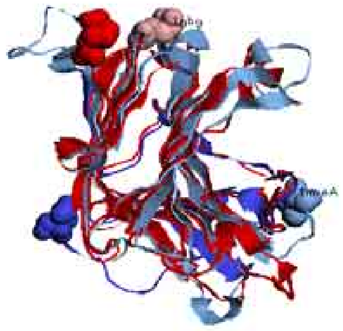
800	1ge9:A<br>(184)<br><br>1rj1:A<br>(148)	RIBOSOME RECYCLING FACTOR<br><br>invertase inhibitor	**85**	32	0.035	0.22	3.43	80	2.0% : 7.5%<br>(1/51 : 6/80)	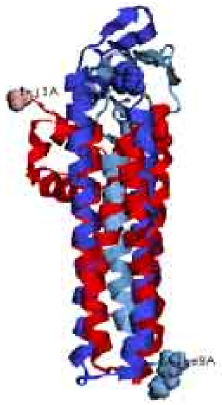

801	1ge9:A (184)	RIBOSOME RECYCLING FACTOR	**82**	31	0.086	0.21	3.38	80	3.4% : 10.0% (2/59 : 8/80)	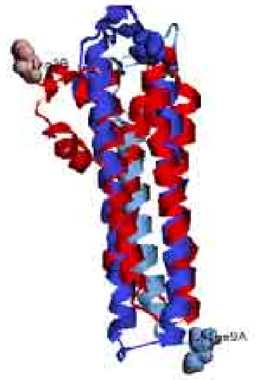
	1xg2:B (151)	Pectinesterase inhibitor								
802	1ggq:A (162)	OUTER SURFACE PROTEIN C	**51**	38	0.0004	0.24	2.56	75	17.2% : 5.3% (11/64 : 4/75)	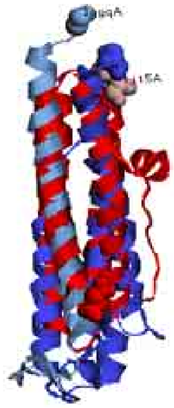
	1ji5:A (142)	Dlp-1								
803	1ggq:A (162)	OUTER SURFACE PROTEIN C	**101**	32	0.029	0.37	3.21	102	13.2% : 8.8% (7/53 : 9/102)	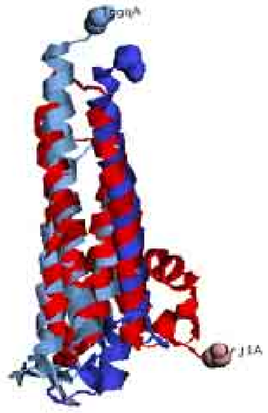
	1rj1:A (148)	invertase inhibitor								

804	* 1gl5:A (67)	TYROSINE-PROTEIN KINASE TEC	**14**	32	0.032	0.48	1.81	52	20.5% : 28.8% (8/39 : 15/52)	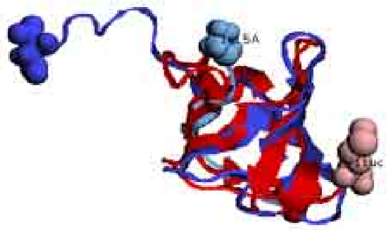
	* 1tuc_ (63)	ALPHA-SPECTRIN								
805	1gmi:A (136)	PROTEIN KINASE C, EPSILON TYPE	**120**	45	3E-06	0.21	2.51	94	25.0% : 22.3% (20/80 : 21/94)	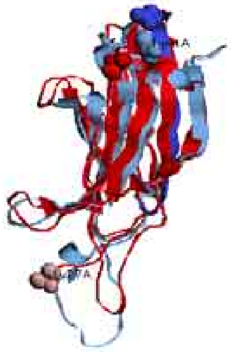
	1v27:A (141)	Regulating synaptic membrane exocytosis prote								
806	1mvq:A (236)	lectin, isoform 1	**125**	133	1E-32	0.84	1.55	214	53.6% : 50.0% (60/112 : 107/214)	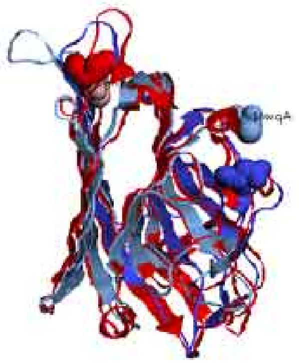
	1gnz:A (236)	LECTIN								

807	1gqa:A (130)	CYTOCHROME C'	**75**	35	0.004	0.39	3.00	82	7.3% : 13.4% (4/55 : 11/82)	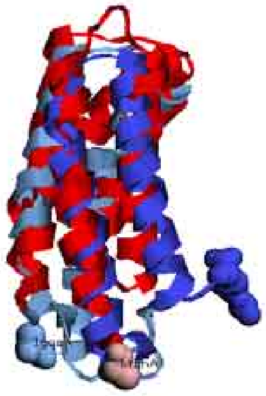
	1i5n:A (128)	CHEMOTAXIS PROTEIN CHEA								
808	1rty:A (159)	yvqk protein	**89**	34	0.012	0.23	3.12	80	7.0% : 5.0% (4/57 : 4/80)	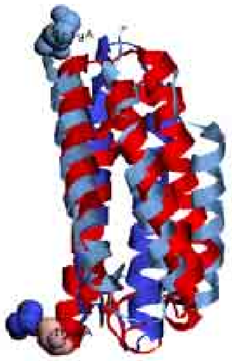
	1gqa:A (130)	CYTOCHROME C'								
809	1rhc:A (330)	F420-dependent alcohol dehydrogenase	**261**	33	0.034	0.33	3.46	173	7.5% : 9.2% (12/160 : 16/173)	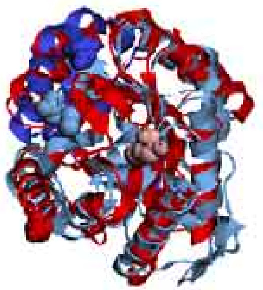
	1gqn:A (252)	3-DEHYDROQUINATE DEHYDRATASE								

810	1z69:A (327)	Coenzyme F420-dependent N(5),N(10)-methylenet	**192**	40	0.0005	0.31	3.49	169	8.3% : 5.3% (11/133 : 9/169)	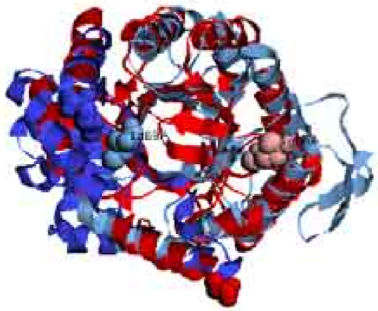
	1gqn:A (252)	3-DEHYDROQUINATE DEHYDRATASE								
811	1gsl:_ (243)	GRIFFONIA SIMPLICIFOLIA LECTIN 4	**122**	143	2E-35	0.84	1.54	218	47.3% : 45.4% (53/112 : 99/218)	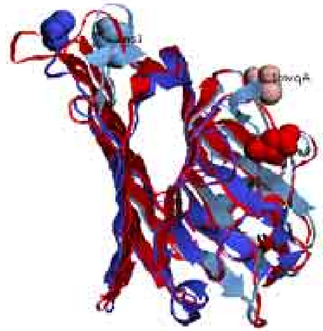
	1mvq:A (236)	lectin, isoform 1								
812	* 1gu3:A (142)	ENDOGLUCANASE C	**31**	35	0.004	0.21	2.47	90	11.6% : 15.6% (8/69 : 14/90)	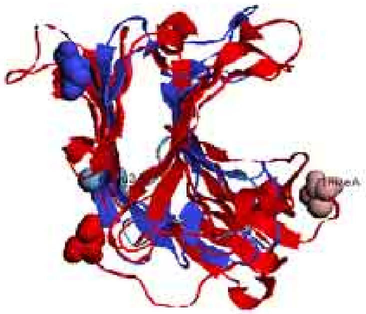
	* 1mve:A (243)	Truncated 1,3-1,4-beta-D-glucanase								

813	* 1mve:A (243)<br>* 1gui:A (155)	Truncated 1,3-1,4-beta-D-glucanase<br>LAMINARINASE 16A	**178**	40	0.0003	0.25	3.35	101	14.9% : 14.9% (11/74 : 15/101)	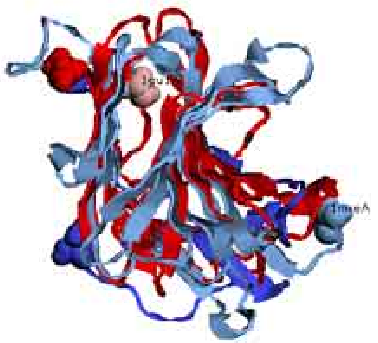
814	1mvq:A (236)<br>1gv9:A (224)	lectin, isoform 1<br>P58/ERGIC-53	**115**	50	3E-07	0.51	2.23	165	20.2% : 18.2% (18/89 : 30/165)	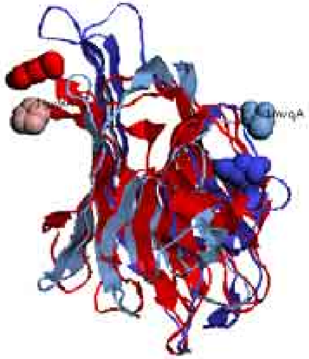
815	1gvf:A (274)<br>2h9a:B (307)	TAGATOSE-BISPHOSPHATE ALDOLASE AGAY<br>CO dehydrogenase/acetyl-CoA synthase, iron-su	**123**	35	0.008	0.24	3.27	157	14.4% : 15.9% (20/139 : 25/157)	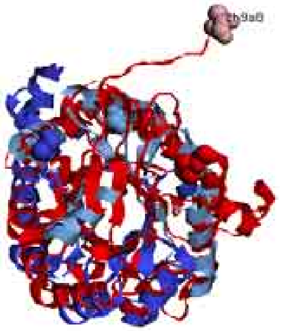

816	1ysm:A (55)	Calcyclin-binding protein	**21**	32	0.03	0.36	2.99	44	10.0% : 4.5% (4/40 : 2/44)	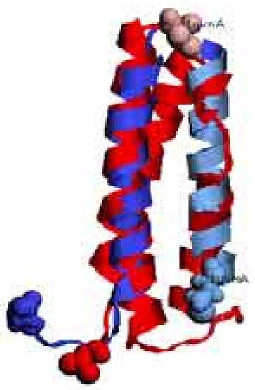
	1gvn:A (87)	EPSILON								
817	* 1mve:A (243)	Truncated 1,3-1,4-beta-D-glucanase	**191**	38	0.0007	0.21	3.47	98	11.8% : 12.2% (9/76 : 12/98)	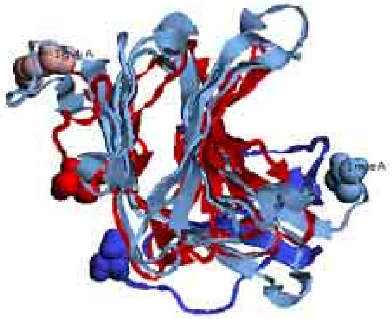
	* 1gwk:A (141)	NON-CATALYTIC PROTEIN 1								
818	1gxr:A (336)	TRANSDUCIN-LIKE ENHANCER PROTEIN 1	**152**	81	2E-16	0.51	2.81	252	15.9% : 11.5% (33/207 : 29/252)	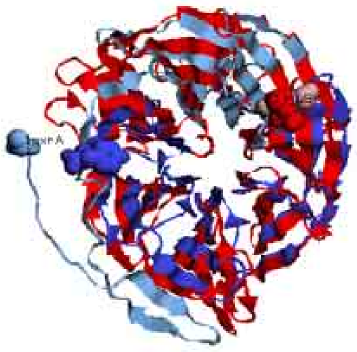
	1jju:B (337)	QUINOHEMOPROTEIN AMINE DEHYDROGENASE								

819	1olz:A (622)	SEMAPHORIN 4D	**347**	36	0.009	0.23	2.67	235	11.6% : 8.9% (26/225 : 21/235)	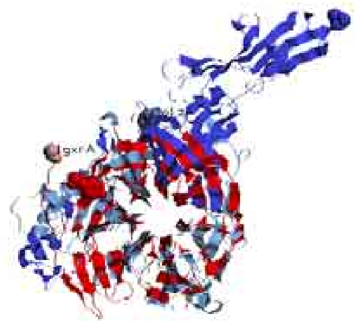
	1gxr:A (336)	TRANSDUCIN-LIKE ENHANCER PROTEIN 1								
820	2ayd:A (76)	WRKY transcription factor 1	**33**	40	0.0001	0.30	2.75	52	3.4% : 13.5% (1/29 : 7/52)	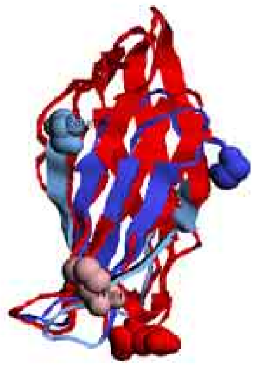
	1gyu:A (120)	ADAPTER-RELATED PROTEIN COMPLEX 1 GAMMA 1 SUB								
821	1gzw:A (134)	GALECTIN-1	**36**	34	0.007	0.28	2.50	91	13.4% : 9.9% (9/67 : 9/91)	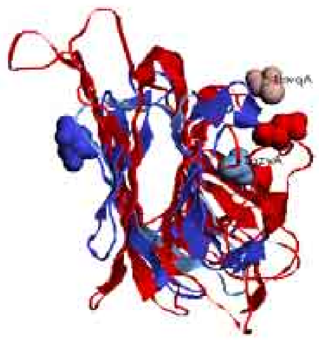
	1mvq:A (236)	lectin, isoform 1								

822	1tmy:_ (118)	CHEY PROTEIN	**41**	37	0.001	0.20	2.95	87	11.1% : 10.3% (9/81 : 9/87)	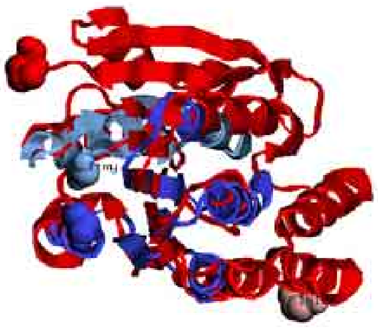
	1h1d:A (215)	CATECHOL-O-METHYLTRANSFERASE								
823	1zke:A (81)	Hypothetical protein HP1531	**49**	33	0.015	0.66	3.31	53	8.2% : 3.8% (4/49 : 2/53)	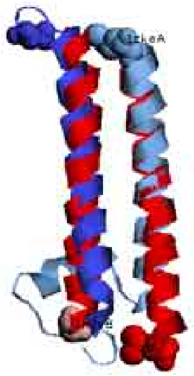
	1h2s:B (60)	SENSORY RHODOPSIN II TRANSDUCER								
824	* 1h3h:A (60)	GRB2-RELATED ADAPTOR PROTEIN 2	**15**	36	0.002	0.48	1.49	52	28.6% : 30.8% (12/42 : 16/52)	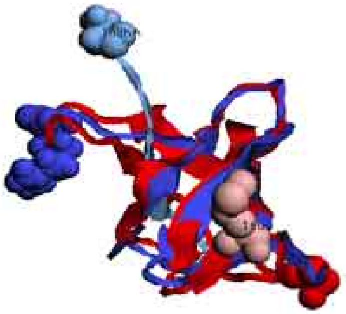
	* 1tuc_ (65)	ALPHA-SPECTRIN								

825	1h3q:A (140)<br>1j3w:A (134)	SEDLIN<br>Giding protein-mglB	**120**	32	0.044	0.29	3.34	98	8.6% : 7.1% (7/81 : 7/98)	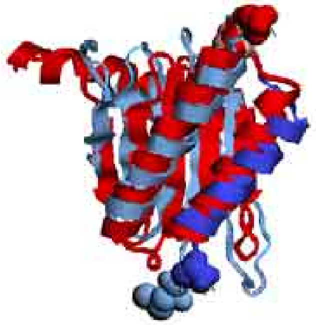
826	1nps:A (87)<br>1h4a:X (174)	DEVELOPMENT-SPECIFIC PROTEIN S<br>GAMMA CRYSTALLIN D	**47**	32	0.038	0.30	1.63	80	23.0% : 27.5% (17/74 : 22/80)	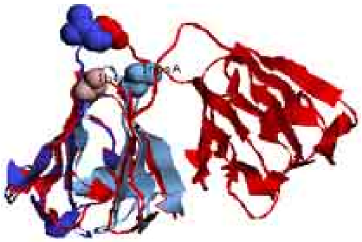
827	1h4g:A (206)<br>1y43:B (171)	XYLANASE<br>Aspergillopepsin II heavy chain	**63**	41	8E-05	0.25	3.19	94	13.3% : 10.6% (6/45 : 10/94)	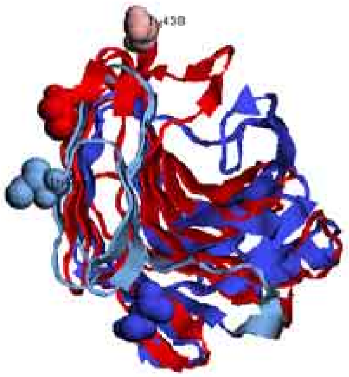

828	1h4x:A (111)	ANTI-SIGMA F FACTOR ANTAGONIST	**31**	33	0.021	0.22	3.25	78	12.9% : 11.5% (9/70 : 9/78)	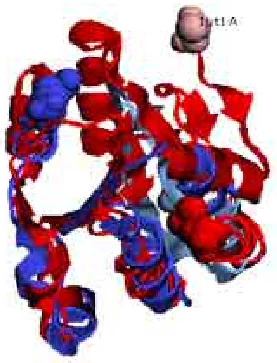
	1ytl:A (158)	Acetyl-CoA decarbonylase/synthase complex eps								
829	1jmw:A (146)	methyl-accepting chemotaxis protein II	**76**	41	8E-05	0.22	3.06	109	12.0% : 11.9% (6/50 : 13/109)	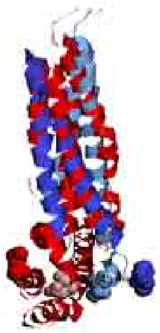
	1h6g:A (255)	ALPHA-1 CATENIN								
830	1rcc:_ (171)	L FERRITIN	**90**	35	0.004	0.24	3.50	63	6.8% : 6.3% (4/59 : 4/63)	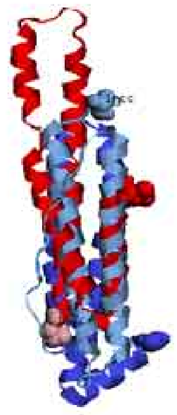
	1h7c:A (103)	TUBULIN-SPECIFIC CHAPERONE A								

831	1z7c:A (165)	Chorionic somatomammotropin hormone	**123**	31	0.088	0.39	2.00	64	3.4% : 7.8% (2/59 : 5/64)	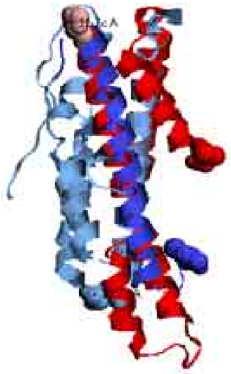
	1h7c:A (103)	TUBULIN-SPECIFIC CHAPERONE A								
832	2gts:A (77)	hypothetical protein HP0062	**38**	37	0.001	0.51	2.65	63	5.2% : 6.3% (3/58 : 4/63)	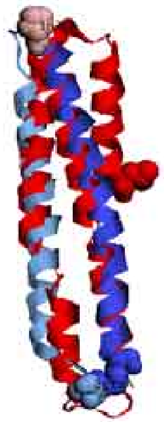
	1h7c:A (103)	TUBULIN-SPECIFIC CHAPERONE A								
833	2hjd:A (89)	Quorum-sensing antiactivator	**43**	30	0.09	0.26	1.34	53	3.2% : 1.9% (1/31 : 1/53)	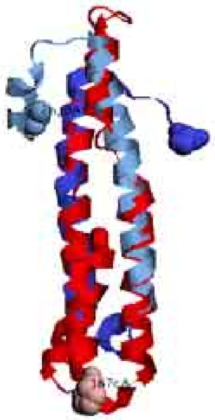
	1h7c:A (103)	TUBULIN-SPECIFIC CHAPERONE A								

834	* 1qok:A (227)	MFE-23 RECOMBINANT ANTIBODY FRAGMENT	121	167	1E-42	0.93	0.94	213	24.3% : 62.4% (45/185 : 133/213)	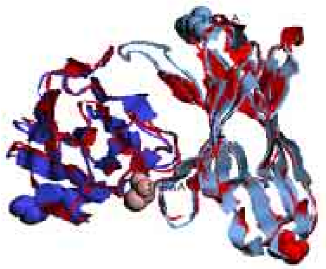
	* 1h8n:A (218)	MUTANT AL2 6E7S9G								
835	* 2ghw:B (233)	anti-sars scFv antibody, 80R	121	182	2E-47	0.90	1.06	213	28.3% : 57.3% (52/184 : 122/213)	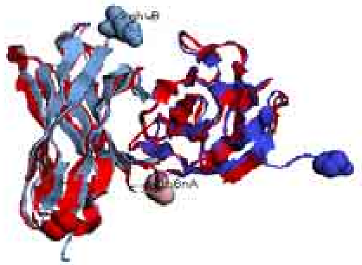
	* 1h8n:A (218)	MUTANT AL2 6E7S9G								
836	1rof:_ (60)	FERREDOXIN	32	32	0.026	0.28	1.91	39	21.1% : 20.5% (8/38 : 8/39)	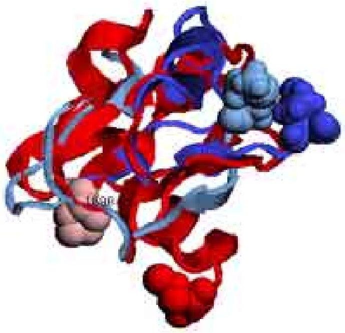
	1h98:A (77)	FERREDOXIN								

837	1nps:A (87)	DEVELOPMENT-SPECIFIC PROTEIN S	45	47	1E-06	0.80	1.59	79	25.0% : 15.2% (18/72 : 12/79)	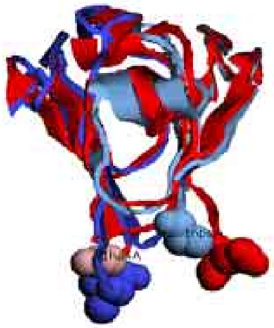
	1ha4:A (87)	GAMMA CRYSTALLIN S								
838	1he1:A (135)	EXOENZYME S	108	33	0.015	0.38	2.92	87	10.0% : 8.0% (5/50 : 7/87)	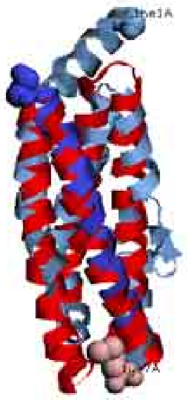
	1yo7:A (120)	Regulatory protein rop								
839	2b59:A (166)	COG1196: Chromosome segregation ATPases	105	32	0.037	0.21	3.16	60	2.4% : 10.0% (1/42 : 6/60)	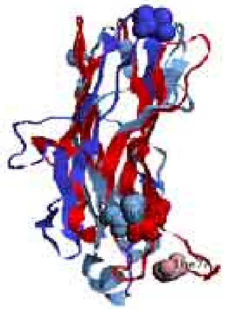
	1he7:A (107)	HIGH AFFINITY NERVE GROWTH FACTOR RECEPTOR								

840	1hg3:A (224)	TRIOSEPHOSPHATE ISOMERASE	**103**	58	9E-10	0.27	2.45	125	12.1% : 13.6% (15/124 : 17/125)	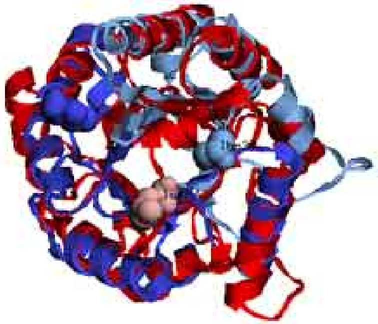
	1o66:A (250)	3-methyl-2-oxobutanoate hydroxymethyltransfer								
841	1hs7:A (97)	SYNTAXIN VAM3	**38**	34	0.007	0.36	2.69	74	12.3% : 6.8% (7/57 : 5/74)	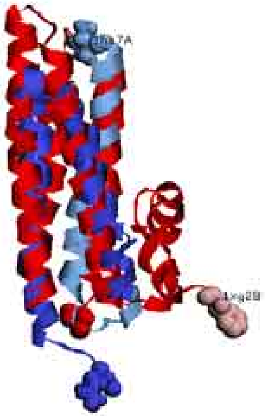
	1xg2:B (151)	Pectinesterase inhibitor								
842	1hs7:A (97)	SYNTAXIN VAM3	**36**	36	0.001	0.66	2.67	85	6.8% : 9.4% (5/73 : 8/85)	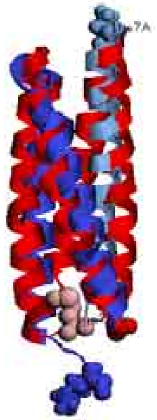
	1yo7:A (120)	Regulatory protein rop								

843	1hs7:A (97)	SYNTAXIN VAM3	**63**	35	0.004	0.39	2.36	64	6.5% : 18.8% (3/46 : 12/64)	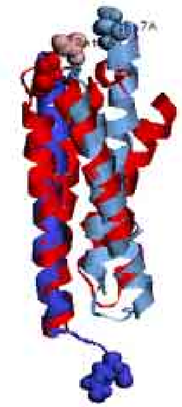
	2ets:A (115)	hypothetical protein								
844	1huq:A (164)	RAB5C	**61**	53	2E-08	0.24	2.80	123	12.5% : 17.9% (11/88 : 22/123)	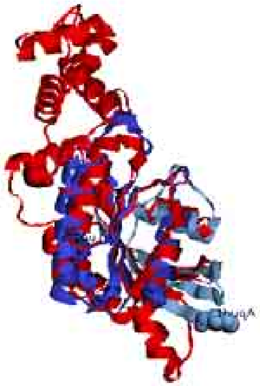
	1puj:A (262)	conserved hypothetical protein ylqF								
845	1hur:A (180)	HUMAN ADP-RIBOSYLATION FACTOR 1	**69**	41	0.0001	0.23	3.30	125	15.3% : 16.8% (13/85 : 21/125)	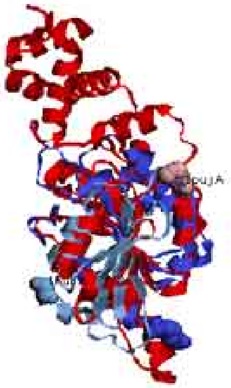
	1puj:A (262)	conserved hypothetical protein ylqF								

846	1hx1:B (112)	BAG-FAMILY MOLECULAR CHAPERONE REGULATOR-1	**82**	44	7E-06	0.33	2.55	79	5.2% : 7.6% (4/77 : 6/79)	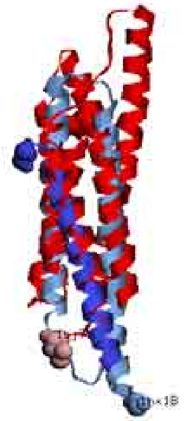
	1sj7:A (167)	Talin 1								
847	1xwm:A (212)	phosphate uptake regulator	**169**	39	0.0005	0.36	2.80	93	11.3% : 12.9% (9/80 : 12/93)	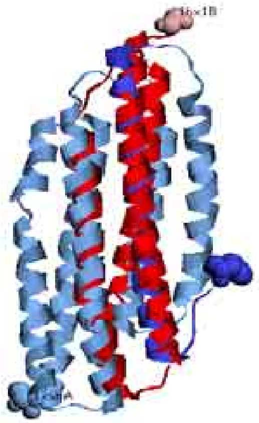
	1hx1:B (112)	BAG-FAMILY MOLECULAR CHAPERONE REGULATOR-1								
848	2ah6:A (152)	BH1595, unknown conserved protein	**77**	36	0.002	0.30	2.50	73	6.5% : 11.0% (4/62 : 8/73)	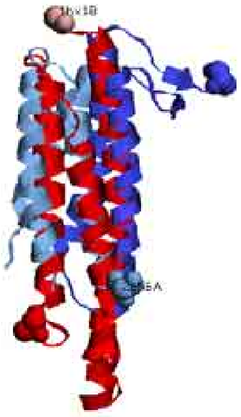
	1hx1:B (112)	BAG-FAMILY MOLECULAR CHAPERONE REGULATOR-1								

849	1hx1:B (112)	BAG-FAMILY MOLECULAR CHAPERONE REGULATOR-1	**43**	43	1E-05	0.45	2.96	92	2.4% : 9.8% (2/83 : 9/92)	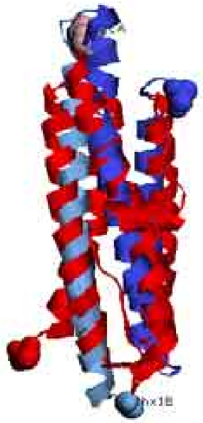
	2fjc:A (151)	Antigen TpF1								
850	2fzt:A (78)	hypothetical protein TM0693	**38**	42	3E-05	0.28	1.83	50	11.4% : 12.0% (5/44 : 6/50)	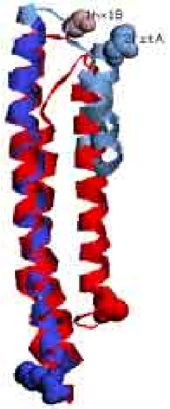
	1hx1:B (112)	BAG-FAMILY MOLECULAR CHAPERONE REGULATOR-1								
851	2gs4:A (159)	Protein yciF	**86**	44	6E-06	0.42	2.60	88	11.8% : 11.4% (10/85 : 10/88)	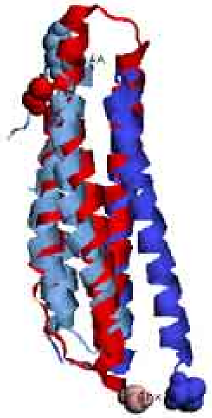
	1hx1:B (112)	BAG-FAMILY MOLECULAR CHAPERONE REGULATOR-1								

852	1i1r:B (167)	VIRAL IL-6	**113**	35	0.004	0.29	2.67	108	13.7% : 10.2% (7/51 : 11/108)	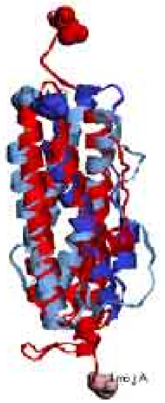
	1moj:A (180)	Dps-like ferritin								
853	1i1r:B (167)	VIRAL IL-6	**128**	40	0.0001	0.33	2.93	113	2.2% : 5.3% (1/45 : 6/113)	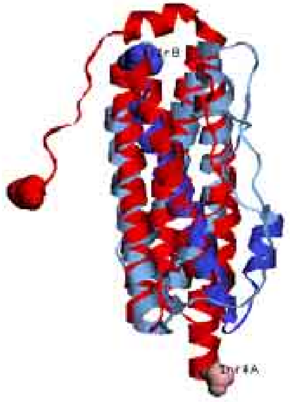
	1nf4:A (169)	bacterioferritin								
854	1i1r:B (167)	VIRAL IL-6	**51**	32	0.037	0.23	3.09	81	6.0% : 12.3% (3/50 : 10/81)	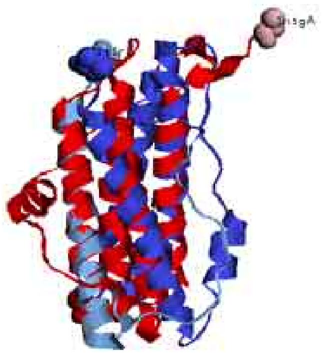
	1nig:A (147)	hypothetical protein TA1238								

855	1o9r:A (162)	AGROBACTERIUM TUMEFACIENS DPS	**48**	33	0.014	0.38	3.04	112	11.2% : 9.8% (10/89 : 11/112)	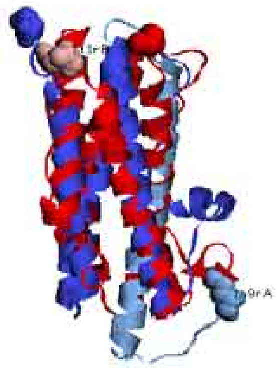
	1i1r:B (167)	VIRAL IL-6								
856	1i1r:B (167)	VIRAL IL-6	**63**	37	0.001	0.38	3.23	103	13.8% : 14.6% (9/65 : 15/103)	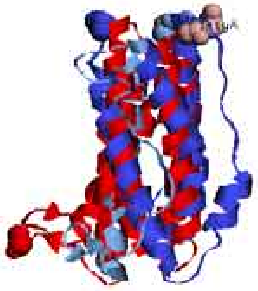
	1rty:A (159)	yvqk protein								
857	1i1r:B (167)	VIRAL IL-6	**118**	33	0.015	0.31	3.18	109	10.9% : 7.3% (10/92 : 8/109)	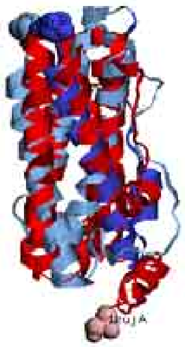
	1zuj:A (168)	hypothetical protein Llacc01001955								

858	1i1r:B (167)	VIRAL IL-6	**110**	33	0.022	0.29	3.25	106	7.4% : 8.5% (4/54 : 9/106)	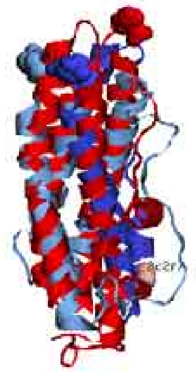
	2c2f:A (178)	DNA-BINDING STRESS RESPONSE PROTEIN								
859	2clb:A (169)	DPS-LIKE PROTEIN	**51**	40	0.0001	0.35	3.08	112	10.0% : 8.0% (5/50 : 9/112)	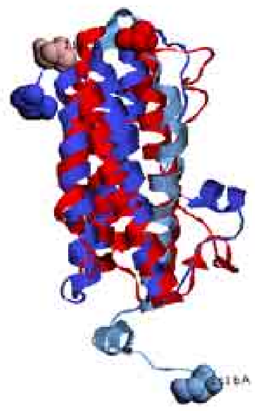
	1i1r:B (167)	VIRAL IL-6								
860	1i1r:B (167)	VIRAL IL-6	**130**	40	0.0002	0.45	3.26	120	9.3% : 5.8% (5/54 : 7/120)	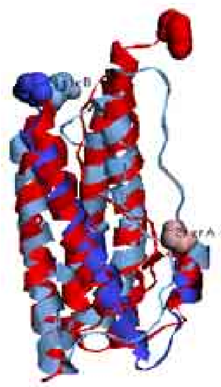
	2fzf:A (158)	hypothetical protein								

861	1i1r:B (167)	VIRAL IL-6	**143**	34	0.011	0.30	3.27	102	6.8% : 9.8% (5/74 : 10/102)	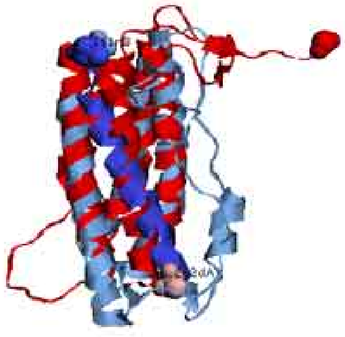
	2g2d:A (160)	ATP:cobalamin adenosyltransferase								
862	2cyj:A (118)	hypothetical protein PH1505	**85**	30	0.089	0.22	3.32	52	5.9% : 17.3% (2/34 : 9/52)	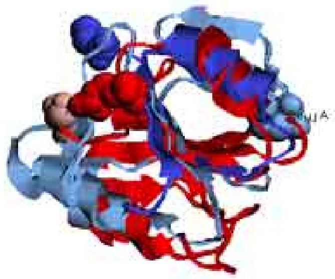
	1i3z:A (102)	EWS/FLI1 ACTIVATED TRANSCRIPT 2								
863	1n1q:A (149)	DPS Protein	**90**	48	5E-07	0.26	2.22	86	18.5% : 10.5% (15/81 : 9/86)	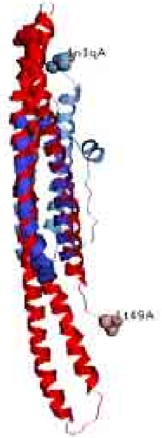
	1i49:A (201)	ARFAPTIN 2								

864	1sof:A (155)	Bacterioferritin	**89**	53	2E-08	0.22	2.24	90	7.3% : 13.3% (6/82 : 12/90)	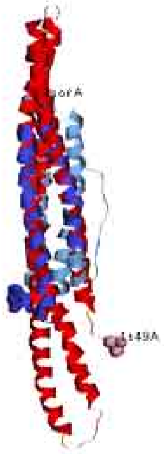
	1i49:A (201)	ARFAPTIN 2								
865	2d5k:A (148)	Dps family protein	**88**	46	2E-06	0.27	2.18	87	14.1% : 8.0% (12/85 : 7/87)	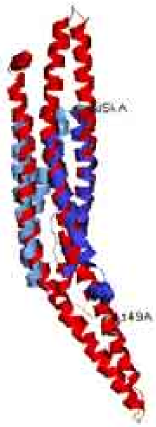
	1i49:A (201)	ARFAPTIN 2								
866	2fjc:A (151)	Antigen TpF1	**94**	48	6E-07	0.25	2.06	85	12.0% : 10.6% (10/83 : 9/85)	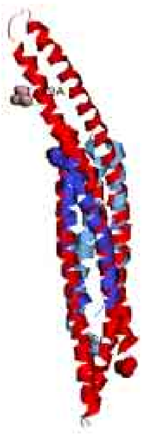
	1i49:A (201)	ARFAPTIN 2								

867	1l6w:A (220)	Fructose-6-phosphate aldolase 1	**52**	45	8E-06	0.37	3.34	169	10.3% : 10.1% (16/155 : 17/169)	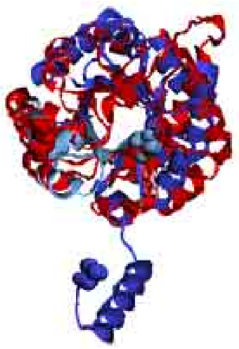
	1i4n:A (251)	INDOLE-3-GLYCEROL PHOSPHATE SYNTHASE								
868	1yo7:A (120)	Regulatory protein rop	**90**	42	3E-05	0.39	2.17	87	12.9% : 10.3% (8/62 : 9/87)	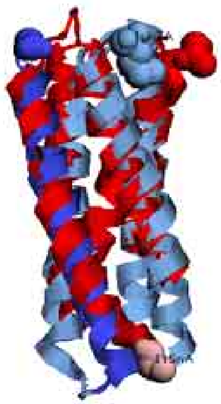
	1i5n:A (128)	CHEMOTAXIS PROTEIN CHEA								
869	1i5n:A (128)	CHEMOTAXIS PROTEIN CHEA	**49**	36	0.003	0.29	3.13	72	10.9% : 11.1% (7/64 : 8/72)	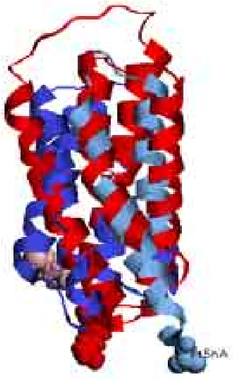
	2hfi:A (123)	Hypothetical protein yppE								

870	1i6z:A (135)	BAG-FAMILY MOLECULAR CHAPERONE REGULATOR-1	**51**	40	0.0002	0.23	1.93	86	16.5% : 7.0% (14/85 : 6/86)	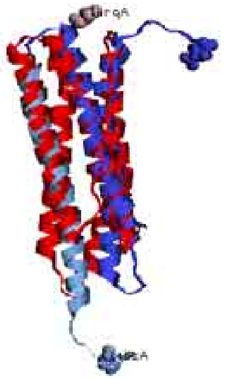
	1krq:A (164)	ferritin								
871	1uru:A (217)	AMPHIPHYSIN	**139**	44	1E-05	0.26	2.30	73	1.4% : 5.5% (1/69 : 4/73)	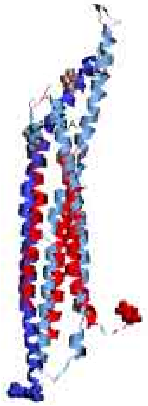
	1i6z:A (135)	BAG-FAMILY MOLECULAR CHAPERONE REGULATOR-1								
872	1vlg:A (164)	ferritin	**78**	40	0.0002	0.23	2.60	83	10.5% : 10.8% (6/57 : 9/83)	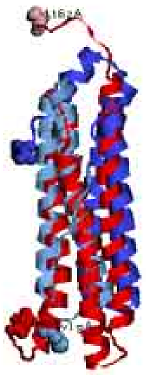
	1i6z:A (135)	BAG-FAMILY MOLECULAR CHAPERONE REGULATOR-1								

873	1lmi:A (131)	Immunogenic protein MPT63/MPB63	**84**	30	0.089	0.27	3.49	68	13.6% : 8.8% (8/59 : 6/68)	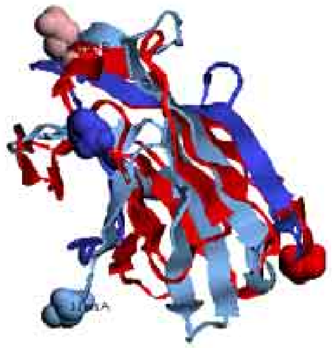
	1i8i:B (119)	EPIDERMAL GROWTH FACTOR RECEPTOR ANTIBODY MR1								
874	1ifq:A (127)	vesicle trafficking protein Sec22b	**111**	38	0.0006	0.28	2.38	91	10.7% : 9.9% (8/75 : 9/91)	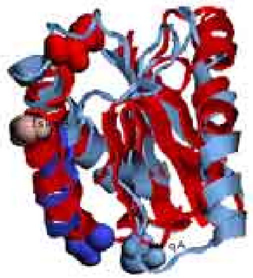
	1sko:A (119)	Mitogen-activated protein kinase kinase 1 int								
875	1igo:A (204)	family 11 xylanase	**63**	41	0.0001	0.23	3.22	90	21.7% : 15.6% (10/46 : 14/90)	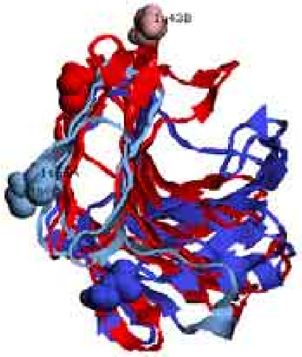
	1y43:B (171)	Aspergillopepsin II heavy chain								

876	1ihj:A (94)<br>2hga:A (103)	InaD<br>Conserved protein MTH1368	**33**	36	0.002	0.34	2.12	69	27.8% : 26.1%<br>(15/54 : 18/69)	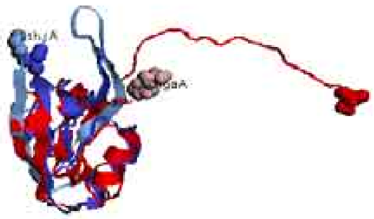
877	2bgo:A (116)<br>1im3:D (95)	ENDO-B1,4-MANNANASE 5C<br>cytomegalovirus protein US2	**50**	37	0.0007	0.24	2.88	52	11.1% : 9.6%<br>(5/45 : 5/52)	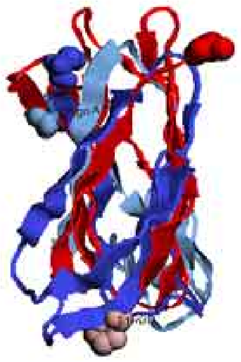
878	1mvq:A (236)<br>1ioa:A (228)	lectin, isoform 1<br>ARCELIN-5A	**123**	117	9E-28	0.81	1.34	209	31.7% : 36.4%<br>(32/101 : 76/209)	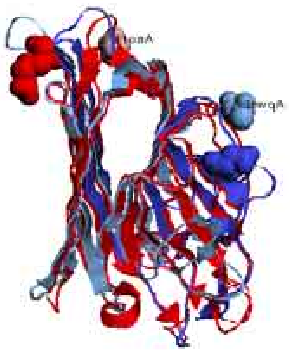

879	1ygt:A (104)<br>1iru:K (199)	Cytoplasmic dynein light chain<br>20S proteasome	**55**	32	0.036	0.27	3.42	61	10.4% : 9.8% (5/48 : 6/61)	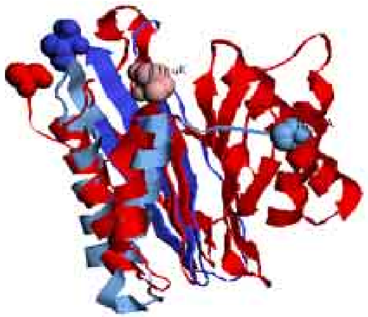
880	1is3:A (134)<br>1mvq:A (236)	CONGERIN II<br>lectin, isoform 1	**38**	43	1E-05	0.29	2.12	93	9.8% : 11.8% (6/61 : 11/93)	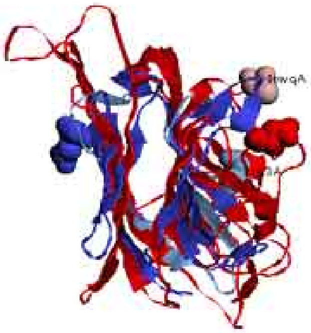
881	1is3:A (134)<br>2mcm_ (112)	CONGERIN II<br>MACROMOMYCIN	**57**	41	7E-05	0.23	3.40	59	9.8% : 13.6% (5/51 : 8/59)	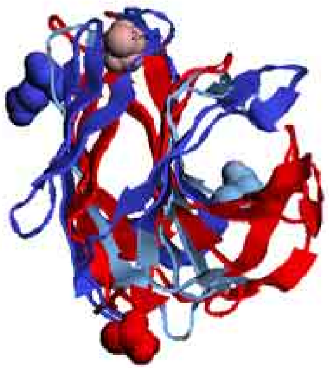

882	1vjx:A (148)	putative ferritin-like diiron-carboxylate pro	**42**	36	0.001	0.27	2.32	89	4.0% : 10.1% (2/50 : 9/89)	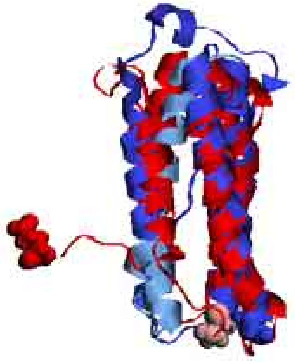
	1itf_ (165)	INTERFERON ALPHA-2A								
883	1iuz:_ (100)	PLASTOCYANIN	**57**	30	0.088	0.25	3.38	50	9.8% : 8.0% (4/41 : 4/50)	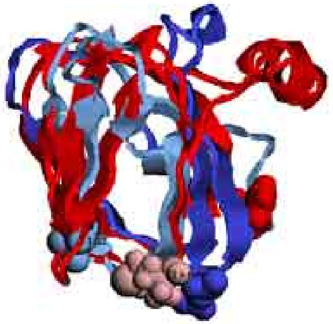
	1lkn:A (89)	hypothetical protein tm1112								
884	2d5k:A (148)	Dps family protein	**59**	37	0.001	0.22	3.26	85	1.7% : 3.5% (1/60 : 3/85)	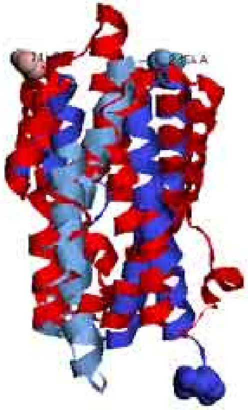
	1iw0:A (207)	Heme oxygenase								

885	1jh5:A (144)	TUMOR NECROSIS FACTOR LIGAND SUPERFAMILY MEMB	**116**	33	0.021	0.25	3.40	60	11.9% : 6.7% (5/42 : 4/60)	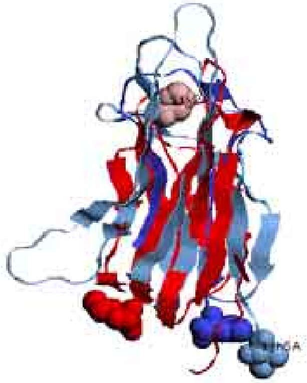
	1ix2:A (102)	PcoC copper resistance protein								
886	1viz:A (226)	PcrB protein homolog	**140**	35	0.009	0.28	3.03	134	13.2% : 9.0% (17/129 : 12/134)	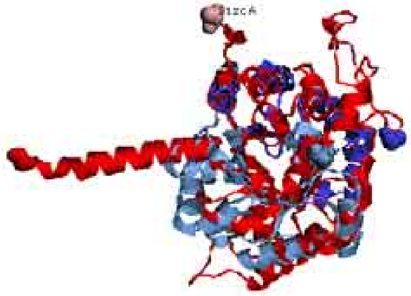
	1izc:A (299)	macrophomate synthase intermolecular Diels-Al								
887	1mvq:A (236)	lectin, isoform 1	**148**	38	0.001	0.25	3.31	125	4.5% : 8.8% (3/66 : 11/125)	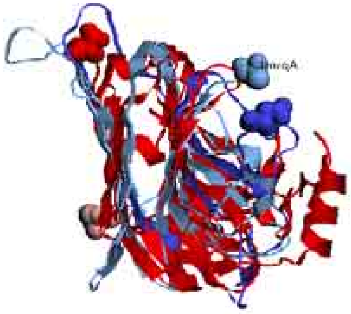
	1j1t:A (228)	Alginate Lyase								

888	1j27:A (98)	hypothetical protein TT1725	**52**	32	0.044	0.33	2.95	52	15.0% : 7.7% (6/40 : 4/52)	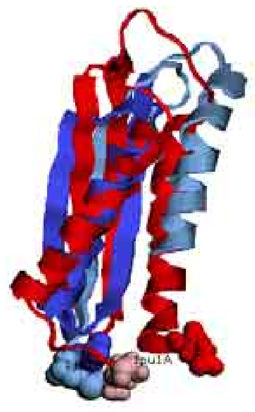
	1pu1:A (91)	Hypothetical protein MTH677								
889	1jaf:A (128)	CYTOCHROME C'	**43**	36	0.002	0.29	2.86	94	10.8% : 11.7% (8/74 : 11/94)	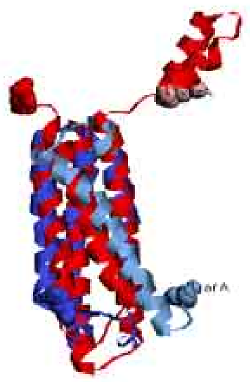
	1x8z:A (151)	invertase/pectin methylesterase inhibitor fam								
890	1jbj:A (186)	CD3 Epsilon and gamma Ectodomain Fragment Com	**105**	50	1E-07	0.58	2.24	130	26.1% : 59.2% (30/115 : 77/130)	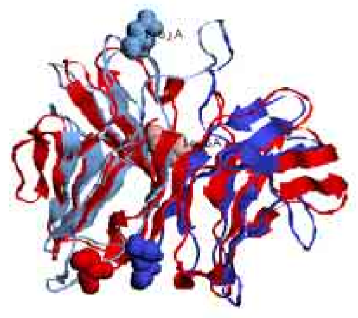
	1sy6:A (169)	T-cell surface glycoprotein CD3 gamma/epsilon								

891	1jf8:A (130)	arsenate reductase	**45**	34	0.007	0.23	2.72	73	11.1% : 11.0% (5/45 : 8/73)	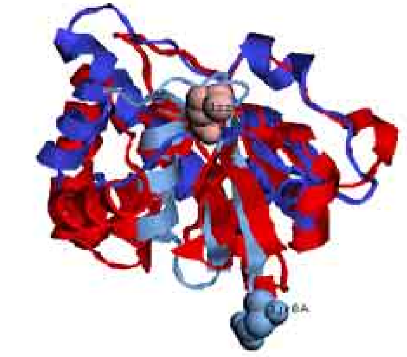
	1zzw:A (147)	Dual specificity protein phosphatase 10								
892	1jf8:A (130)	arsenate reductase	**37**	38	0.0006	0.20	2.47	73	15.1% : 13.7% (8/53 : 10/73)	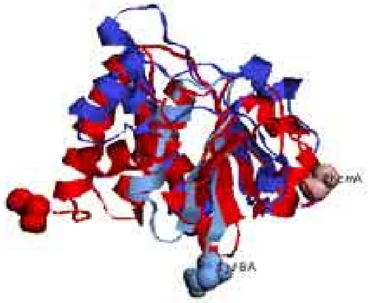
	2hcm:A (159)	Dual specificity protein phosphatase								
893	1jgc:A (160)	bacterioferritin	**36**	42	4E-05	0.21	1.94	79	1.6% : 3.8% (1/62 : 3/79)	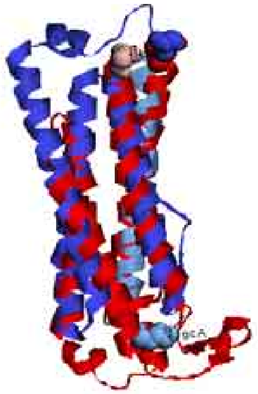
	1yjg:A (157)	Surface protein VspA								

894	1jgc:A (160)	bacterioferritin	87	42	5E-05	0.37	2.16	95	5.4% : 11.6% (5/93 : 11/95)	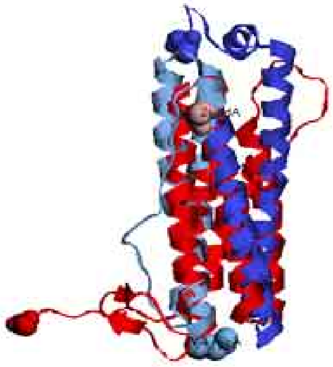
	2g2d:A (160)	ATP:cobalamin adenosyltransferase								
895	1lwr:A (96)	Neural Cell Adhesion Molecule 1, 140 kDa isof	46	36	0.002	0.22	3.42	56	6.4% : 8.9% (3/47 : 5/56)	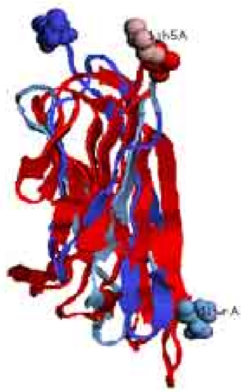
	1jh5:A (144)	TUMOR NECROSIS FACTOR LIGAND SUPERFAMILY MEMB								
896	1owa:A (156)	Spectrin alpha chain, erythrocyte	91	37	0.001	0.24	2.92	85	8.5% : 16.5% (7/82 : 14/85)	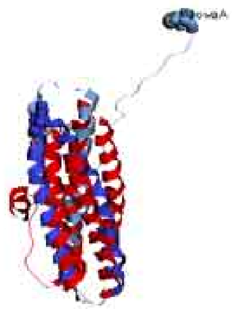
	1ji4:A (144)	NEUTROPHIL-ACTIVATING PROTEIN A								

897	1jju:B<br>(337)<br><br>1nex:B<br>(451)	QUINOHEMOPROTEIN AMINE DEHYDROGENASE<br><br>CDC4 protein	**216**	77	2E-15	0.27	2.75	222	12.8% : 9.0%<br>(28/218 : 20/222)	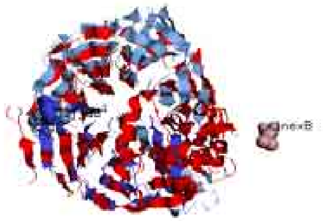
898	1jju:B<br>(337)<br><br>2cnx:A<br>(306)	QUINOHEMOPROTEIN AMINE DEHYDROGENASE<br><br>WD-REPEAT PROTEIN 5	**224**	99	3E-22	0.59	3.18	251	14.2% : 10.4%<br>(30/211 : 26/251)	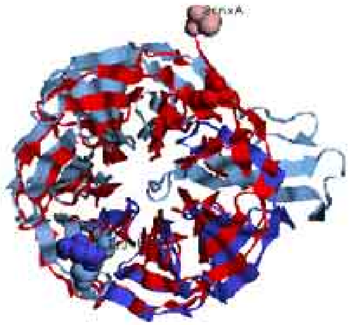
899	*<br>1kal:_<br>(29)<br><br>*<br>1jjz:A<br>(29)	KALATA B1<br><br>KALATA B1	**8**	63	1E-11	0.77	1.11	29	100.0% : 100.0%<br>(21/21 : 29/29)	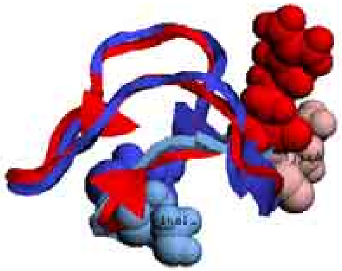

900	* 1jjz:A (29)	KALATA B1	6	57	1E-09	0.61	1.56	29	91.7% : 93.1% (22/24 : 27/29)	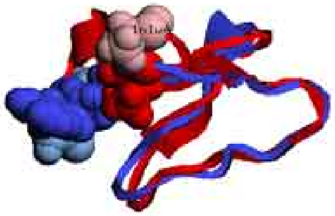
	* 1n1u:A (29)	kalata B1								
901	* 1jjz:A (29)	KALATA B1	6	59	3E-10	0.61	0.83	29	82.6% : 82.8% (19/23 : 24/29)	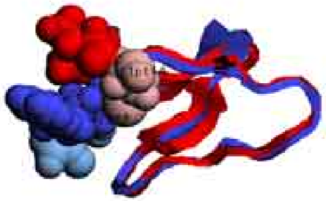
	* 1pt4:A (29)	kalata B2								
902	* 1jjz:A (29)	KALATA B1	6	38	0.0006	0.47	1.85	28	65.2% : 60.7% (15/23 : 17/28)	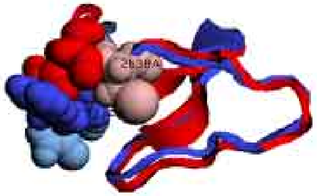
	* 2b38:A (31)	kalata B8								

903	* 1jjz:A (29)	KALATA B1	**6**	59	3E-10	0.61	1.45	29	91.3% : 93.1% (21/23 : 27/29)	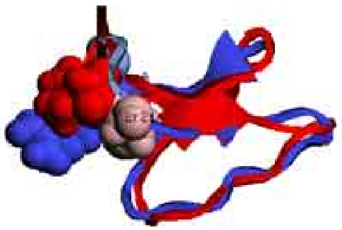
	* 2f2i:A (29)	Kalata-B1								
904	1x1r:A (169)	Ras-related protein M-Ras	**76**	32	0.054	0.23	3.38	84	6.2% : 14.3% (4/65 : 12/84)	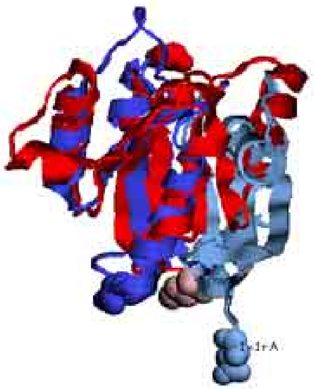
	1jmv:A (140)	Universal Stress Protein A								
905	* 2ged:A (172)	Signal recognition particle receptor beta sub	**64**	31	0.078	0.25	3.42	76	18.1% : 13.2% (13/72 : 10/76)	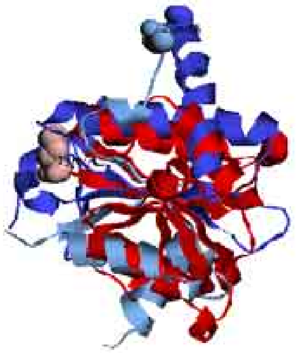
	* 1jmv:A (140)	Universal Stress Protein A								

906	1owa:A (156)	Spectrin alpha chain, erythrocyte	**119**	34	0.011	0.28	3.42	83	11.7% : 8.4% (7/60 : 7/83)	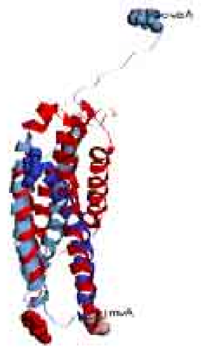
	1jmw:A (146)	methyl-accepting chemotaxis protein II								
907	1jmw:A (146)	methyl-accepting chemotaxis protein II	**70**	58	6E-10	0.38	2.80	111	4.8% : 9.0% (5/105 : 10/111)	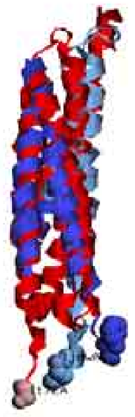
	1t72:A (215)	Phosphate transport system protein phoU homol								
908	1jmw:A (146)	methyl-accepting chemotaxis protein II	**50**	38	0.0004	0.30	2.74	105	10.5% : 9.5% (9/86 : 10/105)	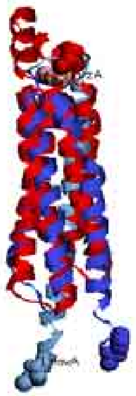
	1x8z:A (151)	invertase/pectin methylesterase inhibitor fam								

909	1jmw:A (146)	methyl-accepting chemotaxis protein II	**77**	52	3E-08	0.48	3.28	124	8.3% : 9.7% (9/109 : 12/124)	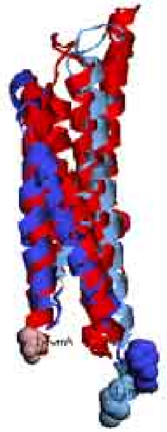
	1xwm:A (212)	phosphate uptake regulator								
910	1jmw:A (146)	methyl-accepting chemotaxis protein II	**48**	36	0.002	0.53	2.90	104	10.1% : 9.6% (8/79 : 10/104)	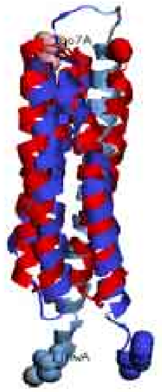
	1yo7:A (120)	Regulatory protein rop								
911	1jmw:A (146)	methyl-accepting chemotaxis protein II	**76**	37	0.001	0.45	3.21	90	10.7% : 14.4% (8/75 : 13/90)	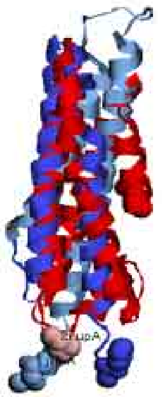
	2fup:A (129)	hypothetical protein PA3352								

912	2g2d:A (160)	ATP:cobalamin adenosyltransferase	**82**	32	0.042	0.26	2.92	90	7.0% : 10.0% (5/71 : 9/90)	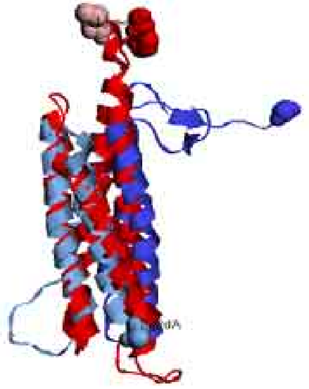
	1jmw:A (146)	methyl-accepting chemotaxis protein II								
913	1jmw:A (146)	methyl-accepting chemotaxis protein II	**119**	35	0.004	0.23	2.45	80	6.6% : 13.8% (4/61 : 11/80)	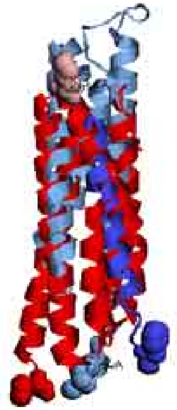
	2gs4:A (159)	Protein yciF								
914	1rgv:A (80)	ferredoxin	**29**	32	0.044	0.21	1.92	62	27.6% : 29.0% (16/58 : 18/62)	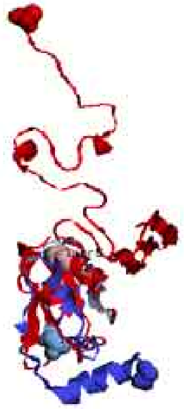
	1jnr:B (149)	adenylylsulfate reductase								

915	1qok:A (227)	MFE-23 RECOMBINANT ANTIBODY FRAGMENT	**122**	175	5E-45	0.91	1.05	220	28.1% : 50.5% (54/192 : 111/220)	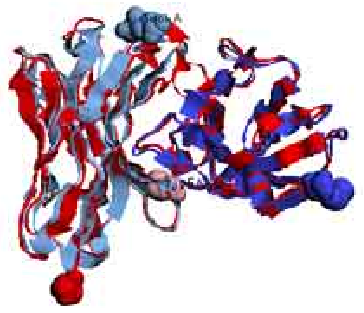
	1jp5:A (233)	single-chain Fv fragment 1696								
916	2ghw:B (233)	anti-sars scFv antibody, 80R	**122**	159	2E-40	0.84	1.09	215	29.6% : 56.7% (56/189 : 122/215)	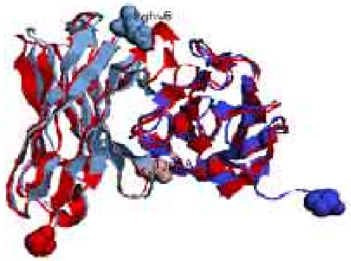
	1jp5:A (233)	single-chain Fv fragment 1696								
917	1jpx:A (60)	gp41 envelope protein	**34**	32	0.044	0.40	2.55	39	18.5% : 10.3% (5/27 : 4/39)	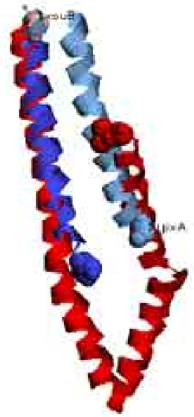
	1xou:B (84)	Z5138 gene product								

918	1jpx:A (60)	gp41 envelope protein	**28**	33	0.021	0.45	2.45	53	0.0% : 11.3% (0/30 : 6/53)	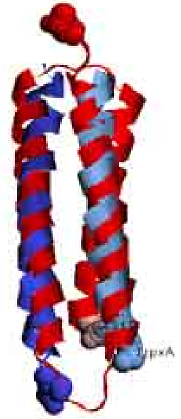
	2c5i:T (94)	T-SNARE AFFECTING A LATE GOLGI COMPARTMENT PR								
919	1q0d:A (117)	Superoxide dismutase [Ni]	**79**	38	0.0004	0.28	1.70	77	6.2% : 6.5% (4/65 : 5/77)	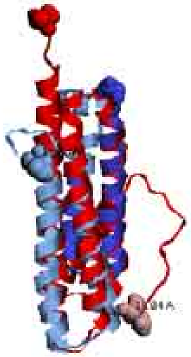
	1k04:A (142)	FOCAL ADHESION KINASE 1								
920	1r0d:A (194)	Huntingtin Interacting Protein 12	**89**	45	7E-06	0.31	1.89	96	11.8% : 9.4% (10/85 : 9/96)	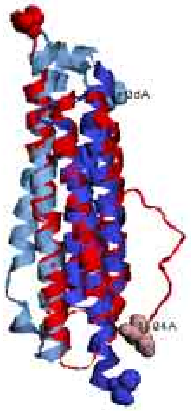
	1k04:A (142)	FOCAL ADHESION KINASE 1								

921	1vjx:A (148)	putative ferritin-like diiron-carboxylate pro	**80**	41	8E-05	0.28	2.93	88	9.7% : 6.8% (7/72 : 6/88)	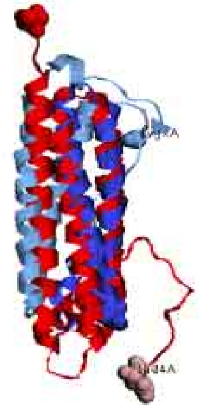
	1k04:A (142)	FOCAL ADHESION KINASE 1								
922	2gs4:A (159)	Protein yciF	**90**	44	9E-06	0.27	2.08	88	9.7% : 10.2% (7/72 : 9/88)	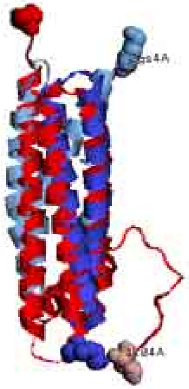
	1k04:A (142)	FOCAL ADHESION KINASE 1								
923	1m62:A (87)	BAG-family molecular chaperone regulator-4	**64**	35	0.004	0.43	3.19	67	11.1% : 13.4% (5/45 : 9/67)	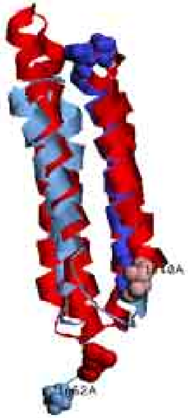
	1k40:A (126)	adhesion kinase								

924	1r0d:A (194)	Huntingtin Interacting Protein 12	**99**	42	5E-05	0.47	1.78	113	7.3% : 10.6% (6/82 : 12/113)	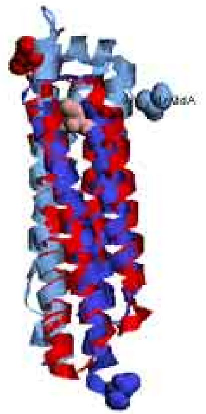
	1k40:A (126)	adhesion kinase								
925	1k40:A (126)	adhesion kinase	**60**	40	0.0002	0.49	3.48	108	17.8% : 13.9% (13/73 : 15/108)	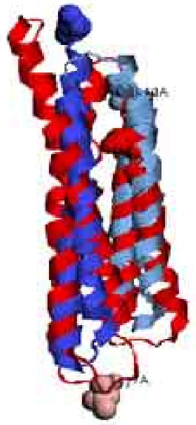
	1sj7:A (167)	Talin 1								
926	1wrd:A (98)	Target of Myb protein 1	**73**	53	2E-08	0.47	3.35	79	11.9% : 6.3% (7/59 : 5/79)	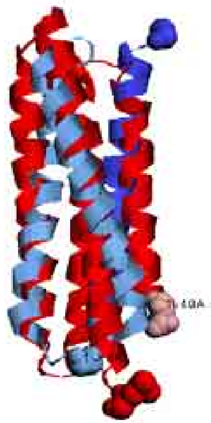
	1k40:A (126)	adhesion kinase								

927	1k40:A (126)	adhesion kinase	**29**	44	8E-06	0.39	2.97	108	10.8% : 12.0% (9/83 : 13/108)	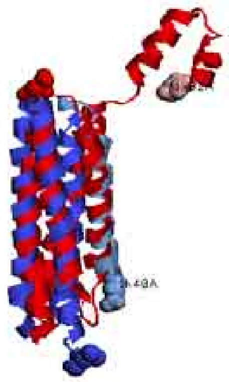
	1x8z:A (151)	invertase/pectin methylesterase inhibitor fam								
928	2d5k:A (148)	Dps family protein	**93**	43	2E-05	0.27	2.61	79	3.6% : 6.3% (2/56 : 5/79)	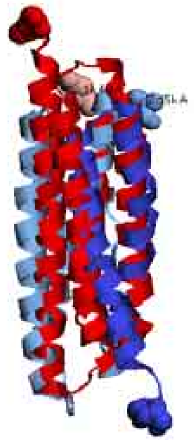
	1k40:A (126)	adhesion kinase								
929	* 1k76:A (60)	SEX MUSCLE ABNORMAL PROTEIN 5	**16**	43	1E-05	0.62	1.71	55	36.6% : 34.5% (15/41 : 19/55)	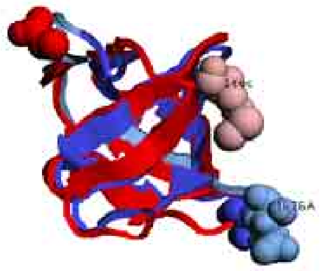
	* 1tuc_ (65)	ALPHA-SPECTRIN								

930	1mda:H<br>(368)<br>1k8k:C<br>(356)	METHYLAMINE DEHYDROGENASE (HEAVY SUBUNIT)<br>ARP2/3 COMPLEX 41 KDA SUBUNIT	**275**	47	3E-06	0.31	3.20	224	9.0% : 7.1%<br>(19/211 : 16/224)	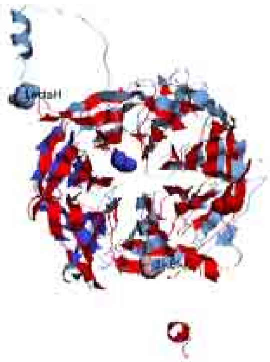
931	*<br>1kal:_<br>(29)<br>*<br>1n1u:A<br>(29)	KALATA B1<br>kalata B1	**13**	57	1E-09	0.98	1.63	29	88.2% : 93.1%<br>(15/17 : 27/29)	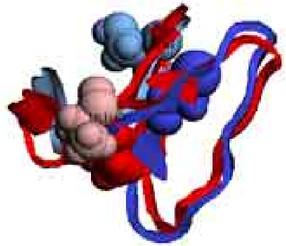
932	*<br>1kal:_<br>(29)<br>*<br>1pt4:A<br>(29)	KALATA B1<br>kalata B2	**13**	59	3E-10	0.98	0.92	29	88.2% : 82.8%<br>(15/17 : 24/29)	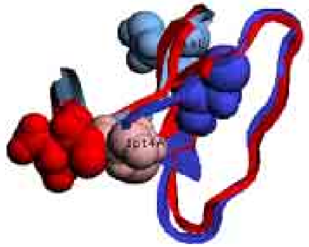

933	* 1kal:_ (29)	KALATA B1	**13**	34	0.011	0.49	1.54	24	62.5% : 50.0% (10/16 : 12/24)	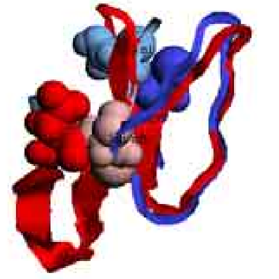
	* 1yp8:A (33)	tricyclon A								
934	* 1kal:_ (29)	KALATA B1	**13**	38	0.0006	0.84	1.95	28	73.3% : 60.7% (11/15 : 17/28)	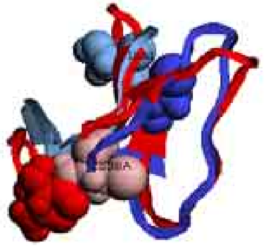
	* 2b38:A (31)	kalata B8								
935	* 1kal:_ (29)	KALATA B1	**13**	59	3E-10	0.98	1.57	29	100.0% : 93.1% (17/17 : 27/29)	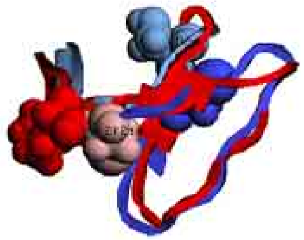
	* 2f2i:A (29)	Kalata-B1								

936	* 1kal:_ (29)	KALATA B1	**9**	36	0.003	0.56	2.14	23	6.3% : 60.9% (1/16 : 14/23)	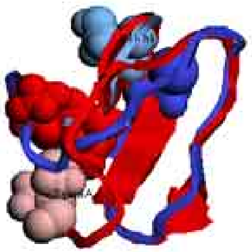
	* 2fqa:A (27)	violacin 1								
937	* 2gj0:A (31)	Cycloviolacin O14	**24**	41	5E-05	0.69	1.87	28	58.8% : 50.0% (10/17 : 14/28)	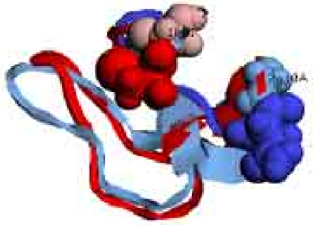
	* 1kal_ (29)	KALATA B1								
938	1kao:_ (167)	RAP2A	**60**	40	0.0001	0.21	2.88	120	11.6% : 17.5% (10/86 : 21/120)	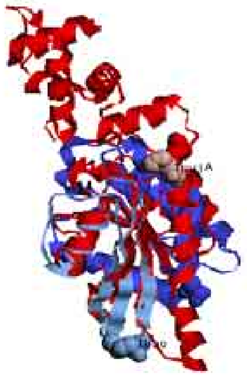
	1puj:A (262)	conserved hypothetical protein ylqF								

939	1mvq:A (236)	lectin, isoform 1	**118**	37	0.002	0.23	3.37	100	5.8% : 8.0% (4/69 : 8/100)	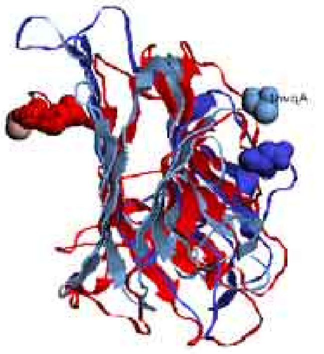
	1kgy:A (181)	EPHRIN TYPE-B RECEPTOR 2								
940	2gr8:A (78)	Adhesin	**53**	31	0.074	0.29	2.90	37	4.0% : 10.8% (1/25 : 4/37)	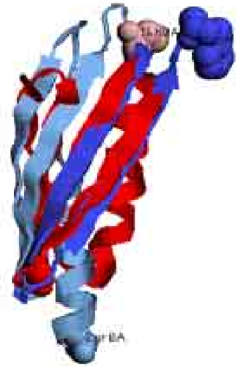
	1kh0:A (65)	protein L								
941	1kjn:A (152)	MTH0777	**70**	37	0.0008	0.34	3.44	112	12.8% : 8.0% (14/109 : 9/112)	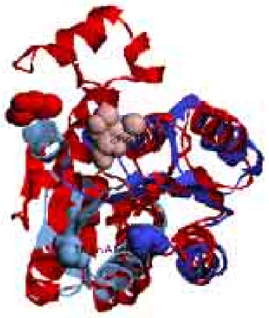
	2hq1:A (223)	Glucose/ribitol dehydrogenase								

942	1krq:A (164)	ferritin	51	43	2E-05	0.22	2.75	79	8.6% : 2.5% (6/70 : 2/79)	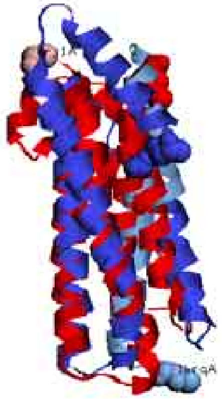
	1rj1:A (148)	invertase inhibitor								
943	1krq:A (164)	ferritin	87	40	0.0001	0.36	2.77	96	2.1% : 6.3% (2/94 : 6/96)	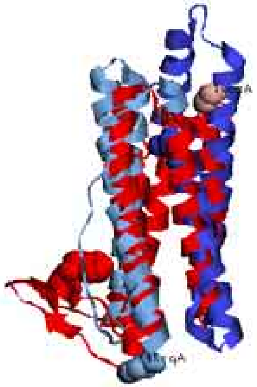
	1woz:A (159)	177aa long conserved hypothetical protein (ST								
944	1krq:A (164)	ferritin	52	41	6E-05	0.25	3.37	87	11.1% : 8.0% (8/72 : 7/87)	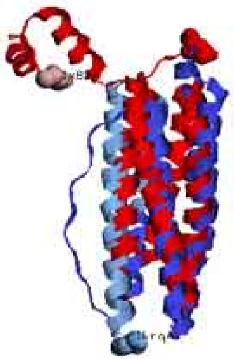
	1x8z:A (151)	invertase/pectin methylesterase inhibitor fam								

945	1krq:A (164)	ferritin	**51**	40	0.0002	0.24	2.08	77	9.3% : 2.6% (7/75 : 2/77)	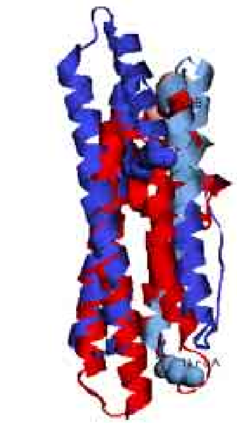
	1xg2:B (151)	Pectinesterase inhibitor								
946	1yjg:A (157)	Surface protein VspA	**123**	51	7E-08	0.22	2.45	83	3.5% : 4.8% (2/57 : 4/83)	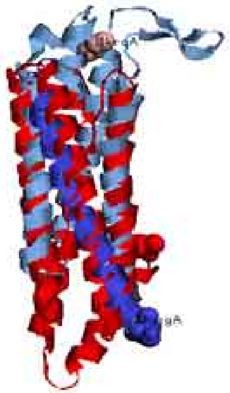
	1krq:A (164)	ferritin								
947	1z23:A (163)	CRK-associated substrate	**87**	34	0.01	0.33	2.81	94	3.5% : 8.5% (2/57 : 8/94)	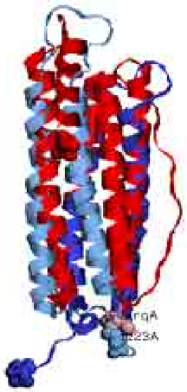
	1krq:A (164)	ferritin								

948	1ksg:A (180)	arf-like protein 2	**70**	47	1E-06	0.23	3.07	127	15.3% : 18.9% (13/85 : 24/127)	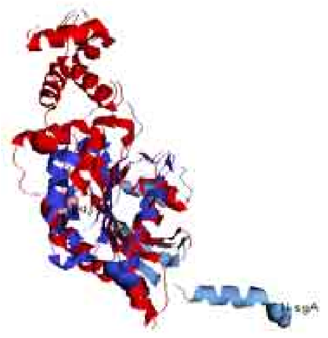
	1puj:A (262)	conserved hypothetical protein ylqF								
949	1ky2:A (180)	GTP-BINDING PROTEIN YPT7P	**75**	57	1E-09	0.21	3.06	122	13.6% : 18.9% (12/88 : 23/122)	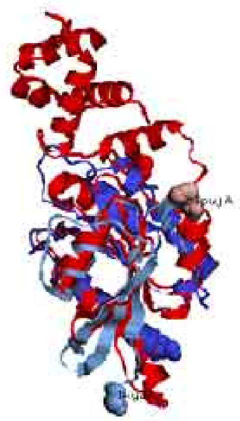
	1puj:A (262)	conserved hypothetical protein ylqF								
950	1kyv:A (147)	6,7-Dimethyl-8-ribityllumazine Synthase	**67**	31	0.074	0.42	3.27	89	9.9% : 6.7% (7/71 : 6/89)	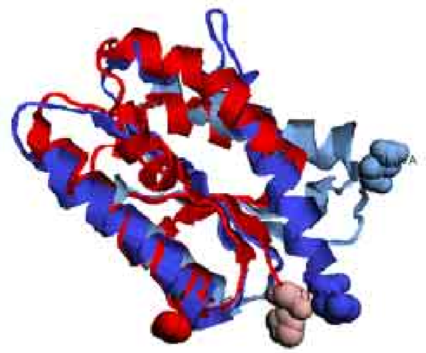
	1t1j:A (119)	hypothetical protein								

951	1l3p:A (102)	POLLEN ALLERGEN Phl p 5b	**49**	52	4E-08	0.66	2.42	86	32.5% : 27.9% (27/83 : 24/86)	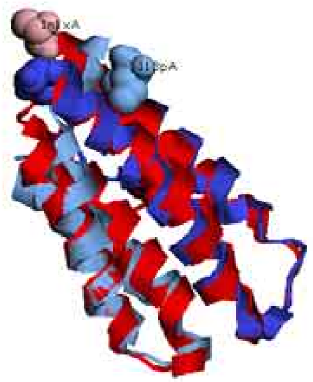
	1nlx:A (104)	Pollen allergen Phl p 6								
952	1l3p:A (102)	POLLEN ALLERGEN Phl p 5b	**77**	35	0.004	0.29	2.28	49	4.3% : 0.0% (2/47 : 0/49)	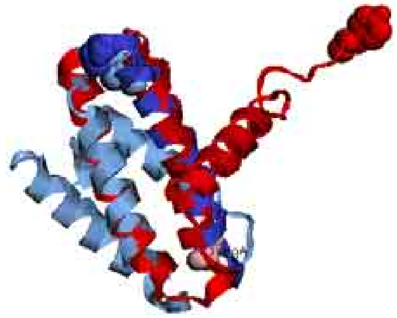
	1xeq:A (89)	Nonstructural protein NS1								
953	1l6o:A (95)	Segment polarity protein dishevelled homolog	**27**	31	0.063	0.37	2.31	70	20.0% : 21.4% (11/55 : 15/70)	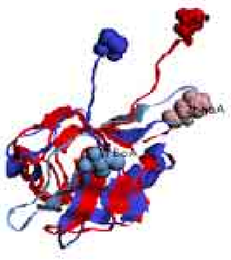
	2hga:A (103)	Conserved protein MTH1368								

954	1rv8:A (298)	fructose-1,6-bisphosphate aldolase	**214**	50	2E-07	0.25	3.45	154	9.8% : 12.3% (15/153 : 19/154)	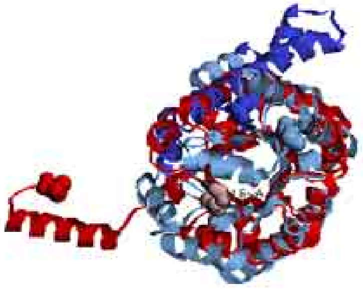
	1l6w:A (220)	Fructose-6-phosphate aldolase 1								
955	1v93:A (292)	5,10-Methylenetetrahydrofolate reductase	**88**	40	0.0004	0.25	3.38	149	11.9% : 12.1% (16/134 : 18/149)	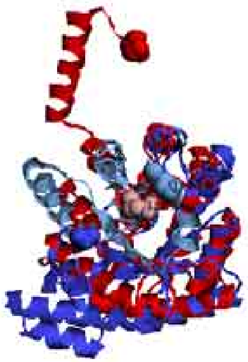
	1l6w:A (220)	Fructose-6-phosphate aldolase 1								
956	1lmi:A (131)	Immunogenic protein MPT63/MPB63	**85**	32	0.03	0.25	3.43	67	14.5% : 13.4% (8/55 : 9/67)	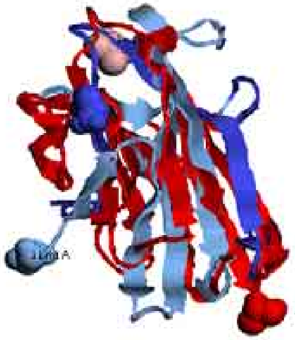
	1ri8:A (124)	camelid ANTIBODY HEAVY CHAIN								

957	1lmi:A (131)	Immunogenic protein MPT63/MPB63	**91**	32	0.036	0.28	3.37	61	6.3% : 11.5% (2/32 : 7/61)	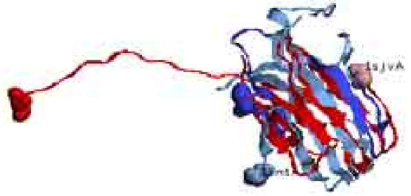
	1sjv:A (103)	r9								
958	1lvf:A (106)	syntaxin 6	**41**	44	1E-05	0.37	2.92	85	7.1% : 5.9% (6/84 : 5/85)	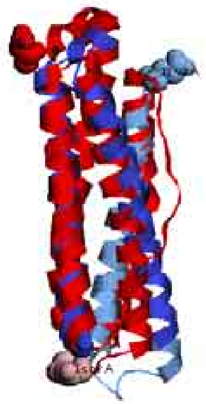
	1sof:A (155)	Bacterioferritin								
959	1wpa:A (107)	Occludin	**78**	41	8E-05	0.36	2.12	66	5.0% : 7.6% (3/60 : 5/66)	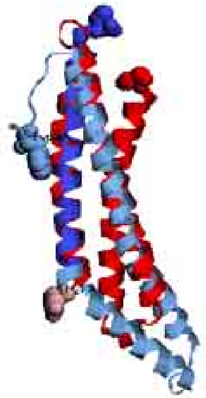
	1lvf:A (106)	syntaxin 6								

960	2ieq:A (89)	Spike glycoprotein	**70**	39	0.0002	0.28	3.43	67	6.7% : 7.5% (2/30 : 5/67)	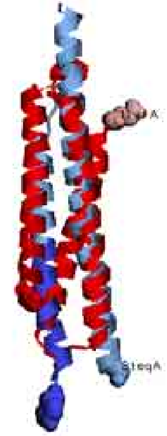
	1lvf:A (106)	syntaxin 6								
961	1lwr:A (96)	Neural Cell Adhesion Molecule 1, 140 kDa isof	**52**	42	2E-05	0.21	2.84	58	7.5% : 8.6% (3/40 : 5/58)	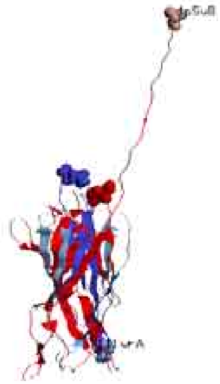
	1p5u:B (149)	F1 capsule antigen								
962	1usm:A (78)	HEPATOCYTE NUCLEAR FACTOR 1-ALPHA	**45**	34	0.006	0.44	3.47	63	6.0% : 6.3% (3/50 : 4/63)	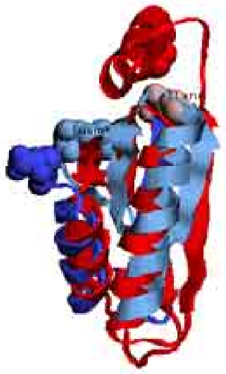
	1lxn:A (99)	HYPOTHETICAL PROTEIN MTH1187								

963	1lyx:A (246)	Triosephosphate Isomerase	**105**	32	0.06	0.22	3.15	151	10.6% : 9.3% (14/132 : 14/151)	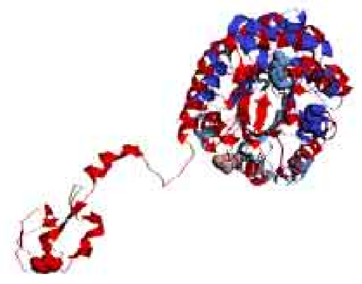
	1xuu:A (348)	polysialic acid capsule biosynthesis protein								
964	* 2d80:A (318)	PHB depolymerase	**175**	32	0.079	0.25	2.80	164	21.2% : 17.1% (21/99 : 28/164)	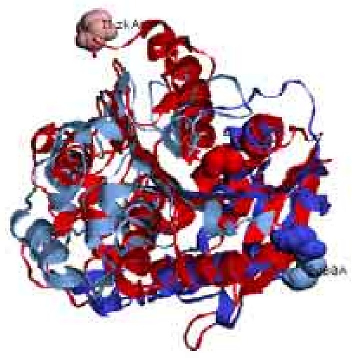
	* 1lzk:A (317)	HEROIN ESTERASE								
965	1qx2:A (76)	Vitamin D-dependent calcium-binding protein,	**25**	32	0.031	0.52	2.86	58	51.9% : 13.8% (27/52 : 8/58)	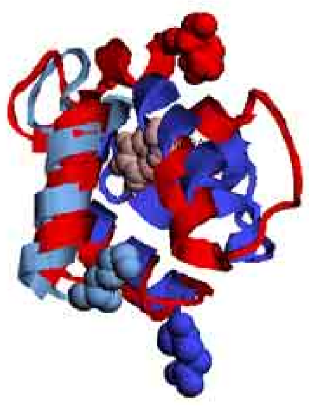
	1m39:A (80)	Caltractin, isoform 1								

966	1m62:A (87)	BAG-family molecular chaperone regulator-4	**57**	32	0.03	0.44	3.36	66	12.8% : 9.1% (6/47 : 6/66)	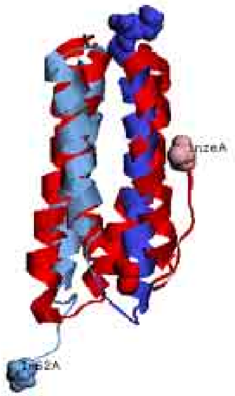
	1nze:A (112)	Oxygen-evolving enhancer protein 3								
967	1m62:A (87)	BAG-family molecular chaperone regulator-4	**60**	38	0.0004	0.37	1.75	68	2.1% : 10.3% (1/48 : 7/68)	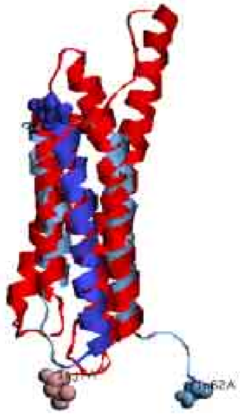
	1sj7:A (167)	Talin 1								
968	1mda:H (368)	METHYLAMINE DEHYDROGENASE (HEAVY SUBUNIT)	**143**	40	0.0003	0.37	3.47	193	8.7% : 13.5% (15/172 : 26/193)	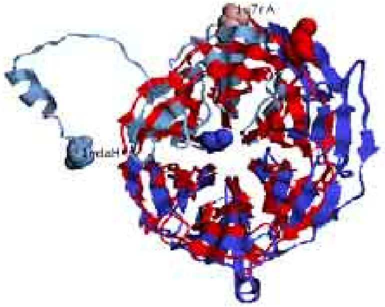
	1q7f:A (279)	brain tumor CG10719-PA								

969	1mfg:A (95)	Erb-B2 INTERACTING PROTEIN	**33**	32	0.044	0.30	2.22	70	30.9% : 27.1% (17/55 : 19/70)	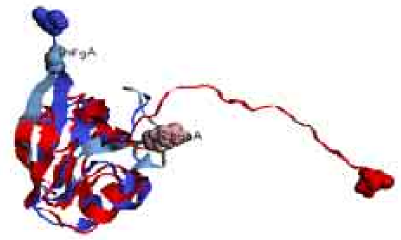
	2hga:A (103)	Conserved protein MTH1368								
970	1moz:A (179)	ADP-ribosylation factor-like protein 1	**67**	48	6E-07	0.21	3.16	123	12.0% : 17.1% (10/83 : 21/123)	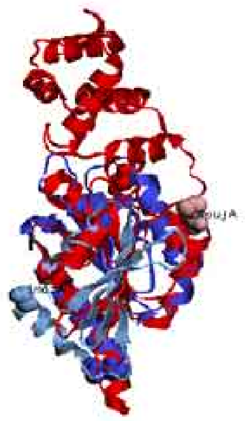
	1puj:A (262)	conserved hypothetical protein ylqF								
971	1mr3:F (177)	ADP-ribosylation factor 2	**66**	38	0.0007	0.21	3.15	115	16.0% : 17.4% (12/75 : 20/115)	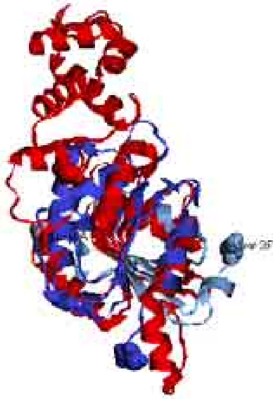
	1puj:A (262)	conserved hypothetical protein ylqF								

972	* 1mve:A (243)	Truncated 1,3-1,4-beta-D-glucanase	**52**	39	0.0005	0.22	3.32	138	11.5% : 11.6% (12/104 : 16/138)	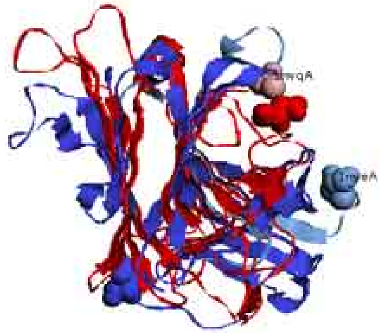
	* 1mvq:A (236)	lectin, isoform 1								
973	* 1o4y:A (270)	beta-agarase A	**80**	47	3E-06	0.34	2.08	184	16.6% : 16.3% (24/145 : 30/184)	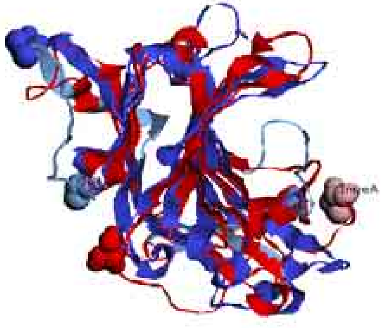
	* 1mve:A (243)	Truncated 1,3-1,4-beta-D-glucanase								
974	* 1mve:A (243)	Truncated 1,3-1,4-beta-D-glucanase	**154**	43	2E-05	0.28	1.92	166	21.5% : 20.5% (28/130 : 34/166)	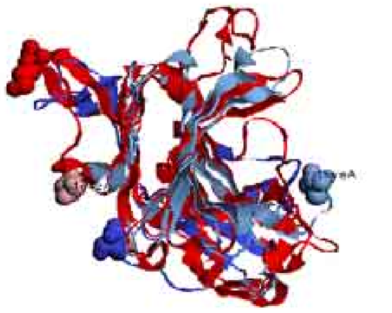
	* 1o4z:A (295)	beta-agarase B								

975	* 1vav:A (222)	Alginate lyase PA1167	**62**	39	0.0005	0.21	3.04	125	5.4% : 12.8% (2/37 : 16/125)	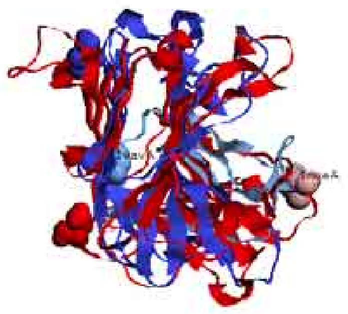
	* 1mve:A (243)	Truncated 1,3-1,4-beta-D-glucanase								
976	* 1mve:A (243)	Truncated 1,3-1,4-beta-D-glucanase	**181**	36	0.005	0.22	2.41	156	15.2% : 14.7% (19/125 : 23/156)	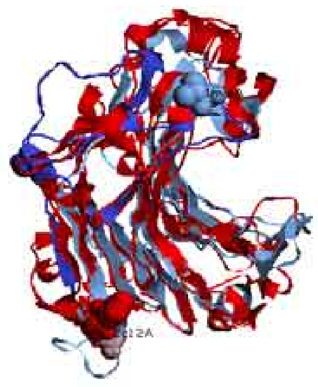
	* 2cl2:A (298)	PUTATIVE LAMINARINASE								
977	* 2cws:A (227)	alginate lyase A1-II'	**65**	41	0.0001	0.28	3.02	146	8.7% : 11.0% (11/126 : 16/146)	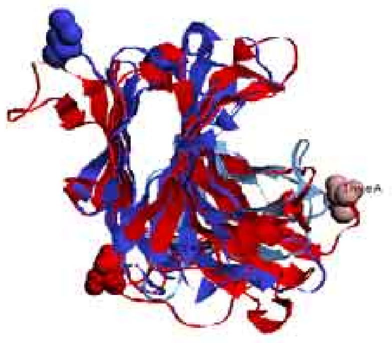
	* 1mve:A (243)	Truncated 1,3-1,4-beta-D-glucanase								

978	1mvo:A (121)	PhoP response regulator	**56**	35	0.004	0.28	3.30	71	11.7% : 22.5% (7/60 : 16/71)	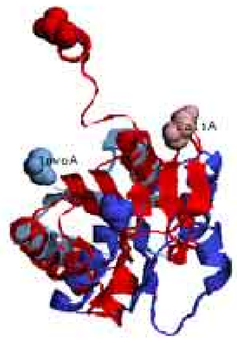
	2a1i:A (129)	DNA excision repair protein ERCC-1								
979	1n3o:A (241)	lectin PAL	**121**	172	2E-44	0.90	1.38	224	44.3% : 47.3% (51/115 : 106/224)	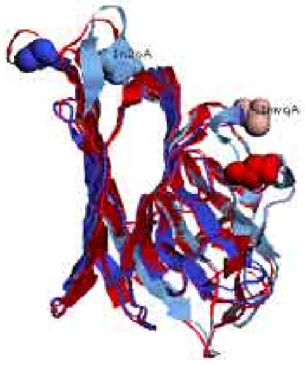
	1mvq:A (236)	lectin, isoform 1								
980	1mvq:A (236)	lectin, isoform 1	**123**	162	3E-41	0.90	1.13	222	48.6% : 46.8% (54/111 : 104/222)	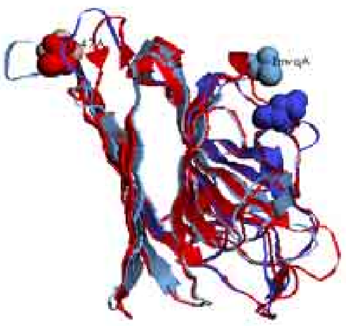
	1n47:A (233)	Isolectin B4								

981	1mvq:A (236)	lectin, isoform 1	**130**	35	0.007	0.33	3.22	132	21.7% : 8.3% (13/60 : 11/132)	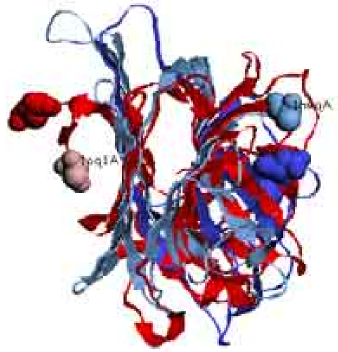
	1oq1:A (223)	Protein yesU								
982	1sla:A (134)	BOVINE GALECTIN-1	**36**	39	0.0002	0.33	2.35	96	16.4% : 11.5% (11/67 : 11/96)	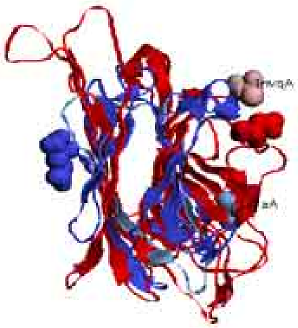
	1mvq:A (236)	lectin, isoform 1								
983	1mvq:A (236)	lectin, isoform 1	**114**	38	0.001	0.26	3.26	116	6.3% : 8.6% (4/64 : 10/116)	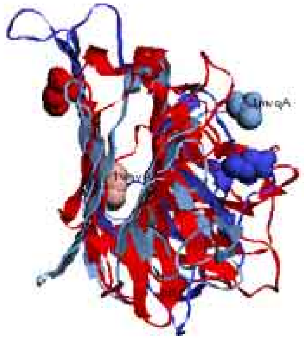
	1vav:A (222)	Alginate lyase PA1167								

984	1wbf:A (237)<br>1mvq:A (236)	PROTEIN (AGGLUTININ)<br>lectin, isoform 1	**115**	136	2E-33	0.85	1.41	218	42.3% : 42.2% (47/111 : 92/218)	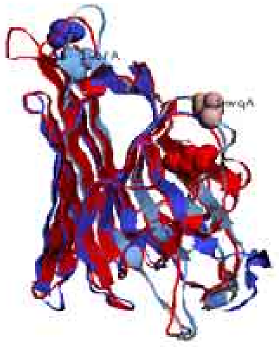
985	2a5z:A (239)<br>1mvq:A (236)	hypothetical protein SO2946<br>lectin, isoform 1	**115**	37	0.002	0.32	3.26	145	9.8% : 10.3% (9/92 : 15/145)	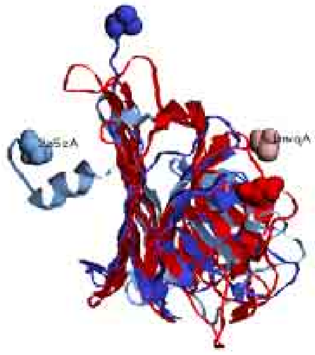
986	1mvq:A (236)<br>2a6v:A (218)	lectin, isoform 1<br>Emp46p	**117**	55	8E-09	0.53	2.55	165	17.6% : 16.4% (15/85 : 27/165)	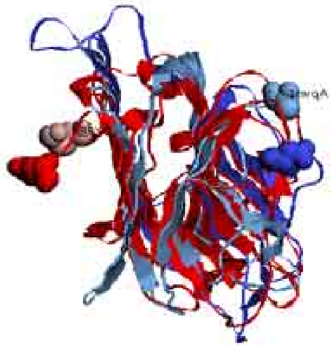

987	1mvq:A (236)	lectin, isoform 1	**117**	40	0.0003	0.45	2.19	159	18.1% : 18.2% (15/83 : 29/159)	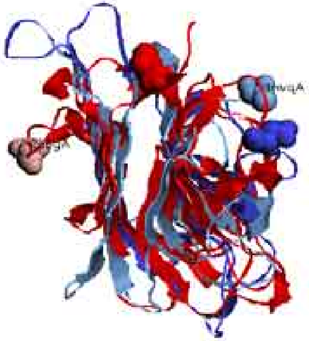
	2a6y:A (232)	Emp47p (form1)								
988	1mvq:A (236)	lectin, isoform 1	**123**	118	4E-28	0.76	1.41	169	39.2% : 40.2% (40/102 : 68/169)	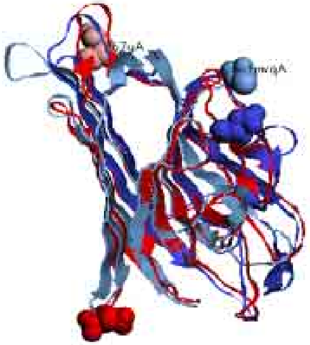
	2b7y:A (181)	Favin beta chain								
989	2cws:A (227)	alginate lyase A1-II'	**112**	35	0.008	0.32	3.18	135	11.0% : 11.9% (9/82 : 16/135)	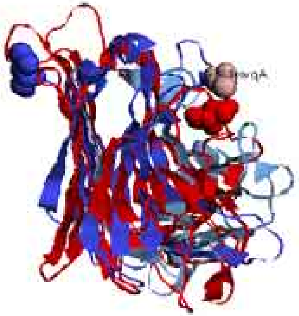
	1mvq:A (236)	lectin, isoform 1								

990	1mvq:A (236)	lectin, isoform 1	**123**	212	4E-56	0.95	0.80	227	65.2% : 60.4% (75/115 : 137/227)	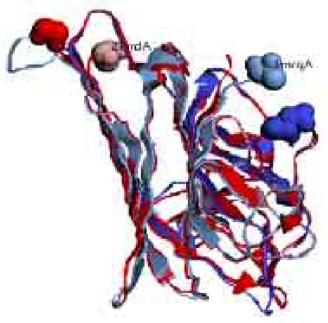
	2fmd:A (233)	Lectin								
991	1sgj:A (231)	citrate lyase, beta subunit	**180**	38	0.001	0.36	3.03	154	8.0% : 11.7% (12/150 : 18/154)	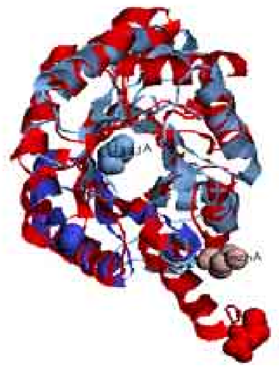
	1mzh:A (225)	Deoxyribose-phosphate aldolase								
992	2d5k:A (148)	Dps family protein	**75**	37	0.001	0.24	2.84	78	6.8% : 5.1% (3/44 : 4/78)	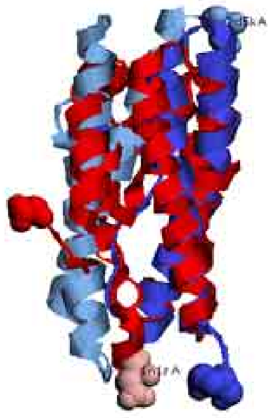
	1n1f:A (154)	interleukin-19								

993	2ib0:A (142)	CONSERVED HYPOTHETICAL ALANINE RICH PROTEIN	**34**	34	0.009	0.33	3.38	101	8.7% : 5.9% (4/46 : 6/101)	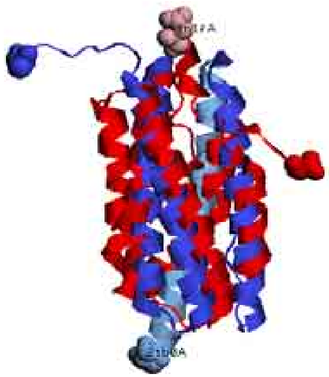
	1n1f:A (154)	interleukin-19								
994	1n1q:A (149)	DPS Protein	**82**	38	0.0006	0.23	2.42	73	7.8% : 16.4% (5/64 : 12/73)	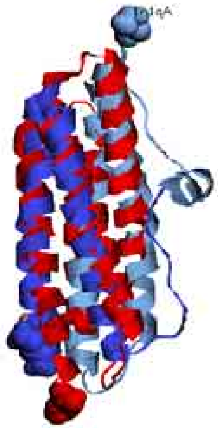
	1orj:A (126)	flagellar protein FliS								
995	1sgj:A (231)	citrate lyase, beta subunit	**51**	33	0.023	0.25	3.22	155	10.1% : 9.7% (13/129 : 15/155)	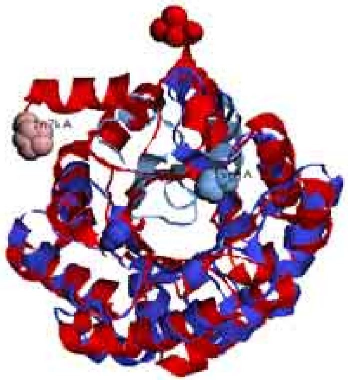
	1n7k:A (234)	deoxyribose-phosphate aldolase								

996	1n7k:A (234)	deoxyribose-phosphate aldolase	**130**	38	0.0006	0.28	3.06	149	16.1% : 10.7% (23/143 : 16/149)	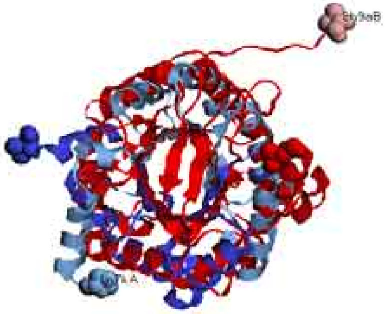
	2h9a:B (307)	CO dehydrogenase/acetyl-CoA synthase, iron-su								
997	1nc5:A (363)	hypothetical protein yTER	**195**	45	1E-05	0.49	3.39	211	9.7% : 9.5% (15/154 : 20/211)	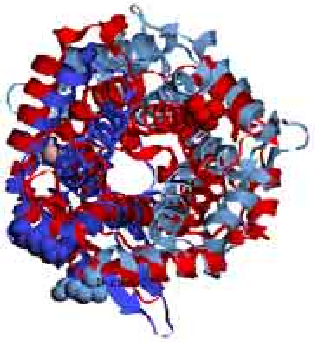
	1qqf:A (277)	PROTEIN (COMPLEMENT C3DG)								
998	1nf4:A (169)	bacterioferritin	**39**	47	1E-06	0.25	2.23	89	4.5% : 9.0% (4/88 : 8/89)	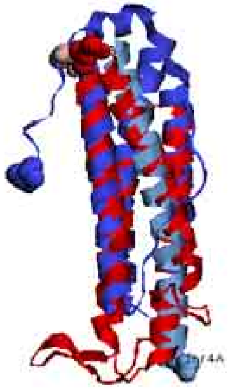
	1yjg:A (157)	Surface protein VspA								

999	1z23:A (163)	CRK-associated substrate	**123**	38	0.0006	0.22	3.04	84	10.7% : 6.0% (6/56 : 5/84)	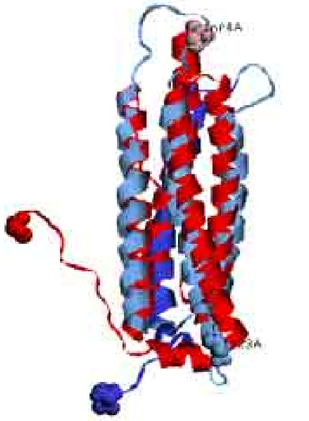
	1nf4:A (169)	bacterioferritin								
1000	1spv:A (172)	putative polyprotein/phosphatase	**149**	55	5E-09	0.21	1.94	136	19.3% : 17.6% (22/114 : 24/136)	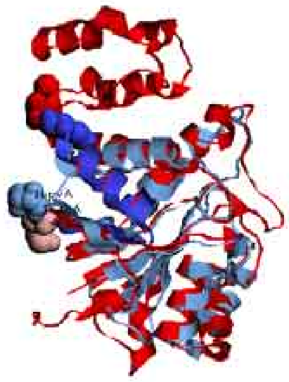
	1njr:A (252)	32.1 kDa protein in ADH3-RCA1 intergenic regi								
1001	1no4:A (77)	HEAD MORPHOGENESIS PROTEIN	**22**	41	6E-05	0.30	2.70	54	9.8% : 13.0% (5/51 : 7/54)	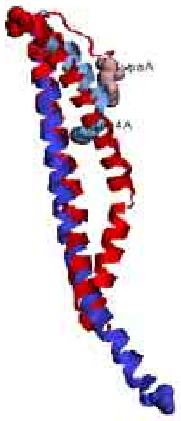
	1wpa:A (107)	Occludin								

1002	1nog:A (149)	conserved hypothetical protein TA0546	**59**	35	0.003	0.20	2.90	77	10.2% : 11.7% (5/49 : 9/77)	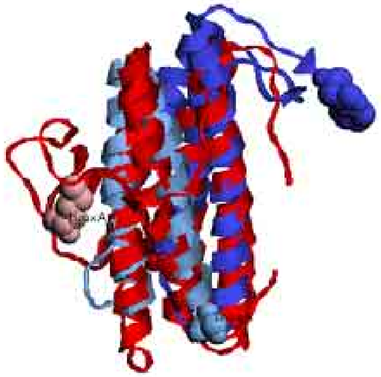
	1yux:A (201)	Nigerythrin								
1003	2fzf:A (158)	hypothetical protein	**92**	32	0.049	0.40	2.56	87	11.4% : 5.7% (8/70 : 5/87)	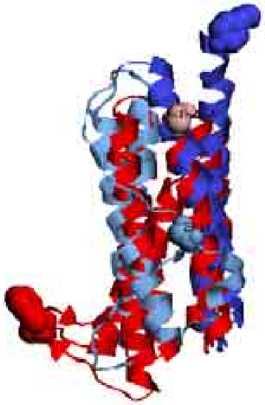
	1nog:A (149)	conserved hypothetical protein TA0546								
1004	1npe:A (263)	nidogen	**107**	41	0.0002	0.34	3.49	164	13.7% : 17.7% (21/153 : 29/164)	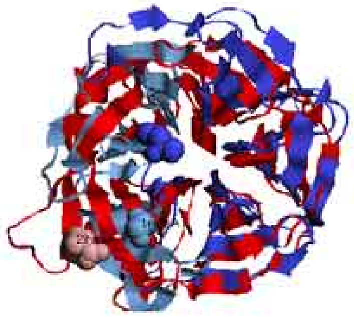
	2faw:A (250)	glutamine cyclotransferase								

1005	1nps:A (87)	DEVELOPMENT-SPECIFIC PROTEIN S	**47**	32	0.046	0.86	1.25	79	24.0% : 35.4% (18/75 : 28/79)	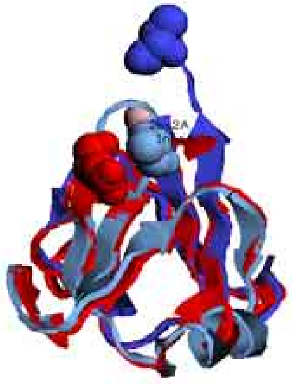
	2bv2:A (83)	CIONA BETAGAMMA-CRYSTALLIN								
1006	2dad:A (93)	Absent in melanoma 1 protein	**45**	41	8E-05	0.69	1.50	78	25.0% : 20.5% (18/72 : 16/78)	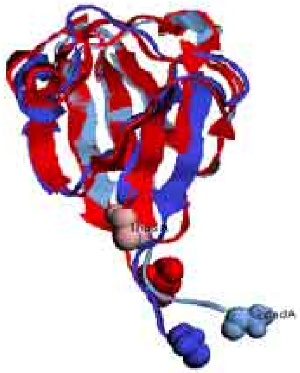
	1nps:A (87)	DEVELOPMENT-SPECIFIC PROTEIN S								
1007	* 2ged:A (172)	Signal recognition particle receptor beta sub	**37**	263	9E-72	0.45	1.62	142	100.0% : 100.0% (109/109 : 142/142)	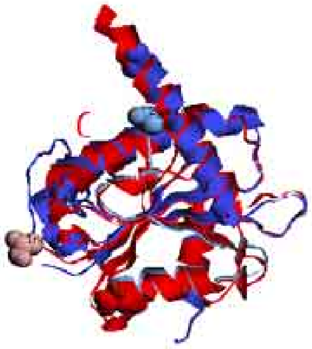
	* 1nrj:B (193)	Signal recognition particle receptor beta sub								

1008	1viz:A (226)<br>1nvm:A (340)	PcrB protein homolog<br>4-hydroxy-2-oxovalerate aldolase	**108**	39	0.0004	0.31	2.78	167	9.2% : 12.0% (14/153 : 20/167)	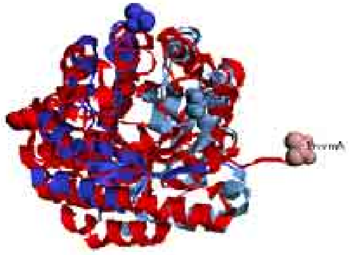
1009	1yx1:A (250)<br>1nvm:A (340)	hypothetical protein PA2260<br>4-hydroxy-2-oxovalerate aldolase	**110**	42	7E-05	0.25	3.33	160	12.0% : 13.1% (17/142 : 21/160)	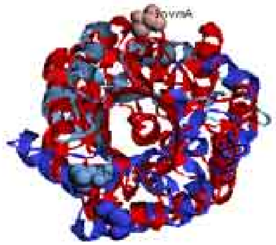
1010	2f6u:A (231)<br>1nvm:A (340)	(S)-3-O-Geranylgeranylglyceryl Phosphate Synt<br>4-hydroxy-2-oxovalerate aldolase	**116**	37	0.001	0.27	3.08	154	9.7% : 11.0% (13/134 : 17/154)	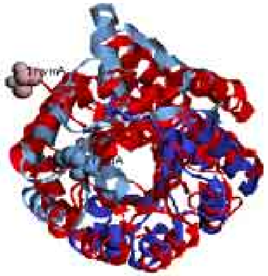

1011	1nxo:A (117)	DNA-binding response regulator	**33**	34	0.01	0.22	3.27	77	5.5% : 11.7% (3/55 : 9/77)	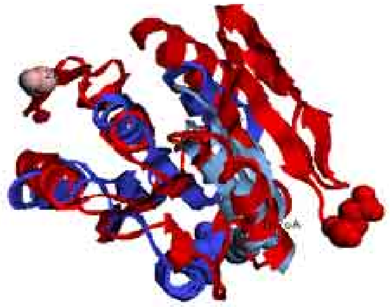
	2fpo:A (178)	methylase yhhF								
1012	* 1wp7:A (65)	fusion protein	**42**	36	0.003	0.22	2.10	46	7.7% : 8.7% (2/26 : 4/46)	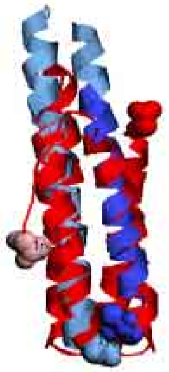
	* 1nze:A (112)	Oxygen-evolving enhancer protein 3								
1013	1wrd:A (98)	Target of Myb protein 1	**73**	36	0.001	0.34	2.48	67	3.7% : 10.4% (2/54 : 7/67)	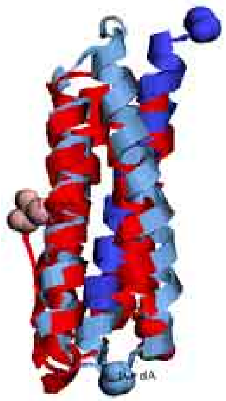
	1nze:A (112)	Oxygen-evolving enhancer protein 3								

1014	1nze:A (112)	Oxygen-evolving enhancer protein 3	**35**	34	0.007	0.33	3.04	82	14.5% : 12.2% (9/62 : 10/82)	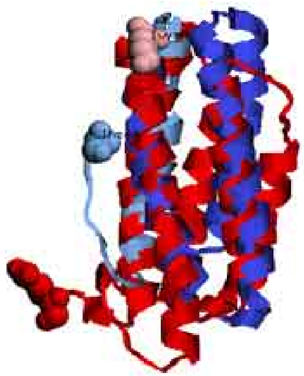
	1wvt:A (148)	hypothetical protein ST2180								
1015	1wy1:A (142)	hypothetical protein PH0671	**85**	36	0.002	0.34	3.03	85	2.1% : 5.9% (1/47 : 5/85)	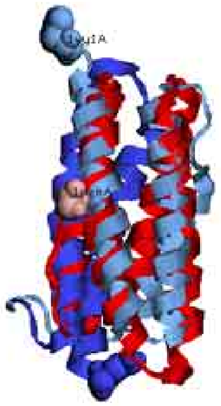
	1nze:A (112)	Oxygen-evolving enhancer protein 3								
1016	1zke:A (81)	Hypothetical protein HP1531	**19**	32	0.031	0.23	2.51	55	6.1% : 18.2% (3/49 : 10/55)	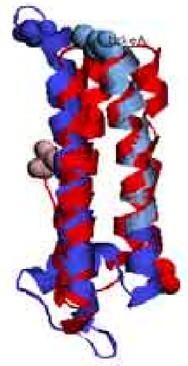
	1nze:A (112)	Oxygen-evolving enhancer protein 3								

1017	2gsc:A (114)	conserved hypothetical protein	**23**	41	8E-05	0.33	2.42	90	12.2% : 12.2% (6/49 : 11/90)	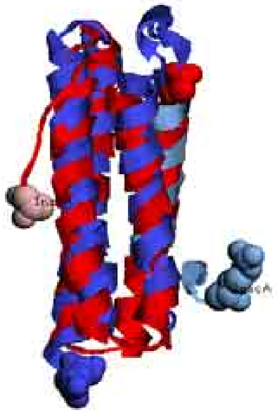
	1nze:A (112)	Oxygen-evolving enhancer protein 3								
1018	1o1z:A (226)	glycerophosphodiester phosphodiesterase	**93**	32	0.077	0.26	2.83	146	20.0% : 12.3% (28/140 : 18/146)	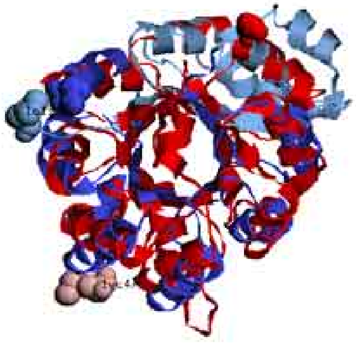
	1vc4:A (254)	Indole-3-Glycerol Phosphate Synthase								
1019	1o3u:A (121)	conserved hypothetical protein TM0613	**80**	42	3E-05	0.35	2.89	78	3.8% : 3.8% (2/52 : 3/78)	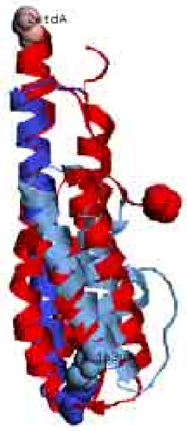
	2etd:A (145)	lemA protein								

1020	1o50:A (142)<br>2j9l:A (168)	CBS domain-containing predicted protein TM093<br>CHLORIDE CHANNEL PROTEIN 5	**73**	43	2E-05	0.66	3.13	120	19.5% : 20.0% (23/118 : 24/120)	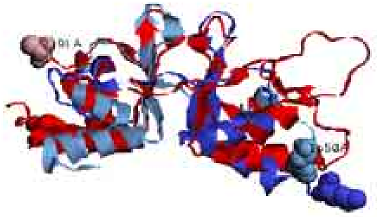
1021	1o51:A (90)<br>2f1f:A (163)	Hypothetical protein TM0021<br>Acetolactate synthase isozyme III small subun	**49**	35	0.004	0.22	2.25	66	8.2% : 12.1% (5/61 : 8/66)	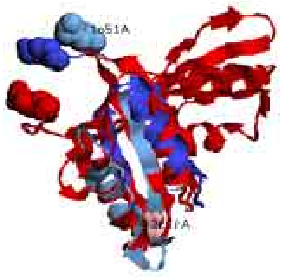
1022	1yx1:A (250)<br>1o5q:A (272)	hypothetical protein PA2260<br>Probable methylisocitrate lyase	**118**	34	0.012	0.28	2.85	142	11.5% : 13.4% (16/139 : 19/142)	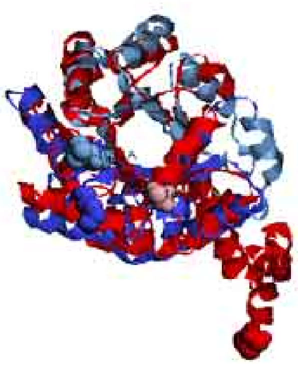

1023	1v77:A (202)	hypothetical protein PH1877	**107**	32	0.067	0.20	3.48	116	17.6% : 11.2% (18/102 : 13/116)	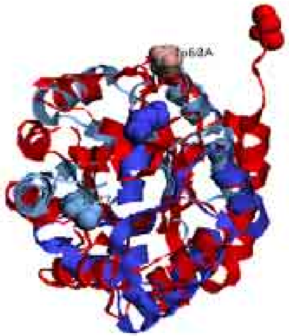
	1o60:A (270)	2-dehydro-3-deoxyphosphooctonate aldolase								
1024	1z69:A (327)	Coenzyme F420-dependent N(5),N(10)-methylenet	**193**	33	0.033	0.25	3.25	165	8.1% : 11.5% (9/111 : 19/165)	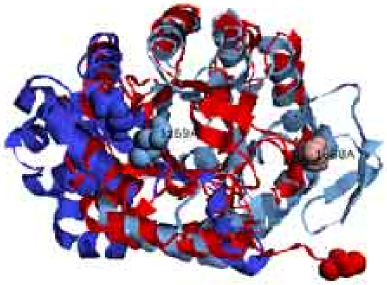
	1o60:A (270)	2-dehydro-3-deoxyphosphooctonate aldolase								
1025	1w0m:A (225)	TRIOSEPHOSPHATE ISOMERASE	**101**	56	3E-09	0.27	3.17	127	13.4% : 18.1% (16/119 : 23/127)	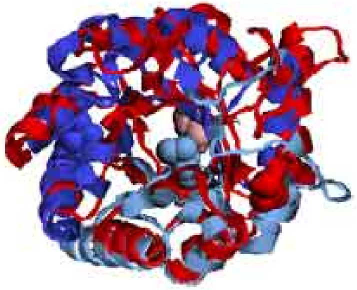
	1o66:A (250)	3-methyl-2-oxobutanoate hydroxymethyltransfer								

1026	2cwr:A (97)	chitinase	**55**	36	0.001	0.28	3.39	57	3.8% : 8.8% (2/52 : 5/57)	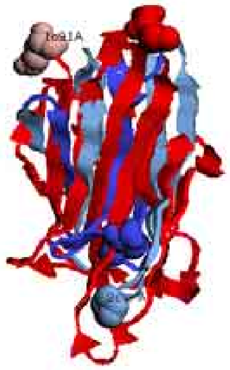
	1o91:A (131)	COLLAGEN ALPHA 1(VIII) CHAIN								
1027	1z7c:A (165)	Chorionic somatomammotropin hormone	**119**	33	0.018	0.30	3.47	97	10.7% : 9.3% (8/75 : 9/97)	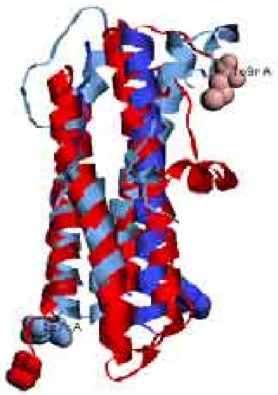
	1o9r:A (162)	AGROBACTERIUM TUMEFACIENS DPS								
1028	1oed:B (128)	ACETYLCHOLINE RECEPTOR PROTEIN, BETA CHAIN	**30**	36	0.002	0.30	3.46	79	1.9% : 5.1% (1/53 : 4/79)	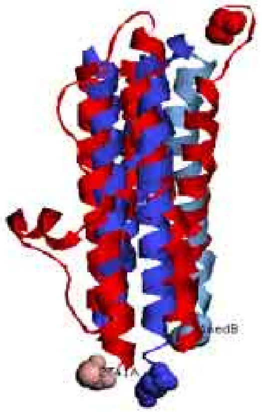
	2c41:A (155)	DPS FAMILY DNA-BINDING STRESS RESPONSE PROTEI								

1029	1y69:8 (113)	Ribosome recycling factor	84	35	0.005	0.34	3.05	76	7.3% : 2.6% (4/55 : 2/76)	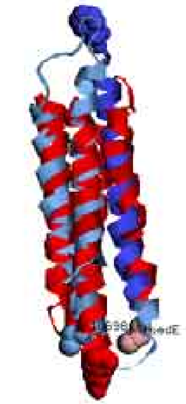
	1oed:E (129)	ACETYLCHOLINE RECEPTOR PROTEIN, GAMMA CHAIN								
1030	1oiv:A (168)	RAS-RELATED PROTEIN RAB-11A	64	51	7E-08	0.22	2.81	121	9.3% : 15.7% (8/86 : 19/121)	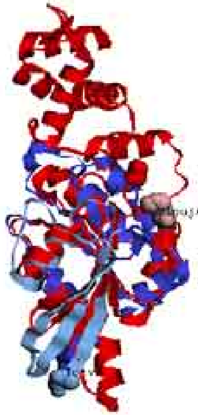
	1puj:A (262)	conserved hypothetical protein ylqF								
1031	1ojh:A (52)	NBLA	13	32	0.044	0.24	2.11	36	4.5% : 11.1% (1/22 : 4/36)	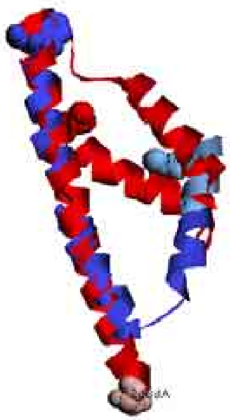
	2d8d:A (80)	phospho-2-dehydro-3-deoxyheptonate aldolase/c								

1032	1olz:A (622)	SEMAPHORIN 4D	**332**	35	0.021	0.22	3.20	234	5.9% : 9.4% (11/185 : 22/234)	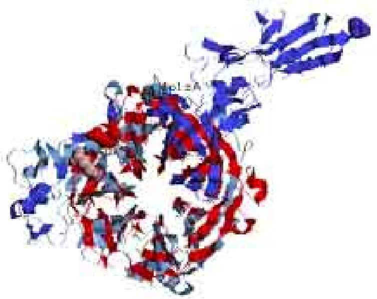
	1s4u:X (361)	Antiviral protein SKI8								
1033	1sfj:A (229)	3-dehydroquinate dehydratase	**77**	33	0.038	0.33	3.42	156	7.0% : 9.0% (10/142 : 14/156)	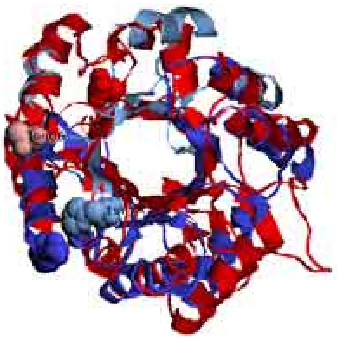
	1om0:A (274)	Xylanase Inhibitor Protein I								
1034	* 1oot:A (58)	Hypothetical 40.4 kDa protein in PES4-HIS2 in	**15**	36	0.003	0.49	2.01	42	34.3% : 35.7% (12/35 : 15/42)	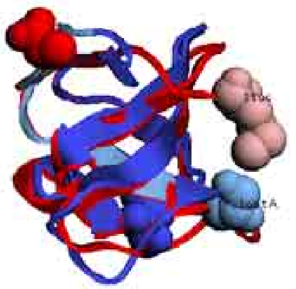
	* 1tuc_ (65)	ALPHA-SPECTRIN								

1035	1vjx:A (148)	putative ferritin-like diiron-carboxylate pro	**79**	44	1E-05	0.25	3.08	73	20.3% : 11.0% (13/64 : 8/73)	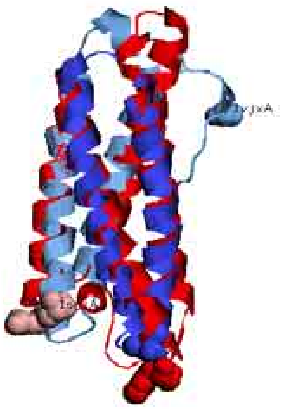
	1orj:A (126)	flagellar protein FliS								
1036	1orj:A (126)	flagellar protein FliS	**66**	39	0.0002	0.22	3.36	75	7.5% : 13.3% (4/53 : 10/75)	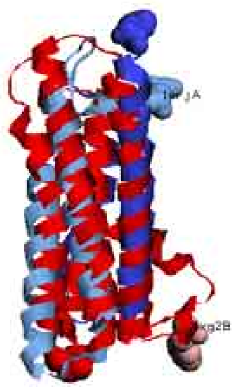
	1xg2:B (151)	Pectinesterase inhibitor								
1037	1yxr:A (77)	vacuolar protein sorting factor 4A	**56**	35	0.003	0.41	2.73	61	10.6% : 11.5% (5/47 : 7/61)	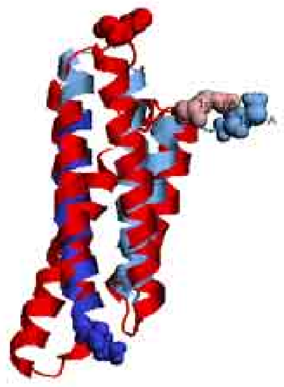
	1orj:A (126)	flagellar protein FliS								

1038	1owa:A (156)	Spectrin alpha chain, erythrocyte	**86**	40	0.0001	0.25	2.72	92	7.4% : 15.2% (6/81 : 14/92)	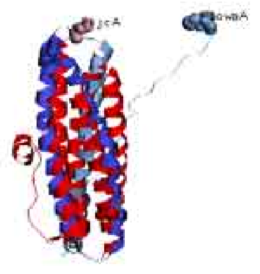
	2fjc:A (151)	Antigen TpF1								
1039	1sgj:A (231)	citrate lyase, beta subunit	**101**	36	0.005	0.31	2.60	142	10.6% : 14.1% (14/132 : 20/142)	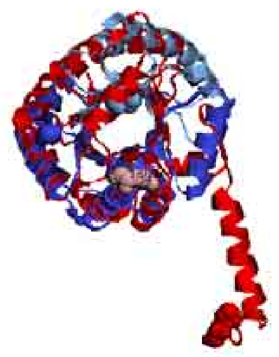
	1oy0:A (249)	Ketopantoate hydroxymethyltransferase								
1040	1p4t:A (155)	outer membrane protein NspA	**107**	36	0.002	0.24	2.83	88	7.7% : 8.0% (6/78 : 7/88)	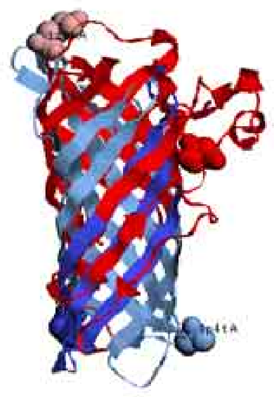
	1z8k:A (174)	At3g25770 protein								

1041	* 1s05:A (129)	Cytochrome c-556	**77**	31	0.075	0.34	3.48	64	6.5% : 7.8% (3/46 : 5/64)	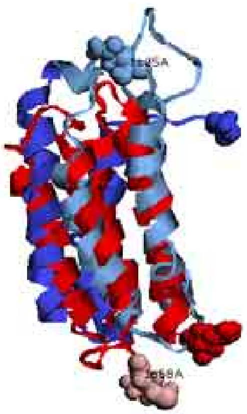
	* 1p68:A (102)	De novo designed protein S-824								
1042	* 1zzp:A (109)	Proto-oncogene tyrosine-protein kinase ABL1	**87**	35	0.004	0.40	2.82	84	6.3% : 11.9% (3/48 : 10/84)	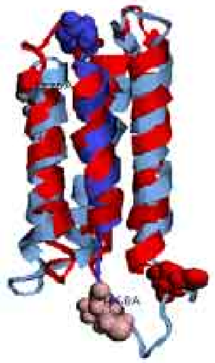
	* 1p68:A (102)	De novo designed protein S-824								
1043	2j9l:A (168)	CHLORIDE CHANNEL PROTEIN 5	**101**	50	1E-07	0.70	3.25	104	18.6% : 18.3% (19/102 : 19/104)	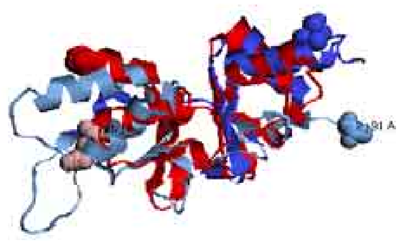
	1pbj:A (120)	hypothetical protein								

1044	1pu1:A (91)	Hypothetical protein MTH677	**71**	32	0.037	0.27	2.57	64	5.7% : 6.3% (3/53 : 4/64)	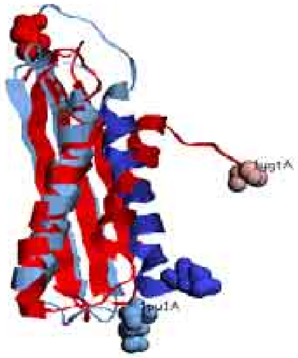
	1ygt:A (104)	Cytoplasmic dynein light chain								
1045	1pui:A (171)	Probable GTP-binding protein engB	**67**	52	3E-08	0.27	3.00	130	20.0% : 20.8% (18/90 : 27/130)	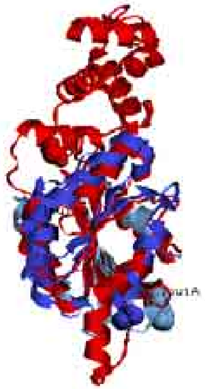
	1puj:A (262)	conserved hypothetical protein ylqF								
1046	1r4a:A (165)	ADP-ribosylation factor-like protein 1	**55**	37	0.001	0.23	2.84	121	17.9% : 22.3% (15/84 : 27/121)	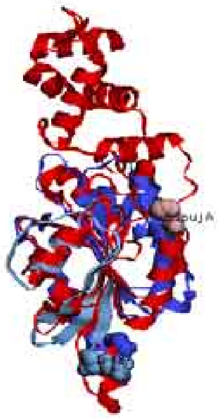
	1puj:A (262)	conserved hypothetical protein ylqF								

1047	1s8f:A (169)	Ras-related protein Rab-9A	**70**	43	2E-05	0.23	2.67	120	11.1% : 17.5% (10/90 : 21/120)	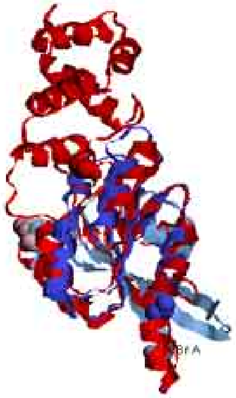
	1puj:A (262)	conserved hypothetical protein ylqF								
1048	1sul:A (187)	GTP-binding protein YsxC	**85**	37	0.001	0.23	2.89	126	18.0% : 20.6% (16/89 : 26/126)	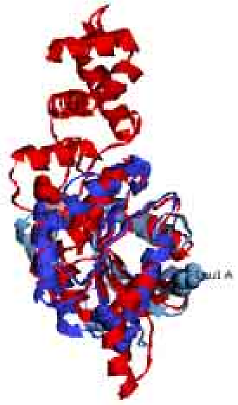
	1puj:A (262)	conserved hypothetical protein ylqF								
1049	1t91:A (176)	Ras-related protein Rab-7	**60**	57	2E-09	0.23	3.12	127	14.6% : 18.1% (13/89 : 23/127)	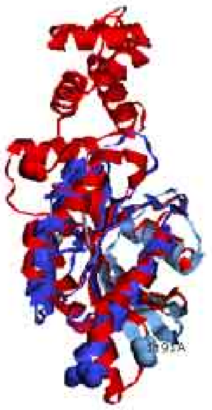
	1puj:A (262)	conserved hypothetical protein ylqF								

1050	1x1r:A (169)	Ras-related protein M-Ras	**68**	41	6E-05	0.24	2.99	123	9.4% : 15.4% (8/85 : 19/123)	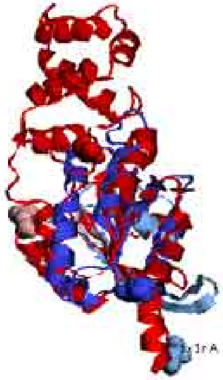
	1puj:A (262)	conserved hypothetical protein ylqF								
1051	1xtq:A (169)	GTP-binding protein Rheb	**68**	46	3E-06	0.25	2.91	125	13.1% : 18.4% (11/84 : 23/125)	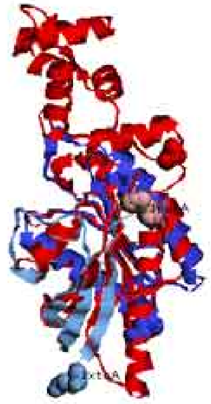
	1puj:A (262)	conserved hypothetical protein ylqF								
1052	1yu9:A (175)	GTP-binding protein	**75**	53	2E-08	0.22	2.70	123	10.0% : 15.4% (9/90 : 19/123)	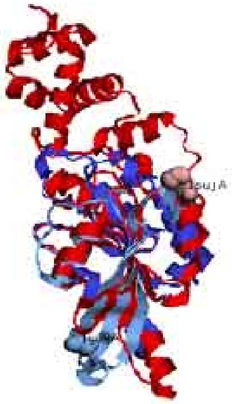
	1puj:A (262)	conserved hypothetical protein ylqF								

1053	1yvd:A (166)	Ras-related protein Rab-22A	**62**	54	1E-08	0.24	2.12	123	8.9% : 14.6% (8/90 : 18/123)	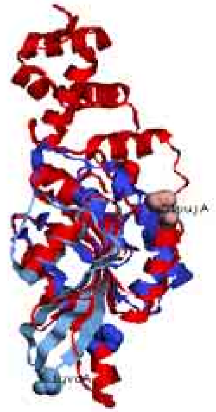
	1puj:A (262)	conserved hypothetical protein ylqF								
1054	1yzg:A (168)	ADP-ribosylation factor-like 8	**64**	45	4E-06	0.23	3.09	119	15.4% : 16.8% (12/78 : 20/119)	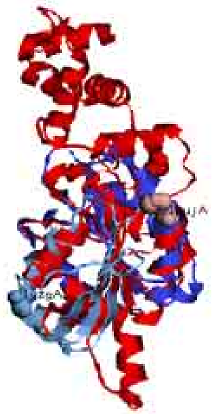
	1puj:A (262)	conserved hypothetical protein ylqF								
1055	1yzq:A (164)	small GTP binding protein RAB6 isoform	**64**	42	4E-05	0.23	2.84	120	11.1% : 17.5% (10/90 : 21/120)	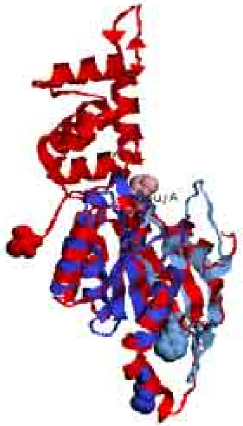
	1puj:A (262)	conserved hypothetical protein ylqF								

1056	1z06:A (165)	Ras-related protein Rab-33B	**61**	42	5E-05	0.26	3.43	123	13.8% : 21.1% (12/87 : 26/123)	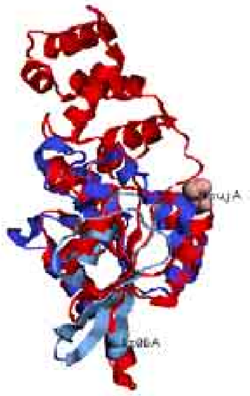
	1puj:A (262)	conserved hypothetical protein ylqF								
1057	1z0a:A (167)	Ras-related protein Rab-2A	**61**	49	3E-07	0.23	2.67	121	8.2% : 15.7% (7/85 : 19/121)	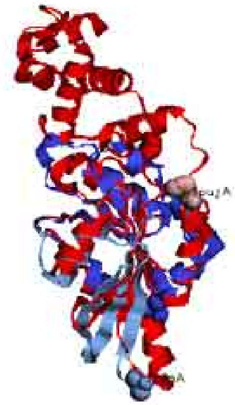
	1puj:A (262)	conserved hypothetical protein ylqF								
1058	1z0f:A (167)	RAB14, member RAS oncogene family	**63**	51	9E-08	0.23	3.04	121	15.1% : 19.8% (13/86 : 24/121)	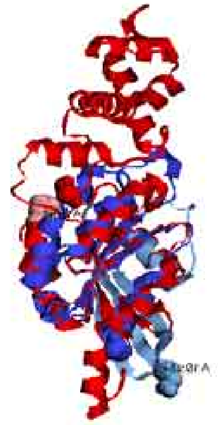
	1puj:A (262)	conserved hypothetical protein ylqF								

1059	1z22:A (164)	Ras-related protein Rab-23	65	45	4E-06	0.26	2.86	127	9.2% : 17.3% (8/87 : 22/127)	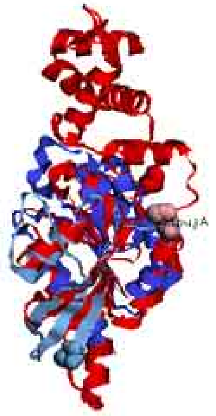
	1puj:A (262)	conserved hypothetical protein ylqF								
1060	1z6x:A (175)	ADP-ribosylation factor 4	67	41	0.0001	0.24	3.37	126	11.8% : 15.1% (10/85 : 19/126)	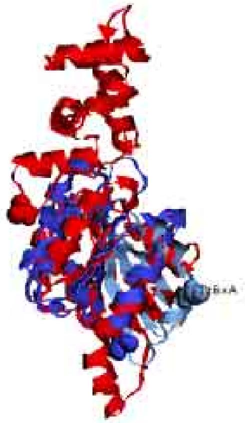
	1puj:A (262)	conserved hypothetical protein ylqF								
1061	1z6y:A (170)	ADP-ribosylation factor-like protein 5	71	46	3E-06	0.23	3.07	118	12.7% : 16.9% (10/79 : 20/118)	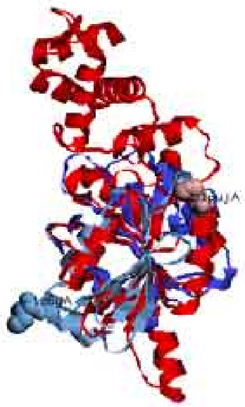
	1puj:A (262)	conserved hypothetical protein ylqF								

1062	1zbd:A (177)<br>1puj:A (262)	RABPHILIN-3A<br>conserved hypothetical protein ylqF	**65**	58	8E-10	0.24	3.17	130	14.1% : 20.8% (13/92 : 27/130)	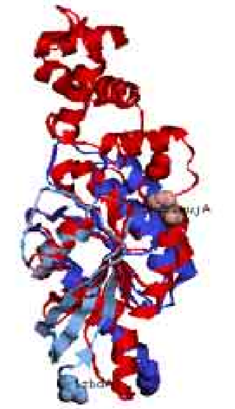
1063	1zd9:A (166)<br>1puj:A (262)	ADP-ribosylation factor-like 10B<br>conserved hypothetical protein ylqF	**58**	42	5E-05	0.23	3.10	121	18.2% : 20.7% (16/88 : 25/121)	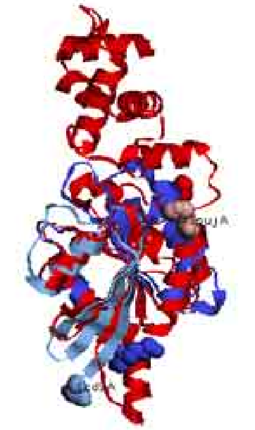
1064	1zj6:A (174)<br>1puj:A (262)	ADP-ribosylation factor-like protein 5<br>conserved hypothetical protein ylqF	**65**	45	4E-06	0.21	3.16	123	19.0% : 18.7% (16/84 : 23/123)	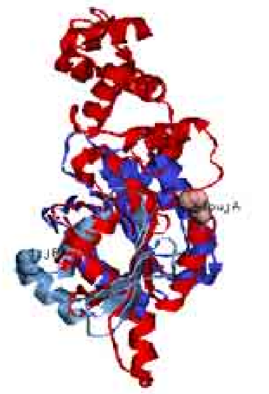

1065	2atv:A (168)	RAS-like estrogen-regulated growth inhibitor	**65**	49	4E-07	0.24	2.78	123	13.8% : 20.3% (12/87 : 25/123)	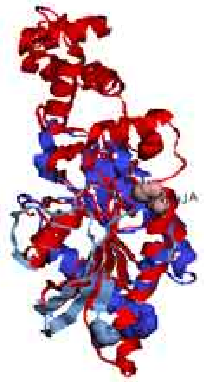
	1puj:A (262)	conserved hypothetical protein ylqF								
1066	2b6h:A (172)	ADP-ribosylation factor 5	**62**	50	2E-07	0.23	3.11	123	13.3% : 17.1% (11/83 : 21/123)	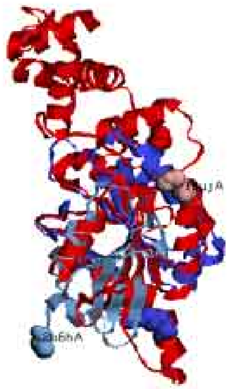
	1puj:A (262)	conserved hypothetical protein ylqF								
1067	2cls:A (179)	RHO-RELATED GTP-BINDING PROTEIN RHO6	**61**	37	0.001	0.21	2.66	119	19.0% : 13.4% (11/58 : 16/119)	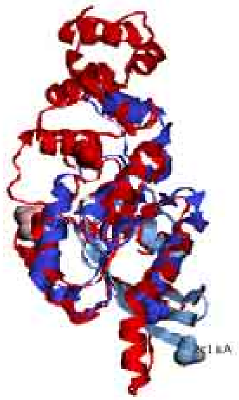
	1puj:A (262)	conserved hypothetical protein ylqF								

1068	2dpx:A (149)	GTP-binding protein RAD	**44**	49	2E-07	0.22	2.83	115	10.7% : 14.8% (9/84 : 17/115)	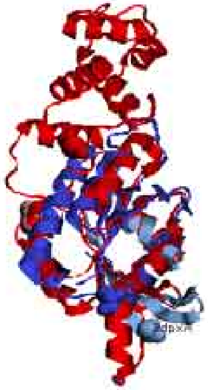
	1puj:A (262)	conserved hypothetical protein ylqF								
1069	2erx:A (169)	GTP-binding protein Di-Ras2	**59**	48	8E-07	0.23	2.87	123	7.0% : 13.8% (6/86 : 17/123)	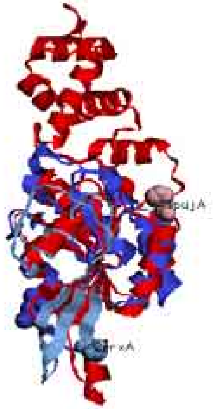
	1puj:A (262)	conserved hypothetical protein ylqF								
1070	2ery:A (168)	Ras-related protein R-Ras2	**59**	48	8E-07	0.21	2.68	121	11.5% : 15.7% (10/87 : 19/121)	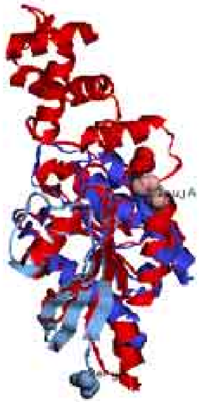
	1puj:A (262)	conserved hypothetical protein ylqF								

1071	2ew1:A (171)	Ras-related protein Rab-30	**63**	54	9E-09	0.24	2.74	128	10.9% : 15.6% (10/92 : 20/128)	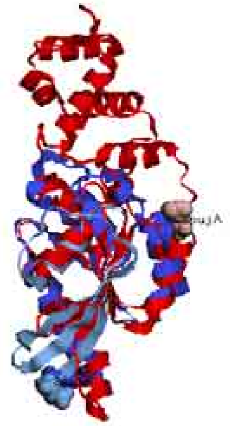
	1puj:A (262)	conserved hypothetical protein ylqF								
1072	2f9l:A (174)	RAB11B, member RAS oncogene family	**61**	49	3E-07	0.23	2.93	126	7.9% : 14.3% (7/89 : 18/126)	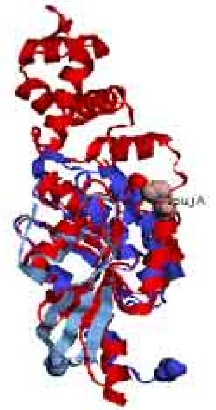
	1puj:A (262)	conserved hypothetical protein ylqF								
1073	2fe4:A (163)	Ras-related protein Rab-6B	**58**	49	4E-07	0.23	2.64	119	12.5% : 19.3% (11/88 : 23/119)	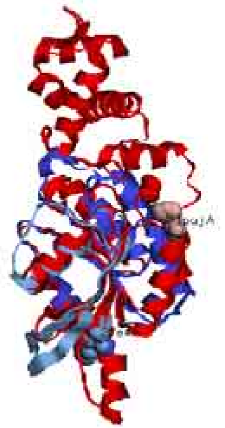
	1puj:A (262)	conserved hypothetical protein ylqF								

1074	2fg5:A (165)	Ras-related protein Rab-31	**61**	49	3E-07	0.24	2.89	124	13.6% : 15.3% (12/88 : 19/124)	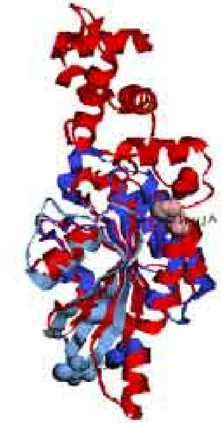
	1puj:A (262)	conserved hypothetical protein ylqF								
1075	2fn4:A (173)	Ras-related protein R-Ras	**70**	51	1E-07	0.26	3.11	129	6.8% : 13.2% (6/88 : 17/129)	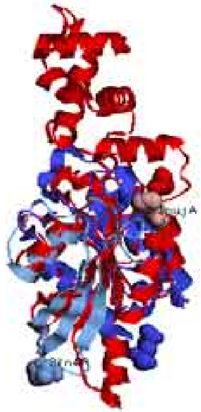
	1puj:A (262)	conserved hypothetical protein ylqF								
1076	2fv8:A (184)	Rho-related GTP-binding protein RhoB	**61**	38	0.0008	0.22	2.88	123	12.9% : 14.6% (11/85 : 18/123)	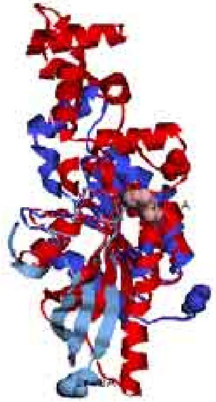
	1puj:A (262)	conserved hypothetical protein ylqF								

1077	2gf0:A (173)	GTP-binding protein Di-Ras1	**61**	46	2E-06	0.22	2.79	124	9.1% : 15.3% (8/88 : 19/124)	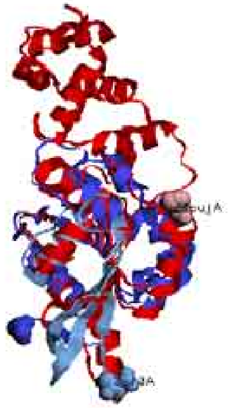
	1puj:A (262)	conserved hypothetical protein ylqF								
1078	2gf9:A (176)	Ras-related protein Rab-3D	**67**	54	1E-08	0.22	2.89	126	15.2% : 19.0% (14/92 : 24/126)	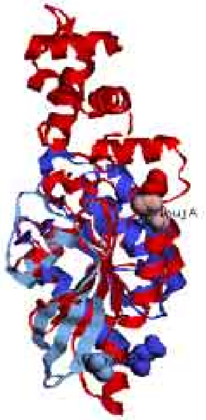
	1puj:A (262)	conserved hypothetical protein ylqF								
1079	2h57:A (165)	ADP-ribosylation factor-like protein 6	**56**	32	0.03	0.24	2.82	119	14.6% : 19.3% (12/82 : 23/119)	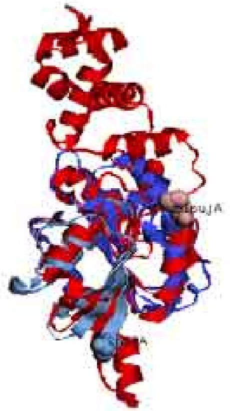
	1puj:A (262)	conserved hypothetical protein ylqF								

1080	2hei:A (164)	Ras-related protein Rab-5B	59	49	4E-07	0.24	2.80	123	11.1% : 16.3% (10/90 : 20/123)	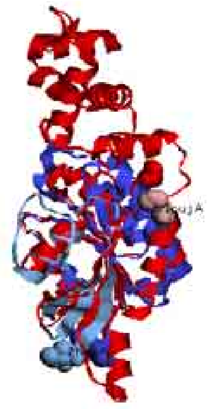
	1puj:A (262)	conserved hypothetical protein ylqF								
1081	2hup:A (163)	RAS-related protein RAB-43	53	53	1E-08	0.27	3.11	129	12.0% : 17.1% (11/92 : 22/129)	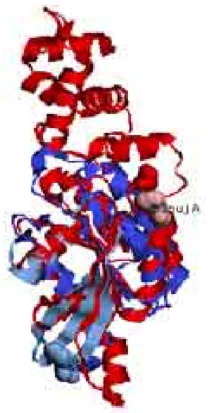
	1puj:A (262)	conserved hypothetical protein ylqF								
1082	2il1:A (164)	Rab12	56	49	4E-07	0.26	2.83	128	8.7% : 16.4% (8/92 : 21/128)	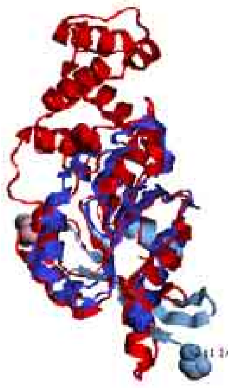
	1puj:A (262)	conserved hypothetical protein ylqF								

1083	2nzj:A (160)<br>1puj:A (262)	GTP-binding protein REM 1<br>conserved hypothetical protein ylqF	**54**	57	1E-09	0.25	2.94	123	12.4% : 18.7% (11/89 : 23/123)	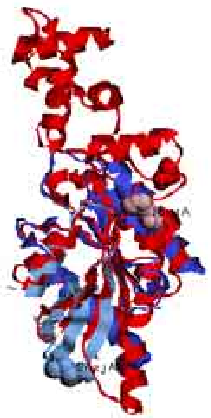
1084	2o52:A (168)<br>1puj:A (262)	Ras-related protein Rab-4B<br>conserved hypothetical protein ylqF	**57**	52	3E-08	0.25	2.92	127	11.4% : 16.5% (10/88 : 21/127)	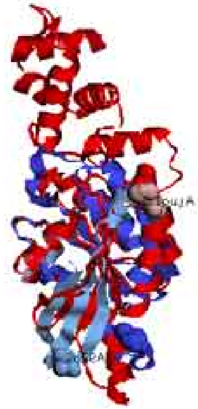
1085	2ocb:A (173)<br>1puj:A (262)	Ras-related protein Rab-9B<br>conserved hypothetical protein ylqF	**73**	51	7E-08	0.23	2.78	121	11.6% : 18.2% (10/86 : 22/121)	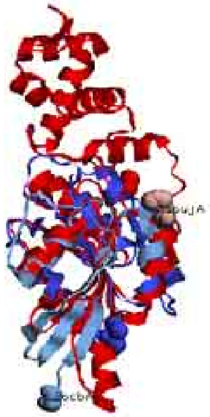

1086	* 1puo:A (142)	Major allergen I polypeptide, fused chain 2,	**70**	83	2E-17	0.88	0.73	135	10.4% : 98.5% (13/125 : 133/135)	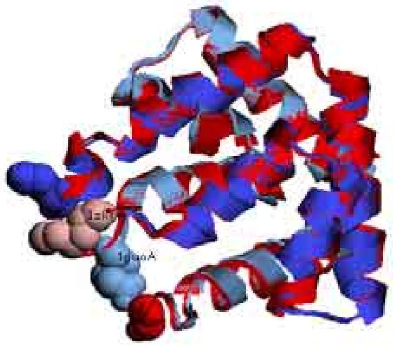
	* 1zkr:A (146)	Major allergen I polypeptide, fused chain 1,								
1087	* 2d80:A (318)	PHB depolymerase	**175**	35	0.008	0.32	2.75	173	18.4% : 17.3% (19/103 : 30/173)	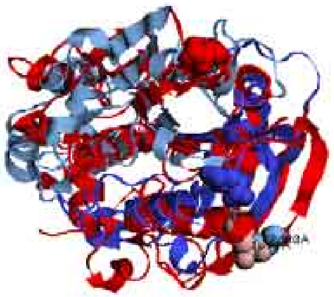
	* 1pv1:A (292)	Hypothetical 33.9 kDa esterase in SMC3-MRPL8								
1088	1q0d:A (117)	Superoxide dismutase [Ni]	**31**	36	0.001	0.25	2.74	77	2.0% : 13.0% (1/49 : 10/77)	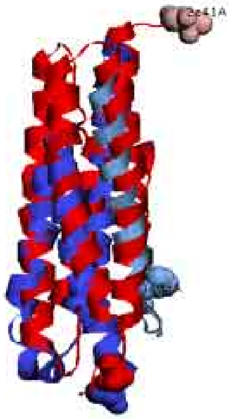
	2c41:A (155)	DPS FAMILY DNA-BINDING STRESS RESPONSE PROTEI								

1089	1q0d:A (117)<br>2d4u:A (167)	Superoxide dismutase [Ni]<br>Methyl-accepting chemotaxis protein I	**61**	45	5E-06	0.36	2.65	87	3.6% : 8.0% (3/83 : 7/87)	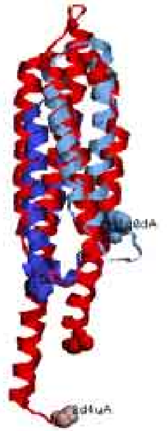
1090	1q0d:A (117)<br>2etd:A (145)	Superoxide dismutase [Ni]<br>lemA protein	**28**	45	4E-06	0.25	2.90	79	2.4% : 5.1% (1/41 : 4/79)	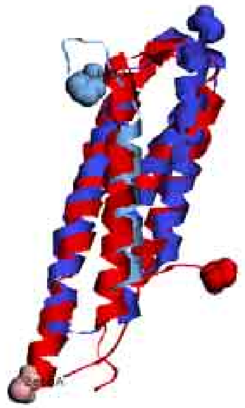
1091	2fcw:A (106)<br>1q0d:A (117)	Alpha-2-macroglobulin receptor-associated pro<br>Superoxide dismutase [Ni]	**33**	39	0.0002	0.23	2.29	61	9.3% : 8.2% (5/54 : 5/61)	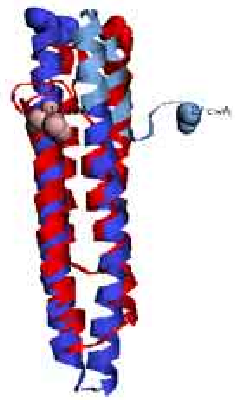

1092	1q0d:A (117)	Superoxide dismutase [Ni]	**92**	36	0.002	0.32	1.60	82	8.2% : 14.6% (4/49 : 12/82)	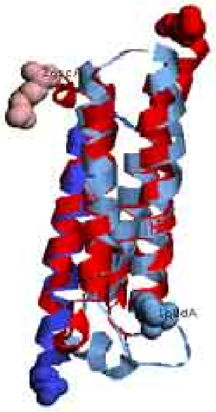
	2gsc:A (114)	conserved hypothetical protein								
1093	1q8b:A (93)	Protein yjcS	**52**	30	0.089	0.55	2.71	64	1.9% : 6.3% (1/54 : 4/64)	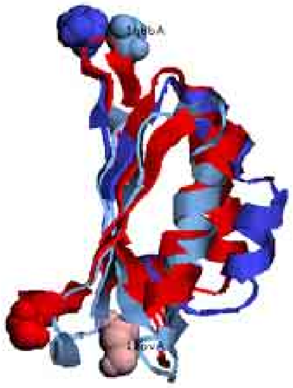
	1zpv:A (86)	ACT domain protein								
1094	1wrd:A (98)	Target of Myb protein 1	**38**	41	8E-05	0.28	2.68	57	6.7% : 10.5% (2/30 : 6/57)	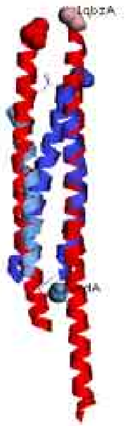
	1qbz:A (103)	PROTEIN (SIV GP41 ECTODOMAIN)								

1095	1xou:A (73)	EspA	**36**	47	1E-06	0.39	2.64	57	0.0% : 5.3% (0/37 : 3/57)	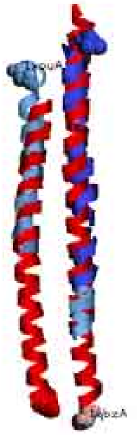
	1qbz:A (103)	PROTEIN (SIV GP41 ECTODOMAIN)								
1096	1qbz:A (103)	PROTEIN (SIV GP41 ECTODOMAIN)	**39**	49	3E-07	0.57	3.33	79	1.3% : 8.9% (1/78 : 7/79)	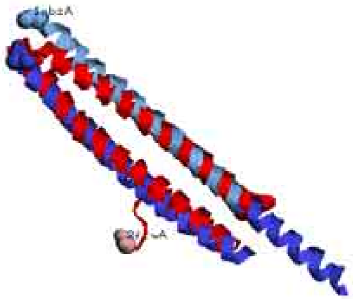
	2fcw:A (106)	Alpha-2-macroglobulin receptor-associated pro								
1097	2f15:A (89)	5'-AMP-activated protein kinase, beta-2 subun	**32**	31	0.052	0.25	3.20	54	2.5% : 5.6% (1/40 : 3/54)	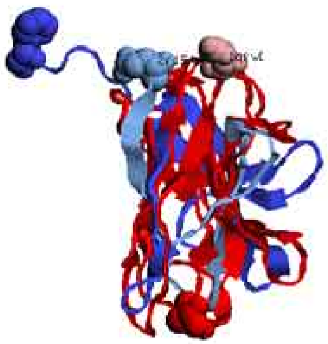
	1qfw:I (120)	ANTIBODY (ANTI BETA SUBUNIT) (HEAVY CHAIN)								

1098	1x8z:A (151)	invertase/pectin methylesterase inhibitor fam	**93**	37	0.001	0.29	3.48	84	7.9% : 6.0% (5/63 : 5/84)	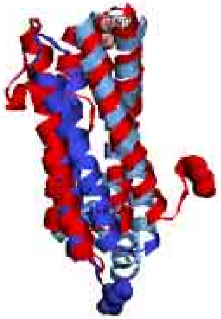
	1qgh:A (150)	NON-HEME IRON-CONTAINING FERRITIN								
1099	1qgh:A (150)	NON-HEME IRON-CONTAINING FERRITIN	**82**	53	1E-08	0.21	3.19	77	2.0% : 9.1% (1/51 : 7/77)	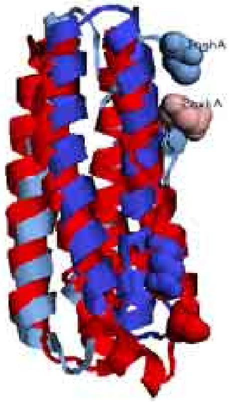
	2avk:A (133)	hemerythrin-like domain protein DcrH								
1100	1sj7:A (167)	Talin 1	**111**	45	6E-06	0.24	2.30	97	9.4% : 10.3% (6/64 : 10/97)	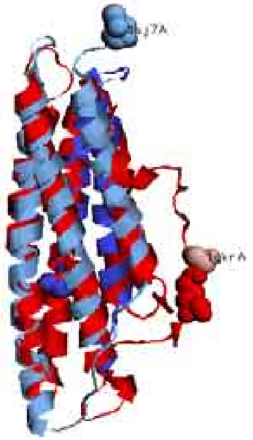
	1qkr:A (181)	VINCULIN								

1101	1yo7:A (120)	Regulatory protein rop	**28**	45	4E-06	0.29	2.41	96	8.9% : 9.4% (5/56 : 9/96)	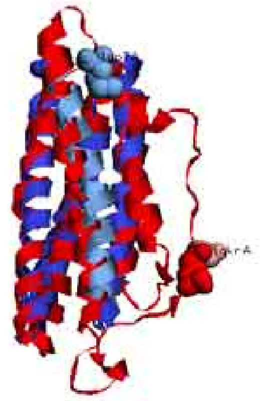
	1qkr:A (181)	VINCULIN								
1102	1qok:A (227)	MFE-23 RECOMBINANT ANTIBODY FRAGMENT	**121**	156	2E-39	0.89	1.64	216	22.6% : 47.7% (43/190 : 103/216)	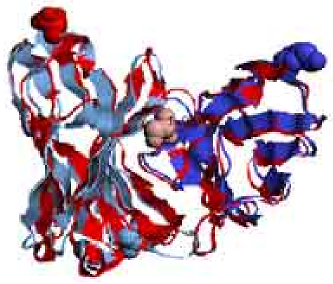
	1x9q:A (232)	4m5.3 anti-fluorescein single chain antibody								
1103	1qok:A (227)	MFE-23 RECOMBINANT ANTIBODY FRAGMENT	**120**	178	5E-46	0.88	1.11	218	24.1% : 53.7% (46/191 : 117/218)	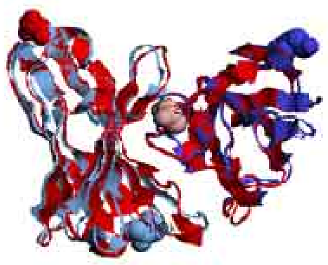
	2gjj:A (237)	A21 single-chain antibody fragment against er								

1104	1z7c:A (165)<br>1qsd:A (102)	Chorionic somatomammotropin hormone<br>PROTEIN (BETA-TUBULIN BINDING POST-CHAPERONIN	**126**	31	0.074	0.35	1.42	63	10.2% : 11.1% (5/49 : 7/63)	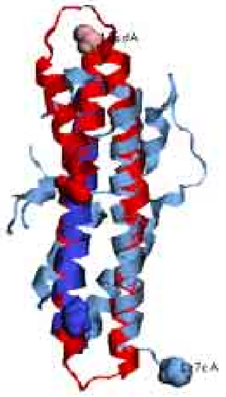
1105	1qsd:A (102)<br>2asr_ (142)	PROTEIN (BETA-TUBULIN BINDING POST-CHAPERONIN<br>ASPARTATE RECEPTOR	**41**	42	3E-05	0.46	2.29	72	3.1% : 5.6% (2/64 : 4/72)	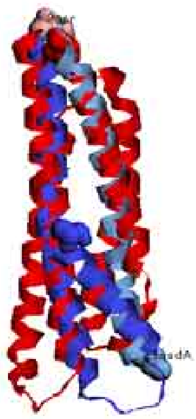
1106	1yad:A (191)<br>1qwg:A (251)	Regulatory protein tenI<br>(2R)-phospho-3-sulfolactate synthase	**94**	35	0.007	0.48	2.79	153	10.4% : 13.7% (15/144 : 21/153)	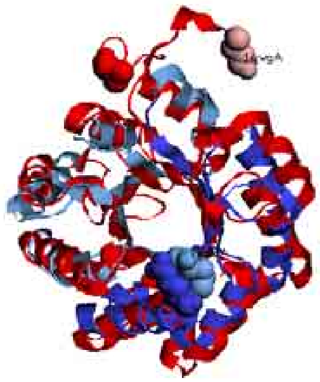

1107	1r03:A (170)	mitochondrial ferritin	60	40	0.0001	0.21	3.27	79	6.8% : 11.4% (5/74 : 9/79)	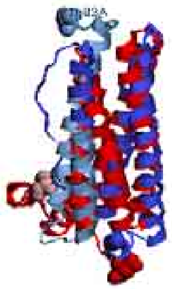
	1x8z:A (151)	invertase/pectin methylesterase inhibitor fam								
1108	1yjg:A (157)	Surface protein VspA	34	42	3E-05	0.23	3.47	85	5.0% : 11.8% (3/60 : 10/85)	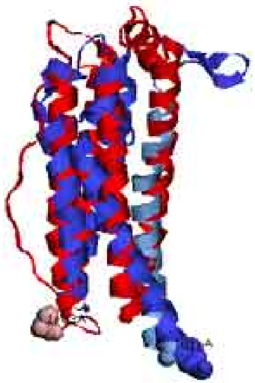
	1r03:A (170)	mitochondrial ferritin								
1109	1r03:A (170)	mitochondrial ferritin	91	39	0.0003	0.41	2.41	84	11.0% : 9.5% (8/73 : 8/84)	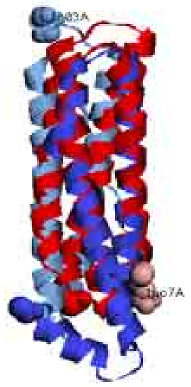
	1yo7:A (120)	Regulatory protein rop								

1110	1z7c:A (165)	Chorionic somatomammotropin hormone	**44**	34	0.01	0.24	3.14	87	6.1% : 3.4% (3/49 : 3/87)	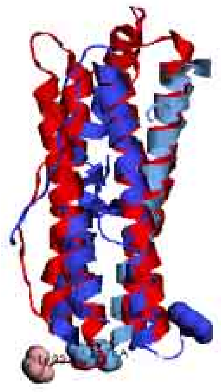
	1r03:A (170)	mitochondrial ferritin								
1111	2ah6:A (152)	BH1595, unknown conserved protein	**82**	35	0.005	0.34	2.87	88	8.1% : 13.6% (5/62 : 12/88)	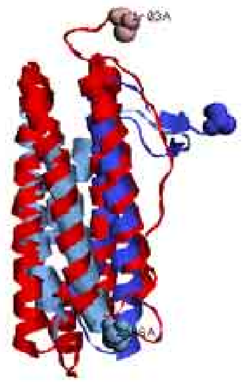
	1r03:A (170)	mitochondrial ferritin								
1112	1z23:A (163)	CRK-associated substrate	**121**	37	0.001	0.29	2.94	111	8.2% : 8.1% (5/61 : 9/111)	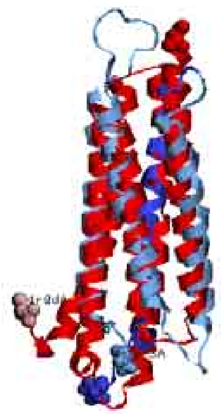
	1r0d:A (194)	Huntingtin Interacting Protein 12								

1113	2asr:_ (142)	ASPARTATE RECEPTOR	**101**	55	4E-09	0.31	2.86	104	12.5% : 8.7% (10/80 : 9/104)	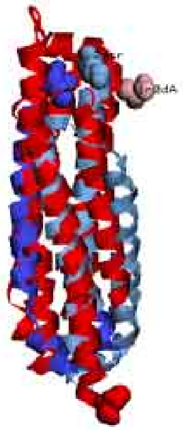
	1r0d:A (194)	Huntingtin Interacting Protein 12								
1114	2fr2:A (161)	hypothetical protein Rv2717c	**74**	40	0.0002	0.39	3.42	102	7.0% : 7.8% (7/100 : 8/102)	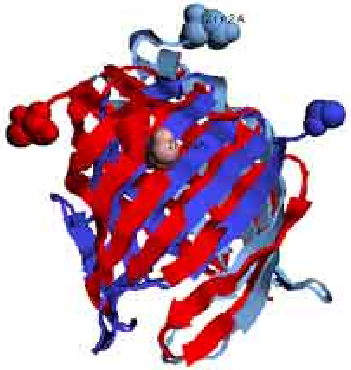
	1r0u:A (144)	protein ywiB								
1115	* 1r4w:A (221)	Glutathione S-transferase, mitochondrial	**129**	38	0.0009	0.48	2.93	137	22.1% : 16.1% (15/68 : 22/137)	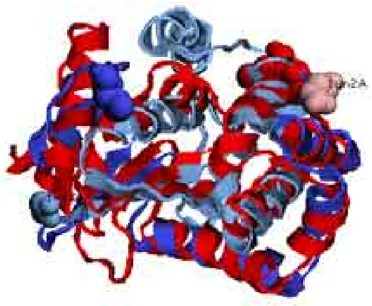
	* 1un2:A (186)	THIOL-DISULFIDE INTERCHANGE PROTEIN								

1116	1r73:A (66)	50S ribosomal protein L29	**34**	34	0.007	0.28	2.28	47	4.0% : 14.9% (1/25 : 7/47)	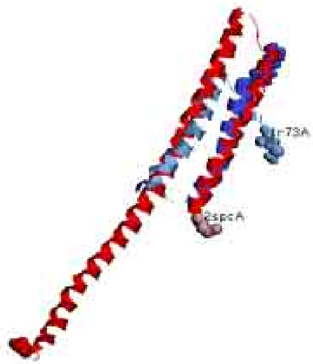
	2spc:A (107)	SPECTRIN								
1117	2b4l:A (268)	Glycine betaine-binding protein	**158**	97	2E-21	0.75	1.96	253	22.0% : 22.9% (33/150 : 58/253)	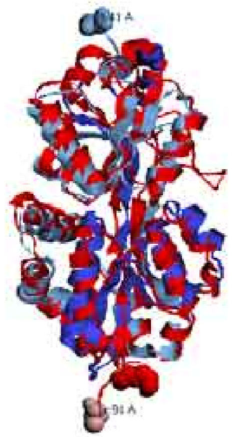
	1r9l:A (309)	Glycine betaine-binding periplasmic protein								
1118	1rcc:_ (171)	L FERRITIN	**59**	48	7E-07	0.22	3.19	83	7.1% : 8.4% (4/56 : 7/83)	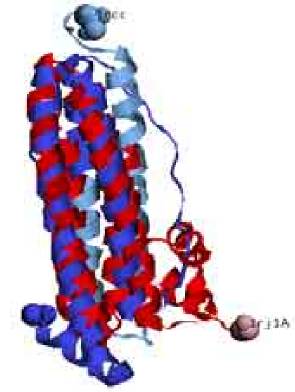
	1rj1:A (148)	invertase inhibitor								

1119	1rcc:_<br>(171)<br>1x8z:A<br>(151)	L FERRITIN<br>invertase/pectin methylesterase inhibitor fam	**60**	41	0.0001	0.22	2.44	76	6.9% : 10.5%<br>(4/58 : 8/76)	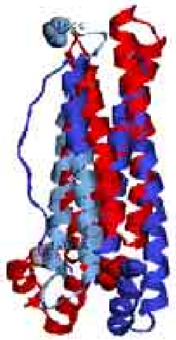
1120	1vpx:A<br>(208)<br>1rd5:A<br>(261)	PROTEIN (Transaldolase (EC 2.2.1.2))<br>Tryptophan synthase alpha chain, chloroplast	**133**	40	0.0003	0.26	2.92	142	13.6% : 19.0%<br>(17/125 : 27/142)	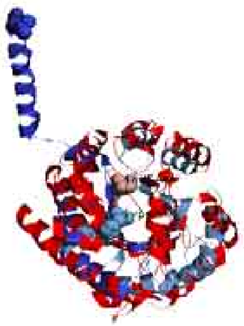
1121	1wx0:A<br>(211)<br>1rd5:A<br>(261)	transaldolase<br>Tryptophan synthase alpha chain, chloroplast	**150**	40	0.0003	0.27	2.71	145	15.5% : 14.5%<br>(22/142 : 21/145)	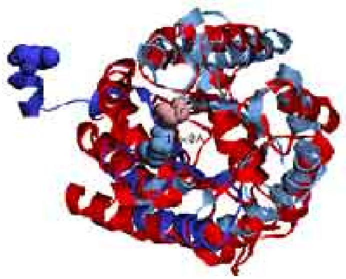

1122	1zke:A (81)<br>1rfy:A (89)	Hypothetical protein HP1531<br>Transcriptional repressor traM	**33**	33	0.018	0.44	1.64	55	15.1% : 7.3% (8/53 : 4/55)	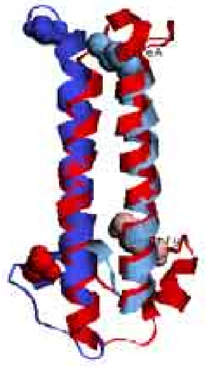
1123	1rhc:A (330)<br>1vs1:A (270)	F420-dependent alcohol dehydrogenase<br>3-deoxy-7-phosphoheptulonate synthase	**206**	32	0.099	0.26	2.70	163	7.5% : 12.3% (12/159 : 20/163)	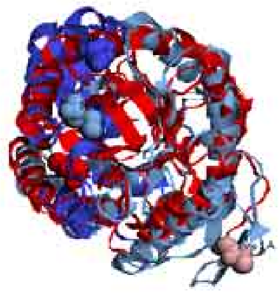
1124	1rhc:A (330)<br>1zco:A (262)	F420-dependent alcohol dehydrogenase<br>2-dehydro-3-deoxyphosphoheptonate aldolase	**204**	32	0.099	0.27	2.77	162	11.2% : 13.0% (18/161 : 21/162)	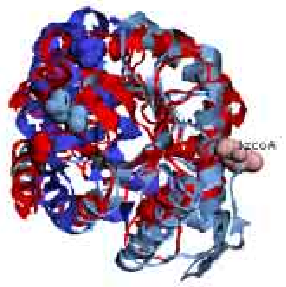

1125	1yjg:A<br>(157)<br>1rj1:A<br>(148)	Surface protein VspA<br>invertase inhibitor	**94**	37	0.0008	0.36	3.18	99	18.6% : 11.1%<br>(11/59 : 11/99)	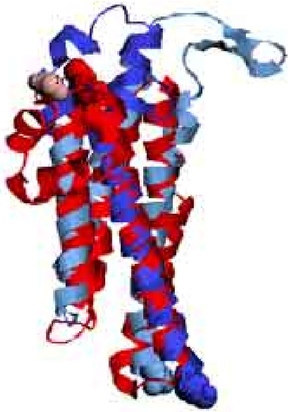
1126	1rj1:A<br>(148)<br>2d4x:A<br>(214)	invertase inhibitor<br>Flagellar hook-associated protein 3	**66**	38	0.0006	0.25	2.12	94	5.2% : 8.5%<br>(3/58 : 8/94)	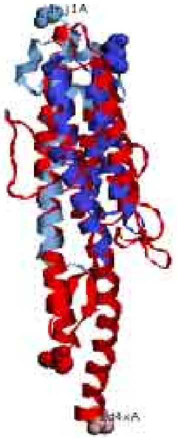
1127	2i8e:A<br>(88)<br>1rlh:A<br>(151)	Hypothetical protein<br>conserved hypothetical protein	**30**	32	0.044	0.21	2.63	53	7.9% : 7.5%<br>(3/38 : 4/53)	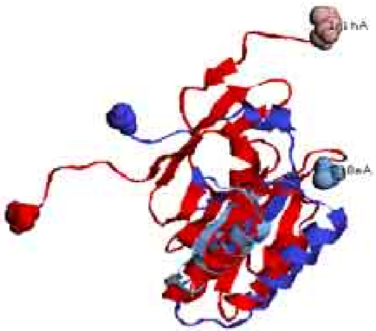

1128	1wrd:A (98)	Target of Myb protein 1	**38**	33	0.013	0.35	2.83	77	13.5% : 9.1% (10/74 : 7/77)	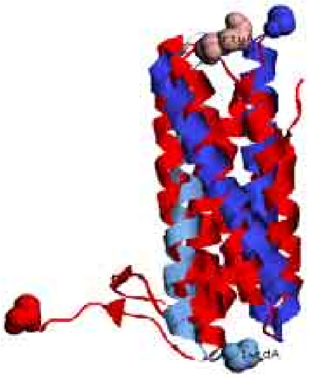
	1rty:A (159)	yvqk protein								
1129	1rty:A (159)	yvqk protein	**83**	35	0.006	0.47	3.45	104	15.5% : 5.8% (15/97 : 6/104)	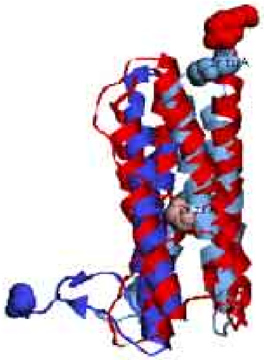
	2fzf:A (158)	hypothetical protein								
1130	1rv8:A (298)	fructose-1,6-bisphosphate aldolase	**226**	46	5E-06	0.22	3.04	150	14.2% : 19.3% (21/148 : 29/150)	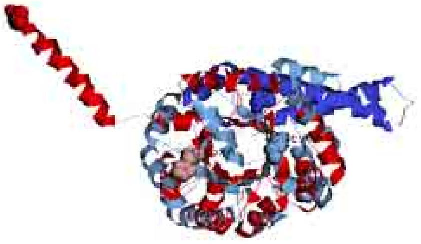
	1vpx:A (208)	PROTEIN (Transaldolase (EC 2.2.1.2))								

1131	1s14:A (171)	Topoisomerase IV subunit B	**127**	35	0.004	0.33	2.71	96	13.7% : 13.5% (10/73 : 13/96)	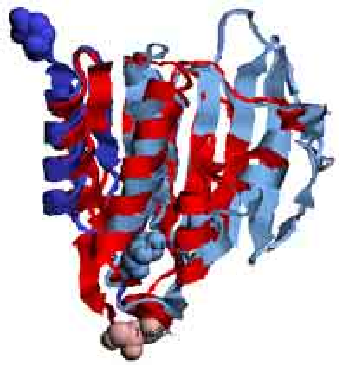
	1ys3:A (138)	Sensor-type histidine kinase prrB								
1132	1s7z:A (106)	Gene 0.3 protein	**73**	30	0.089	0.20	3.42	59	10.3% : 13.6% (4/39 : 8/59)	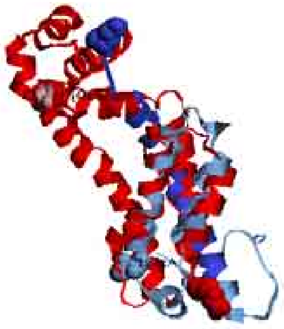
	2fq4:A (184)	Transcriptional regulator, TetR family								
1133	1skv:A (64)	Hypothetical 7.5 kDa protein	**29**	36	0.002	0.38	1.62	56	22.2% : 10.7% (10/45 : 6/56)	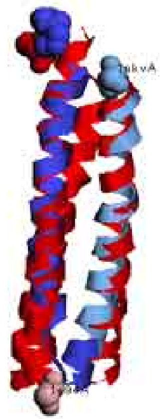
	1s94:A (111)	s-syntaxin								

1134	* 1wp7:A (65)	fusion protein	**30**	37	0.0009	0.37	2.29	58	3.5% : 0.0% (2/57 : 0/58)	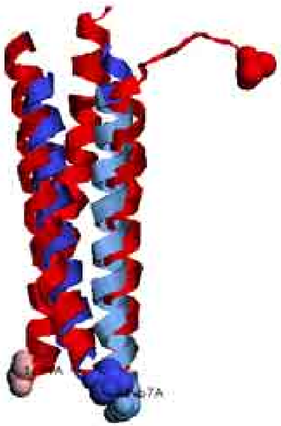
	* 1s94:A (111)	s-syntaxin								
1135	1sgj:A (231)	citrate lyase, beta subunit	**177**	34	0.013	0.36	3.34	146	7.3% : 11.6% (9/124 : 17/146)	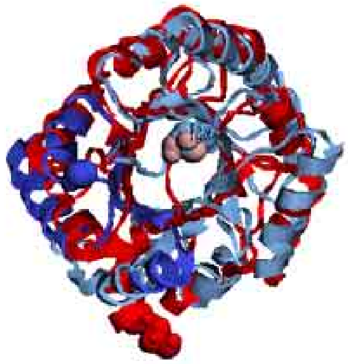
	1y0e:A (222)	Putative N-acetylmannosamine-6-phosphate 2-ep								
1136	1sj7:A (167)	Talin 1	**126**	47	1E-06	0.24	3.00	101	5.9% : 6.9% (4/68 : 7/101)	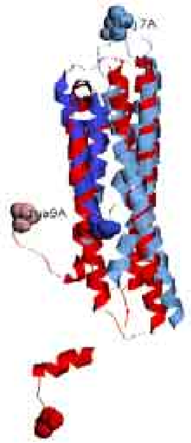
	1ya9:A (169)	Apolipoprotein E								

1137	2j3t:C (141)	TRAFFICKING PROTEIN PARTICLE COMPLEX SUBUNIT	**124**	41	7E-05	0.25	3.44	95	12.0% : 11.6% (10/83 : 11/95)	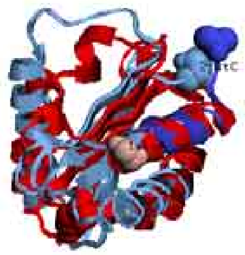
	1sko:A (119)	Mitogen-activated protein kinase kinase 1 int								
1138	1tit:_ (89)	TITIN, I27	**53**	33	0.015	0.24	3.11	51	11.6% : 3.9% (5/43 : 2/51)	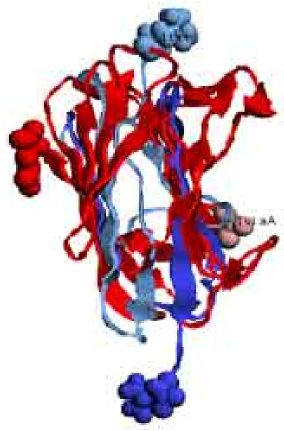
	1sla:A (134)	BOVINE GALECTIN-1								
1139	1woz:A (159)	177aa long conserved hypothetical protein (ST	**81**	41	5E-05	0.41	2.34	97	5.5% : 11.3% (5/91 : 11/97)	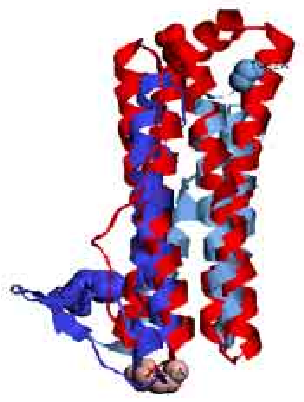
	1sof:A (155)	Bacterioferritin								

1140	1yjg:A (157)	Surface protein VspA	**122**	48	6E-07	0.25	1.90	83	3.9% : 4.8% (3/77 : 4/83)	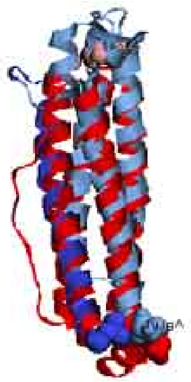
	1sof:A (155)	Bacterioferritin								
1141	2g2d:A (160)	ATP:cobalamin adenosyltransferase	**90**	33	0.024	0.42	2.11	98	6.3% : 12.2% (6/95 : 12/98)	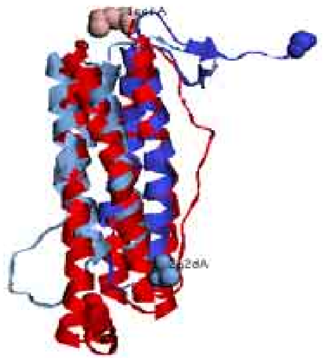
	1sof:A (155)	Bacterioferritin								
1142	1spv:A (172)	putative polyprotein/phosphatase	**68**	39	0.0003	0.21	3.44	88	17.7% : 17.0% (11/62 : 15/88)	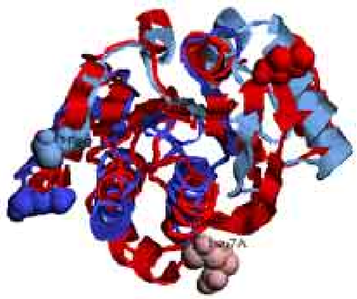
	1wy7:A (197)	hypothetical protein PH1948								

1143	2b4l:A (268)	Glycine betaine-binding protein	**165**	39	0.0006	0.64	2.42	212	11.7% : 14.6% (17/145 : 31/212)	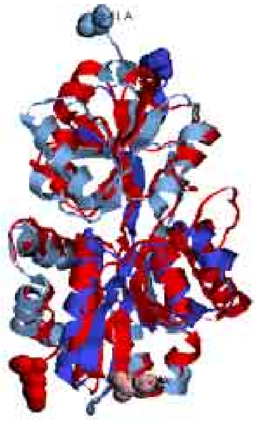
	1sw1:A (270)	osmoprotection protein (proX)								
1144	* 1wbi:A (123)	AVIDIN-RELATED PROTEIN 2	**42**	36	0.002	0.56	1.06	97	26.1% : 29.9% (18/69 : 29/97)	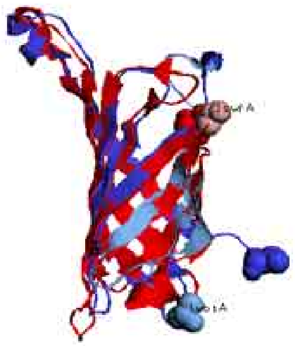
	* 1swf:A (117)	CIRCULARLY PERMUTED CORE-STREPTAVIDIN E51/A46								
1145	* 1y52:X (120)	Avidin-related protein 4/5	**39**	43	2E-05	0.59	1.03	97	32.4% : 35.1% (22/68 : 34/97)	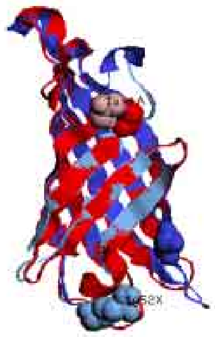
	* 1swf:A (117)	CIRCULARLY PERMUTED CORE-STREPTAVIDIN E51/A46								

1146	1sy6:A (169)	T-cell surface glycoprotein CD3 gamma/epsilon	**63**	31	0.077	0.22	3.38	90	21.3% : 16.7% (13/61 : 15/90)	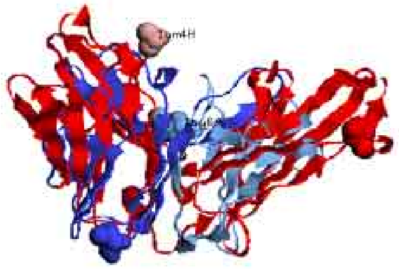
	1um4:H (217)	Antibody 21H3 H chain								
1147	* 1xmw:A (178)	Chimeric CD3 mouse Epsilon and sheep Delta Ec	**93**	47	2E-06	0.50	2.87	120	27.5% : 49.2% (28/102 : 59/120)	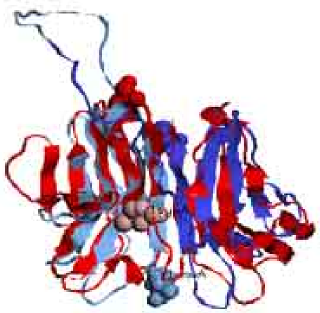
	* 1sy6:A (169)	T-cell surface glycoprotein CD3 gamma/epsilon								
1148	1t6i:A (111)	Superoxide dismutase [Ni]	**24**	35	0.003	0.29	2.47	79	4.8% : 3.8% (3/62 : 3/79)	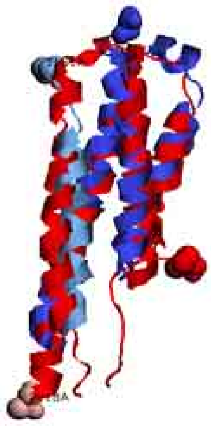
	2etd:A (145)	lemA protein								

1149	1t6i:A (111)	Superoxide dismutase [Ni]	**67**	33	0.021	0.23	1.98	59	3.8% : 10.2% (2/52 : 6/59)	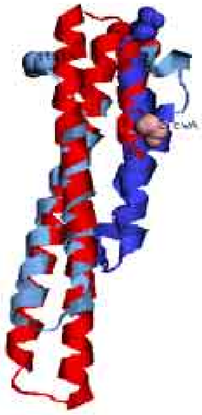
	2fcw:A (106)	Alpha-2-macroglobulin receptor-associated pro								
1150	2fi1:A (187)	hydrolase, haloacid dehalogenase-like family	**135**	36	0.004	0.26	3.49	69	13.3% : 14.5% (8/60 : 10/69)	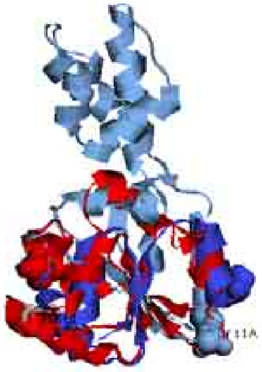
	1t6t:1 (108)	putative protein								
1151	2asr:_ (142)	ASPARTATE RECEPTOR	**67**	64	6E-12	0.44	2.86	114	9.0% : 8.8% (9/100 : 10/114)	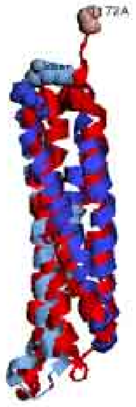
	1t72:A (215)	Phosphate transport system protein phoU homol								

1152	2dtt:A (106)	Hypothetical protein PH0634	**46**	31	0.074	0.34	2.99	56	2.6% : 7.1% (1/39 : 4/56)	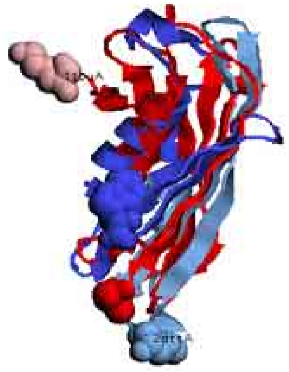
	1tbu:A (97)	Peroxisomal acyl-coenzyme A thioester hydrola								
1153	1zuj:A (168)	hypothetical protein Llacc01001955	**103**	31	0.091	0.29	2.73	57	0.0% : 3.5% (0/40 : 2/57)	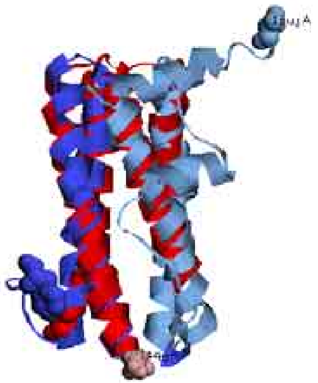
	1tqg:A (105)	Chemotaxis protein cheA								
1154	1tqj:A (217)	Ribulose-phosphate 3-epimerase	**73**	32	0.062	0.23	3.32	151	8.9% : 15.2% (11/124 : 23/151)	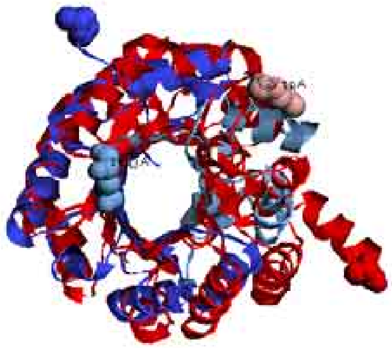
	2ftp:A (300)	hydroxymethylglutaryl-CoA lyase								

1155	* 1tuc:_ (65)	ALPHA-SPECTRIN	**43**	40	0.0002	0.38	1.67	49	30.0% : 34.7% (12/40 : 17/49)	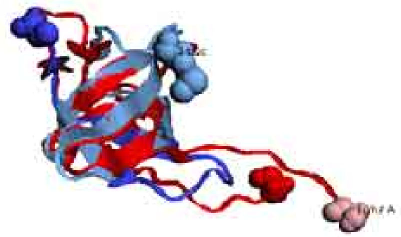
	* 1uhf:A (69)	INTERSECTIN 2								
1156	* 1uj0:A (58)	signal transducing adaptor molecule (SH3 doma	**17**	38	0.0006	0.60	1.58	52	30.8% : 32.7% (12/39 : 17/52)	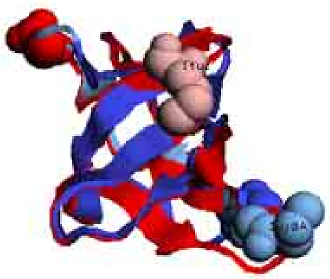
	* 1tuc_ (65)	ALPHA-SPECTRIN								
1157	* 1tuc:_ (63)	ALPHA-SPECTRIN	**41**	36	0.002	0.32	1.88	48	30.2% : 29.2% (13/43 : 14/48)	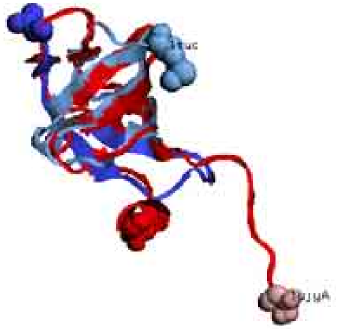
	* 1ujy:A (76)	Rho guanine nucleotide exchange factor 6								

1158	* 1w1f:A (60)	TYROSINE-PROTEIN KINASE LYN	19	39	0.0002	0.53	1.81	49	35.1% : 36.7% (13/37 : 18/49)	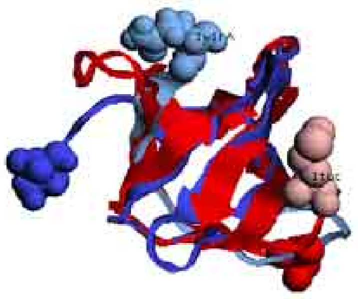
	* 1tuc_ (65)	ALPHA-SPECTRIN								
1159	* 1tuc:_ (65)	ALPHA-SPECTRIN	47	32	0.032	0.43	1.43	49	40.0% : 38.8% (16/40 : 19/49)	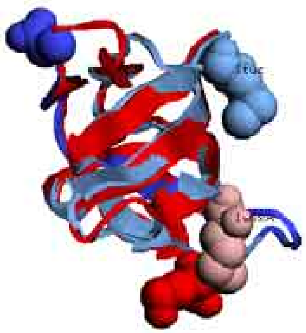
	* 1w6x:A (56)	NEUTROPHIL CYTOSOL FACTOR 4								
1160	* 1wyx:A (68)	CRK-associated substrate	15	33	0.022	0.56	1.60	57	36.4% : 36.8% (16/44 : 21/57)	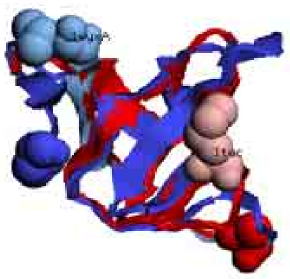
	* 1tuc_ (65)	ALPHA-SPECTRIN								

1161	* 1x2k:A (68)	Osteoclast stimulating factor 1	**19**	35	0.004	0.50	1.63	54	29.3% : 27.8% (12/41 : 15/54)	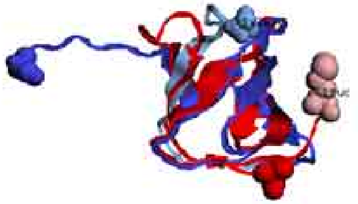
	* 1tuc_ (65)	ALPHA-SPECTRIN								
1162	* 1tuc:_ (65)	ALPHA-SPECTRIN	**44**	32	0.027	0.39	1.42	49	29.7% : 30.6% (11/37 : 15/49)	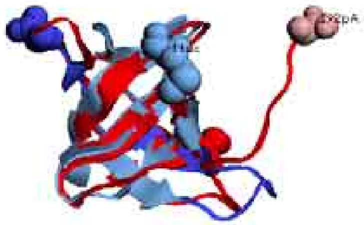
	* 1x2p:A (68)	Protein arginine N-methyltransferase 2								
1163	* 1ynz:A (57)	Pin3p	**14**	34	0.006	0.49	1.26	50	36.6% : 38.0% (15/41 : 19/50)	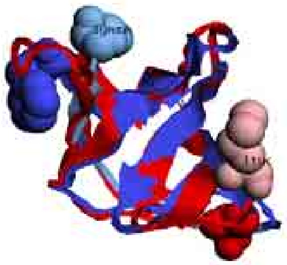
	* 1tuc_ (65)	ALPHA-SPECTRIN								

1164	* 1zsg:A (65)	Rho guanine nucleotide exchange factor 7	**20**	33	0.019	0.50	1.75	54	29.3% : 27.8% (12/41 : 15/54)	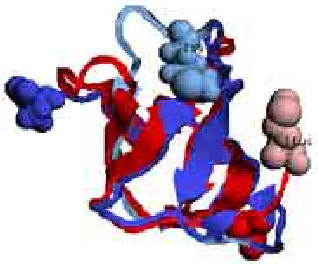
	* 1tuc_ (65)	ALPHA-SPECTRIN								
1165	* 2a08:A (59)	Hypothetical 41.8 kDa protein in SPO13-ARG4 i	**15**	36	0.002	0.59	1.51	52	37.5% : 34.6% (15/40 : 18/52)	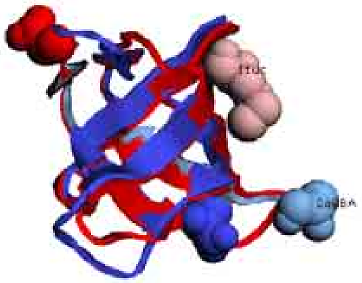
	* 1tuc_ (65)	ALPHA-SPECTRIN								
1166	* 2ak5:A (60)	Rho guanine nucleotide exchange factor 7	**18**	32	0.038	0.52	1.18	53	29.3% : 28.3% (12/41 : 15/53)	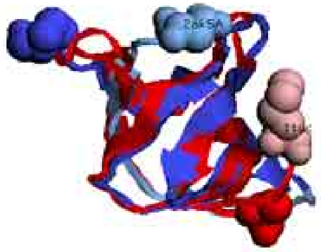
	* 1tuc_ (65)	ALPHA-SPECTRIN								

1167	* 1tuc:_ (63)	ALPHA-SPECTRIN	47	34	0.009	0.44	1.48	48	38.5% : 37.5% (15/39 : 18/48)	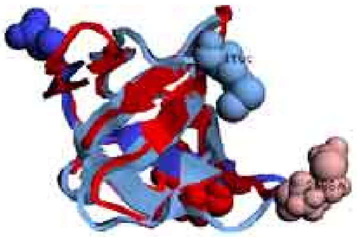
	* 2b86:A (67)	Cytoplasmic protein NCK2								
1168	* 2bz8:A (56)	SH3-DOMAIN KINASE BINDING PROTEIN 1	13	32	0.032	0.54	1.46	51	28.2% : 27.5% (11/39 : 14/51)	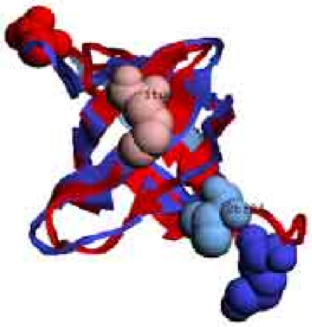
	* 1tuc_ (65)	ALPHA-SPECTRIN								
1169	* 2cre:A (71)	HEF-like protein	19	32	0.038	0.45	1.68	53	39.0% : 39.6% (16/41 : 21/53)	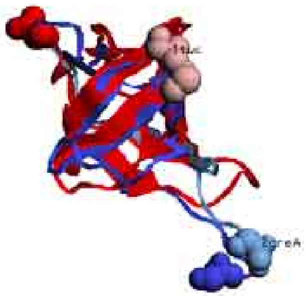
	* 1tuc_ (63)	ALPHA-SPECTRIN								

1170	* 1tuc:_ (63)	ALPHA-SPECTRIN	44	33	0.023	0.30	1.17	47	28.2% : 34.0% (11/39 : 16/47)	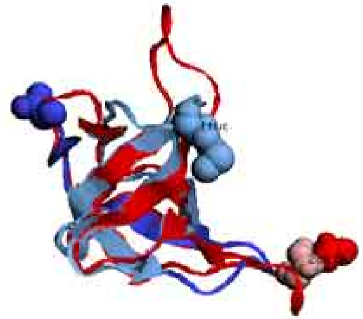
	* 2d8j:A (77)	fyn-related kinase								
1171	* 1tuc:_ (63)	ALPHA-SPECTRIN	44	37	0.0008	0.40	1.46	51	44.2% : 37.3% (19/43 : 19/51)	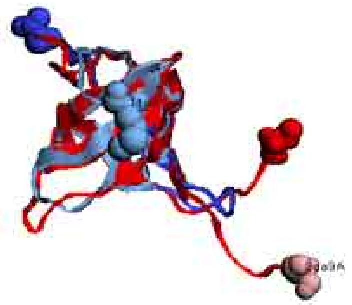
	* 2da9:A (70)	SH3-domain kinase binding protein 1								
1172	* 1tuc:_ (65)	ALPHA-SPECTRIN	44	39	0.0002	0.34	1.49	50	37.5% : 36.0% (15/40 : 18/50)	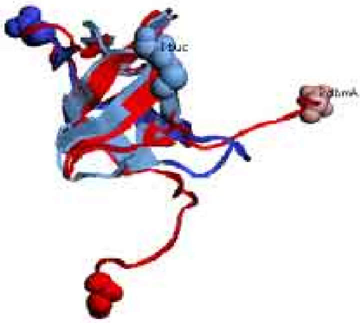
	* 2dbm:A (73)	SH3-containing GRB2-like protein 2								

1173	* 1tuc:_ (65)	ALPHA-SPECTRIN	44	33	0.023	0.37	1.50	49	28.2% : 28.6% (11/39 : 14/49)	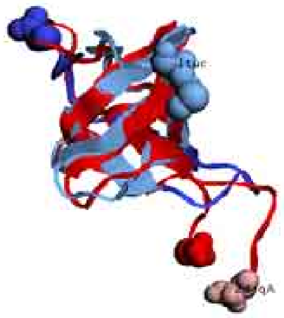
	* 2djq:A (68)	SH3 domain containing ring finger 2								
1174	* 1tuc:_ (65)	ALPHA-SPECTRIN	44	32	0.032	0.34	1.31	49	27.3% : 26.5% (12/44 : 13/49)	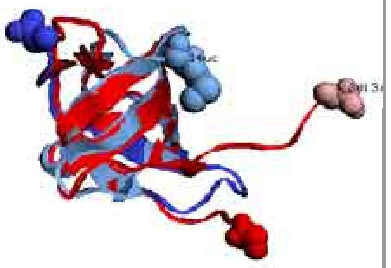
	* 2dl3:A (68)	Sorbin and SH3 domain-containing protein 1								
1175	* 1tuc:_ (65)	ALPHA-SPECTRIN	44	48	4E-07	0.38	1.37	50	48.8% : 44.0% (20/41 : 22/50)	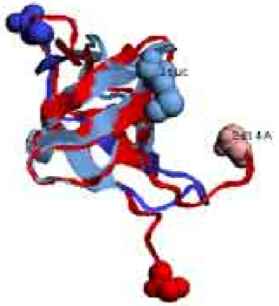
	* 2dl4:A (68)	Protein Stac								

1176	* 2dl7:A (73)	KIAA0769 protein	**20**	32	0.039	0.43	1.77	53	31.7% : 35.8% (13/41 : 19/53)	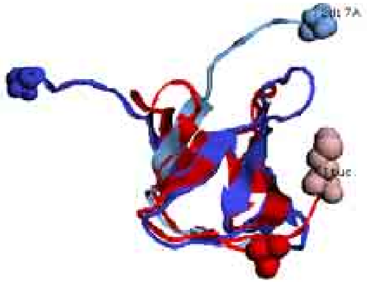
	* 1tuc_ (63)	ALPHA-SPECTRIN								
1177	* 1tuc:_ (65)	ALPHA-SPECTRIN	**44**	36	0.002	0.35	1.41	50	33.3% : 32.0% (14/42 : 16/50)	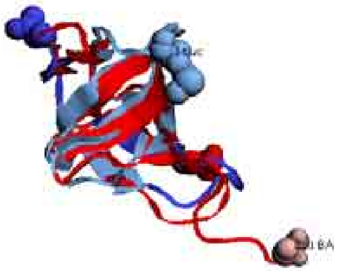
	* 2dl8:A (72)	SLIT-ROBO Rho GTPase-activating protein 2								
1178	* 1tuc:_ (65)	ALPHA-SPECTRIN	**44**	38	0.0004	0.33	1.66	48	39.0% : 35.4% (16/41 : 17/48)	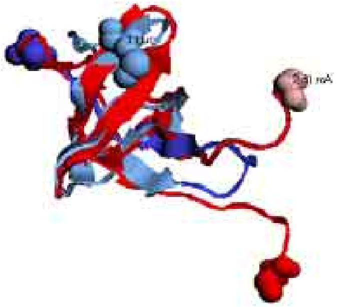
	* 2dlm:A (68)	Vinexin								

1179	* 1tuc:_ (65)	ALPHA-SPECTRIN	**43**	31	0.066	0.31	1.22	48	26.8% : 25.0% (11/41 : 12/48)	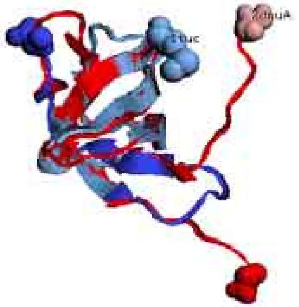
	* 2dnu:A (71)	SH3 multiple domains 1								
1180	* 1tuc:_ (65)	ALPHA-SPECTRIN	**48**	37	0.001	0.47	1.68	51	35.0% : 33.3% (14/40 : 17/51)	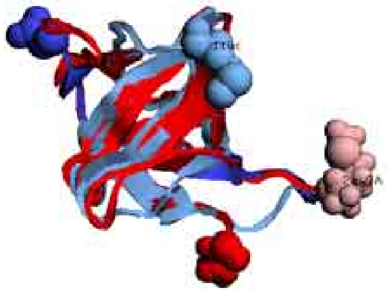
	* 2ew3:A (60)	SH3-containing GRB2-like protein 3								
1181	* 1tuc:_ (65)	ALPHA-SPECTRIN	**50**	32	0.027	0.48	1.64	52	31.7% : 28.8% (13/41 : 15/52)	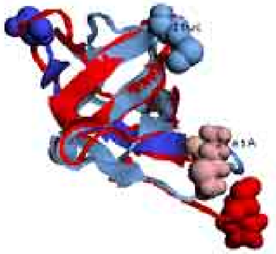
	* 2fei:A (60)	CD2-associated protein								

1182	* 2gnc:A (55)	SLIT-ROBO Rho GTPase-activating protein 1	**18**	36	0.002	0.52	1.05	48	35.9% : 35.4% (14/39 : 17/48)	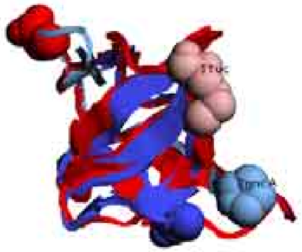
	* 1tuc_ (65)	ALPHA-SPECTRIN								
1183	1z69:A (327)	Coenzyme F420-dependent N(5),N(10)-methylenet	**208**	32	0.082	0.28	2.67	171	10.2% : 14.6% (14/137 : 25/171)	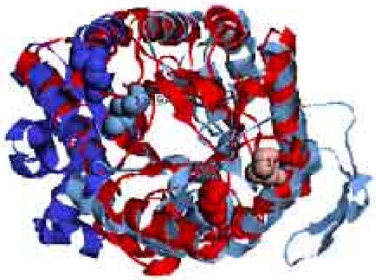
	1tws:A (273)	DHPS, Dihydropteroate synthase								
1184	1u9k:A (110)	triggering receptor expressed on myeloid cell	**56**	40	0.0001	0.26	3.41	65	9.6% : 7.7% (5/52 : 5/65)	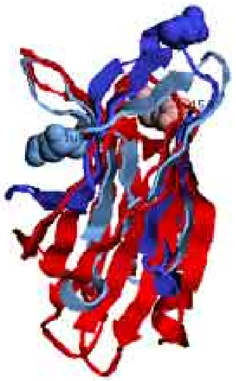
	1w15:A (133)	SYNAPTOTAGMIN IV								

1185	1uta:A (77)	CELL DIVISION PROTEIN FTSN	42	32	0.037	0.41	2.76	51	10.9% : 9.8% (5/46 : 5/51)	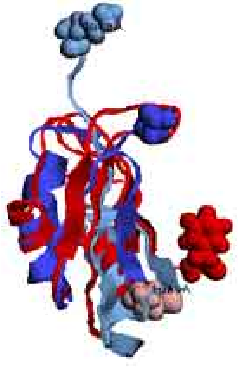
	1uaw:A (77)	mouse-musashi-1								
1186	1x8z:A (151)	invertase/pectin methylesterase inhibitor fam	62	35	0.004	0.25	2.48	88	4.3% : 6.8% (3/69 : 6/88)	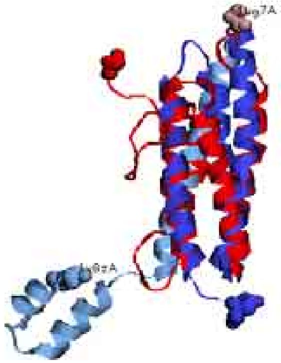
	1ug7:A (128)	2610208M17Rik protein								
1187	2hi7:B (135)	Disulfide bond formation protein B	102	33	0.021	0.23	2.94	70	11.4% : 11.4% (5/44 : 8/70)	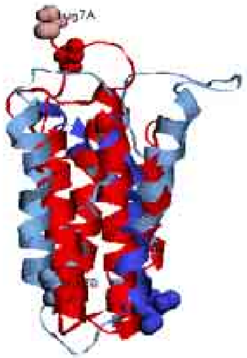
	1ug7:A (128)	2610208M17Rik protein								

1188	1vpx:A (208)<br>1ujp:A (246)	PROTEIN (Transaldolase (EC 2.2.1.2))<br>Tryptophan synthase alpha chain	**134**	39	0.0005	0.26	2.98	143	13.5% : 15.4% (15/111 : 22/143)	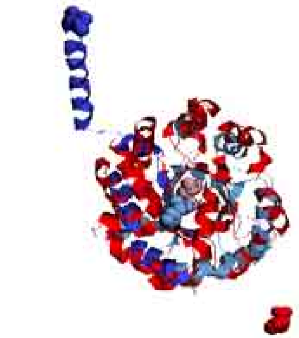
1189	1ul9:A (150)<br>2gu9:A (111)	galectin-2<br>tetracenomycin polyketide synthesis protein	**46**	36	0.002	0.23	3.48	60	5.7% : 13.3% (3/53 : 8/60)	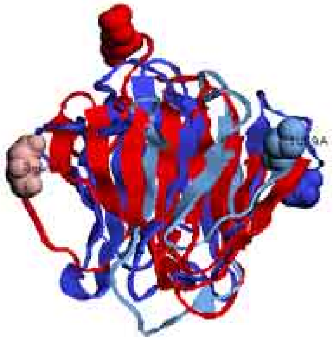
1190	* 1vjq:A (73)<br>* 1ulr:A (87)	designed protein<br>putative acylphosphatase	**35**	32	0.026	0.42	3.21	53	13.6% : 15.1% (6/44 : 8/53)	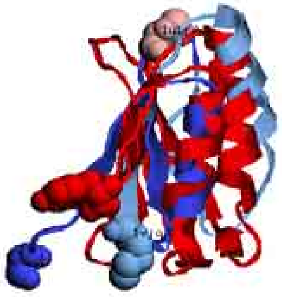

1191	1zhv:A (134)	hypothetical protein Atu0741	**63**	31	0.074	0.24	3.17	63	13.2% : 14.3% (7/53 : 9/63)	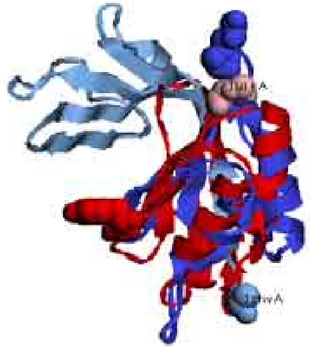
	1ulr:A (87)	putative acylphosphatase								
1192	1wcr:A (103)	PTS SYSTEM, N,N'-DIACETYLCHITOBIOSE-SPECIFIC	**37**	43	1E-05	0.38	3.07	79	8.6% : 12.7% (5/58 : 10/79)	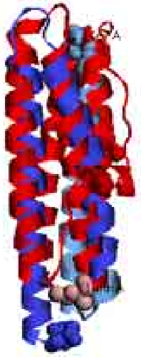
	1umn:A (151)	DPS-LIKE PEROXIDE RESISTANCE PROTEIN								
1193	1umn:A (151)	DPS-LIKE PEROXIDE RESISTANCE PROTEIN	**85**	40	0.0001	0.41	2.75	84	6.1% : 3.6% (5/82 : 3/84)	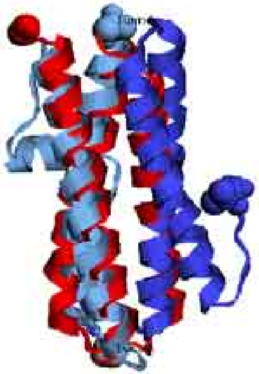
	1wrd:A (98)	Target of Myb protein 1								

1194	2dnx:A (130)	Syntaxin-12	**50**	34	0.006	0.29	2.85	84	9.6% : 4.8% (5/52 : 4/84)	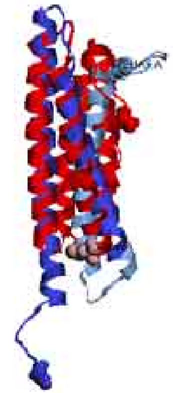
	1umn:A (151)	DPS-LIKE PEROXIDE RESISTANCE PROTEIN								
1195	* 1yzx:A (218)	Glutathione S-transferase kappa 1	**127**	41	0.0001	0.46	2.95	133	18.2% : 15.0% (12/66 : 20/133)	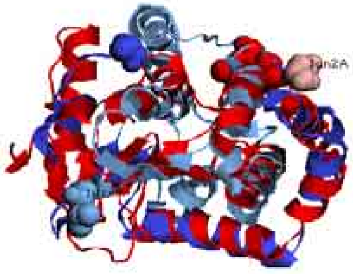
	* 1un2:A (186)	THIOL-DISULFIDE INTERCHANGE PROTEIN								
1196	* 2in3:A (205)	Hypothetical protein	**119**	48	7E-07	0.41	2.11	121	17.2% : 10.7% (11/64 : 13/121)	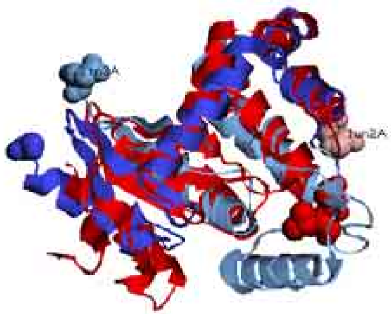
	* 1un2:A (186)	THIOL-DISULFIDE INTERCHANGE PROTEIN								

1197	1zhv:A (134)	hypothetical protein Atu0741	**56**	33	0.021	0.22	2.99	66	12.7% : 12.1% (7/55 : 8/66)	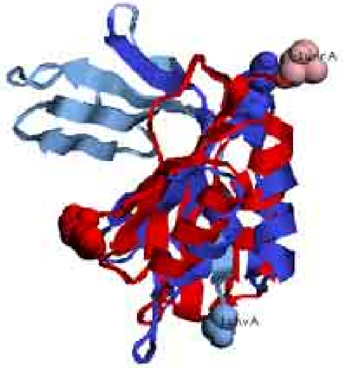
	1urr:A (97)	CG18505 PROTEIN								
1198	1uru:A (217)	AMPHIPHYSIN	**156**	42	7E-05	0.25	2.10	93	6.8% : 14.0% (6/88 : 13/93)	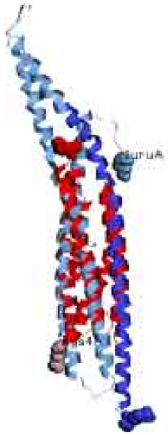
	2gs4:A (159)	Protein yciF								
1199	1uv7:A (77)	GENERAL SECRETION PATHWAY PROTEIN M	**63**	35	0.005	0.27	2.57	61	7.4% : 8.2% (4/54 : 5/61)	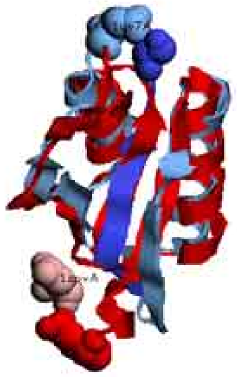
	1zpv:A (86)	ACT domain protein								

1200	1v62:A (117)	KIAA1719 protein	**40**	33	0.021	0.28	2.77	73	28.3% : 23.3% (15/53 : 17/73)	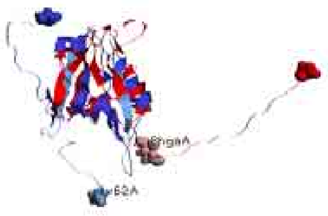
	2hga:A (103)	Conserved protein MTH1368								
1201	1v8e:A (217)	putative glycerophosphoryl diester phosphodie	**88**	35	0.007	0.42	2.88	149	11.2% : 13.4% (15/134 : 20/149)	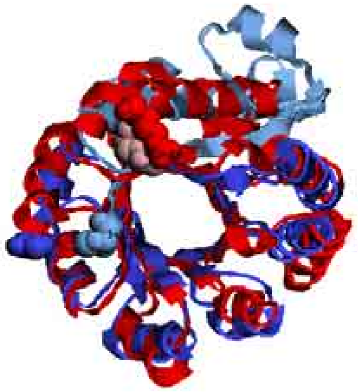
	1yad:A (191)	Regulatory protein tenI								
1202	1vd5:A (377)	unsaturated glucuronyl hydrolase	**97**	68	2E-12	0.36	3.08	258	12.9% : 14.0% (33/256 : 36/258)	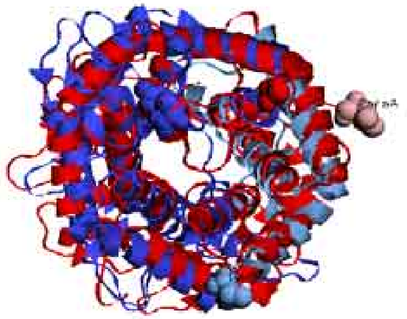
	2afa:A (410)	NAG isomerase								

1203	1vhc:A (212)	Putative KHG/KDPG aldolase	**104**	39	0.0003	0.28	3.37	143	15.1% : 14.7% (16/106 : 21/143)	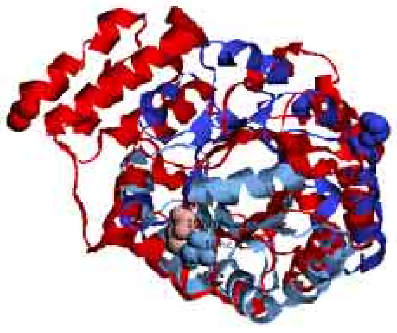
	1vhn:A (305)	putative flavin oxidoreducatase								
1204	1vhc:A (212)	Putative KHG/KDPG aldolase	**172**	38	0.0008	0.20	3.00	131	15.3% : 13.7% (17/111 : 18/131)	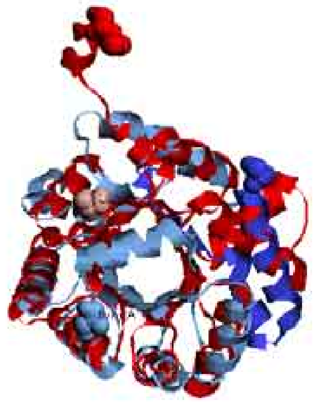
	1wx0:A (211)	transaldolase								
1205	* 1vjq:A (73)	designed protein	**33**	32	0.026	0.55	2.82	61	13.7% : 16.4% (7/51 : 10/61)	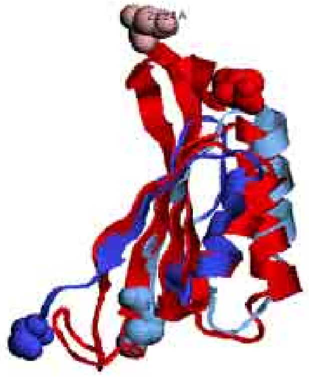
	* 2cz4:A (100)	hypothetical protein TTHA0516								

1206	1vjx:A (148)	putative ferritin-like diiron-carboxylate pro	**89**	40	0.0001	0.35	3.32	85	13.2% : 11.8% (9/68 : 10/85)	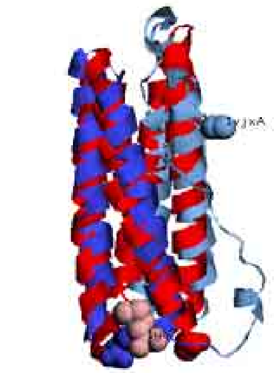
	1yo7:A (120)	Regulatory protein rop								
1207	2avk:A (133)	hemerythrin-like domain protein DcrH	**67**	45	3E-06	0.23	2.41	67	12.2% : 7.5% (6/49 : 5/67)	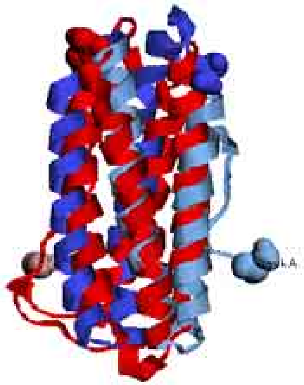
	1vjx:A (148)	putative ferritin-like diiron-carboxylate pro								
1208	1vjx:A (148)	putative ferritin-like diiron-carboxylate pro	**91**	36	0.001	0.40	2.77	77	13.7% : 9.1% (10/73 : 7/77)	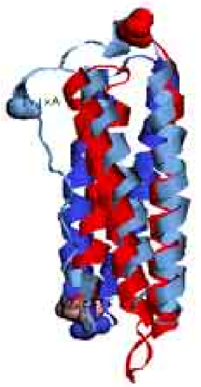
	2c5i:T (94)	T-SNARE AFFECTING A LATE GOLGI COMPARTMENT PR								

1209	1vpx:A (208)<br>1vli:A (359)	PROTEIN (Transaldolase (EC 2.2.1.2))<br>Spore coat polysaccharide biosynthesis protei	**104**	38	0.0007	0.21	3.29	138	10.0% : 8.0% (11/110 : 11/138)	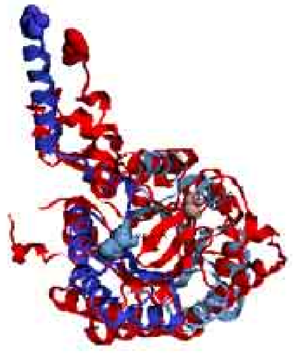
1210	1vpx:A (208)<br>1vlw:A (205)	PROTEIN (Transaldolase (EC 2.2.1.2))<br>2-dehydro-3-deoxyphosphogluconate aldolase/4-	**146**	42	5E-05	0.35	2.88	153	19.7% : 18.3% (23/117 : 28/153)	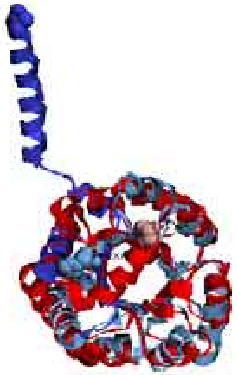
1211	1vlw:A (205)<br>1w37:A (293)	2-dehydro-3-deoxyphosphogluconate aldolase/4-<br>2-KETO-3-DEOXY GLUCONATE ALDOLASE	**152**	31	0.082	0.26	2.85	152	12.2% : 12.5% (17/139 : 19/152)	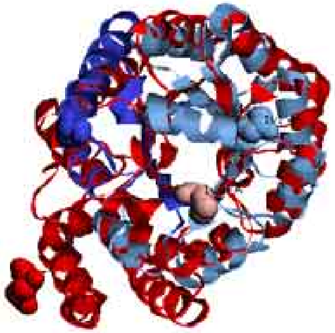

1212	1vlw:A (205)	2-dehydro-3-deoxyphosphogluconate aldolase/4-	159	40	0.0003	0.37	2.84	153	17.9% : 18.3% (21/117 : 28/153)	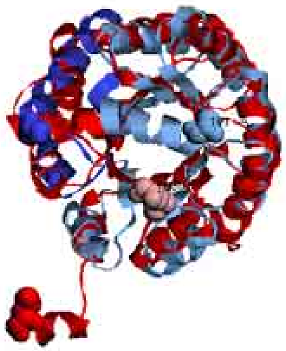
	1wx0:A (211)	transaldolase								
1213	1vp7:A (68)	exodeoxyribonuclease VII small subunit	30	34	0.007	0.55	2.48	54	10.3% : 7.4% (4/39 : 4/54)	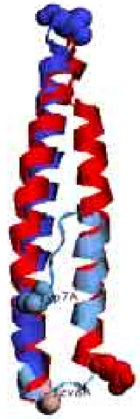
	1zva:A (75)	E2 glycoprotein								
1214	1vp7:A (68)	exodeoxyribonuclease VII small subunit	19	37	0.0009	0.26	2.77	49	5.6% : 12.2% (2/36 : 6/49)	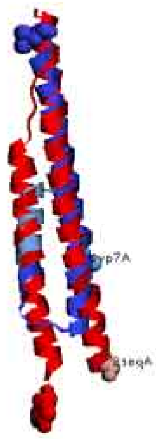
	2ieq:A (89)	Spike glycoprotein								

1215	1vpx:A (208)	PROTEIN (Transaldolase (EC 2.2.1.2))	**101**	46	4E-06	0.24	3.05	146	16.7% : 12.3% (19/114 : 18/146)	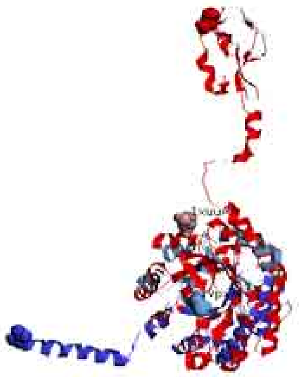
	1xuu:A (348)	polysialic acid capsule biosynthesis protein								
1216	1yad:A (191)	Regulatory protein tenI	**50**	41	0.0001	0.32	2.45	143	22.1% : 12.6% (29/131 : 18/143)	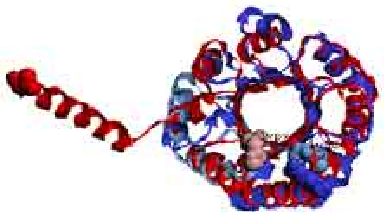
	1vpx:A (208)	PROTEIN (Transaldolase (EC 2.2.1.2))								
1217	1yya:A (250)	triosephosphate isomerase	**203**	32	0.051	0.36	3.36	154	8.0% : 11.0% (9/113 : 17/154)	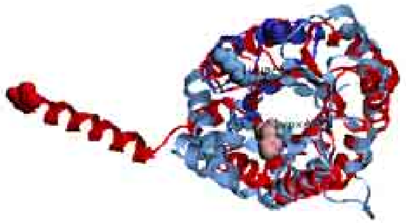
	1vpx:A (208)	PROTEIN (Transaldolase (EC 2.2.1.2))								

1218	1vs1:A (270)	3-deoxy-7-phosphoheptulonate synthase	**86**	35	0.009	0.21	3.31	146	12.1% : 13.0% (15/124 : 19/146)	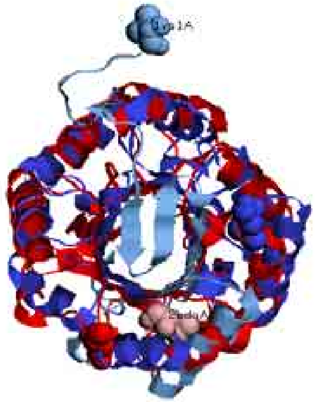
	2bdq:A (210)	copper homeostasis protein CutC								
1219	1vzw:A (226)	PHOSPHORIBOSYL ISOMERASE A	**177**	34	0.013	0.21	2.92	120	10.7% : 11.7% (12/112 : 14/120)	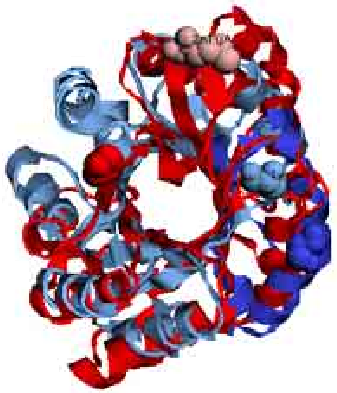
	2nly:A (217)	Divergent polysaccharide deacetylase hypothet								
1220	2avg:A (110)	Myosin-binding protein C, cardiac-type	**54**	41	7E-05	0.21	3.18	66	10.2% : 6.1% (6/59 : 4/66)	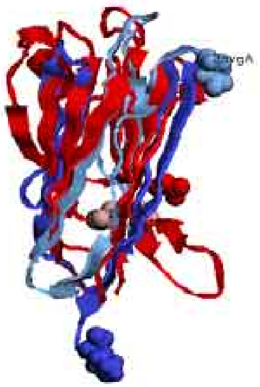
	1w15:A (133)	SYNAPTOTAGMIN IV								

1221	1yx1:A (250)<br>1w37:A (293)	hypothetical protein PA2260<br>2-KETO-3-DEOXY GLUCONATE ALDOLASE	**121**	32	0.061	0.26	3.29	147	9.7% : 11.6% (13/134 : 17/147)	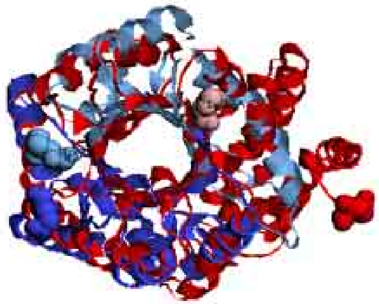
1222	1yad:A (191)<br>1w8g:A (226)	Regulatory protein tenI<br>HYPOTHETICAL UPF0001 PROTEIN YGGS	**70**	31	0.09	0.39	3.31	137	13.3% : 10.9% (18/135 : 15/137)	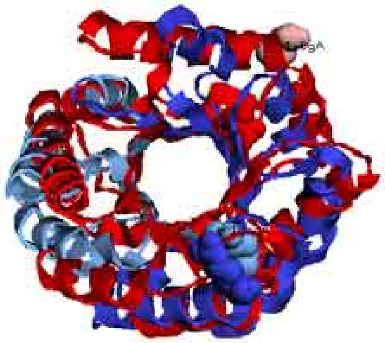
1223	1wap:A (70)<br>2b8m:A (109)	TRP RNA-BINDING ATTENUATION PROTEIN<br>Hypothetical protein MJ0764	**23**	30	0.09	0.34	2.54	54	10.0% : 9.3% (5/50 : 5/54)	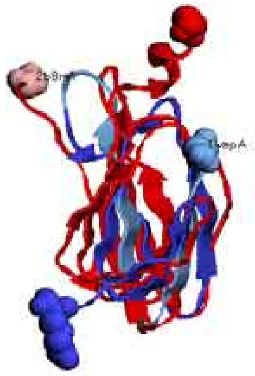

1224	* 2c4i:A (245)		173	90	2E-19	0.33	1.03	115	55.4% : 69.6% (36/65 : 80/115)	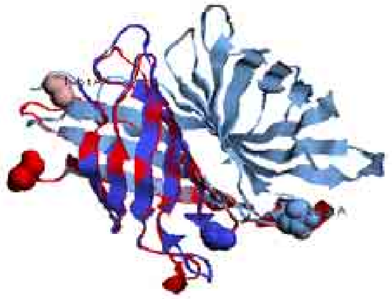
	* 1wbi:A (123)	AVIDIN-RELATED PROTEIN 2								
1225	1wcr:A (103)	PTS SYSTEM, N,N'-DIACETYLCHITOBIOSE-SPECIFIC	42	51	6E-08	0.29	2.71	72	12.1% : 13.9% (7/58 : 10/72)	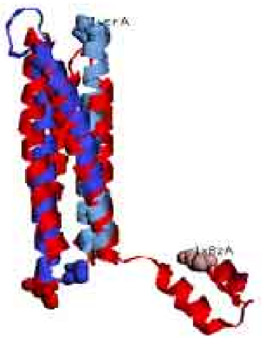
	1x8z:A (151)	invertase/pectin methylesterase inhibitor fam								
1226	1z7c:A (165)	Chorionic somatomammotropin hormone	98	34	0.009	0.28	1.68	56	14.9% : 8.9% (7/47 : 5/56)	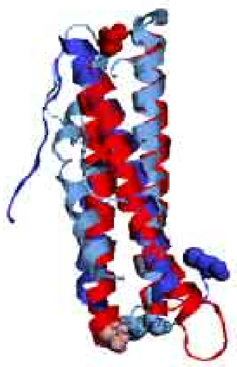
	1wcr:A (103)	PTS SYSTEM, N,N'-DIACETYLCHITOBIOSE-SPECIFIC								

1227	2asr:_ (142)	ASPARTATE RECEPTOR	**76**	37	0.0007	0.56	2.18	87	10.5% : 9.2% (9/86 : 8/87)	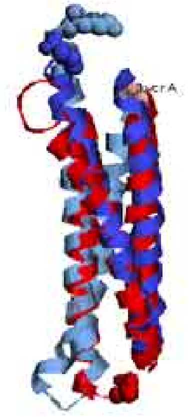
	1wcr:A (103)	PTS SYSTEM, N,N'-DIACETYLCHITOBIOSE-SPECIFIC								
1228	2fjc:A (151)	Antigen TpF1	**92**	43	1E-05	0.42	3.20	86	5.2% : 11.6% (3/58 : 10/86)	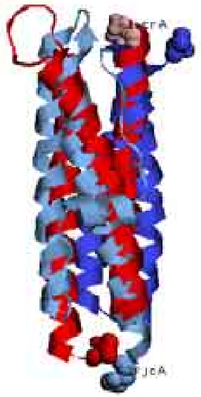
	1wcr:A (103)	PTS SYSTEM, N,N'-DIACETYLCHITOBIOSE-SPECIFIC								
1229	2g38:A (77)	PE FAMILY PROTEIN	**45**	49	3E-07	0.34	2.27	55	14.7% : 7.3% (5/34 : 4/55)	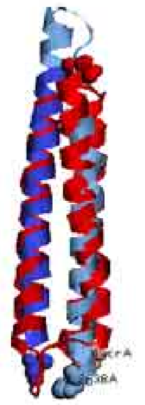
	1wcr:A (103)	PTS SYSTEM, N,N'-DIACETYLCHITOBIOSE-SPECIFIC								

1230	2gs4:A (159)	Protein yciF	**95**	50	2E-07	0.41	3.14	84	11.1% : 9.5% (6/54 : 8/84)	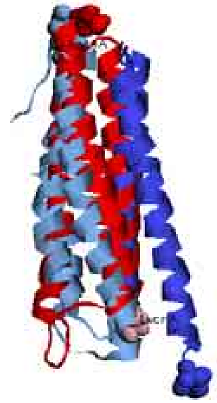
	1wcr:A (103)	PTS SYSTEM, N,N'-DIACETYLCHITOBIOSE-SPECIFIC								
1231	1wdf:A (92)	E2 glycoprotein	**57**	36	0.002	0.42	2.83	72	9.3% : 5.6% (5/54 : 4/72)	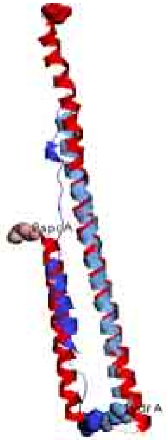
	2spc:A (107)	SPECTRIN								
1232	1wdg:A (82)	E2 glycoprotein	**43**	31	0.062	0.32	2.37	51	13.3% : 3.9% (4/30 : 2/51)	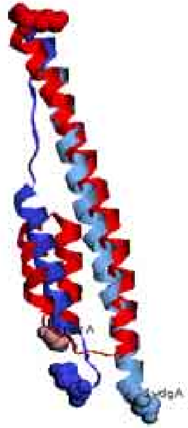
	2fzt:A (78)	hypothetical protein TM0693								

1233	1wdg:A (82)	E2 glycoprotein	**47**	31	0.074	0.45	2.19	57	4.7% : 5.3% (2/43 : 3/57)	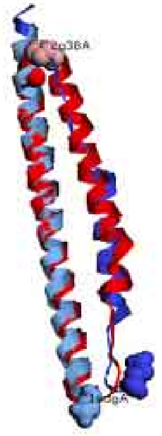
	2g38:A (77)	PE FAMILY PROTEIN								
1234	1y2t:A (142)	lectin	**101**	34	0.009	0.23	2.97	70	14.6% : 10.0% (6/41 : 7/70)	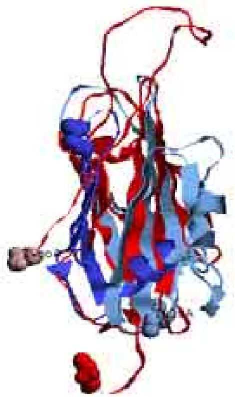
	1wgo:A (123)	VPS10 domain-containing receptor SorCS2								
1235	2hga:A (103)	Conserved protein MTH1368	**59**	35	0.004	0.21	2.00	73	25.0% : 23.3% (14/56 : 17/73)	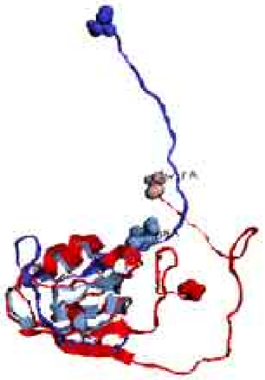
	1wif:A (126)	RIKEN cDNA 4930408O21								

1236	1wlx:A (129)	Alpha-actinin 4	**96**	36	0.002	0.26	3.09	86	9.1% : 15.1% (6/66 : 13/86)	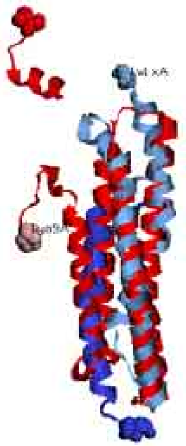
	1ya9:A (169)	Apolipoprotein E								
1237	1wn0:A (131)	histidine-containing phosphotransfer protein	**102**	34	0.006	0.37	2.46	91	9.3% : 9.9% (7/75 : 9/91)	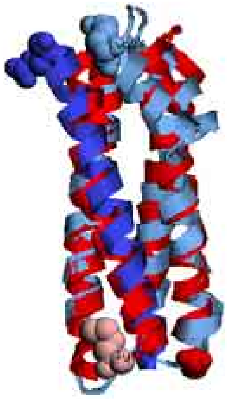
	1yo7:A (120)	Regulatory protein rop								
1238	1wn0:A (131)	histidine-containing phosphotransfer protein	**57**	33	0.015	0.30	3.40	73	4.5% : 13.7% (3/66 : 10/73)	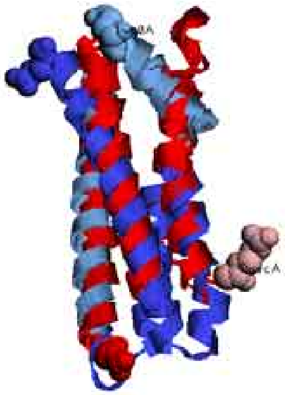
	2gsc:A (114)	conserved hypothetical protein								

1239	1yxr:A (77)	vacuolar protein sorting factor 4A	**22**	33	0.013	0.23	1.89	42	10.0% : 4.8% (3/30 : 2/42)	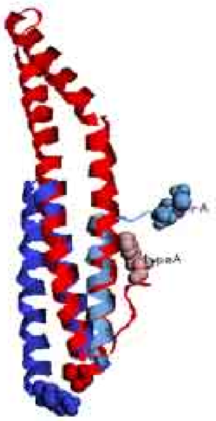
	1wpa:A (107)	Occludin								
1240	1wr6:A (90)	ADP-ribosylation factor binding protein GGA3	**30**	31	0.075	0.40	2.24	66	11.5% : 10.6% (7/61 : 7/66)	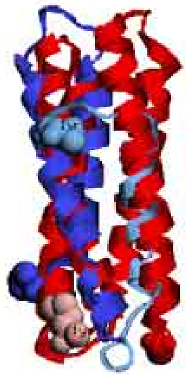
	1yo7:A (120)	Regulatory protein rop								
1241	1wr6:A (90)	ADP-ribosylation factor binding protein GGA3	**28**	41	7E-05	0.23	2.48	67	7.3% : 16.4% (4/55 : 11/67)	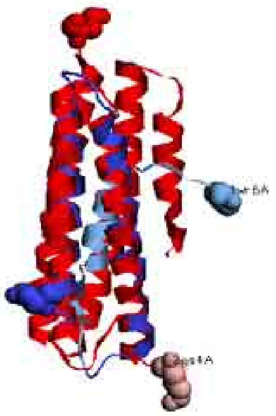
	2gs4:A (159)	Protein yciF								

1242	1z0p:A (74)	hypothetical protein SPy1572	**33**	37	0.001	0.32	1.99	54	5.9% : 5.6% (3/51 : 3/54)	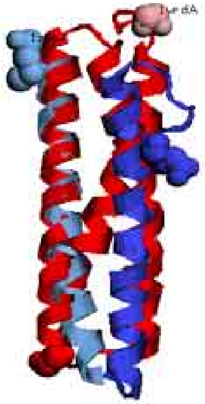
	1wrd:A (98)	Target of Myb protein 1								
1243	2d5k:A (148)	Dps family protein	**83**	40	0.0002	0.40	2.98	83	2.8% : 8.4% (2/72 : 7/83)	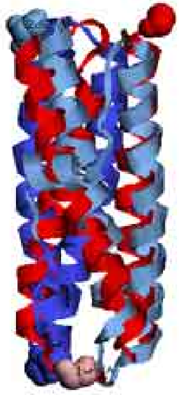
	1wrd:A (98)	Target of Myb protein 1								
1244	2fjc:A (151)	Antigen TpF1	**89**	40	0.0001	0.46	3.17	87	10.5% : 11.5% (9/86 : 10/87)	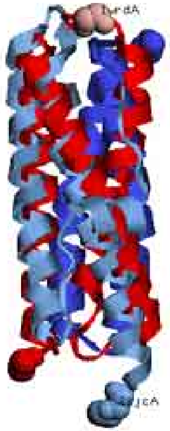
	1wrd:A (98)	Target of Myb protein 1								

1245	1wrd:A (98)	Target of Myb protein 1	**34**	42	3E-05	0.48	3.09	87	8.2% : 10.3% (7/85 : 9/87)	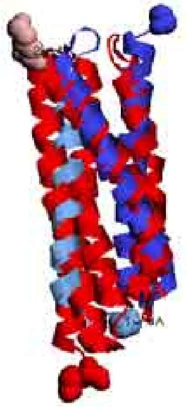
	2gs4:A (159)	Protein yciF								
1246	1wrd:A (98)	Target of Myb protein 1	**71**	38	0.0004	0.41	2.84	70	10.4% : 10.0% (7/67 : 7/70)	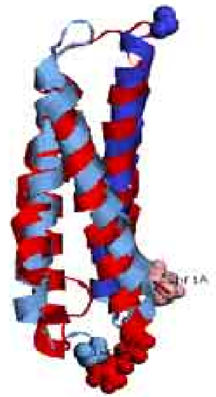
	2hfi:A (123)	Hypothetical protein yppE								
1247	1wrd:A (98)	Target of Myb protein 1	**67**	41	5E-05	0.43	2.38	70	9.2% : 8.6% (6/65 : 6/70)	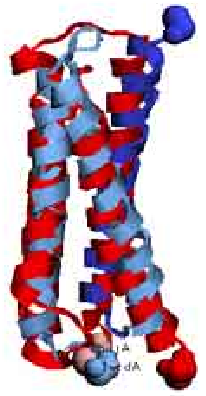
	2huj:A (125)	Lin2004 protein								

1248	1wvt:A (148)	hypothetical protein ST2180	58	34	0.007	0.30	2.52	97	7.5% : 10.3% (7/93 : 10/97)	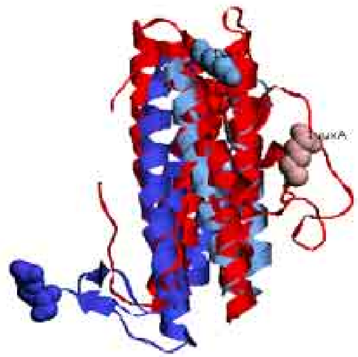
	1yux:A (201)	Nigerythrin								
1249	1yad:A (191)	Regulatory protein tenI	140	39	0.0004	0.45	3.03	150	23.7% : 11.3% (31/131 : 17/150)	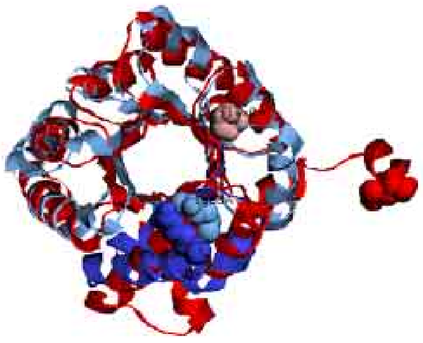
	1wx0:A (211)	transaldolase								
1250	1x8z:A (151)	invertase/pectin methylesterase inhibitor fam	96	42	4E-05	0.24	2.07	76	6.9% : 10.5% (5/72 : 8/76)	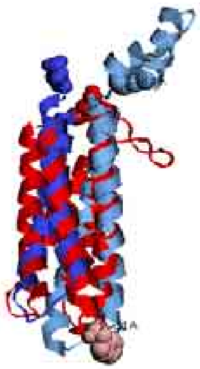
	1wy1:A (142)	hypothetical protein PH0671								

1251	2g38:A (77)	PE FAMILY PROTEIN	**23**	38	0.0004	0.32	1.50	55	5.0% : 9.1% (2/40 : 5/55)	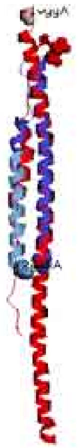
	1wyy:A (127)	E2 Glycoprotein								
1252	2gts:A (77)	hypothetical protein HP0062	**31**	33	0.015	0.37	2.32	55	7.4% : 10.9% (4/54 : 6/55)	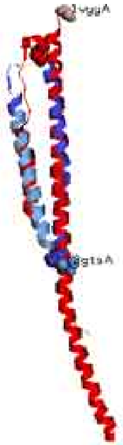
	1wyy:A (127)	E2 Glycoprotein								
1253	1yjg:A (157)	Surface protein VspA	**97**	37	0.001	0.38	2.83	107	9.2% : 9.3% (8/87 : 10/107)	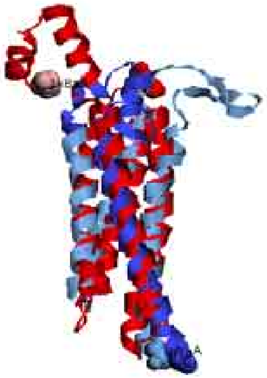
	1x8z:A (151)	invertase/pectin methylesterase inhibitor fam								

1254	1z7c:A (165)	Chorionic somatomammotropin hormone	104	32	0.052	0.25	1.97	80	5.3% : 3.8% (3/57 : 3/80)	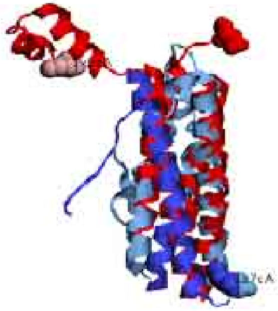
	1x8z:A (151)	invertase/pectin methylesterase inhibitor fam								
1255	2ccy:A (127)	CYTOCHROME C	42	33	0.012	0.27	2.94	89	9.2% : 7.9% (6/65 : 7/89)	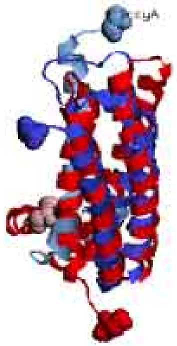
	1x8z:A (151)	invertase/pectin methylesterase inhibitor fam								
1256	2d4x:A (214)	Flagellar hook-associated protein 3	155	51	1E-07	0.22	1.74	88	8.1% : 9.1% (5/62 : 8/88)	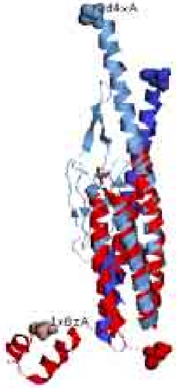
	1x8z:A (151)	invertase/pectin methylesterase inhibitor fam								

1257	1x8z:A (151)	invertase/pectin methylesterase inhibitor fam	123	37	0.0009	0.22	1.67	74	11.3% : 9.5% (6/53 : 7/74)	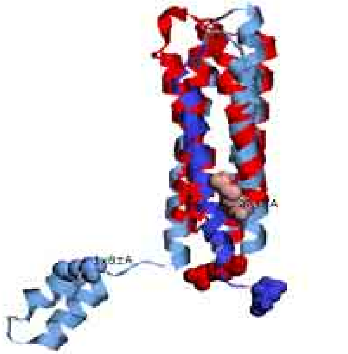
	2gsc:A (114)	conserved hypothetical protein								
1258	2ghw:B (233)	anti-sars scFv antibody, 80R	121	189	3E-49	0.83	1.46	212	26.3% : 59.4% (49/186 : 126/212)	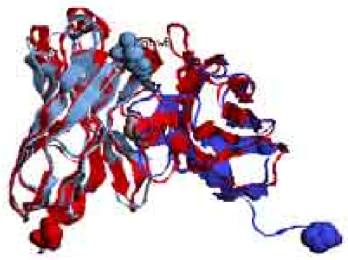
	1x9q:A (232)	4m5.3 anti-fluorescein single chain antibody								
1259	1xe1:A (91)	hypothetical protein PF0907	43	37	0.0009	0.38	2.46	50	16.7% : 16.0% (7/42 : 8/50)	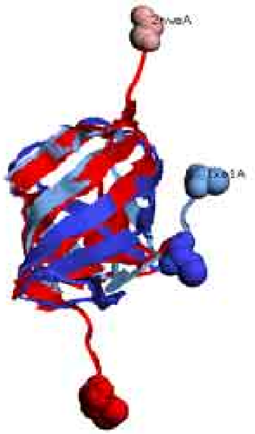
	2nwa:A (81)	Hypothetical protein ytmB								

1260	1z0j:B (51)	FYVE-finger-containing Rab5 effector protein	**25**	32	0.044	0.37	1.91	45	16.3% : 6.7% (7/43 : 3/45)	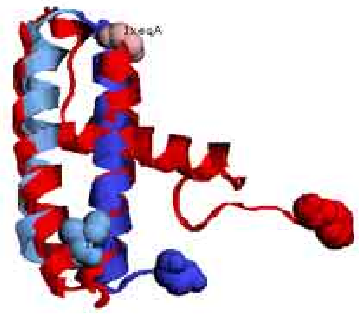
	1xeq:A (89)	Nonstructural protein NS1								
1261	1xg2:B (151)	Pectinesterase inhibitor	**66**	36	0.002	0.37	2.44	107	13.0% : 8.4% (10/77 : 9/107)	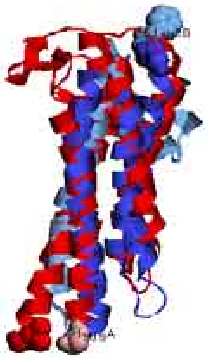
	1yjg:A (157)	Surface protein VspA								
1262	1xg2:B (151)	Pectinesterase inhibitor	**112**	42	4E-05	0.29	2.72	99	8.9% : 13.1% (5/56 : 13/99)	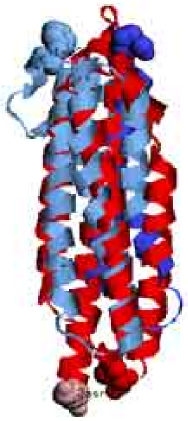
	2asr_ (142)	ASPARTATE RECEPTOR								

1263	2c5i:T (94)	T-SNARE AFFECTING A LATE GOLGI COMPARTMENT PR	**32**	38	0.0005	0.38	3.19	77	4.0% : 6.5% (2/50 : 5/77)	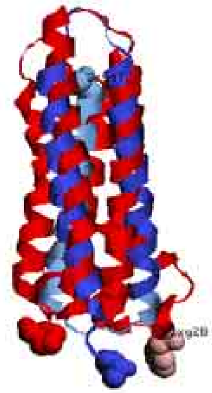
	1xg2:B (151)	Pectinesterase inhibitor								
1264	2d4x:A (214)	Flagellar hook-associated protein 3	**150**	46	3E-06	0.22	1.81	82	12.7% : 3.7% (7/55 : 3/82)	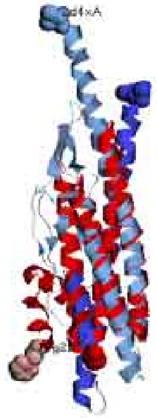
	1xg2:B (151)	Pectinesterase inhibitor								
1265	1xg2:B (151)	Pectinesterase inhibitor	**120**	44	6E-06	0.27	2.85	92	4.1% : 16.3% (3/74 : 15/92)	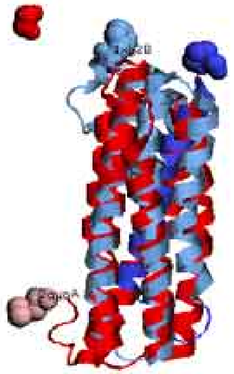
	2gyq:A (162)	ycfI, putative structural protein								

1266	1xou:A<br>(73)<br>2spc:A<br>(107)	EspA<br>SPECTRIN	**42**	48	6E-07	0.39	2.24	52	5.1% : 19.2%<br>(2/39 : 10/52)	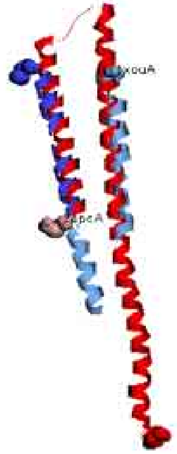
1267	2dav:A<br>(126)<br>1xvs:A<br>(124)	Myosin-binding protein C, slow-type<br>Protein apaG	**58**	32	0.025	0.22	2.71	67	4.8% : 14.9%<br>(3/63 : 10/67)	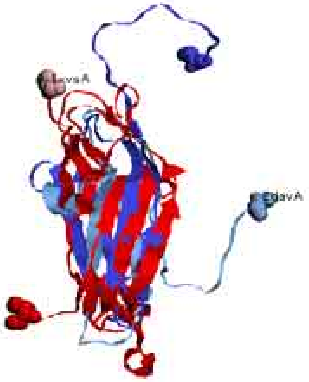
1268	2fz5:A<br>(137)<br>1y5e:A<br>(157)	Flavodoxin<br>Molybdenum cofactor biosynthesis protein B	**79**	31	0.075	0.21	3.00	73	20.3% : 9.6%<br>(14/69 : 7/73)	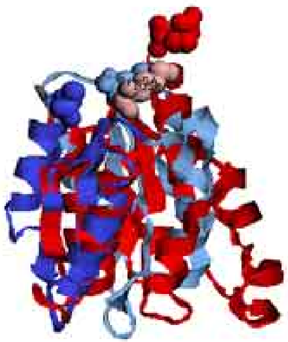

1269	1y74:A (57)	lin 7 homolog b	**26**	31	0.074	0.48	2.62	49	10.6% : 2.0% (5/47 : 1/49)	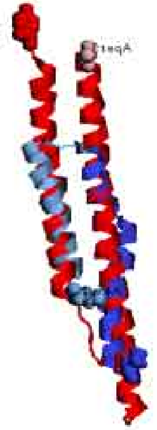
	2ieq:A (89)	Spike glycoprotein								
1270	2asr:_ (142)	ASPARTATE RECEPTOR	**70**	47	1E-06	0.55	3.45	118	11.8% : 10.2% (12/102 : 12/118)	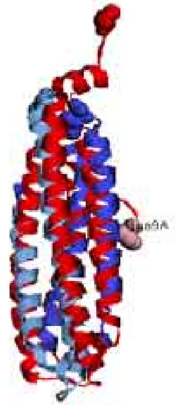
	1ya9:A (169)	Apolipoprotein E								
1271	1yad:A (191)	Regulatory protein tenI	**89**	35	0.005	0.32	3.09	137	14.2% : 11.7% (15/106 : 16/137)	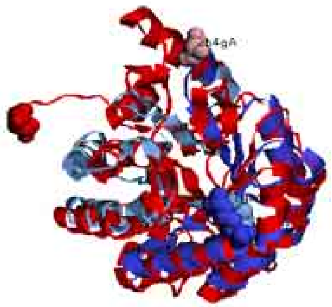
	2b4g:A (312)	dihydroorotate dehydrogenase								

1272	1yad:A (191)	Regulatory protein tenI	80	36	0.004	0.39	2.37	151	9.7% : 19.2% (14/144 : 29/151)	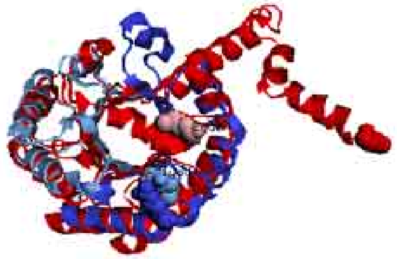
	2dua:A (284)	Phosphonopyruvate hydrolase								
1273	2dle:A (104)	Receptor-type tyrosine-protein phosphatase et	59	35	0.004	0.24	2.54	59	16.1% : 5.1% (9/56 : 3/59)	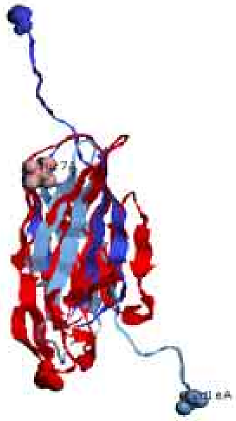
	1yc7:A (116)	anti-VSG immunoglobulin heavy chain variable								
1274	2dtt:A (106)	Hypothetical protein PH0634	35	40	0.0001	0.40	2.68	68	11.4% : 11.8% (5/44 : 8/68)	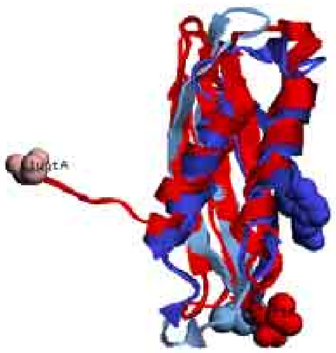
	1ygt:A (104)	Cytoplasmic dynein light chain								

1275	1yjg:A (157)	Surface protein VspA	128	39	0.0003	0.37	3.14	103	3.8% : 12.6% (2/53 : 13/103)	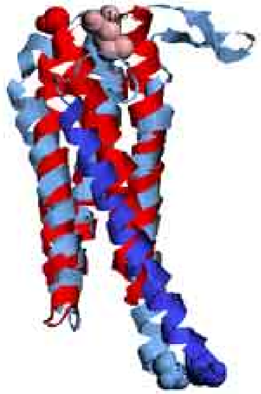
	1yo7:A (120)	Regulatory protein rop								
1276	2c5i:T (94)	T-SNARE AFFECTING A LATE GOLGI COMPARTMENT PR	34	48	4E-07	0.52	2.63	74	3.6% : 2.7% (2/55 : 2/74)	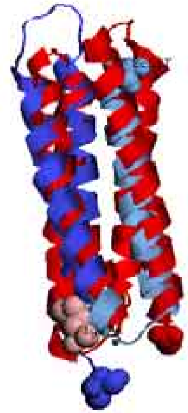
	1yo7:A (120)	Regulatory protein rop								
1277	2ffx:J (173)	ferritin light chain	92	37	0.0009	0.35	2.22	80	10.8% : 12.5% (8/74 : 10/80)	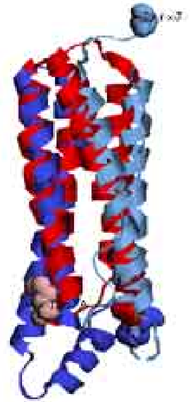
	1yo7:A (120)	Regulatory protein rop								

1278	2huj:A (125)	Lin2004 protein	**96**	37	0.001	0.40	3.04	91	9.1% : 9.9% (5/55 : 9/91)	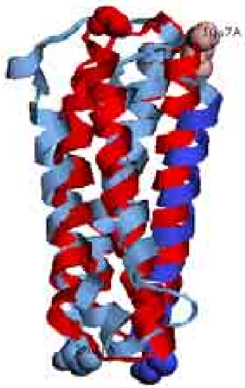
	1yo7:A (120)	Regulatory protein rop								
1279	2ib0:A (142)	CONSERVED HYPOTHETICAL ALANINE RICH PROTEIN	**76**	34	0.009	0.46	1.90	81	3.9% : 3.7% (3/77 : 3/81)	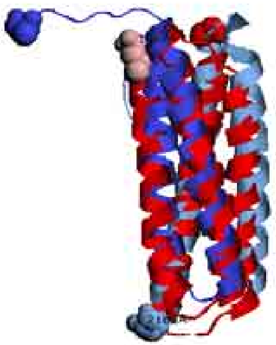
	1yo7:A (120)	Regulatory protein rop								
1280	1ysm:A (55)	Calcyclin-binding protein	**23**	33	0.015	0.37	2.17	48	11.1% : 2.1% (5/45 : 1/48)	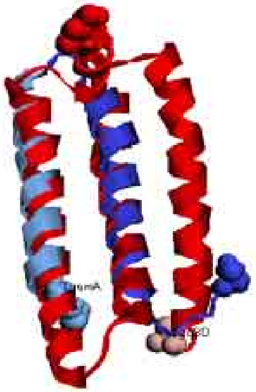
	1yq3:D (102)	SUCCINATE DEHYDROGENASE CYTOCHROME B, SMALL S								

1281	1ysm:A (55)	Calcyclin-binding protein	**19**	34	0.009	0.30	2.24	43	8.8% : 2.3% (3/34 : 1/43)	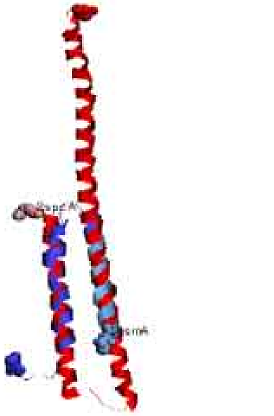
	2spc:A (107)	SPECTRIN								
1282	1z7c:A (165)	Chorionic somatomammotropin hormone	**34**	33	0.018	0.25	2.79	84	10.4% : 9.5% (5/48 : 8/84)	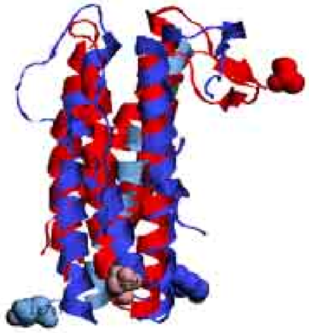
	2ah6:A (152)	BH1595, unknown conserved protein								
1283	1z7c:A (165)	Chorionic somatomammotropin hormone	**116**	31	0.074	0.22	2.94	83	9.8% : 13.3% (6/61 : 11/83)	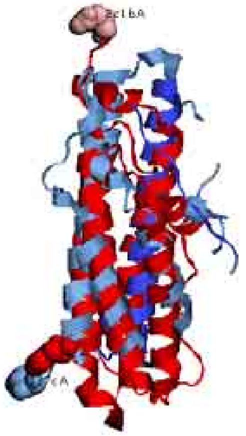
	2clb:A (169)	DPS-LIKE PROTEIN								

1284	1z7c:A (165)<br>2fcw:A (106)	Chorionic somatomammotropin hormone<br>Alpha-2-macroglobulin receptor-associated pro	**98**	33	0.015	0.33	1.21	64	17.5% : 6.3% (10/57 : 4/64)	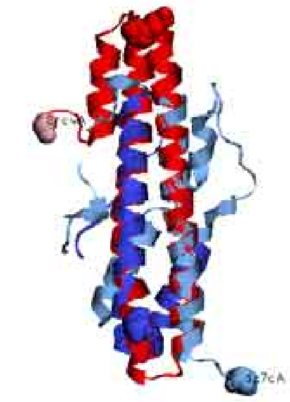
1285	1zh2:A (120)<br>2esr:A (160)	KDP operon transcriptional regulatory protein<br>Methyltransferase	**58**	30	0.089	0.22	3.47	71	12.9% : 9.9% (9/70 : 7/71)	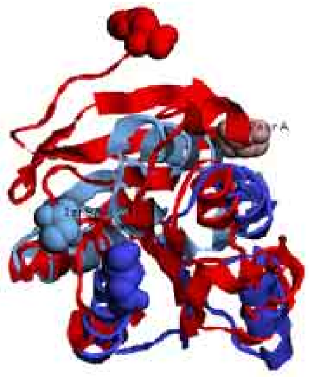
1286	1zke:A (81)<br>2hjd:A (89)	Hypothetical protein HP1531<br>Quorum-sensing antiactivator	**35**	32	0.031	0.44	1.33	55	11.3% : 7.3% (6/53 : 4/55)	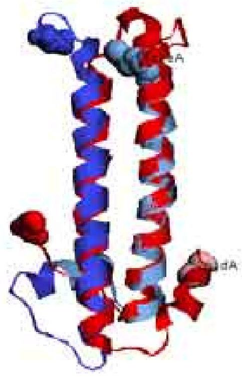

1287	2a10:A (104)	Carbon dioxide concentrating mechanism protei	**35**	39	0.0002	0.28	3.22	68	8.3% : 14.7% (5/60 : 10/68)	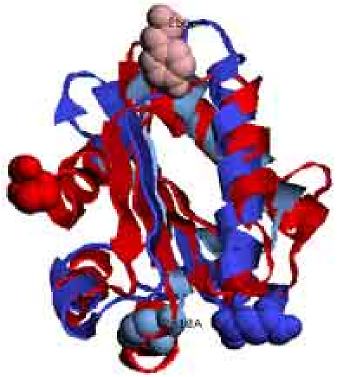
	2b8p:A (130)	Probable nucleoside diphosphate kinase								
1288	2ffx:J (173)	ferritin light chain	**99**	43	2E-05	0.32	2.77	89	8.3% : 9.0% (7/84 : 8/89)	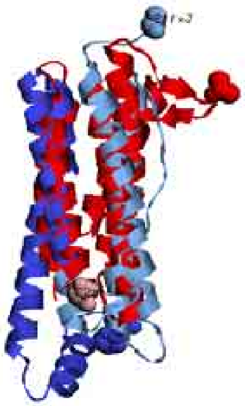
	2ah6:A (152)	BH1595, unknown conserved protein								
1289	2g2d:A (160)	ATP:cobalamin adenosyltransferase	**88**	35	0.004	0.28	3.03	93	10.9% : 7.5% (5/46 : 7/93)	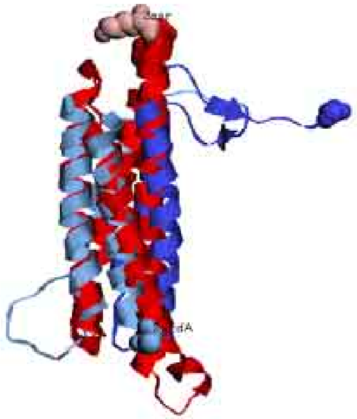
	2asr_ (142)	ASPARTATE RECEPTOR								

1290	2g38:A (77)	PE FAMILY PROTEIN	38	42	2E-05	0.40	2.74	75	7.8% : 5.3% (5/64 : 4/75)	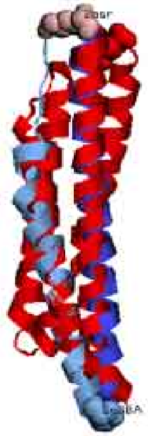
	2asr_ (142)	ASPARTATE RECEPTOR								
1291	2gyq:A (162)	ycfI, putative structural protein	94	40	0.0002	0.29	2.91	90	5.3% : 7.8% (4/75 : 7/90)	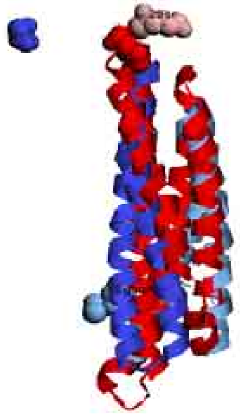
	2asr_ (142)	ASPARTATE RECEPTOR								
1292	2asr:_ (142)	ASPARTATE RECEPTOR	74	61	5E-11	0.58	3.27	131	7.6% : 4.6% (8/105 : 6/131)	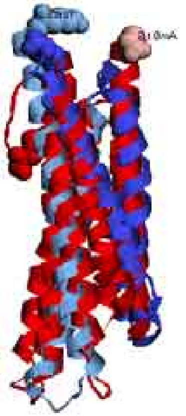
	2i0m:A (210)	Phosphate transport system protein phoU								

1293	2fu2:A<br>(78)	Hypothetical protein SPy2152	**26**	34	0.01	0.34	2.60	64	2.7% : 9.4%<br>(1/37 : 6/64)	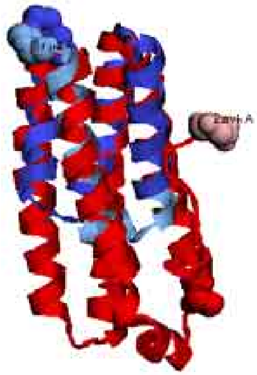
	2avk:A<br>(133)	hemerythrin-like domain protein DcrH								
1294	2avk:A<br>(133)	hemerythrin-like domain protein DcrH	**66**	43	1E-05	0.31	2.68	78	10.9% : 9.0%<br>(6/55 : 7/78)	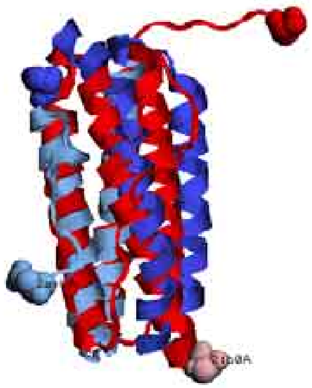
	2ib0:A<br>(142)	CONSERVED HYPOTHETICAL ALANINE RICH PROTEIN								
1295	2d4x:A<br>(214)	Flagellar hook-associated protein 3	**152**	48	8E-07	0.57	2.17	85	12.7% : 5.9%<br>(10/79 : 5/85)	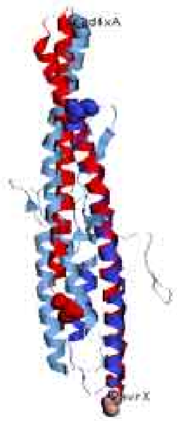
	2avr:X<br>(109)	adhesion A								

1296	2avr:X (109)	adhesion A	**64**	42	3E-05	0.29	2.62	76	8.8% : 7.9% (6/68 : 6/76)	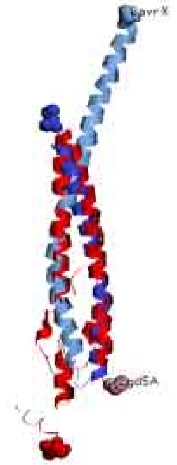
	2gd5:A (143)	Charged multivesicular body protein 3								
1297	* 2b38:A (31)	kalata B8	**27**	42	3E-05	0.48	1.60	30	58.3% : 56.7% (14/24 : 17/30)	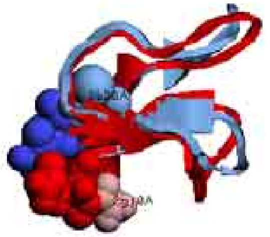
	* 2gj0:A (31)	Cycloviolacin O14								
1298	2ghs:A (294)	AGR_C_1268p	**81**	48	2E-06	0.21	3.48	169	10.1% : 10.1% (14/138 : 17/169)	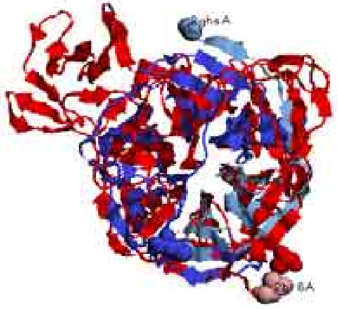
	2bf6:A (448)	EXO-ALPHA-SIALIDASE								

1299	2byg:A (98)	CHANNEL ASSOCIATED PROTEIN OF SYNAPSE-110	**33**	32	0.037	0.35	3.14	74	20.8% : 14.9% (11/53 : 11/74)	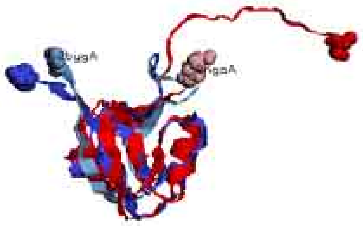
	2hga:A (103)	Conserved protein MTH1368								
1300	2ccv:A (99)	HELIX POMATIA AGGLUTININ	**53**	36	0.001	0.27	2.88	58	11.8% : 10.3% (4/34 : 6/58)	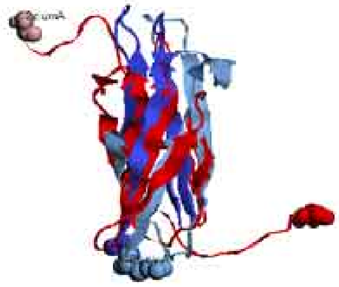
	2cum:A (105)	Tenascin-X								
1301	2gsc:A (114)	conserved hypothetical protein	**40**	40	0.0001	0.32	3.15	73	5.9% : 13.7% (4/68 : 10/73)	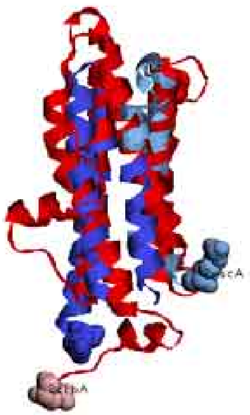
	2clb:A (169)	DPS-LIKE PROTEIN								

1302	2ia9:A (94)	Putative septation protein spoVG	**46**	32	0.036	0.20	2.78	51	11.1% : 7.8% (3/27 : 4/51)	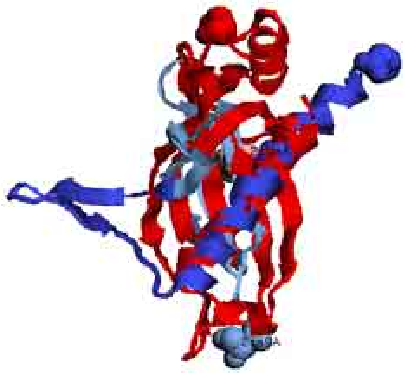
	2cye:A (131)	conserved hypothetical protein, TTHA1846								
1303	2spc:A (107)	SPECTRIN	**45**	63	2E-11	0.35	2.81	73	5.5% : 16.4% (3/55 : 12/73)	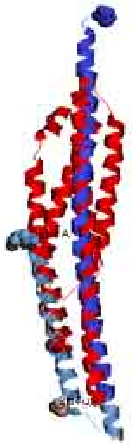
	2d4u:A (167)	Methyl-accepting chemotaxis protein I								
1304	2d4x:A (214)	Flagellar hook-associated protein 3	**168**	44	1E-05	0.36	2.14	93	7.7% : 7.5% (5/65 : 7/93)	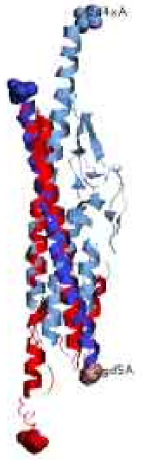
	2gd5:A (143)	Charged multivesicular body protein 3								

1305	2dnx:A<br>(130)<br><br>2fjc:A<br>(151)	Syntaxin-12<br><br>Antigen TpF1	**45**	34	0.01	0.36	3.41	93	7.0% : 9.7%<br>(4/57 : 9/93)	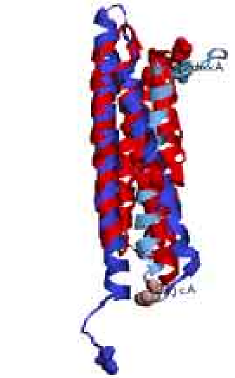
1306	2fe5:A<br>(94)<br><br>2hga:A<br>(103)	Presynaptic protein SAP102<br><br>Conserved protein MTH1368	**31**	31	0.052	0.30	2.17	67	20.4% : 19.4%<br>(10/49 : 13/67)	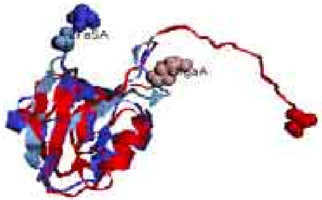
1307	*<br>2ged:A<br>(172)<br><br>*<br>2fh5:B<br>(190)	Signal recognition particle receptor beta sub<br><br>Signal recognition particle receptor beta sub	**42**	64	1E-11	0.41	2.69	133	29.8% : 28.6%<br>(31/104 : 38/133)	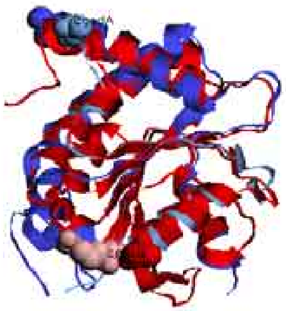

1308	2fne:A (95)	Multiple PDZ domain protein	**29**	35	0.005	0.31	2.13	71	20.0% : 19.7% (11/55 : 14/71)	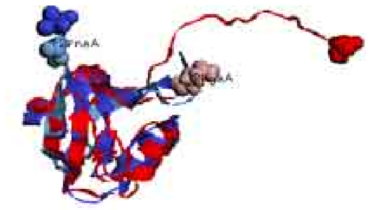
	2hga:A (103)	Conserved protein MTH1368								
1309	2fp8:A (302)	Strictosidine synthase	**164**	50	2E-07	0.37	2.71	185	12.7% : 15.7% (20/157 : 29/185)	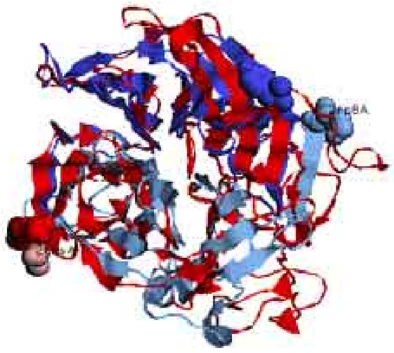
	2g8s:A (347)	Glucose/sorbosone dehydrogenases								
1310	2nt8:A (181)	Cobalamin adenosyltransferase	**108**	34	0.01	0.34	2.43	95	8.7% : 7.4% (8/92 : 7/95)	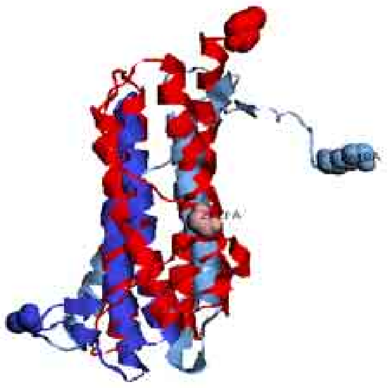
	2fzf:A (158)	hypothetical protein								

1311	2ieq:A (89)	Spike glycoprotein	**55**	37	0.001	0.36	3.33	55	4.8% : 7.3% (2/42 : 4/55)	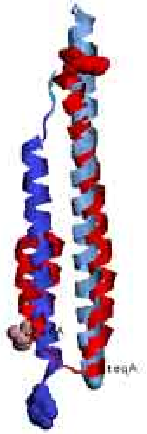
	2fzt:A (78)	hypothetical protein TM0693								
1312	2g8s:A (347)	Glucose/sorbosone dehydrogenases	**216**	40	0.0003	0.31	2.89	182	16.0% : 11.0% (23/144 : 20/182)	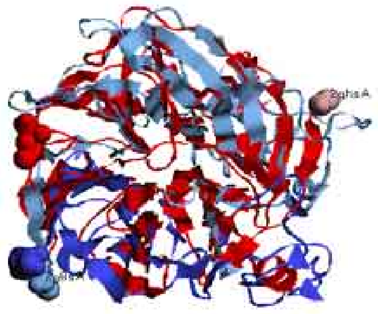
	2ghs:A (294)	AGR_C_1268p								
1313	2ghw:B (233)	anti-sars scFv antibody, 80R	**120**	169	3E-43	0.86	1.13	219	27.7% : 53.4% (52/188 : 117/219)	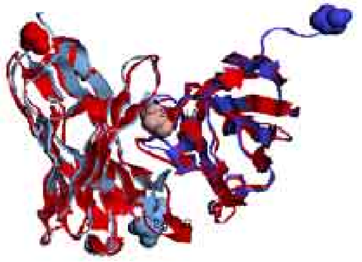
	2gjj:A (237)	A21 single-chain antibody fragment against er								

1314	2hsb:A (126)	Hypothetical UPF0332 protein AF0298	**98**	37	0.001	0.33	3.42	82	4.5% : 11.0% (2/44 : 9/82)	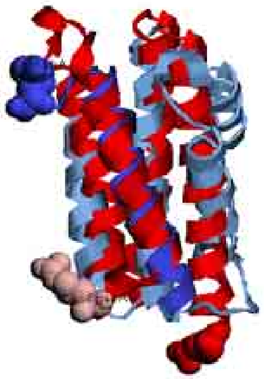
	2gsc:A (114)	conserved hypothetical protein								
1315	2h2b:A (107)	Tight junction protein ZO-1	**37**	36	0.002	0.27	2.17	71	20.8% : 22.5% (11/53 : 16/71)	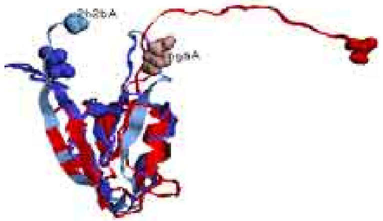
	2hga:A (103)	Conserved protein MTH1368								
1316	2hfi:A (123)	Hypothetical protein yppE	**91**	33	0.021	0.25	3.40	66	5.8% : 7.6% (3/52 : 5/66)	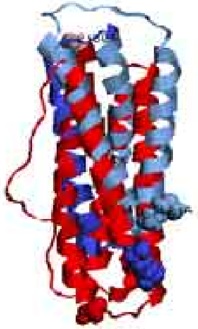
	2ib0:A (142)	CONSERVED HYPOTHETICAL ALANINE RICH PROTEIN								

1317	2ocs:A (86)	Na(+)/H(+) exchange regulatory cofactor NHE-R	**21**	33	0.012	0.33	1.97	69	24.1% : 23.2% (13/54 : 16/69)	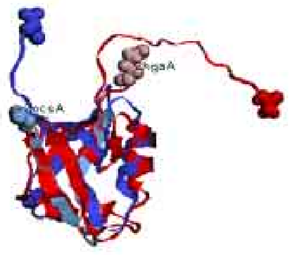
	2hga:A (103)	Conserved protein MTH1368								
1318	2j9l:A (168)	CHLORIDE CHANNEL PROTEIN 5	**92**	34	0.007	0.63	3.03	113	21.3% : 19.5% (23/108 : 22/113)	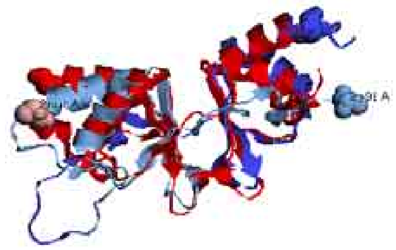
	2nyc:A (132)	Nuclear protein SNF4								

* Engineered CPs, artificial fusion/hybrid proteins or other extensively engineered mutants
