# Supplementary material for: CPSARST: an efficient circular permutation search tool applied to the detection of novel protein structural relationships
Source: Genome Biol. 2008 Jan 18;9(1):R11. doi: 10.1186/gb-2008-9-1-r11 (PMC2395249; doi:10.1186/gb-2008-9-1-r11)
Supplement: Additional data file 5 — Statistical results of protein structural database searches with broad criteria. [file gb-2008-9-1-r11-S5.pdf]

CPSARST – An efficient circular permutation search tool applied to the detection of novel protein structural relationships

Wei-Cheng Lo and Ping-Chiang Lyu

Additional data file 5 – Statistics of protein structural database searches with broad criteria

Description: The criteria of these database searches are RMSD ≤ 6 Å, CP score ≤ 0.1, and permutation size ≥ 10%, which are used for a broader estimation of the natural prevalence of circular permutation.

<table>
<tr><th colspan="3">Database</th><th>nrPDB-90</th><th>nrSCOP-90</th></tr>
<tr><td colspan="3">No. of proteins</td><td>14422</td><td>11688</td></tr>
<tr><td rowspan="5">No. of candidate pairs</td><td colspan="2">1. Detected by amino acid sequence</td><td>8482</td><td>2917</td></tr>
<tr><td colspan="2">2. Detected only by Ramachandran string</td><td>313383</td><td>249153</td></tr>
<tr><td rowspan="3">3. Confirmed after the refinement stage</td><td>Total</td><td>14067</td><td>21809</td></tr>
<tr><td>Symmetric CP</td><td>8656</td><td>13896</td></tr>
<tr><td>Symmetric CP with sequence identity > 30</td><td>129</td><td>102</td></tr>
</table>
