# Supplementary material for: CPSARST: an efficient circular permutation search tool applied to the detection of novel protein structural relationships
Source: Genome Biol. 2008 Jan 18;9(1):R11. doi: 10.1186/gb-2008-9-1-r11 (PMC2395249; doi:10.1186/gb-2008-9-1-r11)
Supplement: Additional data file 7 — Score and E-value ratios calculated from the RCP dataset. [file gb-2008-9-1-r11-S7.pdf]

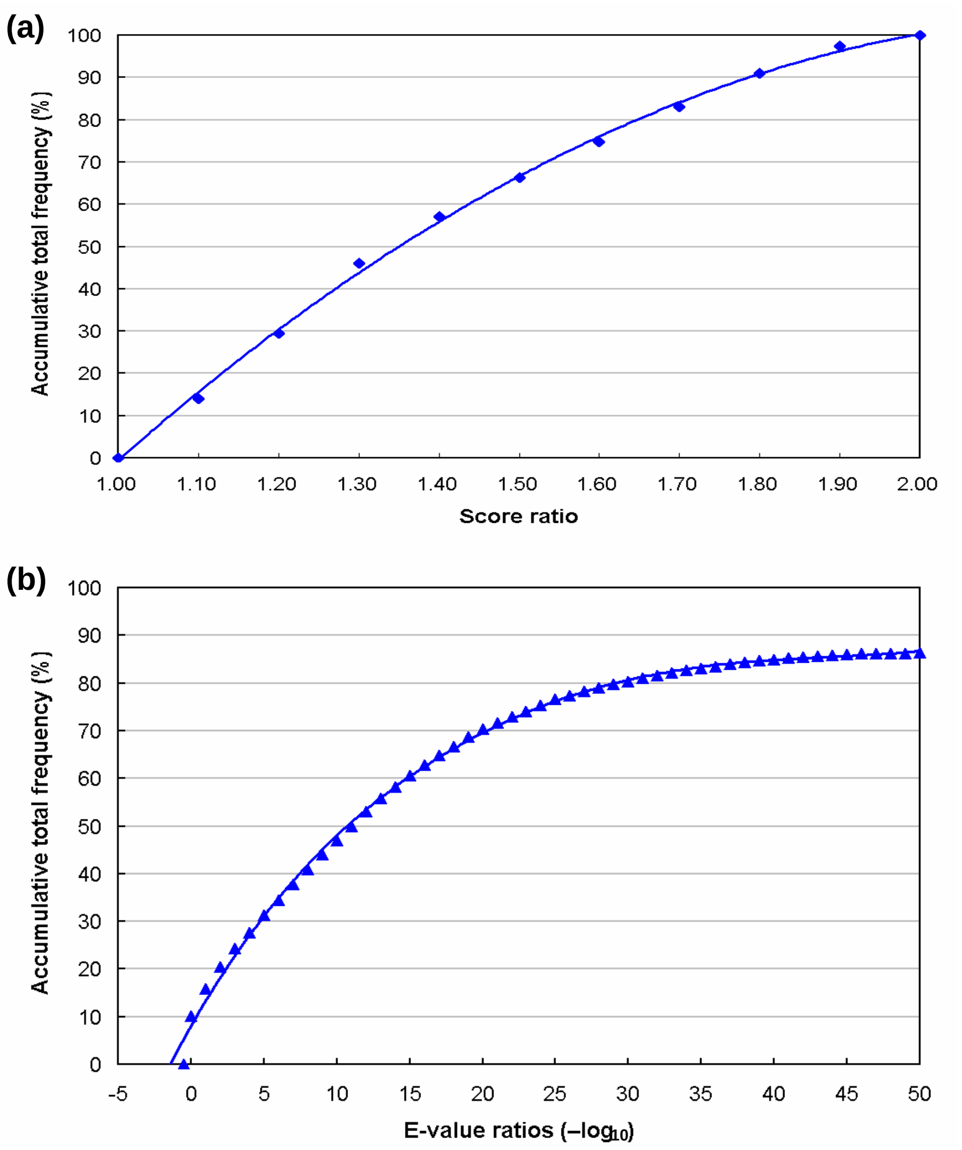

Figure A1. Score and E-value ratios before and after duplicating query sequences. CPSARST detects CP by duplicating the query structural string prior to the database search. This procedure can be applied to amino acid sequences, too. One hundred amino acid sequences were selected to perform *in silico* random circular permutation and 100 different identity levels of regular mutations, leading to 10,000 CP pairs (100 parent sequences × 100 circular permutants; see Methods). Every circular permutant is aligned to its parent sequence with normal length (NL) and duplicated length (DL) by using blast algorithm. Ratios of alignment score ($Score_{DL}/Score_{NL}$) and E-value ($-\log_{10}(Evalue_{DL}/Evalue_{NL})$) were recorded. **(a)** Score ratio–accumulative total frequency curve. All the score ratios are equal to or higher than 1, indicating that when the CP sequence is duplicated, it always aligns to its parent sequence better than the normal length. **(b)** E-value ratio–accumulative total frequency curve. All the $-\log_{10}$ E-value ratios are higher than –0.5. There are only ~20% of the $-\log_{10}$ E-value ratios smaller than 2, implying that 80% of the DL alignments have at least 10^2-fold improvements in the E-value. See Results for the detailed information about E-value. Note that $-\log_{10}$ E-value ratios higher than 50 add up to 13.7% of the total frequency. They are not shown because of the fact that their $Evalue_{DL}$ values are so approaching to zero that they are considered as zero by blast, resulting in the incalculable value: $-\log_{10}0$.
