# Supplementary material for: CPSARST: an efficient circular permutation search tool applied to the detection of novel protein structural relationships
Source: Genome Biol. 2008 Jan 18;9(1):R11. doi: 10.1186/gb-2008-9-1-r11 (PMC2395249; doi:10.1186/gb-2008-9-1-r11)
Supplement: Additional data file 8 — Parameter settings used throughout this article. [file gb-2008-9-1-r11-S8.pdf]

Default settings:

Function	Setting	Note
Searching mode	one-against-all	Alternative: all-against-one[a]
Word size	2	
Gap penalties	G=10, E=3	G: gap-opening; E: gap-extension
Sequence identity improvement	disabled	
Permutation size (S_p) limit	$10\% \leq S_p \leq 50\%$	
Structural similarity improvement	> 25%	
CP score threshold	0.15	
RMSD cutoff	5.0 Å	

[a] All-against-one search is more time-consuming (around 10-fold) but sometimes more sensitive. In a practical database search, "**A** can be used to find **B** (**A➔B**)" does not necessarily mean "**B** can also be used to find **A** (**A🡐B**)" under the same parameter settings. With this "all-against-one" searching mode, where the query and target proteins interchange their roles, "**A⟺B**" can be achieved by using the same parameter settings.

Settings for Figure 3:

Query protein: PDB ID 2B4L, chain A

➔Using default settings expect the gap penalties: G=9, E=2

Settings for Figure 4:

Query protein: PDB ID 1ZBD, chain A

➔Using default settings

Query protein: PDB ID 1PUJ, chain A

➔Using default settings except the searching mode: all-against-one

Settings for Figure 5:

Query protein: PDB ID 1YAD, chain A

➔Using default settings except the S_p limit: $5\% \leq S_p \leq 50\%$

Query protein: PDB ID 2DUA, chain A

➔Using default settings except the searching mode: all-against-one

Settings for Table 1:

Query structure		Retrieved structure		Settings
PDB entry	Chain	PDB entry	Chain	
1AJK	A,B	2AYH		Defaults
1AJO	A,B	2AYH		Defaults
1ALQ		3BLM		Defaults &<br>Structural similarity improvement > 20%
1BD7	A,B	1BLB	C	Defaults &<br>Structural similarity improvement > 10%<br>RMSD cutoff: 16 Å
1CPM		2AYH		Defaults
1CPN		2AYH		Defaults
1FW8	A	3PGK		Defaults
1G2B	A	1SHG		Defaults
1N02	A	2EZM		Defaults
1P5C	A–D	1LW9	A	Defaults &<br>Structural similarity improvement > 15%
1SWF	A–D	1STP		Defaults
1SWG	A–D	1STP		Defaults
1TUC		1SHG		Defaults
1TUD		1SHG		Defaults
1UN2	A	1A2J		Defaults

Settings for Tables 2–4:

➔Using default settings except the S_p limit: 20% ≤ S_p ≤ 50%, and CP score threshold: 0.2.

Settings for Table 5:

Query protein: PDB ID 1PUJ, chain A

➔Using default settings except the searching mode: all-against-one
